# Emergence of a “Cyclosome” in a Primitive Network Capable of Building “Infinite” Proteins †

**DOI:** 10.3390/life9020051

**Published:** 2019-06-18

**Authors:** Jacques Demongeot, Vic Norris

**Affiliations:** 1 Faculty of Medicine, Université Grenoble Alpes, AGEIS EA 7407 Tools for e-Gnosis Medical, 38700 La Tronche, France; 2 Laboratory of Microbiology Signals and Microenvironment, Université de Rouen, 76821 Mont-Saint-Aignan CEDEX, France; victor.norris@univ-rouen.fr

**Keywords:** primitive network, cyclosome, stereochemical hypothesis, small acid-soluble proteins, tRNA synthetases

## Abstract

We argue for the existence of an RNA sequence, called the AL (for ALpha) sequence, which may have played a role at the origin of life; this role entailed the AL sequence helping generate the first peptide assemblies via a primitive network. These peptide assemblies included “infinite” proteins. The AL sequence was constructed on an economy principle as the smallest RNA ring having one representative of each codon’s synonymy class and capable of adopting a non-functional but nevertheless evolutionarily stable hairpin form that resisted denaturation due to environmental changes in pH, hydration, temperature, etc. Long subsequences from the AL ring resemble sequences from tRNAs and 5S rRNAs of numerous species like the proteobacterium, *Rhodobacter sphaeroides*. Pentameric subsequences from the AL are present more frequently than expected in current genomes, in particular, in genes encoding some of the proteins associated with ribosomes like tRNA synthetases. Such relics may help explain the existence of universal sequences like exon/intron frontier regions, Shine-Dalgarno sequence (present in bacterial and archaeal mRNAs), CRISPR and mitochondrial loop sequences.

## 1. Introduction

After the first observations of a ribosome sixty years ago by G.E. Palade and P. Siekevitz [1], theoreticians proposed the first models about the origin of life using tools from statistical mechanics: In 1967, S. Ulam simulated large automata networks and remarked that, with simple growth rules, he obtained complicated patterns similar to those observed in biology [2]. In 1968, inspired by these results, J. Conway started to do similar simulations of cellular automata, in particular a new one called the “Game of Life” by M. Gardner in 1970, because it showed discrete numerical structures moving on a plane and being duplicated [3]. Independently, in parallel, a French team composed of two biologists and one physician-mathematician (J. Besson, P. Gavaudan, and M.P. Schützenberger) worked on the optimality of the genetic code and the existence of primitive RNAs [4] similar to the invariant parts of tRNA loops (see supplementary material in [5]). Unfortunately, Conway’s algorithm did not incorporate realistic genetic considerations in the game that would have made it possible to test, for example, the plausibility of molecular events hypothesized to have led to the appearance of life. In the spirit of such games, we revisit all the above works a half century later by proposing simple RNA structures that could have served as matrices for building first peptides and that constituted what we term here “a cyclosome”.

**Figure 1:**
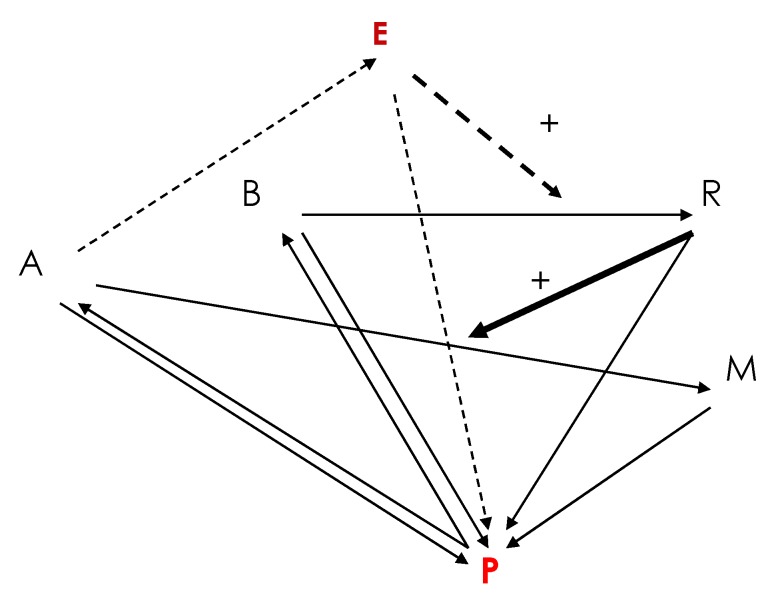
Interaction graph of the genetic network of an autopoiesis model inspired from P. Bourgine and J. Stewart [28] with only activation arrows except the dashed arrow, which can represent either an activation or an inhibition. P (in red) represents the Pool of the elements C, Nand H_2_O, E (in brown) hydrophilic Enzyme peptides, R AL Ring, (**A**) Amino acids, (**B**) nucleotide Bases, and M hydrophobic Membrane peptides.

Since 1984, many papers, including in particular those of Yonath and collaborators, have explored the possibility of a chain of ancestors of the present ribosomal molecules and have proposed for these primitive structures the name of “protoribosome” [6,7,8,9,10,11,12,13,14,15,16,17,18,19,20,21,22,23]. More recent papers have similarly revisited the evolution of the tRNA and aminoacid-tRNA synthetases (or ligases) in the ribosomal ecosystem [24,25]. In the present paper, we try to complement these structural approaches by a pure informatics approach showing that from simple realistic constraints, sequences and secondary RNA structures can be found with selectable properties of resistance to denaturation and of storage of the genetic code. We start the corresponding “game of life” in Section 2 by searching for a realistic primitive genetic network regulating the dynamics of the first actors involved in making peptides. First, we designate an RNA ring, termed AL for ALpha, as the central actor, and then we search for AL candidates in rings having a minimal length and possessing one and only one codon from each synonymy class of the genetic code; these requirements are chosen in order to favor a stereochemical neighborhood of the AL rich in amino acids, and hence favor the synthesis of an ‘infinite’ (theoretically endless but randomly cleaved by environmental factors) protein that would have allowed evolution to select a great many peptides and proteins from the making and subsequent breaking of peptide bonds with a degree of randomness. In order to ensure the survival of the AL in the absence of amino acids and hence in the absence of a selectable function, we ask more of the AL, namely, that it should be able to adopt a hairpin structure that, optimally, would be as stable as possible, have the smallest head (three nucleotides) and the longest tail (9 pairs of nucleotides). In Section 3, we search amongst those species that have two or more types of short RNA sequences (e.g., 5S and 29S ribosomal RNA and the loops of transfer RNA) for those that share with the AL at least one nonameric (9-mer) sequence consistent with their co-evolution. We report in Section 3 those species that satisfy this constraint, e.g., a proteobacterium, *Rhodobacter sphaeroides* (Figure 1A,B). By combining these sequences, we construct a circular AL ring with 22 nucleotides and then search for the most stable hairpin with the same sequence as the AL in large genetic data bases with repeated motifs from the AL, namely pentameric subsequences. Then, in Section 4, we look for AL relics in current Archaea genomes and in some ancient structures like ribozymes. Finally, in Section 5, we propose how the AL arose from catalysis by interfaces between membrane domains and how the AL may have generated “infinite” proteins as part of its role in the evolution towards a “protein-synthesizing machine” in its own right (perhaps ‘the ancestral protein-synthesizing machine’) that we term a “cyclosome”. The latter was facilitated by the production of the first nucleo-peptide conjugates as shown by the frequency of the pentameric relics of the AL which serves as a scalar for proximity to AL.

## 2. A Primitive Network at the Origin of Life

In our hypothesis, amino acids were concentrated around the AL, which acted as a “proto-nucleus” to allow the first “organ” or “cyclosome” to synthesize peptides. Insofar as an object corresponds to a discontinuity in a field of connectivity [26], the boundary of this cyclosome corresponded to a discontinuity in the gradient of peptides around the AL.

The boundary of the first functional “machine” able to build peptides can be defined as a peptide gradient boundary centred on the “proto-nucleus” AL, resulting from an amino acid confinement around the AL favoring the occurrence of peptide bonds. This “organ” functioned as a “cyclosome” in a “proto-membrane”, thus as a “proto-cell” with a circular organization. This proto-cell is a solution to the problem of how to obtain autopoiesis: Peptide synthesis favored by the AL was necessary to repair the proto-cell membrane made of hydrophobic peptides and lipids, which reciprocally protected the AL against denaturation by ensuring the integrity of the proto-nucleus. The autopoiesis network underlying this organization has been studied in [27,28,29] and exhibits exponential growth if the peptide proto-membrane allows the entry of nucleic acids for AL replication. We can represent its dynamics by defining the variables of the network and their interactions using a system of differential equations (1) whose Jacobian graph is given in Figure 1: Let us denote by R, A, B, E, M, and P for, respectively, the concentration of AL Ring, Amino acids, nucleotide Bases, hydrophilic Enzyme peptides, hydrophobic Membrane peptides, and the Pool of lipids plus the elements C, N, and H_2_O:

dR/dt = d_R_∆R + k_B_B − k_R_R
dA/dt = d_A_∆A + k_P_P − k_A_RA − k’_E_RA
dB/dt = d_B_∆B + k’_P_P − k_B_B
dE/dt = d_E_∆E + k’_E_RA − k_E_E
dM/dt = d_M_∆M + k_A_RA − k_M_M
dP/dt = d_P_∆P + k_R_R + k_M_M − k’_P_P − k_P_P (1)

In the absence of diffusion (diffusion coefficients d_i_’s equal to 0), the differential system (1) has an initial exponential growth behaviour and tends, if P is constant and initial values of variables are not zero (which corresponds to an unstable steady state), towards the unique stable stationary state:

(R*,A*,B*,E*,M*) = (K/k_R_, K’k_R_/K(k_A_ + k’_E_), K/k_B_, K’k’_E_/k_E_(k_A_ + k’_E_), K’k_A_/k_M_(k_A_ + k’_E_)) (2)

with the following Jacobian matrix J* equal to:
−k_R_0k_B_00−K’k_R_/K−K(k_A_+k’_E_)/k_E_00000−k_B_00K’k_R_k’_E_/K(k_A_+k’_E_)Kk’_E_/k_R_0−k_E_
K’k_R_k_A_/K(k_A_+k’_E_)Kk_A_/k_R_00−k_M_
whose characteristic polynomial (k_R_ + λ)(K(k_A_ + k’_E_)/k_E_ + λ)(k_B_ + λ)(k_E_ + λ)(k_M_ + λ) = 0 has only negative eigenvalues, ensuring the stability of the stationary state (2) (R*,A*,B*,E*,M*). If we add a diffusion term for the different metabolites, this dynamics leads to the spatial segregation of R, A, B, M, and P into structures like a protein-synthesizing machine made of the AL or the anti-sense AL (R), proto-cytoplasm (A, B), proto-membrane (M), and building blocks (P). The AL serves as a template for the formation of hydrophilic enzymatic peptides E (Figure 1) able to activate (or inhibit depending on their catalytic properties) the AL or anti-sense AL replication with a reaction constant k_r_ [30]. If we introduce diffusion processes (whose viscosity coefficients depend on the membrane concentration M) in the purely reaction differential system, we get the diffusion-reaction equations (1), for which a close discrete analogue has been already simulated in [31,32], and which shows a progressive space segmentation by the M gradient.

During its exponential growth and diffusion, the boundary of the system (1) is chosen as the gradient boundary of peptides polymerized from amino acids. Growth stops in the case of a lack of nucleic acid or protein precursors, i.e., because of the disappearance of the elements of the C, N, and H_2_O pool (which provides the amino acids and nucleotides that are consumed during the growth).

## 3. Construction of the AL RNA Ring

We have shown by using constraint programming and a step-by-step computation [33,34] that only 25 RNA rings satisfy the following constraints:

- All dinucleotides should appear at least once (apart from CG because of CG suppression).
- Among rings satisfying the constraint “to be as short as possible and contain at least one codon of each amino acid synonymy class”, there is no solution for a length below 22 nucleotides. For length 22, 29,520 solutions contain the codon AUN twice, N being G for 52% of the solutions.
- From the 29,520 solutions, only 25 rings allow the formation of a hairpin at least 9-bases long.
- Of these 25 rings, 19 have both start and stop codons.
- Through calculation of the average genetic distances to the others (e.g., circular Hamming distance, permutation distance, and edit distance), one singular ring exhibits a minimum distance as compared to the others. Only one sequence, called AL (for ALpha) is thus acting as the barycenter of the set of the 18 others: 5′-AUGGUACUGCCAUUCAAGAUGA-3′.

**Figure 2:**
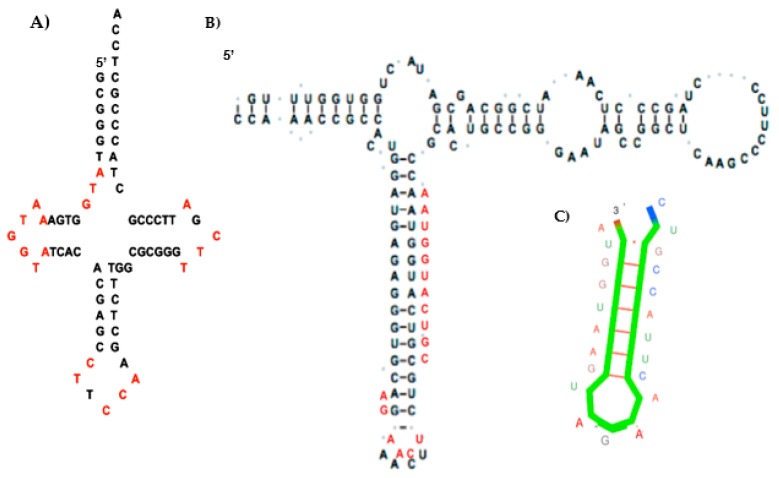
(**A**) AL subsequences (in red) ATG, AATGGTA, CT, and CCATTC from the loops of the Gly-tRNA^TCC^ of *Rhodobacter sphaeroides*; (**B**) AL hemi-sequence AAUGGUACUGC (in red) and AL-hexamer UCAAGA (in red) from the hairpin of the 5S rRNA of *Rhodobacter sphaeroides* (adapted from [35]); (**C**) Optimal hairpin form for AL (from Kinefold [36]).

Then, we remark that AL appears by merging the following sequences of the genome of *Rhodobacter sphaeroides* (Figure 2): AATGGTACTTCCATTCGATATG from the Gly-tRNA^TCC^ loops, AATGGTACTGCGTCTCAAGACG from 5S rRNA [35].

It is possible to design, by using the Kinefold^®^ algorithm [36], the most thermodynamically stable hairpin (Gibbs free energy equal to ∆G = −9.5 kcal/mol in Figure 2) among the 22 RNA chains obtained from the circular permutations of AL (Figure 2C). This structure could explain why, during denaturation, there is first a loss of the AL-hexamer CUGCCA (anticodon loop of current Gly-tRNA^GCC^s) and then a break between AL-heptamers UUCAAGA (the T_Ψ_-loop of current tRNAs) and AAUGGUA (the D-loop of current tRNAs). An argument in favor of this scenario is the distribution of the pentamer frequencies inside the current genome (from Rfam database, http://rfam.xfam.org/), which shows the two highest survival probabilities for the AL-pentamers coming from the most stable part of AL, also parts of the D-loop and T_Ψ_-loop of the present tRNAS, i.e., AAUGG, AUGGU, UGGUA, GGUAC, TTCAA, TCAAG, and CAAGA. If we consider other subsequences of AL, we find many repeated motifs, such as AATGG [37] and GATG [38] from human microsatellites, AGAT from vertebrate repeated UTR motifs [39], and CCATTCA from the Alpha Satellite of Human Chromosome 17 [40] and from the HMG box (High Mobility Group Box, a protein domain involved in DNA binding [41]), as well as the optimal codons that determine mRNA stability in the yeast genome [42].

We can generalize the result obtained from *R. sphaeroides* to other archaeal, bacterial, and eukaryotic species as shown in Table 1. The genetic code consists of 64 triplets made of 3 letters representing purine bases—A for Adenine and G for Guanine—and pyrimidine ones—U for Uracil and C for Cytosine—that can be grouped into 21 synonymy classes.

**Figure 3:**
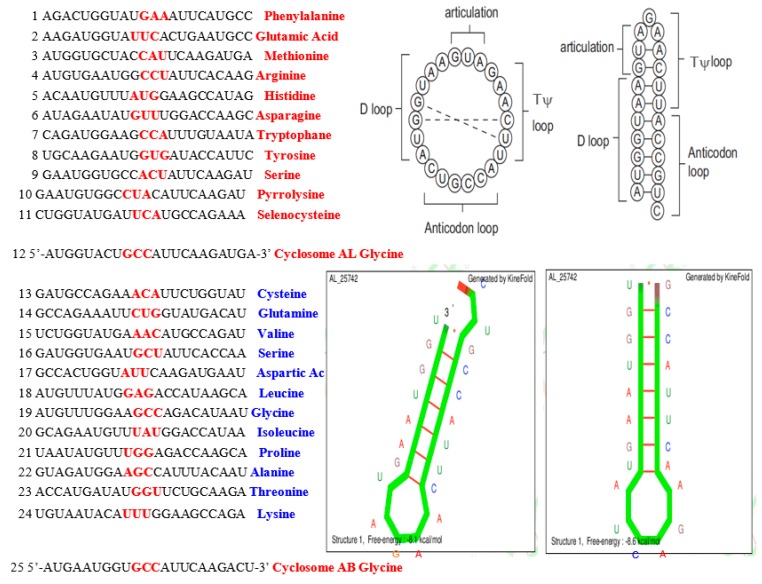
Left: The 25 RNA sequence candidates for the archetypal tRNAs that bound the 22 amino acids. Top Right: The circular and hairpin forms of the Archetypal Loop AL proposed as the “cyclosome”. Bottom Middle: A hairpin form of AL. Bottom Right: The most stable hairpin form of the Archetypal Bound AB proposed as a variant of the cyclosome AL.

Each class contains between 1 and 6 triplets; 20 classes correspond to the 20 amino acids (except for one class containing only 1 triplet, which corresponds either to the amino acid Methionine or, if this triplet initiates a sequence of messenger RNA (mRNA), to a “start” punctuation symbol), plus one class corresponding to the “end” punctuation symbol terminating the mRNA sequences. It has been shown that stereochemical bonds can favor a non-permanent, reversible link between amino acids (AA) and codons or anticodons of their AA synonymy class [43,44,45,46,47]. The 25 selected rings satisfy two opposite constraints corresponding to a min-max problem: (i) to be as short as possible, and (ii) to contain one and only one triplet corresponding to each amino acid synonymy class. The latter constraint would allow the rings to serve as a “matrimonial agency” concentrating amino acids in the vicinity of the ring and thereby favoring the links between any pair of them via peptide bonds [48,49,50,51,52,53,54,55]. The 25 RNA rings selected can be considered as ancestors of the tRNA of the 22 AAs including Pyrrolysine and Selenocysteine (Figure 3), with Serine counted twice, and Tyrosine and Aspartic Acid able to replace C by U in their tRNA anticodons [56,57].

The 12 rings in red in Figure 2 could correspond to an intermediary genetic code using the wobble mechanism present in Archaea [58,59,60] and many other organisms [61,62]. The AL ring (resp. AL’ anti-ring) selects and confines more L-aminoacids (resp. D-aminoacids) and catalyses the synthesis of either hydrophobic or hydrophilic peptides [63,64,65]. We can note that in [9] peptide synthesis was achieved experimentally by using as RNA template a heptameric subsequences of AL, AAUGGU.

## 4. Nucleo-Nucleic and Nucleo-Peptidic Mechanisms

Different intracellular mechanisms involving RNA, DNA, and proteins conserve as relics subsequences of AL, in particular from its short hairpin ATTCAAGATGAAT.

### 4.1. tRNA Loops

**Figure 4:**
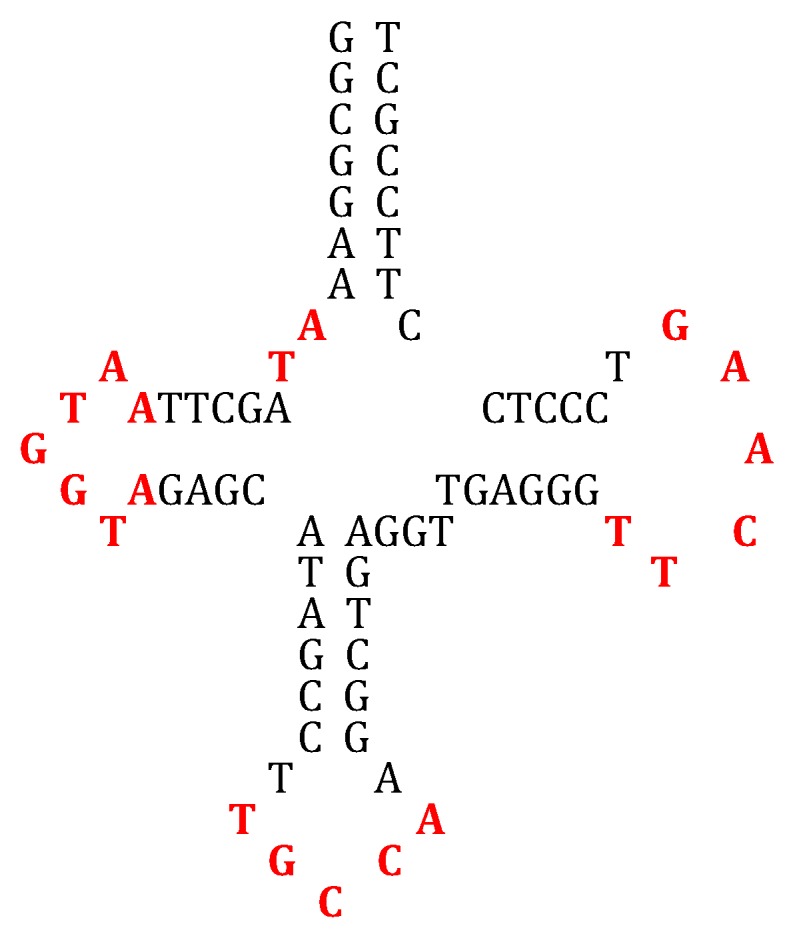
GlytRNA^GCC^ of *Lupine* [66], whose loops (articulation, D-, anti-codon, and T_Ψ_-loops) fit AL almost perfectly with the sequence formed by its loops (in red).

tRNA loops (D-loop, anti-codon loop, T_Ψ_-loop, and articulation loop) form a sequence that has many similarities to AL. For example, loops of mitochondrial GlytRNA^GCC^ of *Lupine* [46] fit AL almost perfectly (Figure 4) and this tRNA exists in 242 species in the NCBI Nucleotide database [59].

In the tRNADB-CE database, a high percentage of tRNAs have loops that fit the AL, with TGGTA in D-loop and TTCNA in T_Ψ_-loop among tRNAs with NTGCCAN as the anticodon loop (Table 2).

### 4.2. Giant Viruses

The hypothesis that de novo template-free RNAs appear spontaneously—as at the origin of life—and invade modern genomes (in particular those related to the giant viruses) is based on their resemblance to the 25 putative ancestors of the present tRNAs (cf. Figure 3 and [67,68]). Moreover, the AL-pentamers are often observed in the sequences of the giant viruses. To quantify the frequency of the AL-pentamers, we define an AL-proximity frequency for a given genome as the percentage of occurrence in this genome of the 9 most frequent pentamers from the AL (Table 2): If this genome contains 1,000,000 nucleotides, the percentage of such occurrences supposed to be random equals 0.88 ± 0.016* (* for the 90%-confidence interval). From calculations using the NCBI nucleotide database [67,68,69], the AL-proximity of the complete genome of numerous giant viruses are: Mega 1.82, Mega Chilensis 1.72, Tupan 1.65, Moumou 1.91, Pitho 2.19, Fausto 1.50, Marseille 1.39, Senegal 1.54, Mimi 2.20, Mama 1.90, Bodo 1.81, Samba 1.89, their mean value being equal to 1.80 and that of their virophages Sputnik and Zamilon to 2.23 (see Supplementary Material 1 for other virophages).

### 4.3. Circular RNAs

The 3801 human circular RNAs from circBase [70] observed after the first discovery of circular RNAs in many organisms [71,72] contain 36228352 possible pentamers; the number of AL-pentamers from a branch of its hairpin form are given in Table 3, which significantly exceeds the number obtained at random.

### 4.4. Ribozymes

An RNA catalytic domain has been found within the sequence of the 359 base long negative-strand satellite RNA of tobacco ringspot virus [73]. The catalytic domain contains 2 minimal sequences of satellite RNA, a 14-base substrate RNA, and a 50-base catalytic RNA containing 2 AL-pentamers:

5′-AAACAGAGAAGUCAACCAGAGAAACACACGUUG**UGGUA**UAUUACC**UGGUA**-3′

A minimal RNA hairpin ribozyme discovered 18 years later [74] shows an interesting catalytic activity due to its chain D with 3 AL-pentamers present in its 19 bases: 5’-UCG**UGGUAC**AUUAC**CUGCC**-3’. The AL-tetramer UGGU is generally not cleavable by ribozymes [75], this empirical fact explaining its survival in present ribozymes. AL–pentamers can also be found in the D Chain of many other hairpin ribozymes [76,77,78,79,80,81,82,83], used to build simple RNA systems, consisting of two ribozymes with concerted activity allowing replication [84].

### 4.5. Intron-Exon Frontier

**Figure 5:**
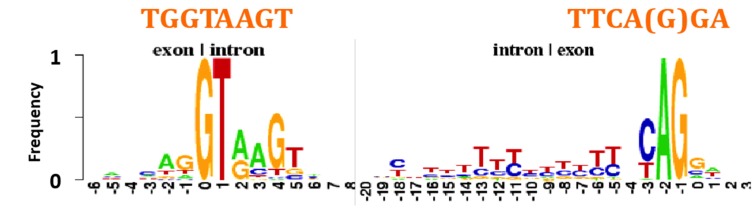
Consensus sequences at exon/intron and intron/exon boundaries in eukaryotes (after [85]).

The heptamers GGTAAGT and TTCA(G)GA present in AL ring are observed frequently at the frontiers of, respectively, exon/intron and intron/exon in genome of many organisms (Figure 5) [85].

### 4.6. Synthetases

Using the AL-proximity calculated from the 9 most frequent pentamers from the AL ring (Table 3), glycyl-tRNA synthetases from [69] have a value more than the 95%-confidence upper threshold, which is equal to 0.88 + 0.49 = 1.37* (calculated for a sequence of size 1000).

**Figure 6:**
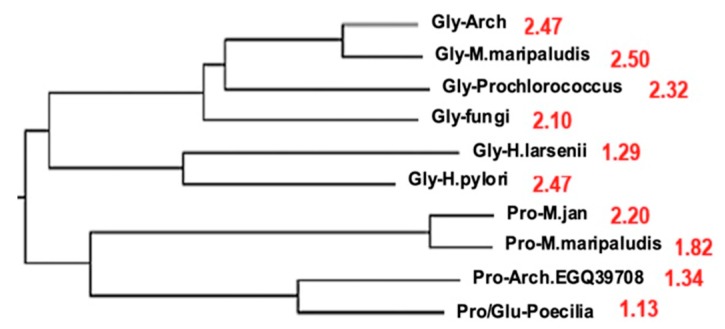
Clustering tree of the synthetases of certain microorganisms based on their sequence resemblance (for the Hamming distance) (after [93]) with indication (in red) of AL-proximity.

Table 4 and Figure 6 show the values of the AL-proximity (for the 9 most frequent AL pentamers) for the tRNA synthetases of the different microorganisms studied in [86,87,88,89,90,91,92,93], especially Bacteria, Archaea, and one Fungus. We observe that Archaea have the maximal values of this proximity and by comparing the sequences of these synthetases [93], we see that the clustering tree based on the sequence resemblance (for the Hamming distance) described as narrow are synthetases having similar values of their AL-proximity (except the pair Haloferax larsenii / Helicobacter pylori).

The Table 5 gives the values of the AL-proximity for different tRNA synthetases (called also tRNA ligases) and ribosomal or transfer RNAs, showing that the 40S ribosomal RNAs; tRNA synthetases; and 60S, 18S, and 16S ribosomal RNAs have, in this order, decreasing proximities to the AL. The high values of the AL-proximity for the synthetases are consistent with a very early role in a protein-synthesizing machine, by increasing the efficacy of amino acid binding to an RNA-oligopeptide complex such as the AL ring coupled with ligases.

### 4.7. Small Acid-Soluble Spore Proteins (SASPs)

SASPs are DNA-binding proteins protecting the DNA backbone from chemical and enzymatic cleavages. Calculating their AL-proximity for all the 22 pentamers of AL (the value in the case of random occurrences of pentamers being equal to 2.1 ± 1.4*) for 100 Bacteria and Archaea randomly chosen in the NCBI database (see Supplementary Material 2) shows that 100% of them have values of AL-proximity over the 95%-confidence upper threshold 2.4*, the mean value being 4.49.

### 4.8. Defence Mechanisms

The CRISPR-CAS system provides bacteria like *Streptococcus agalactiae* with adaptive immunity and the AL-pentamers ATGGT and ATTCA, and AL-hexamers AATGGT and TCAAGAT (corresponding respectively to the D-loop and Tψ−loop of many ^t^RNAs) are often found at many levels of the system (CAS proteins, Casposon TIR and CRISP repeats [94]), e.g., typical repeat sequences for CRISPR1 and CRISPR3 [95] contain AL-heptamers shared by AL and tRNA loops:

GTTTTTGTACTCTCAAGATTTAAGTAACTGTACAAC (CRISPR1)

GTTTTAGAGCTGTGTTGTTTCGAATGGTTCCAAAAC (CRISPR3),

as well as the sequences of TIR and CRISPR compared in [96,97], a consensus sequence from central part of the murine RSS VκL8, Jß2.6, and Jß2.2 [98,99,100], and human RSS spacer common for Vh, V328h2, and V328 [101,102,103] described in Table 6 and also the protein H354 of Mimivirus kasaii [104] (see Supplementary Material 3 for calculations of AL-proximity of CRISPR-CAS proteins).

The probabilities of a match between the AL and sequences of elements involved in defence mechanisms, at different levels of evolution, are the following:

p = 2.10^−9^ for 19 matches (with an insertion) between TIR and CRISPR using the binomial distribution B(1/4,22), p = 8.10^−6^ for 15 anti-matches between AL and CRISPR plus 1 quasi-anti-match G-T using the distribution B(1/4,21) × B(3/8,1),
p = 7.10^−4^ for 13 matches between AL and consensus RSS using the binomial distribution B(1/4,22),
p = 2.10^−6^ for 11 matches between AL and RSS spacer using the binomial distribution B(1/4,12).

In Mimivirus, a mechanism similar to CRISPR has been discovered [104], which involves two exonucleases R 350 and R 354 having respectively 3.02 and 3.71 as the values of the AL-proximity:

- Acanthamoeba castellanii mimivirus DNA, nearly complete genome, strain: Mimivirus kasaii GenBank: AP017644.1 457483-459936 R 350 Lambda-type exonuclease, with AL-proximity **3.02**;
- Acanthamoeba castellanii mimivirus DNA, nearly complete genome, strain: Mimivirus kasaii GenBank: AP017644.1 462878-464527 R 354 Lambda-type exonuclease, with AL-proximity **3.71**.

Moreover, the sequences of the genes of these exonucleases contain numerous heptamers like:

5′-**GATGATGAAGATGATGATGAAGAT**-3′ (MIMIVIRE gene H354).

### 4.9. Mitochondrial D-loop

**Figure 7:**
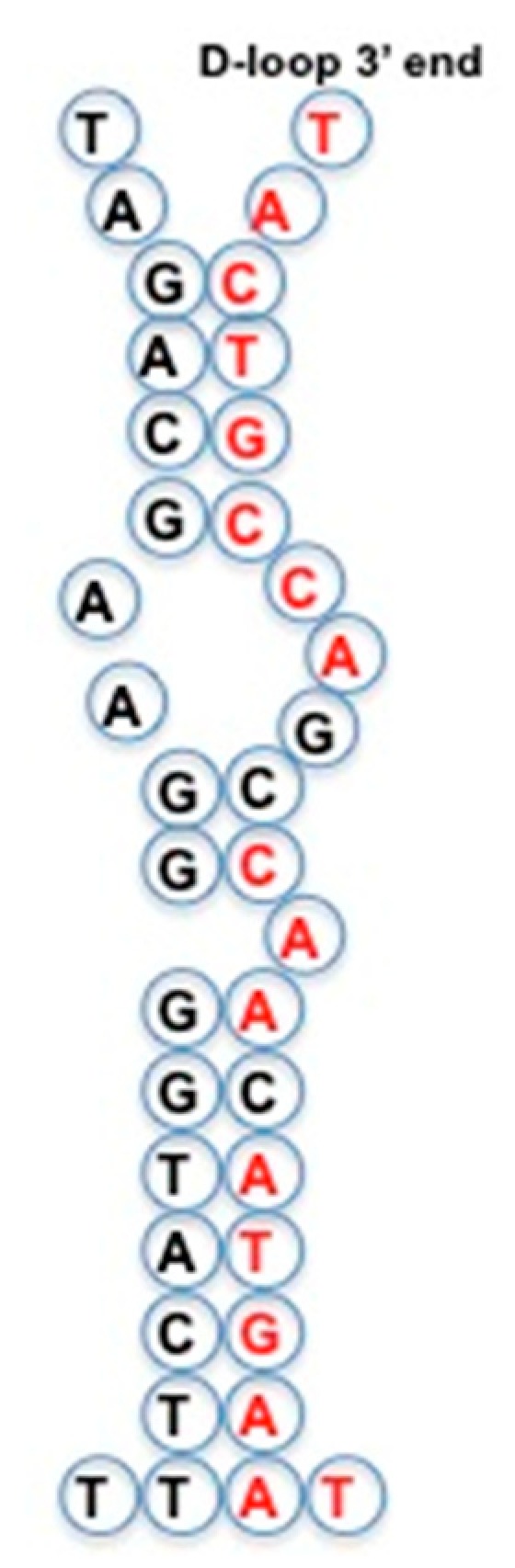
Mitochondrial D-loop (7S mtDNA) with its central AL-octamer and hexamer: TACTGCCAGTCAACATGAAT.

In [105], the 2D-structure of the mitochondrial D-loop (7S mtDNA) is given with its central AL-octamer and hexamer TACTGCCAGTCAACATGAAT and in false colour the frequency of its bases (Figure 7). This D-loop is conserved among different species and contains putative mitochondrial micro-RNAs, called mito^2^miRs in [106].

### 4.10. 5S Ribosomal RNAs

5S ribosomal RNAs are components of the ribosome. Calculating their AL-proximity for all the 22 pentamers of the AL (the value in the case of random occurrences of pentamers being equal to 2.1 ± 1.4*) for 100 Bacteria and Archaea randomly chosen in the NCBI database (see Supplementary Material 4) shows that 78% of them have values of the AL-proximity over the 95%-confidence expected upper threshold of 2.4*, their mean value being equal to 4.01.

### 4.11. Cytidine Deaminases

The AID/APOBEC protein family comprises cytidine deaminases capable of deaminating cytosine to uracil in the context of a single-stranded polynucleotide [107], met primitively in yeast, and after in fishes, birds, amphibians, and mammals: They play a role of RNA-editing enzymes, contributing to the co-evolution of viruses and their antibodies [108], then perhaps initially to the co-evolution of first RNAs. Among 50 members of this family given in [107] (see Supplementary Material 5), 96% of them have values of the AL-proximity over the 95%-confidence expected upper threshold of 2.3*, their mean value being equal to 3.36.

## 5. Discussion

### 5.1. Origins of the AL Ring

**Figure 8:**
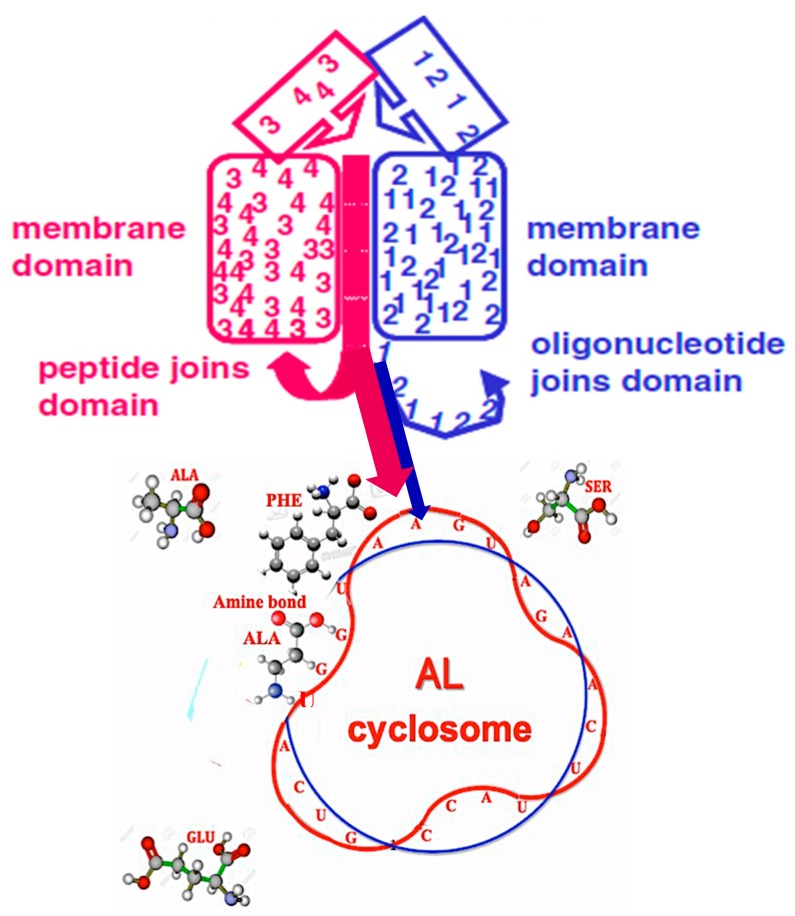
Combination of lipid interfaces mechanism and the functioning of AL as a protein-synthesizing machine without the whole ribosomal machinery.

The sequence of the AL ring was obtained by trying to satisfy the constraints of both being as short as possible and being long enough to encode all the amino acids. A justification for the former constraint can be found in the ‘lipid world’. Even membranes composed of a single species of molecule can have domains in gel and fluid phases whilst membranes composed of different molecules contain many, predominantly small, domains [109]. We have proposed that such interfaces catalysed the polymerisation of both RNAs and amino acids [110]. In this scenario, in which there would have been many very small domains with closed loop interfaces, there would have been a correspondingly greater production of small RNA rings (Figure 8).

A justification for the second constraint can be found in the hypothesis that interaction between amino acids and nucleotides stabilised both species thereby leading to their accumulation in the abiotic flux of molecular creation and destruction, as previously proposed [27,28]. In this case, there would have been a strong selection for RNAs to have compositions that would have resulted in the binding of the maximum proportion of the amino acids present in the prebiotic ecology. Hence, if proteins had been synthesised endlessly they would have remained dynamically attached to the selected RNAs and have protected it.

### 5.2. The AL-Pentamer Proximity as a Marker of Age of the Genome

**Figure 9:**
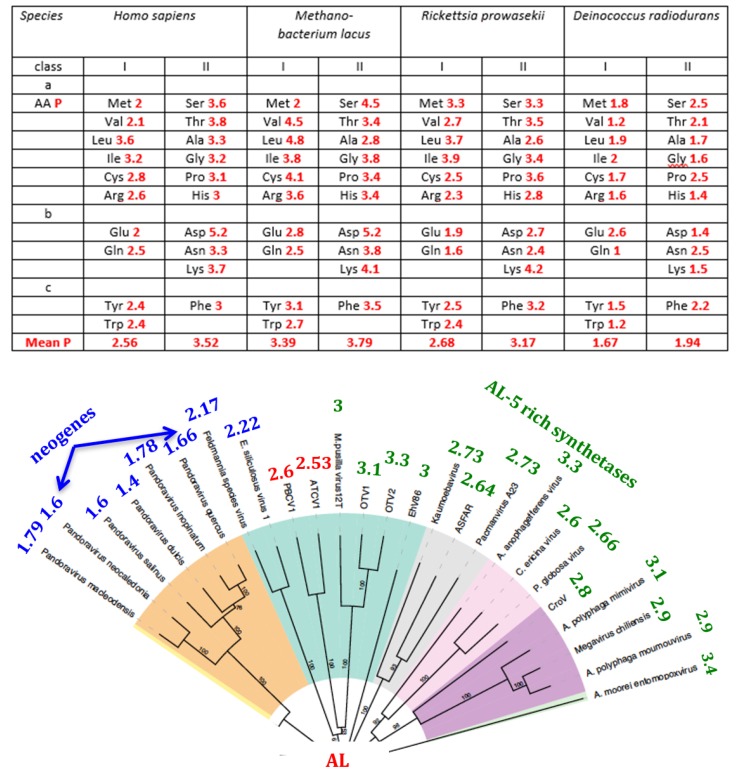
Top: AL-proximity (calculated for the 22 AL-pentamers) of amino-acyl-tRNA synthetases indicated for their classes I and II. Bottom: Giant viruses classification tree. The numbers at the periphery of the circular tree (adapted from [111]) indicate the AL-proximity (calculated for the 9 more frequent AL-pentamers of Table 3) of the Giant viruses’ genomes.

Class II of aminoacyl-tRNA synthetases constitutes a set of very ancient multi domain proteins [25,93]. By calculating their AL-proximity, we see that their genes are closer to AL than the genes of the class I (Figure 9 Top). This is available for the 20 synthetases in human, an archaeum (*Methanobacterium lacus*), a proteobacterium (*Rickettsia prowazekii*) close to mitochondria, and an extremophilic bacterium (*Deinococcus radiodurans*, see Supplementary Material 6).

**Figure 10:**
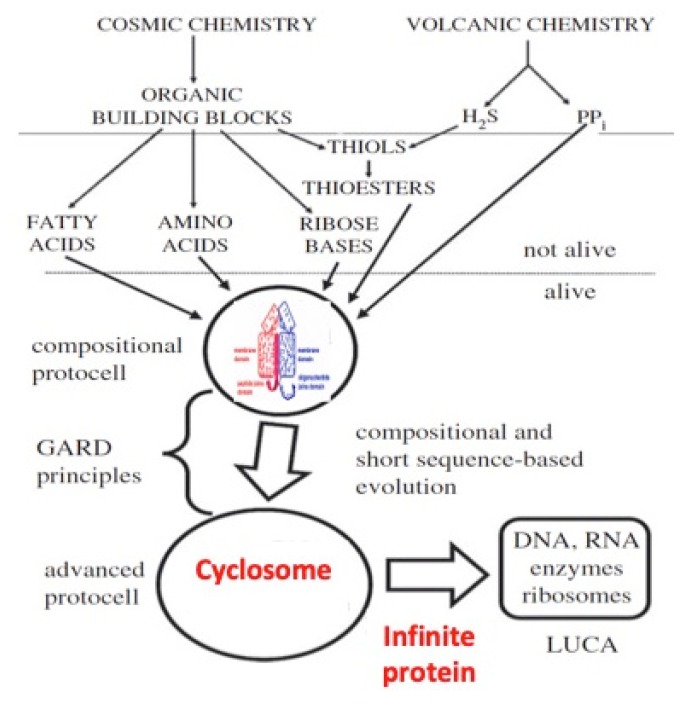
The primitive life machinery (adapted from [92]).

The lowest proximity is observed for *Deinococcus radiodurans*, which is capable of genetic transformation by homologous recombination. We observe the same phenomenon for the *Pandora* viruses (Figure 10 Bottom), which are able to create neogenes and which are considered as recently evolved additions to the large family of giant viruses [111]. These observations as well as the order observed between mean AL-proximities of SASPs (4.49), 5S rRNAs (4.01) and cytidine deaminases (3.36), which respectively protect DNA backbone (SASP), act as mediator between tRNA and ribosome (5S rRNA), and control the cell pyrimidine level (cytidine deaminase) suggest AL-proximity as a marker of genome age, which could constitute a further topic of study.

### 5.3. ‘Infinite’ Proteins

The existence of circular mRNAs makes it possible for ribosomes to translate them without ever encountering a translational stop. This could lead to the synthesis of essentially ‘infinite’ proteins. We propose that the synthesis of such proteins could have occurred at an early stage of the origins of life scenario if the AL cyclosome were simultaneously mRNA/tRNA/synthetase/rRNA. In support of this, the oldest synthetase genes (type II) of *Rickettsia prowazekii* are close to AL (Table 4 and Figure 9 Top), which supports the idea that AL functioned as a primitive protein-synthesizing machine acting without the whole ribosomal machinery for catalysing the first peptides (Figure 10).

A reversible, stereo-binding between AL and amino acids from a Miller-like source could have catalysed peptide bonds to synthesize a protein with a sequence that would have only been partly random since some juxtaposition, alignment, and orientation on the cyclosome would have occurred [110]; the UGA inside the AL would not necessarily have perturbed the machine because neither reading frames nor punctuation codons would have been needed to produce an “infinite” protein in this way. At a later stage of the evolution of the translational machinery, we propose that synthesis of such proteins would have been associated with (1) a relatively weak primitive Shine-Dalgarno RBS sequence GGAGGU which has a weak complementary sequence inside the AL, CUGCCA, and which would have had the advantage of limiting steric problems due to too many ribosomes trying to bind; (2) a relatively long mRNA; (3) a limited codon repertoire; and (4) the tendency of these proteins to form filaments.

### 5.4. tRNA Building

**Figure 11:**
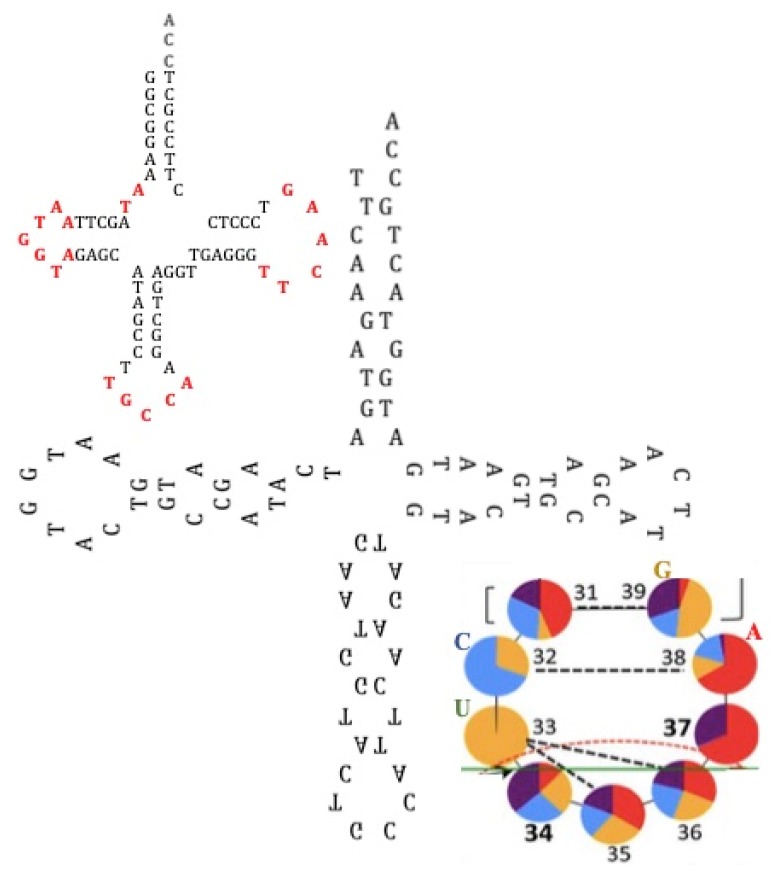
(**a**) The GlytRNA^GCC^ of *Lupine* [66]; (**b**) reconstruction of the loops of the GlytRNA^GCC^ of *Lupine* from four partial hairpins built from AL ring; (**c**) frequency histograms of bases in the anticodon loop of tRNAs belonging to the three domains of life [114].

A way to build a tRNA molecule from four AL hairpins could consist in following as suggested by many studies [112,113,114], which propose that the contemporary tRNA was formed by the ligation of four half-sized hairpin-like RNAs. In Figure 11, four partial hairpins from AL ring have been used for reconstructing the loops of the GlytRNA^GCC^ of *Lupine* [66], this structure having been able to evolve towards the current structure by replacement of unmatched amino acid pairs.

## 6. Conclusions

To conclude, a small circular RNA, called AL, has been proposed with a sequence that has the following features:

- Its subsequences (namely, pentamers) are observed as relics in many parts of modern genomes, especially in Archaea;
- AL relics are often present in ^t^RNA loops, and in mitochondrial D-loops;
- An AL-heptamer constitutes the major part of the exon/intron boundary;
- A scalar proximity to AL explains the relationships between polymerases and, more generally, between complete genomes in phylogenetic trees of Archaea. This proximity suggests a common origin for these genomes.

Hence, the AL cyclosome could have played the role of an ancient protein-synthesizing machine. This claim is central to the stereochemical hypothesis of the genetic code [115] and to the proposal by A. Katchalsky in 1973 [116]: The existence of catalytic RNAs in clays such as the “montmorillonite” may have facilitated the synthesis of small peptides and long RNAs (as is now done by synthetases, polymerases and replicases), thereby constituting an autocatalytic loop at the origin of life.

Hairpin palindromic structure Hairpin size

The existence of a simple RNA structure capable of surviving as a stable hairpin or functioning in a ring form was postulated soon after Katchalsky’s hypothesis [46,47,112], and numerous experimental works [117,118,119] now reinforce this stereochemical hypothesis in a field that continues to advance both experimentally and theoretically.

**Figure 12:**
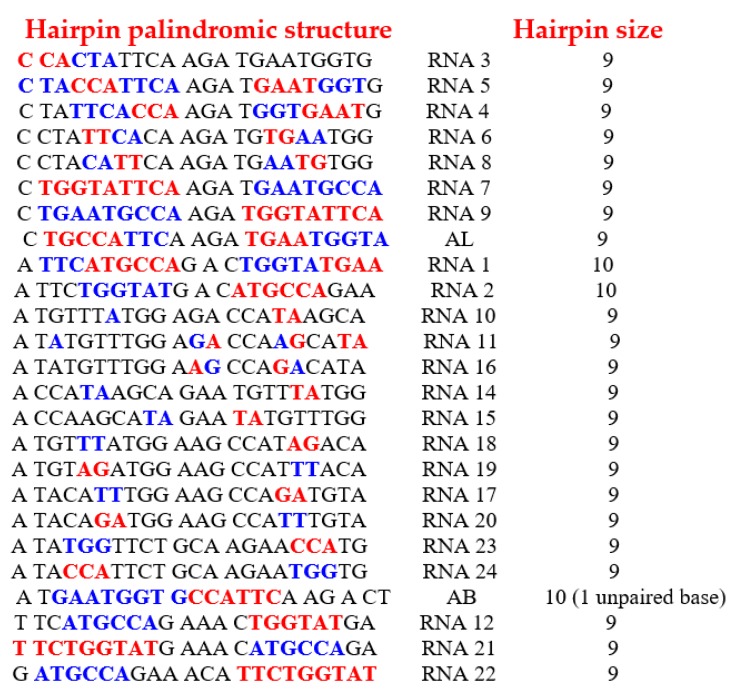
The palindromic structure of the 25 rings. The couples or triplets of rings are obtained by exchanging the blue and the red subsequences.

We anticipate six research developments will follow from the hypothesis presented here:

- An attempt to take into account the potential evolutional path from the AL ring to the large ribosomal subunits (LSU) extracted from the modular organization of the rRNAs structure [17,94,120];
- A search for more AL relics in modern genomes at critical functional steps of the nuclear transcription/translation processes (notably when they are coupled as in Archaea [121], in which the Archaea tRNA^Gly^ presents the following sequence in its three successive loops: TGGTA CTGCCA TTCAA, that is a 16-mer from AL [122]), mitochondrial energetic or cellular immune receptor machineries);
- An attempt to explain the evolution of tRNA secondary structures in relation to the genetic code [123,124,125,126,127,128,129,130];
- An attempt to understand the evolution of immune systems (from CRISPR and TOLL to RAG systems [94,95,96,97]), taking into account the reuse of former AL RNA fragments already present in the “cyclosome”;
- The discovery of sequences linked to AL useful for synthetic biology and studies on “minimal cell” and its primitive genome, with original stable structures as those observed in the “cyclosome” (Figure 12);
- The identification of genetic networks based on common sequences inherited from AL and appearing in regulatory RNAs like microRNAs or circular RNAs.

## Figures and Tables

**Table 1:** Common subsequences among genomes of different species.

**Rhodobacter sphaeroides**
**AATGGTA**T**T**C**CCATTC**G**A**TT**TG tRNA-Gly (http://lowelab.ucsc.edu/GtRNAdb/Rhod_spha_ATCC_17029/rhodSpha_ATCC17029-tRNAs.fa)**
AATGGTACTGCGTC**TCAAGA**C**G 5S rRNA (http://www.combio.pl/rrna/alignment/)**
CC**TGG**A**ACTGCCATT**G**AA**AC**T**C **16S rRNA (https://www.ncbi.nlm.nih.gov/nuccore/636559472?report=fasta)**
**AATGGTACTGCCATTCAAGATG Consensus**
**Rhodospirillum rubrum**
TGAATGGTACTTCCAATTCGAA tRNA-Gly (http://trna.ie.niigata-u.ac.jp/)
CC**AATGGTACTGC**GTC**T**T**AAG**G **5S rRNA (http://www.combio.pl/rrna/alignment/)**
CTCCAGGTACTGCCCTTGATAC 16S rRNA (https://www.arb-silva.de/browser/)
CGAATGGTACTGCCATTTAAAA Consensus
**Rubellimicrobium thermophilum**
AGTGGTACTTCCATTCGACATG tRNA-Gly (http://trna.ie.niigata-u.ac.jp/)
AATGGTACTGCGCCTCAAGACG 5S rRNA (http://www.combio.pl/rrna/alignment/)
GATGGTCCAGGCGCTGCCGCTC 16S rRNA (https://www.arb-silva.de/browser/)
AATGGTACTGCCACTCAAGATG Consensus
**Haematobacter missouriensis**
AGGGGTATTGCCATTCGAATTA tRNA-Gly (http://trna.ie.niigata-u.ac.jp/cgi-bin/trnadb/whole_detail.cgi?SID=2138813)
TATGGTGCTTCCATTCCCGCTA tRNA-Gly (https://www.ncbi.nlm.nih.gov/nuccore/672903602?report=genbank)
**AATGGTACTGC**GTC**TCAAGA**CG **5S rRNA (http://www.combio.pl/rrna/alignment/)**
AATGGTAGTGACAATGGGTTAA 16S rRNA (https://www.arb-silva.de/browser/)
**AATGGTACTGCCATTCAAGATG Consensus**
**Paracoccus sp. S4493**
AATGGTACTTCCCTTCGATTTA tRNA-Gly (https://www.ncbi.nlm.nih.gov/nuccore/NZ_JXYF01000001.1?from=63131&to=63204&sat=19&sat_key=63645080&report=fasta&strand=2)
G**ATGGTACTGC**GTC**T**T**AAGA**CG **5S rRNA **(**http://www.combio.pl/rrna/alignment/)**
AATGGTGGTGACAGTGGGTTAA 16S rRNA (http://www.ebi.ac.uk/ena/data/view/FJ457300&display=fasta)
AATGGTACTGCCATTCAATTTA Consensus
**Flavobacteria bacterium MS024-2A**
CTGGTATTGCCATTCGAATCGC tRNA-Gly (http://gtrnadb.ucsc.edu/genomes/bacteria/Flav_bact_3519_10/flavBact_3519_10-tRNAs.fa)
ATGGTACTGCCATCCGGTGGGA 5S rRNA (http://www.combio.pl/rrna/alignment/)
ATGGTAACGGCATACCAAGGCA 16S RRNA (http://www.ebi.ac.uk/ena/data/view/AM931128&display=fasta)
ATGGTACTGCCATTCGAAGGGA Consensus
**Methanococcus maripaludis**
CTGGTACTTCCATTCAAATCGT tRNA-Gly (http://gtrnadb.ucsc.edu/genomes/archaea/Meth_mari_C5/methMari_C5_1-tRNAs.fa)
TAAGTACTGCCATCUGGUGGGA 5S rRNA (http://biobases.ibch.poznan.pl/htbins/getseq.cgi?name= Methanococcus%20maripaludis)
TCGGTACGGGCCTTGAGAGAGG 16S rRNA (http://www.ebi.ac.uk/ena/data/view/AB546258&display=fasta)
TTGGTACTGCCATTCAGAGAGA Consensus
**Tremella mesenterica**
GATCTGCGAAGTCAAGATGAAT 5S rRNA (http://www.combio.pl/rrna/alignment/)
GGTAA**T**T**C**T**A**GAGCTA**AT**AC**AT18S rRNA (https://www.ncbi.nlm.nih.gov/nuccore/256600119?report=fasta)**
GTAC**C**GTGAGGGA**AAGATGAA**A **28S rRNA (https://www.ncbi.nlm.nih.gov/nuccore/46402656?report=fasta)**
GGTC**C**GTGA**A**G**TCAAGATGAAT Consensus**
**Homo sapiens**
GTGGTACTC**CCATTCAA**TT**TG**G** tRNA (http://trna.bioinf.uni-leipzig.de/DataOutput/Result)**
ATGGTAG**T**CG**C**CG**T**GCCT**A**CC**A 18S rRNA (https://www.ncbi.nlm.nih.gov/nuccore/225637497?report=fasta)**
ATGGTAA**T**C**C**TGC**TCA**GTAC**GA 28S rRNA (https://www.ncbi.nlm.nih.gov/nuccore/1154886866?report=fasta)**
ATGGTACTC**CCATTCAA**T**A**C**GA Consensus**
AATGGTACTGCCATTTAAAACG Consensus Bacteria
**AATGGTACTGCCATTCAAGATG Consensus Bacteria**
**AATGGTACTGCCATTCAAGATG Consensus Bacteria**
AATGGTACTGCCACTCAAGATG Consensus Bacteria
**AATGGTACTGCCATTCAAGATG Consensus Bacteria**
AATGGTACTGCCATTCAATTTA Consensus Bacteria
ATTGGTACTGCCATTCAGAGAG Consensus Archaea
AATGGTC**C**GTGA**A**G**TCAAGATG Consensus Eukaryote** AATGGTACTC**CCATTCAA**T**A**C**G Consensus Eukaryote**
**AATGGTACTGCCATTCAAGATG Consensus consensorum**

**Table 2:** Percentages of tRNAs containing TGGTA and TTCNA in their D- and T_Ψ_-loops, among tRNAs having NTGCCAN as the anticodon loop in different species of the tRNADB-CE database.

Species	Percentages
Archaea	248/584 = 42.5%
Bacteria	131983/155823 = 84.7%
Plant	44/80 = 55%
Fungi	106/115 = 92.2%
Virus	6/18 = 33.3%
Phage	67/276 = 24.3%
Chloroplast	109/116 = 94%

**Table 3:** The most frequent pentamers in 3801 human circular RNAs from circBase. The observed numbers can be compared to the number of pentamers obtained at random, 35,379 ± 310* (* for the 90%-confidence interval).

AL-Pentamer	Observed Number
ATTCA	43,219 *
TTCAA	51,917 *
TCAAG	44,233 *
CAAGA	46,523 *
AAGAT	43,189 *
AGATG	48,717 *
GATGA	34,600
ATGAA	51,794 *
TGAAT	44,410 *

**Table 4:** Gly-tRNA synthetases of different microorganisms [69] with (in red) their AL-proximity.

Homo sapiens mRNA for glycyl-tRNA synthetase, complete cds GenBank: D30658.1 36 × 100/2279 = 1.58
Helicobacter pylori B38 complete genome, strain B38 NCBI Reference Sequence: NC_012973.1: c941829-940933 glycyl—tRNA ligase subunit alpha 15x100/893 = 1.68
Methanococcus maripaludis, strain DSM 2067 chromosome, complete genome NCBI Reference Sequence: NZ_CP026606.1:782166-783890 glycyl-tRNA synthetase 32x100/1721 = 1.86
Fusarium oxysporum f. sp. melonis 26406 unplaced genomic scaffold supercont1.3, whole genome shotgun sequence GenBank: JH659331.1: c2101550-2098309 glycyl-tRNA synthetase 68x100/3238 = 2.10
Prochlorococcus marinus str. NATL1A, complete genome GenBank: CP000553.1: 728949-731111 glycyl-tRNA synthetase 50x100/2155 = 2.32
Methanocaldococcus jannaschii DSM 2661, complete genome GenBank: L77117.1: 219104-220837 glycyl-tRNA synthetase 41x100/1730 = 2.37
Archae Methanococcoides methylutens MM1, complete genome GenBank: CP009518.1: 1554853-1556598 glycyl-tRNA synthetase 43x100/1738 = 2.47
Archae Candidatus Nanosalinarum sp. J07AB56 genomic scaffold scf_7180000039101, whole genome shotgun sequence GenBank: GL982569.1 571934-572506 prolyl-tRNA synthetase 32x100/1286 = 2.49
Methanococcus maripaludis C5, complete genome GenBank: CP000609.1: c1395811-1394084 glycyl-tRNA synthetase 43x100/1720 = 2.50
Methanobacterium formicicum strain BRM9, complete genome GenBank: CP006933.1 556622-558343 glycyl-tRNA synthetase 44x100/1718 = 2.56
Rickettsia prowazekii strain Naples-1chromosome, complete genome GenBank: CP014865.1: c1072366-1071497 glycine-tRNA ligase subunit alpha 25x100/866 = 2.89

**Table 5:** The tRNAs of *Thalassiosira pseudonana* (from https://www.ncbi.nlm.nih.gov/nuccore/) with subsequences from AL (left) and AL-proximities (right) in red.

T**CA**A**TCAAGATGAA**GA**GTAC**GT tRNA synthetase CCMP1335 XM_002286706.1 **2.73**
AG**A**G**TCAAGATGAAT**A**GTA**G**T**A glycyl-tRNA synthetase CCMP1335 XM_002286964.1 **2.44**
CCATG**CAAGATGAATG**TGGG**TG **glycyl-tRNA synthetase CCMP1335 XM_002288084.1 **1.92**
**GCATTCAAGATGAATCTTCTTG arginyl-tRNA synthetase CCMP1335 XM_002288460.1** 2.19
T**C**CA**TC**TCATG**GAATGGTACTG** methionyl-tRNA synthetase CCMP1335 XM_002292549.1 **1.91**
CT**A**CCT**A**G**GATGAA**G**GGT**CA**TG** valyl-tRNA synthetase CCMP1335 XM_002295439.1 **2.32**
G**CAT**A**CAAGA**GT**AATGG**AT**CTG **cysteinyl-tRNA synthetase CCMP1335 XM_002286789.1 **2.04**
**CCATTC**G**A**A**ATG**TT**TGGTA**T**TG **tRNA-Gly mitochondrion DQ186202.1 **7.14**
CCATTGGT**G**T**TG**T**ATGGTA**AAC 60S ribosomal protein CCMP1335 XM_002290416.1 **1.83**
CCA**AGG**A**G**GATG**CGC**G**AG**ACTG 60S ribosomal protein CCMP1335 XM_002290087.1 1.77
C**T**A**G**TCAAGATG**CC**T**C**GT**CTA**G 40S ribosomal protein CCMP1335 XM_002290013.1 2.78
AA**ATT**G**AAGAT**T**A**G**TGGT**GGA**G **40S ribosomal protein CCMP1335 XM_002293773.1 **2.97**
CCAT**GA**A**T**G**T**T**TC**ATG**CCT**CTG 18S ribosomal protein Bc6EHU KP201658.1 1.55
A**C**G**TTCAA**CCAC**A**C**TGG**A**ACTG **16S ribosomal protein BFB575 KC545746.1 **1.51**
**CCATTCAAGATGAATGGTACTG CONSENSUS**

**Table 6:** Sequences of elements involved in defence mechanisms compared to AL.

3′-**ATACATCCC(C)TCTTAAGTTCCCTT**-5′ (TIR)
3′-**TTCCATCCC -TCTTAAGTTCGATT**-5′ (CRISPR)
5′-**ATGGTACTG -- CCATTCAAGATGA**-3′ (AL)
5′-**GTGATACAG -- CCCTTAACAAAAA**-3′ (murine consensus RSS)
5′-**ATTCAACATGAA**-3′ (human RSS spacer)

## Supplementary Materials

The following are available online at https://www.mdpi.com/2075-1729/9/2/51/s1.
