# Supplementary material for: Emergence of a “Cyclosome” in a Primitive Network Capable of Building “Infinite” Proteins
Source: Life (Basel). 2019 Jun 18;9(2):51. doi: 10.3390/life9020051 (PMC6617141; doi:10.3390/life9020051)
Supplement: Supplementary file 1 [file life-09-00051-s001.pdf]

Coding sequences of genes cytidine deaminase

>A2_HOMO
ATGGCCCAGAAGGAAGAGGCTGCTGTGGCCACTGAGG**CTGCC**TCCCA**GAATGG**GGAGGATCTGGAGAACCTGGA
CGACCCTGAGAAGCTGAAAGAGCTGATTGAG**CTGCC**GCCCTTTGAGATTGTCACAGGAGAACGG**CTGCCTGCCA**
ACTTCTTTAAATTCCAGTTCCG**GAATG**TGGAGTACAGTTCCGGGAGGAA**CAAGA**CCTTCCTCTGCTATGTGGTT
GAAGCACAGGGCAAGGGGGGCCAAGTGCAGGCATCTCGGGGATACCTAGAG**GATGA**GCATGCGG**CTGCC**CATGC
AGAGGAAGCTTTC**TTCAA**CACCATC**CTGCCA**GCCTTCGACCCAGCCCTGC**GGTAC**AATGTCACC**TGGTA**TGTGT
CCTCCAGCCCCTGTGCAGCGTGTGCTGACCGCATTATCAAAACCCTTAG**CAAGA**C**CAAGA**ACCTGCGTCTGCT**C**
ATTCTGGTGGGTCGACTCTTCATGTGGGAGGAGCCGGAGATCCAGGCTGCTCTGAAGAAGCTGAAGGAGGCTGG
CTGTAA**ACTGC**GCATC**ATGAA**GCCCCAGGACTTCGAATATGTCTGGCAGAATTTTGTGGAG**CAAGA**AGAGGG**TG**
AATCCAAGGCCTTTCAGCCCTGGGAGGA**CATTCA**GGAGAACTTCCTATACTACGAGGAGAAGTTGGCAGACATC
CTGAAG

24 668 **3.6**

>A1_HOMO
ATGACTCCAGAGGAGGAAGTCCAGAGACAGAGCACCATGACTTCTGAGAAAGGTCC**TTCAA**CCGGTGACCCCAC
TCTGAGGAGAAGAATCGAACCCTGGGAGTTTGACGTCTTCTATGACCCCAGAGAACTTCGTAAAGAGGCCTGTC
TGCTCTACGAAA**TCAAG**TGGGGCATGAGCCGG**AAGAT**CTGGCGAAGCTCAGGCAAAAACACCACCAATCACGTG
GAAGTTAATTTTATAAAAAAATTTACGTCAGAAAGAGATTTTCACCCATCCATGAGCTGCTCCATCACCTGGTT
CTTGTCCTGGAGTCCCTGCTGG**GAATG**CTCCCAGGCTATTAGAGAGTTTCTGAGTCGGCACCCTGGTGTGACTC
TAGTGATCTACGTAGCTCGGCTTTTTTGGCACATGGATCAACAAAATCGGCAAGGTCTCAGGGACCTTGTTAAC
AGTGGAGTAACT**ATTCA**GATTATGAGAGCATCAGAGTATTATC**ACTGC**TGGAGGAATTTTGTCAACTACCCACC
TGGG**GATGAA**GCTCACTGGCCACAATACCCACCTCTGTG**GATGA**TGTTGTACGCACTGGAGCTGC**ACTGC**ATAA
TTCTAAGTCTTCCACCCTGTTTA**AAGATTTCAAGAAGATG**GCAAAATCATCTTACATTTTTCAGACTTCATC**TT**
CAAA**ACTGCCATT**ACCAAACGATTCCGCCACACATCCTTTTAGCTACAGGGCTGATACATCCTTCTGTGGCTTG
GAGA

21 740 **2.8**

>AID_HOMO
ATGGACAGCCTCTT**GATGAA**CCGGAGGAAGTTTCTTTACCA**ATTCAA**AAATGTCCGCTGGGCTAAGGGTCGGCG
TGAGACCTACCTGTGCTACGTAGTGAAGAGGCGTGACAGTGCTACATCCTTTTCACTGGACTTTGGTTATCTTC
GCAATAAGAACGG**CTGCCA**CGTGGAATTGCTCTTCCTCCGCTACATCTCGGACTGGGACCTAGACCCTGGCCGC
TGCTACCGCGTCACCTGGTTCACCTCCTGGAGCCCCTGCTACGACTGTGCCCGACATGTGGCCGACTTTCTGCG
AGGGAACCCCAACCTCAGTCTGAGGATCTTCACCGCGCGCCTCTACTTCTGTGAGGACCGCAAGGCTGAGCCCG
AGGGGCTGCGGCGGCTGCACCGCGCCGGGGTGCAAATA**GCCAT**CATGACC**TTCAAAGAT**TATTTT**TACTGC**TGG
AATACTTTTGTAGAAAACC**ATGAA**AGAACT**TTCAA**AGCCTGGGAAGGGCTGC**ATGAA**A**ATTCA**GTTCGTCTCTC
CAGACAGCTTCGGCGCATCCTTTTGCCCCTGTATGAGGTT**GATGA**CTTACGAGACGCATTTC**GTACT**TTGGGAC
TT

17 590 **2.9**

>A3A_HOMO
ATGGAAGCCAGCCCAGCATCCGGGCCCAGACACTTGATGGATCCACACAT**ATTCA**CTTCCAACTTTAAC**AATGG**
CATTGGAAGGCATAAGACCTACCTGTGCTACGAAGTGGAGCGCCTGGAC**AATGG**CACCTCGG**TCAAGATG**GACC
AGCACAGGGGCTTTCTACACAACCAGGCTAAGAATCTTCTCTGTGGCTTTTACGGCC**GCCAT**GCGGAGCTGCGC
TTCTTGGACCTGGTTCCTTCTTTGCAGTTGGACCCGGCCCAGATCTACAGGGTCACTTGGTTCATCTCCTGGAG
CCCCTGCTTCTCCTGGGGCTGTGCCGGGGAAGTGCGTGCGTTCCTTCAGGAGAACACACACGTGAG**ACTGC**GCA
TCTTCG**CTGCC**CGCATCTATGATTACGACCCCCTATATAAGGAGGCGCTGCAAATGCTGCGGGATGCTGGGGCC
CAAGTCTCCATCATGACCTAC**GATGAAT**TTAAGC**ACTGC**TGGGACACCTTTGTGGACCACCAGGGATGTCCCTT
CCAGCCCTGGGATGGACT**AGATGA**GCACAGCCAAGCCCTGAGTGGGAGGCTGCGG**GCCATTC**TCCAGAATCAGG
GAAAC

19 593 **3.2**

>A3B_N_HOMO
ATGAATCCACAGATCAGAAATCCGATGGAGCGGATGTATCGAGACA**CATTC**TACGACAACTTTGAAAACGAACC
CATCCTCT**ATGGT**CGGAGCTACACTTGGCTGTGCT**ATGAA**GTGAAAATAAAGAGGGGCCGCTCAAATCTCCTTT
GGGACACAGGGGTCTTTCGAGGCCAGGTGTAT**TTCAAG**CCTCAGTACCACGCAGAAATGTGCTTCCTCTCTTGG
TTCTGTGGCAACCAG**CTGCC**TGCTTACAAGTGTTTCCAGATCACCTGGTTTGTATCCTGGACCCC**CTGCC**CGGA
CTGTGTGGCGAAGCTGGCCGAATTCCTGTCTGAGCACCCCAATGTCACCCTGACCATCT**CTGCC**GCCCGCCTCT
ACTAC**TACTG**GGAAAGAGATTACCGAAGGGCGCTCTGCAGGCTGAGTCAGGCAGGAGCCCGCGTGACGATCATG
GACT**ATGAA**GAATTTGCA**TACTGC**TGGGAAAACTTTGTGTACA**ATGAA**GGTCAGCA**ATTCA**TGCCT**TGGTAC**AA
ATTC**GATGAA**AATTATG**CATTC**CTGCACCGCACGCTAAAGGAGATTCTCAGA

21 566 **3.7**

>A3B_C_HOMO
TACCTGATGGATCCAGACA**CATTCA**CT**TTCAA**CTTTAATAATGACCCTTTGGTCCTTCGACGGCGCCAGACCTA
CTTGTGCTATGAGGTGGAGCGCCTGGAC**AATGG**CACCTGGGTCCTGATGGACCAGCACATGGGCTTTCTATGCA
ACGAGGCTAAGAATCTTCTCTGTGGCTTTTACGGCC**GCCAT**GCGGAGCTGCGCTTCTTGGACCTGGTTCCTTCT
TTGCAGTTGGACCCGGCCCAGATCTACAGGGTCACTTGGTTCATCTCCTGGAGCCCCTGCTTCTCCTGGGGCTG
TGCCGGGGAAGTGCGTGCGTTCCTTCAGGAGAACACACACGTGAG**ACTGC**GCATCTTCG**CTGCC**CGCATCTATG
ATTACGACCCCCTATATAAGGAGGCGCTGCAAATGCTGCGGGATGCTGGGGCCCAAGTCTCCATCATGACCTAC
GATGAGTTTGA**GTACTGC**TGGGACACCTTTGTGTACCGCCAGGGATGTCCCTTCCAGCCCTGGGATGGACTAGA
GGAGCACAGCCAAGCCCTGAGTGGGAGGCTGCGG**GCCATTC**TCCAGAATCAGGGAAAC

14 572 **2.5**

>A3C_HOMO
ATGAATCCACAGATCAGAAACCC**GATGAA**GGCAATGTATCCAGGCA**CATTC**TACTTCCAATTTAAAAACCTATG
GGAAGCCAACGATCGGGACGAAACTTGGCTGTGCTTCACCGTGGAAGGTATAAAGCGCCGCTCAGTTGTCTCCT
GGAAGACGGGCGTCTTCCGAAACCAGGTGGATTCTGAGAC**CCATT**GTCATGCAGAAAGGTGCTTCCTCTCTTGG
TTCTGCGACGACA**TACTG**TCTCCTAACACAAAGTACCAGGTCACC**TGGTAC**ACATCTTGGAGCCCTTGCCCAGA
CTGTGCAGGGGAGGTGGCCGAGTTCCTGGCCAGGCACAGCAACG**TGAAT**CTCACCATCTTCACCGCCCGCCTCT
ACTACTTCCAGTATCCATGTTACCAGGAGGGGCTCCGCAGCCTGAGTCAGGAAGGGGTCGCTGTGGAGATCATG
GACT**ATGAAGAT**TTTAAATATTGTTGGGAAAACTTTGTGTACAATGATAATGA**GCCATTCAAG**CCTTGGAAGGG
ATTAAAAACCAACTTTCGACTTCTGAAAAGAAGGCTACGGGAGAGTCTCCAG

18 566 **3.2**

>A3D_N_HOMO
ATGAATCCACAGATCAGAAATCCGATGGAGCGGATGTATCGAGACA**CATTC**TACGACAACTTTGAAAACGAACC
CATCCTCT**ATGGT**CGGAGCTACACTTGGCTGTGCT**ATGAA**GTGAAAATAAAGAGGGGCCGCTCAAATCTCCTTT
GGGACACAGGGGTCTTTCGAGGCCC**GGTACT**ACCCAAACGTCAGTCGAATCACAGGCAGGAGGTGTATTTCCGG
TTTGAGAACCACGCAGAAATGTGCTTCTTATCTTGGTTCTGTGGCAACCG**ACTGCC**TGCTAACAGGCGCTTCCA
GATCACCTGGTTTGTATCATGGAACCC**CTGCC**TGCCCTGTGTGGTGAAGGTGACCAAATTCTTGGCTGAGCACC
CCAATGTCACCCTGACCATCT**CTGCC**GCCCGCCTCTACTACTACCGGGATAGAGATTGGCGGTGGGTGCTCCTC
AGGCTGCATAAGGCAGGGGCCCGTGTG**AAGAT**CATGGACT**ATGAA**GACTTTGCA**TACTGC**TGGGAAAACTTTGT
GTGCA**ATGAA**GGTCA**GCCATTCA**TGCCT**TGGTAC**AAATTC**GATGA**CAATTATGCATCCCTGCACCGCACGCTAA
AGGAGATTCTCAGA

23 602 **3.8**

>A3D_C_HOMO
AACCCGATGGAGGCAATGTACCCACACATATTCTACTTCCACTTTAAAAACC**TACTG**AAAGCCTGTGGTCGGAA
CGAAAGCTGGCTGTGCTTCACCATGGAAGTTACAAAGCACCACTCAGCTGTCTTCCGGAAGAGGGGCGTCTTCC
GAAACCAGGTGGATCCTGAGAC**CCATT**GTCATGCAGAAAGGTGCTTCCTCTCTTGGTTCTGTGACGACA**TACTG**
TCTCCTAACACAAACTACGAGGTCACC**TGGTAC**ACATCTTGGAGCCCTTGCCCAGAGTGTGCAGGGGAGGTGGC
CGAGTTCCTGGCCAGGCACAGCAACG**TGAAT**CTCACCATCTTCACCGCCCGCCTCTGCTACTTCTGGGATACAG
ATTACCAGGAGGGGCTCTGCAGCCTGAGTCAGGAAGGGGCCTCCGTG**AAGAT**CATGGGCTACA**AAGAT**TTTGTA

TCTTGTTGGAAAAACTTTGTGTACAGTGATGATGAGCCATTCAAGCCTTGGAAGGGACTACAAACCAACTTTCG
ACTTCTGAAAAGAAGGCTACGGGAGATTCTCCAG

16 548 **2.9**

>A3F_N_HOMO
ATGAAGCCTCACTTCAGAAACACAGTGGAGCGAATGTATCGAGACACATTCTCCTACAACTTTTATAATAGACC
CATCCTTTCTCGTCGGAATACCGTCTGGCTGTGCTACGAAGTGAAAACAAAGGGTCCCTCAAGGCCCCGTTTGG
ACGCAAAGATCTTTCGAGGCCAGGTGTATTCCCAGCCTGAGCACCACGCAGAAATGTGCTTCCTCTCTTGGTTC
TGTGGCAACCAGCTGCCTGCTTACAAGTGTTTCCAGATCACCTGGTTTGTATCCTGGACCCCCTGCCCGGACTG
TGTGGCGAAGCTGGCCGAATTCCTGTCTGAGCACCCCAATGTCACCCTGACCATCTCCGCCGCCCGCCTCTACT
ACTACTGGGAAAGAGATTACCGAAGGGCGCTCTGCAGGCTGAGTCAGGCAGGGGCCCGTGTGAAGATTATGGAC
GATGAAGAATTTGCATACTGCTGGGAAAACTTTGTGTACAGTGAAGGTCAGCCATTCATGCCTTGGTACAAATT
CGATGACAATTATGCATTCCTGCACCGCACGCTAAAGGAGATTCTCAGA

20 563 **3.6**

>A3F_C_HOMO
AACCCGATGGAGGCAATGTATCCACACATATTCTACTTCCACTTTAAAAACCTACGCAAAGCCTATGGTCGGAA
CGAAAGCTGGCTGTGCTTCACCATGGAAGTTGTAAAGCACCACTCACCTGTCTCCTGGAAGAGGGGCGTCTTCC
GAAACCAGGTGGATCCTGAGACCCATTGTCATGCAGAAAGGTGCTTCCTCTCTTGGTTCTGTGACGACATACTG
TCTCCTAACACAAACTACGAGGTCACCTGGTACACATCTTGGAGCCCTTGCCCAGAGTGTGCAGGGGAGGTGGC
CGAGTTCCTGGCCAGGCACAGCAACGTGAATCTCACCATCTTCACCGCCCGCCTCTACTACTTCTGGGATACAG
ATTACCAGGAGGGGCTCCGCAGCCTGAGTCAGGAAGGGGCCTCCGTGGAGATCATGGGCTACAAAGATTTTAAA
TATTGTTGGGAAAACTTTGTGTACAATGATGATGAGCCATTCAAGCCTTGGAAAGGACTAAAATACAACTTTCT
ATTCCTGGACAGCAAGCTGCAGGAGATTCTCGAG

14 548 **2.6**

>A3G_N_HOMO
ATGAAGCCTCACTTCAGAAACACAGTGGAGCGAATGTATCGAGACACATTCTCCTACAACTTTTATAATAGACC
CATCCTTTCTCGTCGGAATACCGTCTGGCTGTGCTACGAAGTGAAAACAAAGGGTCCCTCAAGGCCCCCTTTGG
ACGCAAAGATCTTTCGAGGCCAGGTGTATTCCGAACTTAAGTACCACCCAGAGATGAGATTCTTCCACTGGTTC
AGCAAGTGGAGGAAGCTGCATCGTGACCAGGAGTATGAGGTCACCTGGTACATATCCTGGAGCCCCTGCACAAA
GTGTACAAGGGATATGGCCACGTTCCTGGCCGAGGACCCGAAGGTTACCCTGACCATCTTCGTTGCCCGCCTCT
ACTACTTCTGGGACCCAGATTACCAGGAGGCGCTTCGCAGCCTGTGTCAGAAAAGAGACGGTCCGCGTGCCACC
ATGAAGATCATGAATTATGACGAATTTCAGCACTGTTGGAGCAAGTTCGTGTACAGCCAAAGAGAGCTATTTGA
GCCTTGGAATAATCTGCCTAAATATTATATATTACTGCACATCATGCTGGGGGAGATTCTCAGA

17 578 **2.9**

>A3G_C_HOMO
CACTCGATGGATCCACCCACATTCACTTTCAACTTTAACAATGAACCTTGGGTCAGAGGACGGCATGAGACTTA
CCTGTGTTATGAGGTGGAGCGCATGCACAATGACACCTGGGTCCTGCTGAACCAGCGCAGGGGCTTTCTATGCA
ACCAGGCTCCACATAAACACGGTTTCCTTGAAGGCCGCCATGCAGAGCTGTGCTTCCTGGACGTGATTCCCTTT
TGGAAGCTGGACCTGGACCAGGACTACAGGGTTACCTGCTTCACCTCCTGGAGCCCCTGCTTCAGCTGTGCCCA
GGAAATGGCTAAATTCATTTCAAAAAACAAACACGTGAGCCTGTGCATCTTCACTGCCCGCATCTATGATGATC
AAGGAAGATGTCAGGAGGGGCTGCGCACCCTGGCCGAGGCTGGGGCCAAAATTTCAATAATGACATACAGTGAA
TTTAAGCACTGCTGGGACACCTTTGTGGACCACCAGGGATGTCCCTTCCAGCCCTGGATGGACTAGATGAGCA
CAGCCAAGACCTGAGTGGGAGGCTGCGGGCCATTCTCCAGAATCAGGAAAAC

23 566 **4.1**

>A3H_HOMO
ATGGCTCTGTTAACAGCCGAAACATTCCGCTTACAGTTTAACAACAAGCGCCGCCTCAGAAGGCCTTACTACCC
GAGGAAGGCCCTCTTGTGTTACCAGCTGACGCCGCAGAATGGCTCCACGCCCACGAGAGGCTACTTTGAAAACA
AGAAAAGTGCCATGCAGAAATTTGCTTTATTAACGAGATCAAGTCCATGGGACTGGACGAAACGCAGTGCTAC

CAAGTCACCTGTTACCTCACGTGGAGCCCCTGCTCCTCCTGTGCCTGGGAGCTGGTTGACTTCA**TCAAG**GCTCA
CGACCATCTGAACCTGGGCATCTTCGCCTCCCGCCT**GTACT**ACCACTGGTGCAAGCCCCAGCAGAAGGGGCTGC
GGCTTCTGTGTGGATCCCAGGTCCCGGTGGAGGTCATGGGCTTCCCAGAGTTTGCTG**ACTGC**TGGGAAAACTTT
GTGGACCACGAGAAACCGCTTTCC**TTCAA**CCCCTAT**AAGATG**TTAGAGGAGCTAGATAAAAACAGTCGA**GCCAT**
AAAGCGACGGCTTGAGAGGATAAAGCAGTCC

14 545 **2.6**

>A3-PSEUDO_HOMO
ATGAAGCCTCAGATCAGAAACCTGATGGAGTGGATGTATCCAGGCA**CATTC**TACTACAACTTTGAAAACAGACC
CATCCTCTCAGGTTGGAACACCACCTGGCTGTGCTACAAA**ATGAA**AACAAAGAAGGACCCCTCAAAGCCCCCTT
TGGACGCAAGGATCTTTGGAGGCCAGGTCTATTCCAAGCCTGAACACCACCCAG**AGATGA**GATTCGTAGATTGG
TTCTGCAACTCGAGGCTGCATCGTGACCAGGACTACCTGGTCATC**TGGTAC**ATCTCCTGGAGTCCCTGCTCAGA
GTATGCAGGGAACGTGGCAGAGTTCCTGGCCAAGGATGGCAAGGTCACCCTGACCATCTTCGTTGCCCACCTCT
ACTACTTCTGGGAAGCAGATTAC**CAAGA**GGAGCTTCAC**AGATGGT**GTCAGAAAAAACAGCCACA**TGCCA**GC**ATG**
AAGATC**ATGAA**CTAT**GATGAATTTCAA**CACTGTTGGGACAAATTTGTGTACAACCAAA**GAATG**CTATTTGATCC
TTGGAAGCAAC**TGAAT**ACAAATTATGCAT**TACTGC**ACAGCATGCTGGGGGAGATTCTCAGACACTTCACTTACA
ATTTTACCAATGATCCTTCAGTCTCTGGGCAGCACCAGACCTACCTGTGCTGCAAGGTGGAG**TGCCA**AAACAAT
GATGCCTGGGTCCCTCTGGACCAGCAACAAGGGCATCCTACCCAGCCAGGCTT**TGAAT**CAGTTGCTATTAAGTT
CCATGCAGAGCAGTGCTTCCTGTACCTGATTTCCTTTTGGAAGCTGGACCGGGCTCAGTGCTACAGGGTCACCT
GGTTCATCTCCTGGAGTCCCTGCTTCAGCTGTGCCCAGCAAGTGGCTACATTTCATTGGGAGAAC**AGATG**TGTG
AGCCTGCACATCTTCGCTGCATGCATCTATAATTATCTCCCAGGATATGAGGGGCTGTGCATGCTGCAGAGGGC
TGGGACCCAAATCTCCATCATGACCCGGAAGTTTAGGC**ACTGC**TGGGTCACCTTTGTGGACCACCAGGGATGTC
CCTTCCAGCCCTGGGATGGACT**AGATGA**GCACAG**TCAAG**CCCTAAGTGGGAGGCTGCAG**GCCATTC**TCCAGAAT
CAGGAAAAC

32 1115 **2.9**

>A2_MUS
ATGGCTCAGAAGGAAGAGGCCGCTGAGGCTGCGGCGCCAGCCTCTCA**GAATGGAGATGA**TTTGGAGAACCTGGA
AGACCCTGAGAAGCTGAAAGAGCTGATCGATCTTCCGCCCTTCGAGATTGTCACTGGGGTGCGGCTACCAGTCA
ACTTC**TTCAAG**TTTCAGTTCCGGAACGTGGAATACAGTTCCGGGCGGAATAAGACCTTTCTCTGCTATGTGGTC
GAAGTACAGAGTAAGGGCGGCCAAGCGCAGGCGACGCAGGGCTACCTGGAG**GATGAA**CACGCAGGTGCCCACGC
CGAGGAGGCTTTCTTTAACACCATC**CTGCCA**GCTTTCGACCCGGCCC**TCAAG**TACAATGTCACC**TGGTA**TGTGT
CCTCCAGCCCCTGTGCAGCCTGCGCTGACCGGATTCTCAAAACCCTCAG**CAAGA**CTAAGAACCTTCGTCTGCT**C**
ATTCTGGTGAGCCGGCTCTTCATGTGGGAGGAGCCAGAGGTCCAGGCCGCTCTGAAAAAGCTGAAGGAGGCCGG
CTGCAAACTTCGCATC**ATGAA**ACCCCAGGACTTCGAGTACATCTGGCAGAATTTTGTGGAG**CAAGA**AGAGGG**TG**
AATCCAAGGCCTTTGAGCCCTGGGAGGA**CATTCA**GGAGAACTTCCTATACTATGAGGAGAAGTTGGCAGACATC
CTGAAG

19 668 **2.8**

>A1_MUS
ATGAGTTCCGAGACAGGCCCTGTAGCTGTTGATCCCACTCTGAGGAGAAGAATTGAGCCCCACGAGTTTGAAGT
CTTCTTTGACCCCCGGGAGCTTCGGAAAGAGACCTGTCTGCTGTATGAGATCAACTGGGGTGGAAGGCACAGTG
TCTGGCGACACACGAGCCAAAACACCAGCAACCACGTTGAAGTCAACTTCTTAGAAAAATTTACTACAGA**AAGA**
TACTTTCGTCCGAACACC**AGATG**CT**CCATT**ACCTGGTTCCTGTCCTGGAGTCCCTGCGGGGAGTGCTCCAGG**GC**
CATTACAGAGTTTCTGAGCCGACACCCCTATGTAACTCTGTTTATTTACATAGCACGGCTTTATCACCACACGG
ATCAGCGAAACCGCCAAGGACTCAGGGACCTTATTAGCAGCGGTGTGACTATCCAGATCATGACAGAG**CAAGA**G
TATTGT**TACTGC**TGGAGGAATTTCGTCAACTACCCCCC**TTCAA**ACGAAGCTTATTGGCCAA**GGTAC**CCCCATCT
GTGGGTGAAACTGTAT**GTACTG**GAGCTC**TACTGC**ATCATTTAGGACTTCCACCCTGTTTAAAAATTTAAGAA
GAAAGCAACCTCAACTCACGTTTTTCACAATTACTC**TTCAA**AC**CTGCCATT**ACCAAAGGATACCACCCCATCTC
CTTTGGGCTACAGGGTTGAAA

19 683 **2.8**

>A3_N_MUS
ATGCAACCCCAGCGCCTGGGGCCCAGAGCTGGGATGGGA**CCATTC**TGTCTGGGATGCA**GCCAT**CGCAAATGCT**A**
TTCACCGATCAGAAACCTGATATC**TCAAGA**AA**CATTCAAG**TTCCACTTTAAGAACCTAGGCTA**TGCCA**AAGGCC
GGA**AAGAT**ACCTTCTTGTGCT**ATGAA**GTGACTAGAAAGG**ACTGCGATTCA**CCCGTCTCCCTTCACCATGGGGTC
TTTAAGAACAAGGACAACATCCACGCTGAAATCTGCTTTTTA**TACTG**GTTCCATGACAAA**GTACTG**AAAGTGCT
GTCTCCGAGAGAAGAG**TTCAAGAT**CACC**TGGTA**TATGTCCTGGAGCCCCTGTTTC**GAATG**TGCAGAGCAGATAG
TAAGGTTCCTGGCTACACACCACAACCTGAGCCTGGACATCTTCAGCTCCCGCCTCTACAACGTACAGGACCCA
GAAACCCAGCAGAATCTTTGCAGGCTGGTTCAGGAAGGAGCCCAGGTGG**CTGCCAT**GGACCTATACGAATTTAA
AAAGTGTTGGAAGAAGTTTGTGGAC**AATGGT**GGCAGGCG**ATTCA**GGCCTTGGAAAAG**ACTGC**TTACAAATTTTA
GATACCAGGATTCTAAGCTTCAGGAGATTCTGAGG

31 623 **5**

>A3_C_MUS
C**GAATGG**ACCCGCTAAGTGAAGAGGAATTTTACTCGCAGTTTTACAACCAACGAG**TCAAG**CATCTCTGCTACTA
CCACCGC**ATGAA**GCCCTATCTATGCTACCAGCTGGAGCAG**TTCAATGG**CCAAGCGCCACTCAAAGG**CTGCC**TGC
TAAGCGAGAAAGGCAAACAGCATGCAGAAATCCTCTTCCTTGAT**AAGAT**TCGGTCCATGGAGCTGAGCCAAGTG
ACAATCACCTGCTACCTCACCTGGAGCCC**CTGCC**CAAACTGTGCCTGGCAACTGGCGG**CATTCAA**AAGGGATCG
TCCAGATCTAATTCTGCATATCTACACCTCCCGCCTGTATTTCCACTGGAAGAGGCCCTTCCAGAAGGGGCTGT
GTTCTCTGTGGCAATCAGGGATCCTGGTGGACGTCATGGACCTCCCACAGTT**TACTGACTGC**TGGACAAACTTT
GTGAACCCGAAAAGGCCGTTTTG**GCCAT**GGAAAGGATTGGAGATAATCAGCAGGCGCACACAAAGGCGGCTCCG
CAGGA**TCAAG**GAGTCCTGGGGTCTG**CAAGAT**TTGG**TGAATG**ACTTTGGAAACCTACAGCTTGGACCCCCGATGT
CT

20 590 **3.4**

>AID_MUS
ATGGACAGCCTTCT**GATGAA**GCAAAAGAAGTTTCTTTA**CCATTTCAA**AAATGTCCGCTGGGCCAAGGGACGGCA
TGAGACCTACCTCTGCTACGTGGTGAAGAGGAGAGATAG**TGCCA**CCTCCTGCTCACTGGACTTCGGCCACCTTC
GCAACAAGTCTGG**CTGCCA**CGTGGAATTGTTGTTCCTACGCTACATCTCAGACTGGGACCTGGACCCGGGCCGG
TGTTACCGCGTCACCTGGTTCACCTCCTGGAGCCCGTGCTATGACTGTGCCCGGCACGTGGCTGAGTTTCTG**AG**
ATGGAACCCTAACCTCAGCCTGAGGATTTTCACCGCGCGCCTCTACTTCTGTGAAGACCGCAAGGCTGAGCCTG
AGGGGCTGCGGAG**ACTGCA**CCGCGCTGGGGTCCAGATCGGGATCATGACC**TTCAA**AGACTATTTT**TACTGC**TGG
AATACATTTGTAGAAAATCGTGAAAGAACT**TTCAA**AGCCTGGGAAGGGCTAC**ATGAA**AATTCTGTCCGGCTAAC
CAGACAACTTCGGCGCATCCTTTTGCCCTTGTACGAAGTC**GATGA**CTTGCG**AGATG**CATTTCGTATGTTGGGAT
TT

17 590 **2.9**

>A2_RATTUS
ATGGCTCAGAAGGAAGAAGCCGCTGAGGCCGCGGCACCAGCCTCTCAGAACGGAGACGATTTGGAGAACCTAGA
AGACCCTGAGAAGCTGAAGGAGCTGATCGAT**CTGCC**GCCCTTCGAGATCGTCACTGGGGTGCG**ACTGCC**TGTCA
ACTTCTTTAAGTTTCAGTTCCGGAACGTGGAATACAGTTCCGGGCGGAA**CAAGA**CCTTTCTCTGCTATGTGGTC
GAAGCACAGAGCAAGGGAGGCCAAGTGCAGGCGACGCAGGGCTACCTGGAG**GATGAA**CACGCTGGTGCCCACGC
TGAGGAGGCTTTCTTTAACACCATC**CTGCCA**GCTTTCGACCCGGCCCTGAAGTATAATGTCACC**TGGTA**TGTGT
CCTCCAGCCCCTGTGCAGCCTGCGCCGACCGAATTC**TCAAGA**CCCTCAG**CAAGA**C**CAAGA**ACCTGCGGCTGCTC
ATCCTGGTGAGCCGGCTCTTCATGTGGGAGGAGCCAGAGGTCCAGGCCGCCCTGAAGAAGCTGAAGGAGGCGGG
CTGCAAACTTCGCATC**ATGAA**ACCCCAGGACTTCGAGTACCTCTGGCAGAATTTTGTGGAG**CAAGA**AGAGGG**TG**
AATCCAAGGCCTTTGAGCCCTGGGAGGA**CATTCA**GGAGAACTTCCTATACTATGAGGAGAAGTTGGCAGACATC
CTGAAG

18 668 **2.7**

>A1_RATTUS

ATGAGTTCCGAGACAGGCCCTGTAGCTGTTGATCCCACTCTGAGGAGAAGAATTGAGCCCCACGAGTTTGAAGT
CTTCTTTGACCCCCGGGAACTTCGGAAAGAGACCTGTCTGCTGTATGAGATCAACTGGGGAGGAAGGCACAGCA

TCTGGCGACACACGAGCCAAAACACCAACAAACACGTTGAAGTCAATTTCATAGAAAAATTTACTACAGA**AAGA**
TACTTTTGTCCAAACACC**AGATG**CT**CCATT**ACCTGGTTCCTGTCCTGGAGTCCCTGTGGGGAGTGCTCCAGGG**GC**
CATTACAGAATTTTTGAGCCGATACCCCCATGTAACTCTGTTTATTTATATAGCACGGCTTTATCACCACGCAG
ATCCTCGAAATCGGCAAGGACTCAGGGACCTTATTAGCAGCGGTGTTACTATCCAGATCATGACGGAG**CAAGA**G
TCTGGC**TACTGC**TGGAGGAATTTTGTCAACTACTCCCCTTC**GAATGAA**GCTCATTGGCCAAGGTACCCCCATCT
GTGGGTGAGGCTGTAC**GTACTG**GAACTC**TACTGC**ATCATTTAGGACTTCCACCCTGTTTAAATATTTTAAGAA
GAAAACAACCTCAACTCACGTTTTTCACGATTGCTC**TTCAA**AG**CTGCCATT**ACCAAAGGCTACCACCCCACATC
CTGTGGGCCACAGGGTTGAAA

20 683 **2.9**

>A3_N_RATTUS
AGAAACCCGCTAAAGAAGTTATATCAACAAA**CATTC**TACTTTCATTTTAAGAACGTACGCTATGCCTGGGGTCG
AAAGAATAACTTCTTGTGCT**ATGAA**G**TGAATGG**GATGG**ACTGC**GCTTTACCTGTCCCCCTTCGCCAAGGGGTCT
TCGGAAACAGAGCCCTGCTCCTTCCCTCCAA**TCAAGA**GTTCCCCTTTGTCACCCAGGGCCACATCCACGCCGAA
CTCTGCTTCATA**TACTG**GTTCCACGACAAAGTCCTGAGAGTGCTGTCCCCGATGGAAGAG**TTCAAG**GTCACG**TG**
GTACATGTCCTGGAGCCCCTGCAGCAAGTGCGCGGAGCAGGTAGCCAGGTTCCTGGCCGCACACCGCAACCTAA
GCCTG**GCCAT**CTTCAGCTCCCGCTT**GTACT**ACTACTTAAGGAACCCGAACTACCAGCAGAAGCTCTGCAGGCTG
ATTCAGGAAGGAGTCCACGTGG**CTGCCAT**GGACCTACCAGAATTTAAAAAGTGTTGGAACAAGTTTGTGGACAA
TGACGGCCAA**CCATTCA**GGCCTTG**GATGA**GACTGAGAATAAATTTAGTTTCTATGATTGCAAGCTTCAGGAGA
TTTTCAGC

23 596 **3.9**

>A3_C_RATTUS
C**GAATGAAT**CTGCTAAGGG**AAGATG**TATTTTACTTGCAATTTAACAACAGCCACCGGG**TCAAG**CCAGTCCAGAA
TCGCTACTATCGCAGGAAGTCCTATCTGTGCTACCAACTGGAGCGGGCC**AATGG**C**CAAGA**GCCACTCAAAGGCT
ACCTGCTATA**CAAGA**AAGGTGAACAGCATGTAGAAATCCTCTTCCTTGAG**AAGATG**CGGTCCATGGAGCTGAGC
CAAGTGCGAATTACCTGCTACCTCACCTGGAGCCC**CTGCC**CAAACTGTGCCCGGCAACTCGCTG**CATTCAA**AAA
GGATCACCCAGACCTAATTCTGCGGATCTATACCTCCCGCCTGTATTTC**TACTG**GAGGAAGAAGTTCCAGAAGG
GGCTGT**GTACT**CTGTGGCGATCAGGGATCCACGTGGACGTCATGGACCTCCCTCAGTTTGCTG**ACTGC**TGGACA
AACTTTGTGAACCCGCAAAGGCCCTTTAG**GCCAT**G**GAATGAA**CTGGAGAAAAACAGCTGGCGCATACAAAGGCG
GCTCCGGAGGATC

21 527 **4**

>AID_RATTUS
AGCCTCTT**GATGAA**GCAAAAGAAGTTTCTTTACCAC**TTCAA**AAATGTCCGCTGGGCTAAGGGTCGGCACGAGAC
CTACCTGTGCTATGTGGTGAAGAGGAGAGATAG**TGCCA**CCTCCTTCTCACTGGACTTTGGCCACCTTCGCAACA
AGTCGGG**CTGCCA**CGTGGAATTGTTGTTCCTACGCTACATCTCGGACTGGGACCTGGACCCCGGCCGGTGTTAC
CGTGTCACCTGGTTCACTTCCTGGAGCCCCTGCTACGACTGTGCGCGGCACGTGGCTGAGTTTCTG**AGATG**GAA
CCCTAACCTCAGCCTGAGGATTTTCACCGCGCGCCTCTACTTCTGCGAAGACCGCAAGGCTGAGCCTGAGGGGC
TGCGGAGGCTGCACCGCGCCGGAGTCCAGATCGGGATCATGACC**TTCAA**AGACTATTTT**TACTGC**TGGAATACA
TTTGTAGAAAATC**ATGAA**AGAACT**TTCAA**AGCCTGGGAAGGGCTGC**ATGAA**AACTCCGTCAGGCTAACCAGACA
GCTTCGGCGCATCCTTTTGCCCTTGT**ATGAA**GTC**GATGA**CTTGAG**AGATG**CGTTTCGTATTTTGGGACTT

16 584 **2.7**

>AID_FUGU
ATGCTTTT**TGCCAAGA**AAGAAGTTCATCTA**CCATT**A**CAAGAATG**TGCGATGGGCACGGGGCCGGCACGAGACCTA
CCTCTGCTTTGTGGTGAAGAGGCGAGTGGGCCCAGACACGCTAACCTTTGACTTTGGACACCTCCGCAATCGCA
GTGG**CTGCCA**CGTAGAGCTGCTTTTCCTGCGTTACCTGGGAGCCTTATGCCCTGGGTTGTGGGGTTACGGAGCC
GCCGGAGAGAAGAGGCTCAGTTACTCCGTTACCTGGTTCTGCTCCTGGTCTCCCTGTGTCA**ACTGC**TCCATCCA
ACT**CTGCCA**GTTTCTCAATAACACCCCCAACCTTCGCCTCAGGATCTTTGTCTCTCGCCTTTACTTTTGTGACC
TGGAGGACAGCCTTGAAAGAGAAGGCCTGAGGATGCTGACCAAAGCCGGCGTGAGGATCTCAGT**GATGA**GCTAC
AAAGACTATTTCTATTGCTGGCAGAAATTTGTGGATTGCAAAAAGAGCAAC**TTCAAG**GCCTGGGAAGAGCTGCA
TCAAAACTCTGTACGCCTCACCCGCAAACTCAATCG**CATTC**TG

14 557 **2.5**

>A2_FUGU
GAAC**AGATGA**GC**CCATTC**TAC**TTCAAG**TTTCAGTTCAGGAACGTTGAGT**ATTCA**TCAGGGAGGAA**CAAGA**CCCT
GCTGTGCTTCAGAGTGGATTCGCCAGGAGGCAGCACCGAGCCAC**TCAAG**GGTTACATGG**AAGATGAA**CA**TGCCA**
CAGCACATGCAGAGGAGGCCTTCT**TTCAA**CAAGTG**CTGCC**TAATCCATCTCTGGAGTATGACATCAC**ATGGTA**T
GTATCATCCAGTCCGTGTATCTCTTG**TGCCA**ACACGCTGGCA**AAGATG**CTCCAGCAACGCAAAAAAGTCCGCCT
CTGTGTATTCTGCTCCCGGCTCTTTGAGTGGGACCAGCCAGAAGTAGTAGAG**GCCAT**CCGGACCCTGGTGAGGG
CCGGATGCAAACTAAG**GATGATGAA**GCCCTCTGACTTCCAGCATGTGTGGGAAACTTATGTGGAGAAGGAGGGC
GAAAGCTTCAC**GCCAT**GGGAGGATTGTCAGGACAACTACAATTATTACAAGGAGATATTAGCTGATATCC**TCAA**
G

26 515 **5.1**

>A2*_FUGU
TTTCAGTTTCAG**TTCAAGAATG**TAGAATACTCTTCGGGACGGAA**CAAGA**CCTTTCTTTGCTACCTGGTGGACAC
GGGGAAAGCC**GATGA**GGGGCTACAGAGGGGATATTTAGAG**GATGA**GCA**TACTG**GTTCACATGCTGAGGAGGCCT
TCTTCACACAGTGT**CTGCCA**CATTACGACCCCGCCC**TCAAGTACT**CAGTGACC**TGGTA**TATGTCTTCCAGTCCT
TGCTGTGCTTGTGCAGCT**AAGATG**GCTGAAGCATTAAAGGCCAGGAGAAACATTAAGTTGAGCATCTTTGCTGC
TCGTTTATTTGAGTGGGAGGAAGCAGAGATCCAGGCTGGACTGAAAGCTCTACACATTGCTGGCTG**CAAGAT**CA
GAGT**GATGAA**GCCTCTGGACTTCTCTTACACCTGGGACACTTTTGTAGA**GAATG**AGGATCAGCCACTGAACCTC
TGGGCAGATTGCAAAGAAAACTATGA**GTACT**ACCACGAGAGGTTGGCTGA**CATTC**TACAG

22 500 **4.4**

>AID_DANIO
AGTGTGCTCATGACCCAGAAGAA**ATTCA**TCTTCCACTATAA**GAATG**TGCGCTGGGCTCGAGGGAGACACGAAAC
CTACCTTTGTTTTGTAGTAAAGCGACGCATCGGCCCTGATTCCCTCTCTTTTGACTTTGGACACCTGCGCAATC
GCTCCGGA**TGCCAT**GTAGAGCTTCTCTTTCTGCGTCACTTGGGTGCGTTGTGTCCGGGCCTGAGCGCTTCTAGT
GTGGACGGTG**CAAGAT**TGTGTTACTCAGTGACCTGGTTCTGCTCCTGGTCGCCCTGCTCTAAATGTGCTCAACA
GCTCGCCCACTTCCTGTCACAGACGCCCAATCTGAGGCTGAGGATCTTTGTGTCACGCCT**GTACT**TCTGT**GATG**
AAGAGGACAGCGTGGAGAGAGAAGGTCTGCGACACCTGAAGAGGGCAGGAGTTCAGATCTCGGTCATGACTTAT
AAAGACTTTTTC**TACTGC**TGGCAAACGTTTGTTGCGAGGAGGGAGCGGAGTTTTAAAGCCTGGGATGGACTTC**A**
TGAAAACTCTGTCCGGCTTGTTCGGAAACTCAATCGGATTCTG

12 557 **2.2**

>A2_DANIO
GAAGTGCCGATGGAGAACGGAGAGGCCGGAGCTGCTGCTGCAGACGGAGAAAAACCAGAACCTATAGAG**CTGCC**
GCCATTCGAGACCATCACAGGGGATCGCATGGACCCGTTTTTC**TTCAAG**TTCCAG**TTCAAGA**ACGTGGA**GTACT**
CGTCCGGCCGGAATAAAACCTTCCTGTGTTACCTGGTGGACCACGGCGGCGAAGGCCT**GATGA**GGGGCTACATT
GAGGACGAGCACGCTGGCGGACACGCAGAGGAAGCCTTTTTCCAGCAGATCCTGACCAACTACGACCCGGCCTG
CAAATACACCATCACCTGGCACTCCTCCAGTCCCTGCGCCA**ACTGC**GCAACCAAGCTGGCGGAGATCCTGCGCT
CCAGGAAGAACATCCGGCTG**GCCAT**CTTTTCGTCGCGGCTGTTCGAGTGGGAGGAGCCTGAGATCCAGGCAGGG
CTGAAGCTACTAGCGTCCGTCGGCTGCAA**ACTGC**GCAT**GATGAA**GCCGCTGGATTTCACCTACACCTGGGACAC
GTTCGTGGAGAGCGACGAACAGCAGTTCACACCCTGGGAGGACTGTCAGGAGAACTACGA**GTACT**ACCAGGACA
AACTGGCCGACATCCTGCAG

17 608 **2.8**

>A2*_DANIO
AATGGGGAGATACCAGAAGAAGCCAAAGGCCCAGTCAAAAATGAGGAGTTTGAGCTAGAGCCGATGGAG**CTGCC**
GCCATTTGAGATCATTGTAGGAGACCGAATAAACCCAACCTTC**TTCAAG**TTCCAATTTAAAAATGTGGA**GTACT**
CGTCCGGGCGCAA**CAAGA**CCTTCCTGTGCTACCAGGTCGACATACAAGGTGGAGCAGCAGAGACGGATGGCGTT
CGTGGTTACCTGGAG**GATGAGCATTCA**GGCTCACATGCCGAAGAGGCCTTCT**TTCAA**CAGGTGCTGGCCTATTA
TGACAAGTCCCTCCGCTATACAGTGACT**TGGTAC**ACATCGTCCAGCCCTTGTGTGGCCTGCGCTGCTAAGCTTG
TGGAGATCTTGAAAGCACGCAAGGCGCTGCGCCTTAATATCC**ACTGC**TCCCGTCTCTT**TGAATGG**GAGGAACCG
GAGATACAGGCCGGACTTCAGGCGCTAGTTAGGGCAGGGTGTAAACTACGCAT**GATGA**GGCCTGTGGATTTCGT

GTATGTCTGGTCTTTTGTTGAGAACGAAG**AAGAT**AATTTTACACCCTGGGAAGACTG**TCAAGAT**AACTTTGAGT
ATTAT**GATGA**GAGATTGCAGGATATTTTAAAG

24 620 **3.9**

>A2_GALLUS
ATGGCAGAAAAGCAGGAGGAGCCCAGCAACACCCAGAACGGTGAACCCGACAACGCAGAGGAGGGTGAGGGGAA
AAAGAAGAAGAAGTTGAAGAGGG**AAGATCTGCC**TCCGTTTGAAATCGTGACAGGGGAACGCCTCCCA**GCCAT**AT
TT**TTCAAG**TTCCAGTTCAGGAACGTGGAATACAGCTCAGGCAGGAACAAAACCTTCCTGTGTTATGTTGTAGAG
ACCCAAGGCAAAGAGTCAAAGACCTCCCGGGGTTACCTGG**AAGATGA**GCATGCAGCCTCCCATGCAGAAATTGC
TTTCTTTAACACGATTT**TGCCA**AAGTG**TGAATCAAG**CCTCCGCTACAACATCACC**TGGTAC**GTCTCCTCCAGCC
CCTGCGTGACCTGTGCTGATCGGATAAGTGAGACCCTAAGGAAGAA**CAAGA**ACCTGCGTCTGACAATC**ATGGTA**
GGCCGCCTCTTCATGTGGGAAGAGCCAGAAATGCAGG**CTGCC**CTGAAAAAA**ATGAA**GTCTGCTGGCTGCAAGCT
GAGGATT**ATGAA**GCC**TCAAGA**CTTTGAGTACGTCTGGCAGAACTTTGTGGAACAGGAAGAAGGAGAGGAAGCAA
AGGCTTTCGTGCCGTGGGAGGA**CATTCAAGA**GAACTTCCAATACTATGAGGAAAAGCTGGCTGAGATTCTGCAG

26 662 **3.9**

>AID_GALLUS
ATGGACAGCCTCTTG**ATGAA**GAGGAAGCTCTTCCTCTACAAT**TTCAAGA**ACCTGCGCTGGGCCAAAGGCCGTCG
TGAAACCTACCTCTGTTATGTTGTGAAGCGCCGTGACAGTGCTACATCATGCTCCCTGGACTTTGGATACCTGC
GTAA**CAAGATG**GGT**TGCCAT**GTGGAGGTTCTCTTCCTACGCTACATCTCAGCTTGGGACCTGGACCCAGGCCGC
TGCTACCGCATCAC**ATGGT**TCACCTCCTGGAGCCCCTGTTATGACTGTGCCCGACATGTGGCTGACTTCCTTCG
TGCCTACCCAAACTTGACCCTCCGCATTTTC**ACTGCC**CGCCTCTACTTCTGTG**AAGAT**CGCAAGGCTGAGCCTG
AGGGGCTGAGACGCCTGCACCGGGCTGGGGCCCAAATC**GCCAT**CATGACT**TTCAAAGAT**TTCTTC**TACTGC**TGG
AACACGTTTGTGGAGAACAGGGAAAAGA**CATTCAA**AGCCTGGGAAGGGCTGC**ATGAA**AACTCTGTCCATCTGTC
CAGGAAACTCCGACGGATCCTT**CTGCCA**CTGT**ATGAA**GT**AGATGA**TTTACG**AGATG**CCTTTAAAACTCTGGGAC
TT

29 590 **4.9**

>A2_XENOPUS
ATGGCACAGAGGCAAAATA**ATTCA**CAGTCTTCCAAGGATCCTATCAATAACACAGAGAAAGCAGAGAAAGAAGA
GAAAGCAGAA**AATGG**CGAGGGAAATTCCAAAAAGAAAGAACTAGAGGAGTTGCCTCCCTTTGAAATAGTGGAGG
GGAGTCGCATCCCTGCTTCTTCTTTCATGTTTCAGTTTAAGAACGTGGAGTACAGTTCTGGACGTAA**CAAGA**CT
ATTCTGTGCTACACTGTGGAAAGACCTGAGGCTCAAATATTCCATGGATATTTAGAG**GATGAA**CATGTCTCAGC
TCATGCCGAGGAAGCTTTCTTTACATCTGTC**CTGCC**TCAGTTTCTCACCTCAGGGTCAGTCACTGTAACTTGCT
ATGTGTCATCTAGTCCATGCGTTACTTGTG**CTGCC**TCCATAGCTCAATGCCTGCGGAAAAACAAAACTGTACGA
ATACAGCTGGCAGTGGCCCGGCTATTCC**AATGG**GAGGAGCCCGAGATCCGAAGGGCCCTGAAAGGCCTCCGCTC
CGCAGGT**TGCCA**AGTCA**GAATGATGA**GGGGTGCTGACTATATTTATGTGTGGAAGAACTTTGTTGAGCCTGACA
TCACCCTG**GATGA**TGAGG**GAATGG**AGGTAGTGAAG**GATGA**GCA**TCAAGA**CTTCATACCCTGGG**AAGAT**TTGGAG
GAAAACTCCAGGTATT**ATGAA**GAGAAGCTGGCAGAGATTCTAAGA

19 707 **2.7**

>A2_ORYZIAS_BJ502566
TCTGCTGCTGCAGTGGAAAACAGTGA**GAATGG**CACACTTCAGTTTGAGCCCATGGAGCTACCCCCTTTTGAAGT
CATCACTGGAGACCGGATCGAC**CCATT**TGTC**TTCAAG**TTCCAG**TTCAA**AAATGTGGA**GTACT**CGTCCGGCCGCA
A**CAAGA**CGCTGCTGTGTTACCTGGTTGA**TACTG**GGGGTGCAGGGGATGGGCTGCTAAGGGGATACCTGGAG**GAT**
GAACACGCTGGGATCCACGCTGAGGAGG**CATTC**TTCACACACTGT**CTGCC**GAACATTGACCCCAATGTTCAGTA
TACAATAACC**TGGTAC**GTGTCGTCCAGCCCT**TGCCA**CGCTTGTTCCTTAAAGCTAGCAGAGGTGCTGAAGGCCA
GGAAAAATG**TCAAG**CTCAGCATCTTTTCTGCTCGCCTGTTCGAGTGGGAGGCAGAGGAGGTCCAGGCTGGTCTG
AAGGCCCTGCACAGCGCCGGCTGCAAGCTTAGAGT**GATGAA**GCCTCTGGACTTCTCTTACACCTGGGACACCTT
TGTGGAAACTGAAGAGCAACCGCTGAACCTTTGGGAGG**ACTGC**A**AAGATNA**CTATGA**GTACT**ACCACGAGAAGC
TGGACCAGATTCTGCAC

24 605 **4**

>A1_MONODELPHIS
ATGAATTCTAAGACAGGTCCATCAGTAGGAGACGCCACTCTAAGGAGGAGGATCAAACCCTGGGAATTTGTGGC
CTTCTTTAACCC**TCAAGA**ACTCCGTAAAGAGACATGTCTCCTCT**ATGAA**ATTA**AATGG**GGTAATCAAAATATTT
GGCGACACTCCAATCAAAATACATCTCAACATGCTGAAATCAACTTCATGGAAAA**ATTCACTGC**AGAAAGACAT
TTTAACTCTTCTGTT**AGATG**CT**CCATT**ACCTGGTTCCTCTCTTGGAGCCCTTGCTGGGAGTGTTCCAAG**GCCAT**
AAGAAAGTTTTTGGATCATTACCCCAATGTGACTCT**TGCCATT**TTTATTTCTCGGCTTTATTGGCACATGGATC
AACAGCATCG**TCAAG**GAC**TCAAG**GAACTTGTCCACTCTGGAGTGACTATCCAGATTATGAGCTACTCAGAGTAT
CAT**TACTG**TTGGAGAAATTTTGTTGACTATCCACAAGGGGAAG**AAGATTACTG**GCCCAAATACCCCTACCTTTG
GATTATGTTATATGTCCTGGAACTCC**ACTGC**ATAATTTTAGGGCTTCCACCTTGTTTA**AAGAT**TTCAGGGAGT**C**
ATTCCAATCAGCTTGCGCTCTTCAGCCTTGATTTG**CAAGACTGCCATT**ACCAA**AAGAT**TCCATATAATGTTCTT
GTAGCCACAGGGCTGGTCCA**GCCATT**TGTGACCTGGAGA

30 701 **4.3**

>A1_ORYCTOLAGUS
ATGGCTTCCGAGAAAGGTCC**TTCAA**ACAAGGATTACACGTTGAGGAGAAGAATTGAACCCTGGGAATTTGAAGT
CTTCTTTGACCCC**CAAGAACTGC**GTAAAGAGGC**CTGCC**TGCTCTACGAAA**TCAAG**TGGGGGGCAAGCTC**CAAGA**
CCTGGCGCAGCTCGGG**CAAGA**ACACCACCAATCACGTGGAAGTCAACTTCCTAGAAAAGCTGACTTCAGAGGGG
CGGCTTGGCCCATCCACCTGCTGCTCCATCACCTGGTTCTTGTCTTGGAGCCCCTGCTGG**GAATG**TTCCATGGC
GATTAGAGAATTTTTGAGTCAACACCCGGGTGTGACTCTGATAATTTTTGTAGCTCGGCTATTTCAGCACATGG
ATCGCCGGAACCGGCAAGGGC**TCAAG**GACCTCGTTACCAGCGGCGTAACTGTCCGGGTTATGAGCGTCTCAGAG
TATTGT**TACTGC**TGGGAGAATTTTGTCAACTACCCGCCGGGGAAAGCAGCTC**AATGG**CCAA**GGTAC**CCACCTCG
GTGGATGCTGATGTATGCACTGGAACTC**TACTGC**AT**CATTC**TAGGTCTCCCACCCTGTTTA**AAGATTTCAAGA**A
GACATCAAAAGCAGCTTACATTTTTCAGCCTTACTCCTCAATAT**TGCCATT**ATAAAATGATTCCACCATACATC
CTTTTAGCTACAGGATTGTTACAACCTTCTGTGCCTTGGAGA

23 704 **3.3**

>A3A_EQUUS
ATGGAGGCCAGTGCAGCGCCCATGGCCAGACGCCTGATGCCTGAAGACACCTTCACTGAGAAC**TTCAA**CAATGT
GGTTTGGCAGAAGGCGACCTATCTGTGCTATGAGGTGGAGCTCCCGGATGGCGACTACAGGGTCCCGCCAGGCC
AGGACAAGGGCTTCCTGCACAACCAGGGTGGTCACATGGCCGGGCCACCCCGCCACGCAG**AGATG**CGCTTCCTG
GACCTGATCTCTTCTTGGAACCTGGACCAGGATCTGTGCTACAGGGTCACCTGTTTCATCTCCTGGAGCCCCTG
CGCTGAGTGTGCTCAGAGACTGGCTGAGTTCCTGCGGGAGAACAGCCACGTGAGCCTGCGCATCTTCGCTTCCC
GCATCTACACCAAGGGAGA**GAATGG**AGACTACAGGGAGGG**ACTGC**GCACCCTGCAGGAGGCTGGGGCCCAAATC
GCCATCATGACCTCAAAAGAGTTTGAGC**ACTGC**TGGAAGACCTTCGTGGACAACCAGGGAAGGACCTTCCAGGC
CTGG**GATGA**GCTGGATGCTGAGAGTCGC**TACTG**GTCCATGGAGCTGCAGCG**CATTC**TACAGCCG**ACTGC**TCCTC
CCTCC**CTGCC**TCCCCTCTACTTTCCTCCCTCCCATCCTGTCCCTGGCCCTTCCTTTCGCTCTCTCAGAGGCTCC
TCTGGG

12 668 **1.8**

>A3B_EQUUS
ATGGAGGCCAGGTGCCTCATG**GATGAA**GACACCTTCACTGAGAAC**TTCAA**CAATGTGAACTGGCCACG**CAAGA**C
CTACCTGTGCTATGAGGTGGAGCTCCCGGATGGCGACTCCAGGGTCCCGCCAGGCCGGGACAAGGGCTTCCTGT
GCAACAAGCCTGTTGATGTGGCTGGGCCACGCCGCGACGCAG**AGATG**CGCTTCCTGGACCTGATCTCTTCTTGG
AACCTGGACCAGAAGCTGTGCTACAGGGTCACCTGTTTCATCTCCTGGAGCCCCTGCGCTGACTGTGCTCAGAG
ATTGGCTGAGTTCCTGCGGGAGAACAGCCACCTGAGCCTGCGCATATTCGCTTCCCGCATCTACAC**CAAGA**GAA
AATATGGAGACTACGGGGCGGG**ACTGC**GCACCCTACAGGCGGCTGGGGCCCAAATC**GCCAT**CATGGCCTCAGAA
GAGTTTGAGC**ACTGC**TGGGACACCTTCGTGGACCACCAGGGAAGACCCTTCCAGCCC**TGGTA**TGCGCTGGA**TGC**
CAAGAGCCGTTACCTATCTGAGCAGCTGCAGCG**CATTC**TACAGCCG**ACTGC**TCCTCCCTCC**CTGCC**TCCCCTCT
ACTTTCCTCCCTCC**CTGCC**TCTCCCTCTACTTTCCTCCCTCCCATCCTGTCCCTGGCCCTTCCTTTCGCTCTCT

16 662 **2.4**

>A3A_BOS
GCCGCGGCTCGAGCAACAA**TCAAG**GGTCGAGGTTCCAAAACCTACCTGTGCTACGAGGTGGAGAGGCTGGACGG
AAACGCTACGATCCCTCTGGACGAGTACAAGGGCTTTGTGCGCAACAAGGGTTTGGATCAACCGGAGGAACC**CT**
GCCATGCAGAGCTCTACTTCCTGGGA**AAGAT**CCGTTCTTGGAATCTGGACCGGAATCAGCACTACAGGCTCACC
TGTTTCATCTCCTGGAGCCCCTGTTACGATTGTGCCCAGAAACTGACTA**CATTC**TTGAAGGAGAACCGCCACAT
AAGCCTGCACATCCTTGCCTCCCGCATCTATACCCGCAA**CCATT**TTGGG**TGCCAT**CAGAGTGGCCTGTGCGA**AC**
TGCAAGCGGCTGGGGCCCGAATCACCATCATGACCTTTGAGGACTTTAAGC**ACTGC**TGGGAAACCTTCGTGGAC
CACAAGGGAAAACCCTTTCAGCCCTGGGAGGGGCTAAATGTAAAGAGTCAGGCGCTCTGTGCGGA**ACTGC**AG**GC**
CATTCTCAAGACTCAGCAAAAC

17 536 **3.2**

>A3_C_SUS
AACACA**ATGAAT**CTACTAAAAGAAAATAT**ATTCAA**ACAACAGTTTGGCAACCAGCCCCGGGTCTCGGCGCCCTA
CTATCTGAGGAAGACCTACTTGTGCTACCAAGTGAAGGGGCCC**GATGA**CT**CCATTC**TTGACAAAGGCTGCTTCC
AAAA**CAAGA**AAAAGCGACATGCAGAAATTCGTTTTATTGA**CAAGAT**CAATTCCT**TGAAT**CTGGACCAGAACCAG
TGCTACAGAATCATCTGCTATGTCACCTGGAGCCCTTGTCACAACTG**TGCCA**AGGAGCTGGTTGATTTCATCAG
CAATCGGCACCACCTGAGCCTGCAGCTCTTTGCCTCCCGCCT**GTACT**TCCACTGGGTCAGGTGTTATCAGAGGG
GTCTGCAGCGTCTGCAGGCAAATCGGGTTTCCGTGGCTGTC**ATGAA**AGGCCCAGAG**TTCAA**AG**ACTGC**TGGGAA
AAGTTTGTGGACCCCCAGGGGGAGAGCTTCCCATCCTGGGAGAAGCTGGAGCAATACAGCGAGAGCATAAGCCG
ACGGCTC**TCAAG**GATCCTGCGTTTCGCGAATCAGAATAATTTAGAGGATTCCTTCAGAGATTTGCGACTCGGTT
CCCCATCACCTTCATCA**TCAAGA**AGTGACTCGAGA

19 623 **3**

>Sus scrofa cytidine deaminase (CDA), mRNA NCBI Reference Sequence:
NM_001244385.1

CACACTGGCTGTGAGGTCTCTTTCCGATCCT**CTGCCA**GCCCCAGGTGGACCAACATGGCC**CAAGA**GCGTC
CCAGTCCCGCCTTGGAGCCCGAGCACGTCCAGCAGCTGCTCTTCTC**CTGCC**GGGAGGCCAAGAAGTCCGC
C**TACTGCC**CCTACAGTCGCTTCCCGGTGGGGGCGGCCCTCCTCACCGGGGACGGGAGGATTTTCTCCGGG
TGCAACATAGAAAATGCCTGCTACCCGCTGGGCGTCTGTGCTGAACGGACC**GCCAT**CCAGAAG**GCCAT**CT
CAGAAGGATACAAAGAGTTCAGGGCAAT**TGCCAT**CGCCAGTGATCTG**CAAGATGA**TTTTATTTC**GCCAT**G
CGGGGCCTGCAGGCAAGTCATGAGAGAGTTTGGCACCAACTGGGCCGTCTACATGACCAAGCCGGATGGC
ACATACGTTGTCAGGACCATCCAGGAGCTGCTAACGGCCTCCTTTGGGCCCGAGGACCTGCAG**AAGAT**CC
AGTGAAAGGTG

17 497 **3.4**

>CDA_FUGU
ATGGCGAACAAAAACGAGGCGGCGCAATTATCGGCAGAAACCA**TCAAGATG**CTGATCGACCAATCTCACGAAGT
GAAAAAGCACTCACACTGTCCTTACAGTAA**ATTCA**GAGTGGGGTCTGCTCTTCTGACCGTCGACAACCGCGTGA
TTACAGGCTGCAATGTGGA**GAATG**CATGTTTCTACCTGGGATTTTGTGCTGAAAGAAACGCTATAGCCAAGGCA
GTGTCAGAAGGGAGCAAAAA**ATTCAAGGCCATT**GCGAT**TGCCA**GTGACATGACTGACCAGTTCATCTCCCCGTG
TGGTGCCTGCAGGCAGTACATGCGGGAGTTTGGGACAAACTGGAGAGTGTTCCTGTCAAAACCCGACGGTTCTT
ACCTGGAGAGAACACTAGAGGACCTGCTTCCCGTTTCCTTCGGGCCCG**AAGAT**

13 419 **3.1**

>CDA_HUMAN_978
ATGGCCCAGAAGCGTC**CTGCC**TGCACCCTGAAGCCTGAGTGTGTCCAGCAGCTGCTGGTTTGCTCCCAGGAGGC
CAAGAAGTCAGCC**TACTGCC**CCTACAGTCACTTTCCTGTGGGGG**CTGCC**CTGCTCACCCAGGAGGGGAGAATC**T**
TCAAAGGGTGCAACATAGAAAATGCCTGCTACCCGCTGGGCATCTGTGCTGAACGGACCGCTATCCAGAAGGCC
GTCTCAGAAG**GGTAC**AAGGATTTCAGGGCAATTGCTATCGCCAGTGACATG**CAAGATGA**TTTTATCTCTCCATG
TGGGGCCTGCAGGCAAGTCATGAGAGAGTTTGGCACCAACTGGCCCGTGTACATGACCAAGCCGG**ATGGTAC**GT
ATATTGTCATGACGGTCCAGGAGCTG**CTGCC**CTCCTCCTTTGGGCCTGAGGACCTGCAGAAGACTCAG

16 434 **3.7**

>DCMPDA_FUGU_Q9BVD8
GTCTCTACCGGGAAAAGAGAAGACTACCTGGAGTGGCCCGAATACTTCATGGCTGTAGCCTTCCTGTCTGCACA
AAGGAGTAAAGATCCAAGTTCACAGGTGGGAGCATGTATCGTGAACCAAGAGAAAAAAATAGTCGGTATTGGTT
ATAATGGTATGCCGAATGGCTGCGATGACGACCTGTTGCCTTGGTGCCGTTCCGCCGAAGACCATCTCGACACC
AAATATCCTTATGTGTGTCACGCTGAGATGAACGCCATCATGAACCAAGAACAGTGCCGACGTGAAAGGCTGCGC
CATGTACGTGGCTTTGTTCCCCTGCAACGAGTGCGCCAAGCTCATCATCCAGGCAGGTCTGAAAGAGGTGATCT
ACCTCTCCGATAAGTACCACAACACCCCAGGAATGATCGCTTCCAGAAAGTTGCTTGACTTGGCTGGGATTCAG
TACAGG

17 446 3.8

>DCMPDA_HOMO_1635
ATGAGTGAAGTTTCCTGCAAGAAACGGACGACTATTTGGAATGGCCAGAGTATTTTATGGCTGTGGCCTTCTT
ATCAGCACAGAGAAGCAAAGATCCAAATTCCCAGGTCGGCGCCTGCATCGTGAATTCAGAAAACAAGATTGTCG
GGATTGGGTACAATGGGATGCCAAATGGGTGCAGTGATGACGTGTTGCCTTGGAGAAGGACAGCAGAGAATAAG
CTGGACACCAAATACCCGTACGTGTGCCATGCGGAGCTGAATGCCATCATGAACAAAAATTCGACCGATGTGAA
AGGCTGTAGTATGTATGTCGCCTTGTTCCCTTGTAATGAATGCGCTAAGCTCATCATCCAGGCAGGTATAAAAG
AAGTGATTTTCACGTCTGATAAATACCATGATAGTGACGAGGCAACTGCTGCGAGGCTCCTGTTTAATATGGCC
GGGGTGACATTCCGGAAATTCATACCGAAGTGCAGCAAGATTGTCATTGACTTTGATTCAATTAACAGCAGACC
GAGTCAAAAGCTTCAG

30 530 5.7

>ADAT2_HOMO_NM_182503
ATGGTCTACAACAATGAAGTTGTAGGGAAGGGGAGAAATGAAGTTAACCAAACCAAAAATGCTACTCGACATGC
AGAAATGGTGGCCATCGATCAGGTCCTCGATTGGTGTCGTCAAAGTGGCAAGAGTCCCTCTGAAGTATTTGAAC
ACACTGTGTTGTATGTCACTGTGGAGCCGTGCATTATGTGTGCAGCTGCTCTCCGCCTGATGAAAATCCCGCTG
GTTGTATATGGCTGTCAGAATGAACGATTTGGTGGTTGTGGCTCTGTTCTAAATATTGCCTCTGCTGACCTACC
AAACACTGGGAGACCCATTTCAGTGTATCCCTGGATATCGGGCTGAGGAAGCAGTGGAAATGTTAAAGACCTTCT
ACAAACAAGAAAATCCAAATGCACCAAAATCGAAAGTTCGGAAAAAGGAATGTCAGAAATCT

14 428 3.3

>ADAT2_FUGU_Q9CX14
GCCAAAGATGCTCTGCAGAATGGAGAAGTGCCAGTTGGGTGTCTGATGGTTTACAACAGTGAAGTTGTTGGCAA
AGGGAGGAATGAAGTCAACGAGACGAAAAACGCCACCCGTCATGCTGAGATGGTGGCCCTGGATGAGCTCCTTA
ACTGGTGTCACAGCAGCAACTTGGACGTGAGCAGAGTGAGCAGGAACACGGTTCTGTACGTCACCGTTGAACCG
TGTGTAATGTGTGCTGCTGCACTGCGTCTGTTGAACATCCCCACAGTCGTGTACGGCTGCAGGAACGAGCGGTT
TGGAGGCTGTGGTTCCATTCTGGATGTTTCCTCTGCAGATTTACCTCAGACTGGAAACACCTTTAAG

13 359 3.6

>Homo sapiens cytidine deaminase (CDA), mRNA NCBI Reference Sequence:
NM_001785.3
CCGCTGCTCTGCTGCCTGCCCGGGGTACCAACATGGCCCAGAAGCGTCCTGCCTGCACCCTGAAGCCTG
AGTGTGTCCAGCAGCTGCTGGTTTGCTCCCAGGAGGCCAAGAAGTCAGCCTACTGCCCCTACAGTCACTT
TCCTGTGGGGGCTGCCCTGCTCACCCAGGAGGGGAGAATCTTCAAAGGGTGCAACATAGAAAATGCCTGC
TACCCGCTGGGCATCTGTGCTGAACGGACCGCTATCCAGAAGGCCGTCTCAGAAGGGTACAAGGATTTCA
GGGCAATTGCTATCGCCAGTGACATGCAAGATGATTTTATCTCTCCATGTGGGGCCTGCAGGCAAGTCAT
GAGAGAGTTTGGCACCAACTGGCCCGTGTACATGACCAAGCCGGATGGTACGTATATTGTCATGACGGTC
CAGGAGCTGCTGCCCTCCTCCTTTGGGCCTGAGGACCTGCAGAAGACCCAGTGACAGCCAGAGAATGCCC
ACTGCCTGTAACAGCCACCTGGAGAACTTCATAAAGATGTCTCACAGCCCTGGGGACACCTGCCCAGTGG
GCCCCAGCCCTACAGGGACTGGGCAAAGATGATGTTTCCAGATTACACTCCAGCCTGAGTCAGCACCCCT
CCTAGCAACCTGCCTTGGGACTTAGAACACCGCCGCCCCCTGCCCCACCTTTCCTTTCCTTCCTGTGGGC
CCTCTTTCAAAGTCCAGCCTAGTCTGGACTGCTTCCCCATCAGCCTTCCCAAGGTTCTATCCTGTTCCGA
GCAACTTTTCTAATTATAAACATCACAGAACATCCTGGA

30 805 3.7

>Saccharomyces cerevisiae DNA, contig: contig11, whole genome shotgun sequence GenBank: BEGW01000011.1: 423374-423802 cytidine deaminase

ATGAAAGTAGGTGGCATAGAAGACAGACAACTGGAAGCCTTAAAAAGGGCGGCACTGAAGGCGTGTGAAC
TCTCCTATAGCCCCTATTCTCACTTCCGTGTCGGTTGTTCCATATTAACAAATAACGATGTTATCTTCAC
TGGTGCTAATGTCGA**GAATG**CAAGCTACAGTAATTGTATATGTGCAGAACGATCTGCTATGATACAAGTT
CT**AATGG**CAG**GCCAT**CGCTCAGGATGGAAATGC**ATGGT**CATTTGTGGCGATTCTGAGGACCAGTGTGTTT
CCCCTTGCGGCGTTTGCAGGCAGTTTATCA**ATGAAT**TTGTGG**TCAAG**GACTTTCCAATAGTCATGCTTAA
TTCCACAGGTTCTCGTTCTAAAGTCATGAC**AATGG**GAGA**ACTGCTGCC**CATGGCTTTTGGTCCATCTCAT
TTAAACTAG

11 425 **2.6**

Observed AL pentamer frequency: 4.49
Expected: 22/1024 ± 1.65x$(22x1002/1024^2x100)^{1/2}$ ≈ 2.15% ± 0.25%*

4.49% ≈ 2.15% + 15 σ, where σ ≈ 0.15%

P(Observed ≥ 4.45%) ≤ exp(-225) ≈ $(e^{-2.25})^{100} \approx 10^{100}$

Caulobacter crescentus CB15 strain CB15 5S ribosomal RNA, complete sequence NCBI Reference Sequence: NR_075118.1

GACCTGGTGGCTATGCCGGGGGTTCCCCACCCGATC**CCATTC**CGAACTCGGTCGTTAAGTCCCCCTGGGC
C**AATGGTACT**TCGTC**TCAAG**GCGCGGGAGAGTAGGTCGCCGCCAGG

8 112 **7.2**

Thermotoga maritima MSB8 strain MSB8 5S ribosomal RNA, complete sequence NCBI Reference Sequence: NR_103224.1

ATCCCCGGGTGCCGA**TACTG**GGGCGGGAAACACCCGGTT**CCATTC**CGAACCCGGCCGTTAAGCCGCCCTG
GGCCG**ATGGTA**GTATGGGGGCAGCCCCATGCGAGAGTAGGTAGCGCCCGGGGATTTGT

5 124 **4**

Helicobacter pylori 26695 strain 26695 5S ribosomal RNA, complete sequence NCBI Reference Sequence: NR_075103.1

TCCCTTTTCCTTGTGCCTTTAGAGAAGAGGAACTACCCAGTTAA**CCATTC**CGAACCTGGAAG**TCAAG**CTC
TTCATCGCTGATAA**TACTGC**TCTT**TTCAAGA**GTGG**GAATG**TAGGTCGGTGCAGGGATAGGGAAAT

9 131 **6.9**

Pseudomonas aeruginosa PAO1 strain PAO1 5S ribosomal RNA, complete sequence NCBI Reference Sequence: NR_075116.1

TGCTTGACGATCATAGAGCGTTGGAACCACCTGATCCCTTCCCGAACTCAGAAGTGAAACGACGCATCGC
CG**ATGGTA**GTGTGGGGTCTCCCCATGTGAGAGTAGGTCATCG**TCAAG**CTC

3 116 **2.6**

Mycolicibacterium smegmatis MC2 155 5S ribosomal RNA, complete sequence NCBI Reference Sequence: NR_075427.1

GTTACGGCGGTCCATAGCGGCAGGGAAACGCCCGGTCCCATCCCGAACCCGGAAGCTAAGC**CTGCCA**GCG
CC**GATGA**TACTACCCTTCCGGGTGGAAAAGTAGGACACCGCCGAACAC

3 114 **2.6**

Methanosarcina sp. 795 genome GenBank: CP011449.1: c670055-669934 5S rRNA

TGTGGCG**GCCAT**AGCGGCAGGGCAACTCCTGTACCCATCCCGAACACAG**AAGAT**AAGC**CTGCC**CACGTTC
CTGACT**GTACTG**AAGTGCGCGAGCCTTCGGGAACTCAGGATCG**CTGCCAT**GC

8 118 **6.8**

Escherichia coli str. K-12 substr. MG1655 strain K-12 5S ribosomal RNA, complete sequence NCBI Reference Sequence: NR_103249.1

TGCCTGGCGGCCGTAGCGCGGTGGTCCCACCTGACCCCATGCCGAACTCAGAAGTGAAACGCCGTAGCGC
CG**ATGGTA**GTGTGGGGTCTCCCCATGCGAGAGTAGGGA**ACTGCCA**GGCAT

5 116 **4.3**

Bacillus subtilis subsp. subtilis str. 168 strain 168 5S ribosomal RNA, complete sequence NCBI Reference Sequence: NR_103286.1

TCTGGT**GATGA**TGGCGAAGAGGTCACACCCGTTCCCATGCCGAACACGGAAGTTAAGCTCTTCAGCGCCG
ATGGTAGTCGGGGGTTTCCCCCTGTGAGAGTAGGACATCGCCAGGC

3 112 **2.7**

Mycobacterium tuberculosis H37Rv strain H37Rv 5S ribosomal RNA, complete sequence NCBI Reference Sequence: NR_103329.1

TTACGGCGGCCACAGCGGCAGGGAAACGCCCGGTC**CCATTC**CGAACCCGGAAGCTAAGC**CTGCCA**GCGCC
GATGATACTGCCCCTCCGGGTGGAAAAGTAGGACACCGCCGAACA

8 111 **7.2**

Salmonella enterica subsp. Enterica serovar Typhimurium str. LT2 strain LT2; SGSC 1412; ATCC 700720 5S ribosomal RNA, complete sequence NCBI Reference Sequence: NR_075120

TGCCTGGCGGCACTAGCGCGGTGGTCCCACCTGACCCCATGCCGAACTCAGAAGTGAAACGCCGTAGCGC
CG**ATGGTA**GTGTGGGGTCTCCCCATGCGAGAGTAGGGA**ACTGCCA**GGCAT

5 116 **4.3**

Pseudomonas putida KT2440 strain KT2440 5S ribosomal RNA, complete sequence NCBI Reference Sequence: NR_075145.1

TTCTTGACGACCATAGAGCATTGGAACCACCTGATCCCATCCCGAACTCAGTAGTGAAACGATGCATCGC
CG**ATGGTA**GTGTGGGGTTTCCCCATGTGAGAGTAGGTCATCG**TCAAGAT**T

5 116 **4.3**

Agrobacterium fabrum str. C58 strain C58 5S ribosomal RNA, complete sequence NCBI Reference Sequence: NR_075124.1

GACCTGGTGGTTATCGCGGGGCGGCTGCACCCGTTC**CCATTC**CGAACACGGCCGTGAAACGCCCCAGCGC
C**AATGGTACT**CCGTC**TCAAGA**CGCGGGAGAGTAGGTCG**CTGCCA**GGTCT

11 115 **9.6**

Bradyrhizobium japonicum USDA 110 strain USDA110 5S ribosomal RNA, complete sequence NCBI Reference Sequence: NR_075299.1

GGCCTGGTGGTTTTAGCGAAGAGCCTCAACCCGATCCCATCCCGAACTCGGCCGTTAAACTCTTCAGCGC
C**AATGGTACT**ATGGCTTAAGCCCTGGGAGAGTAGGTCG**CTGCCA**GGCCT

7 115 **6.1**

Corynebacterium glutamicum ATCC 13032 strain ATCC 13032 5S ribosomal RNA, complete sequence NCBI Reference Sequence: NR_075297.1

TGTGTCGGTGGTGATAGTAGCAGGGAAACGCCCGGTCCCTTTCCGAACCCGGAAGCTAAGCCTGGTTACG
CTG**ATGGTACTGC**ACTCGGGAGGGTGTGGGAGAGTAGGTTACCGCCGACC

6 116 **5.2**

Archaeoglobus profundus DSM 5631 strain DSM 5631 5S ribosomal RNA, complete sequence NCBI Reference Sequence: NR_075715.1

GCCGACG**GCCAT**AGGGGTCGGGGAAACACCCGGACT**CATTC**CGAACCCGGAAGTTAAGCCCGACCCCGTT
CCGCG**TGGTACTG**TGTTCCGAGAGGGCACGGGAAGCGCGGAAGCCGTCGGCA

6 118 **5.1**

Burkholderia pseudomallei K96243 strain K96243 5S ribosomal RNA, complete sequence NCBI Reference Sequence: NR_075304.1

TGCCT**GATGA**CCATAGCGAGTCGGTCCCACCCCTTCCCATCCCGAACAGGACCGTGAAACGACTCCACGC
C**GATGA**TAGTGCGGATTGCCCGTGTGAAAGTAGGTAATCGTCAGGC

2 112 **1.8**

Bacillus cereus ATCC 14579 strain ATCC 14579 5S ribosomal RNA, complete sequence NCBI Reference Sequence: NR_075158.1

TTTGGT**GATGA**TGGCAGAGAGGTCACACCCGTTCCCATACCGAACACGGAAGTTAAGCTCTCTAGCGCCG
ATGGTAGTTGGGACCTTGTCCCTGTGAGAGTAGGACGTCGCCAAG

3 111 **2.7**

Streptococcus pneumoniae R6 strain R6 5S ribosomal RNA, complete sequence NCBI Reference Sequence: NR_075123.1

TAAGTGACGATAGCCTAGGAGATACACCTGTACCCATGCCGAACACAGAAGTTAAGCCCTAGAACGCCGG
AAGTAGTTGGGGGTTGCCCCCTGTGAGATAGGGAAGTCGCTTAGC

0 **0**

Shewanella oneidensis MR-1 strain MR-1 5S ribosomal RNA, complete sequence NCBI Reference Sequence: NR_075142.1

TGCTTGGTGACAATAGCATTGTGGTCCCACCTGATCCCATCCCGAACTCAGAAGTGAAACGCAATCGCGC
CG**ATGGTA**GTGTGGGGTCTCCCCATGTGAGAGTAGGTCATCGCCAAGCGC

2 116 **1.7**

Aster altaicus chloroplast, complete genome NCBI Reference Sequence: NC_034996.1: c129302-129182 5S ribosomal RNA

TATTCTGGTGTCCTAGGCGTAGAGGAACCACACCAATCCATCCCGAACTTGGTGGTTAAACTC**TACTGC**G
GTGACGA**TACTG**TAGGGGAGGTCCTGCGGAAAAATAGCTCGACGCCAGGAT

3 117 **2.6**

Datura stramonium chloroplast, complete genome NCBI Reference Sequence: NC_018117.1: c132806-132686 5S ribosomal RNA

TATTCTGGTGTCCTAGGCGTAGAGGAACCACACCAATCCATCCCGAACTTGGTGGTTAAACTC**TACTGC**G
GTGACGA**TACTG**TAGGGGAGGTCCTGCGGAAAAATAGCTCGACGCCAGGAT

3 117 **2.6**

Rubus takesimensis chloroplast, complete genome NCBI Reference Sequence: NC_037991.1: c132382-132262 5S ribosomal RNA

TATTCTGGTGTCCTAGGCGTAGAGGAACCACACCAATCCATCCCGAACTTGGTGGTTAAACTC**TACTGC**G
GTGACGA**TACTG**TAGGGGAGGTCCTGCGGAAAAATAGCTCGACGCCAGGAT

3 117 **2.6**

Cicuta virosa chloroplast, complete genome NCBI Reference Sequence: NC_037711.1: c131571-131451 5S ribosomal RNA

TATTCTGGTGTCCTAGGCGTAGAGGAACCACACCAATCCATCCCGAACTTGGTGGTTAAACTC**TACTGC**G
GTGACGA**TACTG**TAGGGGAGGTCCTGCGGAAAAATAGCTCGACGCCAGGAT

3 117 **2.6**

Gallus gallus breed Red Jungle Fowl isolate RJF #256 unplaced genomic scaffold, GRCg6a CHRUN_242 NCBI Reference Sequence: NW_020109910.1: c2060-1941 5S ribosomal RNA

AAGCCTACAGCACCCGGTATTCCCAGGAGGTCTCCCATCCAA**GTACT**AACCAGGCCCGACCCTGCTTAGC
TTCCGAGATCAGACGAGATCGGGCGTTACCAGGGTGGGATGGCCGTAGGC

1 116 **0.9**

Cricetulus griseus unplaced genomic scaffold, CriGri_1.0 scaffold884, whole genome shotgun sequence NCBI Reference Sequence: NW_003613683.1: c1840240-1840122 5S ribosomal RNA

ATC**TACTGCCAT**ACCACCCTGAACGTGCCGGATCTCATCTGATCTCAGAAGCTAAGCAGGGTCGGGCCTG
GCTA**GTACT**TGGATGGGAGACCACCTGGGAA**TACTG**GGTGCTGTAGGCA

7 115 **6.1**

Cephus cinctus unplaced genomic scaffold, Ccin1 Ccin1_scaffold0299, whole genome shotgun sequence NCBI Reference Sequence: NW_014333002.1: 26981-27099 5S

GGCAAGGTCCATACCACGT**TGAAT**ACACCAGTTCTCGTCCGATCACTGAAGTTAAGCAACATTGGGCGCG
GTCA**GTACT**TGGATGGGTGACCGCTTGGGAACACCGCGTGTCCTTGCCT

2 115 **1.7**

Saccharomyces cerevisiae S288C chromosome XII, complete sequence NCBI Reference Sequence: NC_001144.5 :459676-459796 5S

GGTTGCG**GCCAT**ATCTACCAGAAAGCACCGTTTCCCGTCCGATCAACTGTAGTTAAGC**TGGTA**AGAGCCT
GACCGAGTAGTGTAGTGGGTGACCATACGCGAAACTCAGGTGCTGCAATCT

2 117 1.7

Glaucocystis incrassata strain SAG 229-2 plastid, complete genome NCBI Reference Sequence: NC_040152.1:c27253-27129 5S

CCTGGTGCCTATGGCGTGGTGGACCCACACCGAAACCATCCCGAACTC**GGTACT**AGAAGTGAAACACTAC
AGCGGTG**AAGAT**ACTAGAGGGGCAGCTCTCTGGGAAAATAGCTCGG**TGCCA**GGAT

4 121 **3.3**

Callitropsis vietnamensis plastid, complete genome NCBI Reference Sequence: NC_026298.1: 60600-60720 5S ribosomal RNA

AATTCTGGTGTCTCAGGCGTAGGGGAACCACACCAATCCATCCCGAACTTGGTGGTTAAA CTT**TACTGC**G
GTGACGA**TACTGC**AGGGGAAGCCCTGTGGAAAAATAGCTCGA**TGCCA**GAAT

5 117 **4.3**

Hesperocyparis glabra plastid, complete genome NCBI Reference Sequence: NC_026297.1: 60712-60832 5S ribosomal RNA

AATTCTGGTGTCTCAGGCGTAGGGGAACCACACCAATCCATCCCGAACTTGGTGGTTAAACTT**TACTGC**G
GTGACGA**TACTGC**AGGGGAAGCCCTGTGGAAAAATAGCTCGA**TGCCA**GAAT

5 117 **4.3**

Cupressus sempervirens plastid, complete genome NCBI Reference Sequence: NC_026296.1: 62043-62163 5S ribosomal RNA

AATTCTGGTGTCTCAGGCGTAGGGGAACCACACCAATCCATCCCGAACTTGGTGGTTAAACTT**TACTGC**G
GTGACGA**TACTGC**AGGGGAAGCCCTGTGGAAAAATAGCTCGA**TGCCA**GAAT

5 117 **4.3**

Callitropsis nootkatensis plastid, complete genome NCBI Reference Sequence: NC_026295.1: 60515-60635 5S ribosomal RNA

AATTCTGGTGTCTCAGGCGTAGGGGAACCACACCAATCCATCCCGAACTTGGTGGTTAAACTT**TACTGC**G
GTGACGA**TACTGC**AGGGGAAGCCCTGTGGAAAAATAGCTCGA**TGCCA**GAAT

5 117 **4.3**

Cavia porcellus strain inbred line 2N unplaced genomic scaffold, Cavpor3.0 supercont2_13, whole genome shotgun sequence NCBI Reference Sequence: NT_176406.1: c36908410-36908290 5S ribosomal RNA

GTCTACG**GCCA**TACCACCCTGAACGCGCCCGATCTCGTCTGATCTCGGAAGCTAAGCAGGGTCGGGCCTG
GTTA**GTACT**TGGATGGGAGACCGCCTGGGAATACCGGGTGCTGTAGGCTTT

2 117 **1.7**

Malus domestica cultivar Golden Delicious isolate X9273 #13 chromosome 9, ASM211411v1, whole genome shotgun sequence NCBI Reference Sequence: NC_041797.1: c9964992-9964874 5S ribosomal RNA

GAATGCGATCATACCAACTAATATGCACCGGATTCCATCAGAACTCTGAAGTTAAGGGAGTTTGGG**CAAG**
ATTA**GTACT**AGGATGGGTGACCTCCTAGGAAGTCCTCGTGTTGCATCCC

4 115 **3.5**

Antrostomus carolinensis isolate BGI_N321 unplaced genomic scaffold, ASM70074v1 scaffold4047, whole genome shotgun sequence NCBI Reference Sequence: NW_010326526.1: c34865-34747 5S ribosomal RNA

GCCTACGG**GCCAT**ACCACCCTGAGAACTCCCAATCTCATCTGATCTCGGAAGCTAAGCAGGGTTGGGCCCG
GTTA**GTACT**TGGATGGGAGACCGCCTTGGAATACCAGGTGCTGTAGGCT

2 115 **1.7**

Ornithorhynchus anatinus isolate Pmale09 chromosome 3, mOrnAna1.p.v1, whole genome shotgun sequence NCBI Reference Sequence: NC_041730.1: c53600548-53600430 5S ribosomal RNA

ATCTGCGG**GCCAT**ACCACCCTGAACGTGCCCGATCTCGTCTGATCTCGGAAGCTAAGCAGAGTTGGGCCTG
GTTA**GTACT**TGGATGGGA**GAATG**CCTGGGAATACCTGGTGCTGTAGGTT

3 115 **2.6**

Denticeps clupeoides chromosome 1, fDenClu1.1, whole genome shotgun sequence NCBI Reference Sequence: NC_041707.1: 30970836-30970954 5S ribosomal RNA

GCTTACGG**GCCAT**ACCACCCTGAGAACGCCCGATCTCGTCCGATCTCGGAAGCTAAGCAGGGTCGGGCCTG
GTTA**GTACT**TGGATGGGAGACCGCCTGGGAATACCAGGTGCTGTAAGCT

2 115 **1.7**

Carassius auratus strain Wakin unplaced genomic scaffold, ASM336829v1 scaf_tig00037271, whole genome shotgun sequence NCBI Reference Sequence: NW_020526371.1 :48919-49037 5S ribosomal RNA

GCTTACGG**GCCAT**ACCAACCTGGCTATGCCCGATCTCGTCTGATCTCGGAAGCTAAGCAGGTTTGGGCCTG
GTTA**GTACT**TGGATGGGAGACCGCCTGGGAATACCGGGTGCTGTAAGCT

2 115 **1.7**

Pteropus vampyrus isolate Shadow unplaced genomic scaffold, Pvam_2.0 Scaffold66, whole genome shotgun sequence NCBI Reference Sequence: NW_011888847.1: c8268243-8268125 5S ribosomal RNA

GTCTACA**GCCAT**GGCACCCTGAACATGCCTGATCTCATCTGATCTCAGAAGCTAAGCAGGGTCAGGCCTG
GTTACTACTTGGATGGCAGACCACGTAGGAATACCAGGTGCTGTAGGCA

1 115 **0.9**

Haloferax volcanii DS2, complete sequence NCBI Reference Sequence: NC_013967.1: 1603076-1603197 5S ribosomal RNA

TAAGGCGGCCAGAGCGGTGAGGTTCCACCCGTACCCATCCCGAACACGGAAGTTAAGCTCACCTGCGTTC
TGGTCA**GTACTG**GAGTGAGCGATCCTCTGGGAAATCCAGTTCGCCGCCCCTA

2 118 **1.7**

Halomicrobium sp. ZP60 chromosome, complete genome NCBI Reference Sequence: NZ_CP039375.1: 2602863-2602984 5S ribosomal RNA

AAAGGCGG**GCCAT**AGCGGCAGGGAAACACCCGTACCCATCCCGAACACGGACGTTAAGC**CTGCCA**GCGTTG
CGGTGA**GTACTG**GGGTGTGCGAACCCCTGGGAAAGCCGGTTCGCCGC**CTGCC**

6 118 **5.1**

Haloterrigena jeotgali strain A29 plasmid unnamed6, complete sequence NCBI Reference Sequence: NZ_CP031304.1: c666418-666297 5S ribosomal RNA

TACGGCGG**GCCAT**AGCGGCGAGGTGCCTCCCGTACCCATCCCGAACACGG**AAGAT**AAGCTCGCCTGCGTTT
CGGTCA**GTACTG**GAGTGGGCGACCCTCTGGGAAATCTGATTCGCCGCCACCA

4 118 **3.4**

Halobellus limi strain CGMCC 1.10331 chromosome, complete genome NCBI Reference Sequence: NZ_CP031311.1: 1536989-1537110 5S ribosomal RNA

TAAGGCGGCCAGAGCGGCAGGGCGACACCCGTACCCATCCCGAACACGGAAGTTAAGC**CTGCCA**GCGTGT
CAGCAA**GTACTG**GAGTGAGCGATCCTCTGGGAACACTGGCTCGCCGCCTCCC

4 118 **3.4**

Halobacterium salinarum strain 91-R6 chromosome, complete genome NCBI Reference Sequence: NZ_CP038631.1: 75841-75962 5S ribosomal RNA

TAAGGCG**GCCAT**AGCGGTGGGGTTACTCCCGTACCCATCCCGAACACGG**AAGAT**AAGCCCGCCTGCGTTC
CGGTCA**GTACTG**GAGTGCGCGAGCCTCTGGGAAATCCGGTTCGCCGCCTACT

4 118 **3.4**

Natronorubrum bangense strain JCM 10635 plasmid unnamed4, complete sequence NCBI Reference Sequence: NZ_CP031309.1: c108047-107926 5S ribosomal RNA

TACGGCGGCCACAGCGGCGGGGTTCCTCCCGTACCCATCCCGAACACGG**AAGAT**AAGC**CTGCC**TGCGTTC
CGGCGA**GTACTG**GAGTGCGCGAGCCTCTGGGAAATCCGTTTCGCCGCCTCCA

4 118 **3.4**

Halapricum salinum strain CBA1105 chromosome, complete genome NCBI Reference Sequence: NZ_CP031310.1: 1480608-1480729 5S ribosomal RNA

TAAGGCG**GCCAT**AGCGGCGGGGCAACTCCCGTACCCATCCCGAACACGG**AAGAT**AAGCCCGCCTGCGTTC
CGGTGA**GTACTG**GAGTGCGCGAGCCTCTGGGAAACTCGGTTCGCCGCCTACT

4 118 **3.4**

Candidatus Nitrosocosmicus franklandus isolate NFRAN1 genome assembly, chromosome: NFRAN NCBI Reference Sequence: NZ_LR216287.1 :c2458839-2458721 5S ribosomal RNA

GTAGCCG**GCCAT**AGCGTTAGGGCCCGACCCGGTC**CCATTC**CGAACCCGGAAGTCAAACCTAACGTCGCTG
CTGGA**TACTG**ACGTGCGAGAGCCGTTGGGAAACAGCAGTGCTGGCACCT

4 115 **3.5**

Halorubrum ezzemoulense strain Fb21 chromosome, complete genome NCBI Reference Sequence: NZ_CP034940.1: c1789778-1789657 5S ribosomal RNA

AAAGGCGGCCAGAGCGGTGGGGACACACCCGTAC**CCATTC**CGAACACGGAAGTTAAGCCCACCAGCGTAC
CGTGAA**GTACTG**GAGTGAGCGATCCTCTGGGAACCACGGGTCGCCGC**CTGCC**

5 118 **4.2**

Halorubrum sp. BOL3-1 chromosome, complete genome NCBI Reference Sequence: NZ_CP034692.1: 2178713-2178834 5S ribosomal RNA

GAAGGCGGCCAAAGCGGTGGAGACACACCCGTAC**CCATTC**CGAACACGGAAGTTAAGCCCACCAGCGTAT
CGTGAA**GTACTG**GAGTGAGCGATCCTCTGGGAACCACGAGTCGCCGCCTACC

4 118 **3.4**

Sulfodiicoccus acidiphilus HS-1 DNA, complete genome NCBI Reference Sequence: NZ_AP018553.1: c2264953-2264834 5S ribosomal RNA

CCAACCG**GCCATGGT**GAACGGGAAACACCCGGACTCATTTCGAACCCGGAAGTTAAGCCGTTCACGTTCG
GC**TGGTA**GTGGGACTCCGTGAGGACCCGCAAC**CTGCC**GGAGCCGGTATGG

4 116 **3.5**

Haloarcula marismortui ATCC 43049 strain ATCC 43049 5S ribosomal RNA, complete sequence NCBI Reference Sequence: NR_075280.1

TTAGGCGGCCACAGCGGTGGGGTTGCCTCCCGTACCCATCCCGAACACGG**AAGAT**AAGCCCACCAGCGTT
CCAGGGA**GTACTG**GAGTGCGCGAGCCTCTGGGAAATCCGGTTCGCCGCCACC

3 118 **2.6**

Acorus calamus plastid, complete genome NCBI Reference Sequence: NC_007407.1: c130993-130873 5S rRNA

TATTCTGGTGTCCTAGGCGTAGAGGAACCACACCAACCCATCCCGAACTTGGTGGTTAAACTC**TACTGC**G
GTGACGA**TACTG**TAGGGGAGGTCCTGCGGAAAAATAGCTCGACGCCAGGAT

3 117 **2.6**

Ammopiptanthus mongolicus chloroplast, complete genome NCBI Reference Sequence: NC_034742.1: c129838-129718 5S rRNA

TATTCTGGTGTCCTAGGCGTAGAGGAACCACACCAATCCATCCCGAACTTGGTGGTTAAACTC**TACTGC**G
GTGACGA**TACTG**TAGGGGAGGTCCTGCGGAAAAATAGCTCGGCGCCAGGAT

3 117 **2.6**

Ammopiptanthus nanus chloroplast, complete genome NCBI Reference Sequence: NC_034743.1: 108087-108207 5S rRNA

TATTCTGGTGTCCTAGGCGTAGAGGAACCACACCAATCCATCCCGAACTTGGTGGTTAAACTC**TACTGC**G
GTGACGA**TACTG**TAGGGGAGGTCCTGCGGAAAAATAGCTCGGCGCCAGGAT

3 117 **2.6**

Ammopiptanthus nanus chloroplast, complete genome NCBI Reference Sequence: NC_034743.1: 108087-108207 5S rRNA

TATTCTGGTGTCCTAGGCGTAGAGGAACCACACCAATCCATCCCGAACTTGGTGGTTAAACTC**TACTGC**G
GTGACGA**TACTG**TAGGGGAGGTCCTGCGGAAAAATAGCTCGGCGCCAGGAT

3 117 **2.6**

Arabis alpina complete chloroplast genome NCBI Reference Sequence: NC_023367.1: c129079-128959 5S rRNA

TATTCTGGTGTCCTAGGCGTAGAGGAACAACACCAATCCATCCCGAACTTGGTGGTTAAACTC**TACTGC**G
GTGACGA**TACTG**TAGGGGAGGTCCTGCGGAAAAATAGCTCGACGCCAGGAT

3 117 **2.6**

Methanoregula boonei 6A8 strain 6A8 5S ribosomal RNA, complete sequence NCBI Reference Sequence: NR_075507.1

GATAGCG**GCCAT**AGCTGTCGGGAAACACCTGGTCT**CATTC**CGAACCCAGCAGTTAAGCTGACACACGTAG
TATGCT**GTACT**TG**GGTAC**GCGAGTCCCTGGGAACCATACCAAGCTGCTATCA

4 118 **3.4**

Ignicoccus hospitalis KIN4/I strain KIN4/I 5S ribosomal RNA, complete sequence NCBI Reference Sequence: NR_075517.1

CCCGGCCCGGCCACAGCGACCGGGTAACACCCGGAC**CCATT**TCGAACCCGGAAGTTAAGCCGGTCGCGCG
GGGTCCGGCAGTGGCGTCCGAGAGGCGCCGCAGCCGCCCCGTGCTGGGACCGGGC

1 121 **0.8**

Thermococcus onnurineus NA1 strain NA1 5S ribosomal RNA, complete sequence NCBI Reference Sequence: NR_075529.1

GGTACGGCGGTCATAGCGGCGGGGTCACACCCGGTCTCGTTTCGACCCCGGAAGTTAAGCCCGCCAGCGA
TCCCGGGT**GTACTGCC**CTCCGAGAGGGGGCGGGAAACCGGGAACGCCGCCGGCCAC

4 122 **3.3**

Methanococcus aeolicus Nankai-3 strain Nankai-3 5S ribosomal RNA, complete sequence NCBI Reference Sequence: NR_075496.1

TAACGGTCATAGCGAAGGTGTTATATCCGATC**CCATTC**CGATCTCGGAAA**TCAAG**CCCTTCAGCGATTCC
CTAA**GTACTGCCATTC**GGTGGAAACAGGGAGACGCTGTTAGT

11 108 **10.2**

Methanococcus vannielii SB strain SB 5S ribosomal RNA, complete sequence NCBI Reference Sequence: NR_075495.1

TAACGGTCAAAGCGGAGGTGTAACATCCGATCCCATCCCGATCTCGGAAATTAAGCCCTCCAGCGATTCT
TTAA**GTACTG**CTATCTAGTGGGAACAAAGTGACGCCGTTAGTCA

2 110 **1.8**

Metallosphaera sedula DSM 5348 strain DSM 5348 5S ribosomal RNA, complete sequence NCBI Reference Sequence: NR_075478.1

TCCACCCGGCCATAGCGGGGGAGCAACACCCGGACTCATCTCGAACCCGGAAGTAAAGTCCCCTGCGTTG
GGGATGCGGTGGGATCCGAAAGGGCCCGCAGCTACCCCAAGCTGGGATGGA

1 117 **0.9**

Methanobrevibacter smithii ATCC 35061 strain ATCC 35061; PS; DSMZ 861 5S ribosomal RNA, complete sequence NCBI Reference Sequence: NR_075474.1

GTTTTGCGGTCATAGCATGGGGGTTATACCTGGTCTCGTTTCGATCCCAGTAGTGAAGTCCTTTTGTGTT
TTGTTTGT**GTACTATGGT**TTTCTATGGGAATTTCATTTCGCTGCAAG

2 113 **1.8**

Staphylothermus marinus F1 strain F1 5S ribosomal RNA, complete sequence NCBI Reference Sequence: NR_075459.1

CCCG**GCCAT**AGTGGCCGGGCAACACCCGGTCTCATATCGAACCCGGAAGTTAAGCCGGCCACGTTGGGGT
GGGCCGTGAGGTCCGAGAGGCCTCGCAGCCGCCCCAAGCTGGGAT

1 111 **0.9**

Methanoculleus marisnigri JR1 strain JR1 5S ribosomal RNA, complete sequence NCBI Reference Sequence: NR_075455.1

TAGCG**GCCAT**AGCAGAGGGGAAACACCTGGAC**CCATTC**CGAACCCAGCAGTTAAGCCCTCTCACG**TGCCA**
TGTGGTACTGAGGTGCGCGAGCCCTCGGGAAGCACGGCTCGCTGCTATTAC

9 111 **8.1**

Pyrobaculum calidifontis JCM 11548 strain JCM 11548 5S ribosomal RNA, complete sequence NCBI Reference Sequence: NR_075454.1

CCCG**GCCAT**AGGCGCCGGTGCTACACCCGGTCTCATCAGAACCCGGAAGTTAAGGCCGGCGCCGCGCTCG
GGA**GTACTG**GGCTCCGCGAGGGCCCGGGAAACCGGCGTGCTGGGAG

3 112 **2.7**

Methanocorpusculum labreanum Z strain Z 5S ribosomal RNA, complete sequence NCBI Reference Sequence: NR_075452.1

TCATGCGGCCATAGCAGAGAGGTTCCACCCGGACCCATTCCGAACCCGGCAGTTAAGCTCTCTTACGTCG
GCTATTGTACTGAGATATGCGAATTCTCGGGAAGTTGTCAACGCTGCACCCC

5 118 **4.2**

Pyrobaculum islandicum DSM 4184 strain DSM 4184 5S ribosomal RNA, complete sequence NCBI Reference Sequence: NR_075435.1

CCCGGCCATAGGCGCCGGTGCTACACCCGGACTCATCAGAACCCGGAAGTTAAGGCCGGCGCCGCGGACG
GGAGTACTGGGCTCCGCGAGGGCCCGGGAAACCGCCGTGCT

3 107 **2.8**

Rahnella aquatilis HX2 strain HX2 5S ribosomal RNA, complete sequence NCBI Reference Sequence: NR_075987.1

CCTGGCGGCAGTAGCGCGGTGGTCCCACCTGACCCCATGCCGAACTCAGAAGTGAAACGCCGTAGCGCCG
ATGGTAGTGTGGGGTCTCCCCATGCGAGAGTAGGGAACTGCCAGGC

5 112 **4.5**

Frateuria aurantia DSM 6220 strain DSM 6220 5S ribosomal RNA, complete sequence NCBI Reference Sequence: NR_075981.1

CCTGGCGGCTATAGCGCTGTGGTCCCACCCGATCCCATCCCGAACTCGGAAGTGAAACGCAGTTGCGCCG
ATGGTAGTGTAGCTTGCTATGCAAGAGTAGGTCACCGCCAGGG

3 109 **2.8**

Solitalea canadensis DSM 3403 strain DSM 3403 5S ribosomal RNA, complete sequence NCBI Reference Sequence: NR_075980.1

TCAGGTGCCTATATCGGCGGTGTCCACCTCTTCCCATTCCGAACAGAGAAGTTAAGCCCGCCAGAGCCGA
TGGTACTGCCGTAACAGGTGGGAGAGTAGGTCGGTGCCGATT

9 108 **8.3**

Flavobacterium columnare ATCC 49512 strain ATCC 49512 5S ribosomal RNA, complete sequence NCBI Reference Sequence: NR_075965.1

TAAGGTGGTTATTGCGGCGGGGCTCACCTCTTCCCATTTCGAACAGAGAAGTTAAGCCCGCCAGCGTCGA
TGGTACTGCATTTTGTGGGAGAGTAGATCGCCGCCTTTC

7 108 **6.5**

Desulfovibrio africanus str. Walvis Bay strain Walvis Bay 5S ribosomal RNA, complete sequence NCBI Reference Sequence: NR_075964.1

CCTGGCGGCCATAGAGGTGGGGCCACACCCGATCCCATTCCGAACTCGGCAGTTAAGACCACCCTCGCCG
ATGATACTTGGAGTCCACTCCTGGGAAAGTAGGTCGCCGCCAGGT

4 111 **3.6**

Sulfobacillus acidophilus DSM 10332 strain DSM 10332 5S ribosomal RNA, complete sequence NCBI Reference Sequence: NR_075961.1

GTCGGTGCGTTGCGCCGGGTGAGTTGACCCGTTCCCATGCCGAACACGGTCGTCCCCCGCCCGTACGCTG
AGACTACTGGTCCCGTCGGGGACTGGGATCATCAGGCCGTGCCGACC

1 113 **0.9**

Niastella koreensis GR20-10 strain GR20-10 5S ribosomal RNA, complete sequence NCBI Reference Sequence: NR_075960.1

T**ATGGT**GATTATACCGAGGGTGTTCACCTCTTC**CCATTC**CGAACAGAGAAGTTAAGCCCCTCATGGCCG**A**
TGGTACTACACAACAATGTGGGAGAGTAGGTAGT**TGCCAT**AT

9 108 **8.3**

Owenweeksia hongkongensis DSM 17368 strain DSM 17368 5S ribosomal RNA, complete sequence NCBI Reference Sequence: NR_075957.1

T**ATGGT**GACTATAGCGGTGGGGATCACCTCTTC**CCATTC**CGAACAGAGCAGTTAAGCCCACCAGCGCAG**A**
TGGTACTGGAGTAATATCCGGGAGAGTATGTCGT**TGCCA**CTT

9 108 **8.3**

Streptococcus parasanguinis FW213 strain FW213 5S ribosomal RNA, complete sequence NCBI Reference Sequence: NR_075949.1

TAAGTGACGATAGCCTAGGAGATACACCTGTACCCATGCCGAACACAGAAGTTAAGCCCTAGAACGCCGG
AAGTAGTTGGGGGTTGCCCCCTGTGAGATAAGGAAGTCGCTTAGC

0

Gordonia polyisoprenivorans VH2 strain VH2 5S ribosomal RNA, complete sequence NCBI Reference Sequence: NR_075948.1

CGGCGGCTATAGCGGTGGGGAAACGCCCGGTC**CCATTC**CGAACCCGGAAGCTAAGCCCACCAGCGCC**AAT**
GGTACTGCACCCTAATCAGTGTGGGAGAGTAGGACACCGCCGAACA

9 112 **8**

Paenibacillus terrae HPL-003 strain HPL-003 5S ribosomal RNA, complete sequence NCBI Reference Sequence: NR_075946.1

TTTGGTGGCGATAGCGGAGGGGTTCCACACGTACCCATCCCGAACACGACCGTTAAGCCCTCCAGCGCCG
ATGGTACTTGGACCGCAGGGTCCTGGGAGAGTAGGACGTCGCCAAGC

4 113 **3.5**

Thermovirga lienii DSM 17291 strain DSM 17291 5S ribosomal RNA, complete sequence NCBI Reference Sequence: NR_075945.1

TCTGGTGGTCATAGCGGAGGGGAAACACCCGGATCCATGCCGAACCCGGCAGTTAAGCCCTCCAGCGCCG
ATGGTACTGCGATGGGTAATCGTGGGAGAGTAGGTCGCCGCCAGAA

6 112 **5.4**

Exiguobacterium antarcticum B7 strain Eab7 5S ribosomal RNA, complete sequence NCBI Reference Sequence: NR_075943.1

TGGTGACGATAGCCAAGTGGTCACACCCGTTCCCATGCCGAACACGGAAGTTAAGCACTTGAACGCCGAA
AGTAGTTGGGGGCTTCTCCCTGTGAGGATAGGACGT**TGCCA**GGCC

1 111 **0.9**

Muricauda ruestringensis DSM 13258 strain DSM 13258 5S ribosomal RNA, complete sequence NCBI Reference Sequence: NR_075936.1

CTAGGTG**GCCAT**GGCGACGGGGCCCACCCCTTT**CCATTC**CGAACAGGGAAGTTAAGCCCGTTTGCGCCG**A**
TGGTACTGCCACACCAGGTGGGAGAGTAGGTCGCCGCCTTCT

11 108 **10.2**

Acetobacterium woodii DSM 1030 strain DSM 1030 5S ribosomal RNA, complete sequence NCBI Reference Sequence: NR_075932.1

CGTAATGATGGCGGAAGGGCCACACCTGTTCCCATACCGAACACAGAAGTAAAGTCTTCCAGCGCCGAAA
GTACTTGGTGGGTAA**CTGCC**TGGGAGGATAGGACATTGCGGGACT

2 111 **1.8**

Emticicia oligotrophica DSM 17448 strain DSM 17448 5S ribosomal RNA, complete sequence NCBI Reference Sequence: NR_075929.1

C**ATGGT**GTTAATAGGGCGGGTGTTCACCTCTTCCCATCTCGAACAGAGAAGTTAAGCCCCGCACCGCCG**A**
TGGTACTAATGTCAAAATTGGGAGAGTAGGTAGACGCCAGGA

5 108 **4.6**

Turneriella parva DSM 21527 strain DSM 21527 5S ribosomal RNA, complete sequence NCBI Reference Sequence: NR_075928.1

TCTGTGCGCAATAGAGCAAGGGTCACACCCGTTCCCATCCCGAACACGGAAGTTAAGACTTGCATCGCC**A**
ATGGTACTCGGTTCTTCGGAGCCCGGGAGAGTAGGACGCGCACAGGT

5 113 **4.4**

Cyclobacterium marinum DSM 745 strain DSM 745 5S ribosomal RNA, complete sequence NCBI Reference Sequence: NR_075927.1

TTAGGCGGCCCGGCGCAGGGGACCCACCTCTTCCCATCCCGAACAGAGAAGTTAAGCCCTGCAGCGCCG**A**
TGGTACTGGGGTTACACCTGGGAGAGTAGGTCGCCGCCTTCT

5 108 **4.6**

Spirochaeta thermophila DSM 6578 strain DSM 6578 5S ribosomal RNA, complete sequence NCBI Reference Sequence: NR_075923.1

CCTGGTGACTCGAGCGGAGGGGCAACACCCGTTC**CCATTC**CGAACACGGAAGTTAAGCCCTCCAGCGCCG
ATGGTACTGCCCCATGAGGGGTGGGAGAGTAGGCCGTCGCCAGGC

9 111 **8.1**

Runella slithyformis DSM 19594 strain DSM 19594 5S ribosomal RNA, complete sequence NCBI Reference Sequence: NR_075917.1

G**ATGGT**GGTTACAGGGCGGGTGTTCACCTCTTC**CCATTC**CGAACAGAGAAGTTAAGCCCCGCTCCGCCGA
TGA**TACTG**AGATAAAACTTGGAAAAGTAGGTAGCCGCCACAA

4 108 **3.7**

Flexistipes sinusarabici DSM 4947 strain DSM 4947 5S ribosomal RNA, complete sequence NCBI Reference Sequence: NR_075916.1

ACTGGTGGCTATGGCGGAGGTGCAACACCCGTAC**CCATTC**CGAACACGGAAGTTAAGACCTCCAGCGCCG
AAGATACTCGGGCTAACCAAGCCCCGGAAAAATAGGTCGCCGCCAGTG

3 114 **2.6**

Myxococcus fulvus HW-1 strain HW-1 5S ribosomal RNA, complete sequence NCBI Reference Sequence: NR_075913.1

TCCGGTGGCTATGTCGGAGGGGTCCCACCCGTTCCCATCCCGAACACGGAAGTTAAGCCCTCCAGAGCCG
ATGGTACTTCGCGGGAAACCGCGCGGGAGAGTAGGTCG**CTGCC**GGAT

5 113 **4.4**

Oligotropha carboxidovorans OM5 strain OM5 5S ribosomal RNA, complete sequence NCBI Reference Sequence: NR_075911.1

TGGTGGTTCTAGCGAGGAGCCTCAACCCGATC**CCATTC**CGAACTCGGCCGTTAAACTCCTCAGCGCCG**AT**
GGTACTATGGC**TCAAG**CCCTGGGAGAGTAGGTCG**CTGCCA**GGCC

9 110 **8.2**

Corynebacterium ulcerans BR-AD22 strain BR-AD22 5S ribosomal RNA, complete sequence NCBI Reference Sequence: NR_075906.1

TGGTGGTTATAGCGTCGGGGGTACGCCCGGTCCCTTTCCGAACCCGGAAGCTAAGCCCGATTGCGCTG**AT**
GGTACTGCACCTGGGAGGGTGTGGGAGAGTAGGTCGCCGCCAACCA

6 114 **5.3**

Lacinutrix sp. 5H-3-7-4 strain 5H-3-7-4 5S ribosomal RNA, complete sequence NCBI Reference Sequence: NR_075910.1

TAAGGTGATTATAGCGATAGGGCTCACCTCTTA**CCATTC**CGAACAGAGAAGTTAAGCCTATTAGCGCCG**A**
TGGTACTACATTTGTGGGAGAGTAGGTCGTCGCCTTTT

6 104 **5.8**

Treponema brennaborense DSM 12168 strain DSM 12168 5S ribosomal RNA, complete sequence NCBI Reference Sequence: NR_075894.1

CCTGGTG**GCCAT**AGTGGAGAGGTAATACCCGTTCCCATCCCGAACACGGAAG**TCAAG**CTCTCTTACGCCC**G**
ATGATACTGCTATTGCGGGAAAGTAGGTTG**CTGCCA**GGC

7 105 **6.7**

Thermodesulfobium narugense DSM 14796 strain DSM 14796 5S ribosomal RNA, complete sequence NCBI Reference Sequence: NR_075892.1

CCCGGTGACCATAGCAAAGGTGCCCCACCCGTTC**CCATTC**CGAACACGGAAGTGAAAGCCTTTAGCGCCG
ACGATACTATTGGGGAAAATAGGACGTTGCCGGGA

2 101 **2**

Porphyromonas asaccharolytica DSM 20707 strain DSM 20707 5S ribosomal RNA, complete sequence NCBI Reference Sequence: NR_075891.1

TAAGGTGGTTATAGCGTTGGGGATCCACCTCTTC**CCATTC**CGAACAGAGCAGTTAAGCCCAATAGCGCCG
ATGGTACTGAGTAAAACCGGGAGAGTAGGTAACCGCCTTTC

7 107 **6.5**

Thermodesulfatator indicus DSM 15286 strain DSM 15286 5S ribosomal RNA, complete sequence NCBI Reference Sequence: NR_075890.1

CCGGGTGCCCATACCGGAGGGGCCACACCCGTTC**CCATTC**CGAACACGGTCGTTAAGCCCTCCAGGGCCG
ATGGTACTGCTGGGTTACCCCGGTGGGAGAGTAGGTCGGCGCCCGGG

8 113 **7.1**

Sphaerochaeta coccoides DSM 17374 strain DSM 17374 5S ribosomal RNA, complete sequence NCBI Reference Sequence: NR_075885.1

CCCGGTGGTCATAGCAGAGTGGTCATACCCGTTCCCATCCCGAACACGGCAG**TCAAG**CACTCTCGCGCCG
ATGGTACTGCGTTTTTCGCGGGAGAGTAGGTCGCCGCCGGGC

7 108 **6.5**

Verrucosispora maris AB-18-032 strain AB-18-032 5S ribosomal RNA, complete sequence NCBI Reference Sequence: NR_075884.1

TACGGCGGTTATGGCGGAAGGGAAACGCCCGGTTA**CATTC**CGAACCCGGAAGCTAAGCCTTCCAGCGCCG
ATGGTACTGCACTCGGGAGGGTGTGGGAGAGTAGGACACCGCCGGAC

7 113 **6.2**

Selenomonas sputigena ATCC 35185 strain ATCC 35185 5S ribosomal RNA, complete sequence NCBI Reference Sequence: NR_075883.1

TCCAGTGGCGATAGCTGAGTGGTTCCACCTGTTCCCATCCCGAACACAGTCGTTAAGCACTCATACGTCG
AAA**GTACT**TGGTTGGAGACGACCTGGGAGGATAGAAAGCTGCTGGTT

1 113 **8.9**

Observed AL pentamer frequency: 401.2 /100 ≈ 4%
Expected: $22/1024 \pm 1.65x(22x1002/1024^2x100)^{1/2} \approx 2.15\% \pm 0.25\%$*

$4\% \approx 2.15\% + 12\ \sigma$, where $\sigma \approx 0.15\%$

$P(\text{Observed} \geq 4\%) \leq \exp(-144) \approx (e^{-1.44})^{100} \approx 3\ 10^{-63}$

AL-pentamer proximity for 100 SASP proteins

Clostridioides difficile strain 630 delta erm, complete genome GenBank: CP016318.1: 1503870-1504070 SASP

ATGGATGATGTTTCAAGACAAAATGCTATAAAAGCTTTAAAACAAACAAAAATGGAAATAGCAGGAGAGT
ATGGAATGAATTATGAAGATGCTTTTGAAATAATAGAAAATGCGTCTAATAAAGGTGTTTTAGAAGGTTA
CTTTAAAAAGCTTGAAAAGAAAAAAAATTTAGGTCAAGGTATATCTAGGCATTTAGAATAA

13 197 **6.6**

Pseudoflavonifractor capillosus ATCC 29799 B_capillosus-2.0.1_Cont39, whole genome shotgun sequence GenBank: AAXG02000007.1: c199825-199622 SASP

TGGAGGCCGCTTCCGAGGTTGGCGTCAACCTGAAGCAGGGCTACAACGGCGACCTGACCTCCCGTGAGGC
CGGCTCCGTGGGCGGCCAGATGGTCAAGAAGATGATCCAGGCCTACGAGAACGGAATGCAGTAA

8 130 **6.2**

Eubacterium ventriosum ATCC 27560 E_ventriosum-MSIQ_Cont10.2, whole genome shotgun sequence GenBank: AAVL02000028.1: 64678-64878 Small, acid-soluble spore protein, alpha/beta type SASP

ATGAGTAACACTAACAAAGTTAATGTTCCTGAAGCAAAGGGTGCTATGAACAGATTCAAAATGGAATGTG
CATCAGAGGTAGGCGTTCAGCTTACAGATGGTTATAACGGAAACTTAACATCAGCCCAGGCAGGTTCTGT
GGGTGGACAGATGGTTAAGAAGATGATTGAAGCTCAGGAAAGACAGATGAGCGGTAAATAA

14 197 **8.2**

Dorea longicatena DSM 13814 D_longicatena-MSIQ_Cont14.1, whole genome shotgun sequence GenBank: AAXB02000016.1: 61524-61730 Small, acid-soluble spore protein, alpha/beta type SASP

ATGGCAAATCGTTCATCAAACAAAGCAGCCGTACCTGAGGCAAAGGGTGCATTAGACAAATTCAAGTATG
AGGTAGCAAGCGAACTTGGAGTTCCGTTATCAGACGGCTACAACGGAGACCTGACATCAAGACAAAACGG
ATCTGTCGGCGGTTATATGGTTAAGAAAATGATTGAACAGCAGGAAAAACAGATGGCAGGCAAATAG

7 203 **3.5**

Intestinibacter bartlettii DSM 16795 C_bartlettii-2.0.1_Cont622, whole genome shotgun sequence GenBank: ABEZ02000022.1: 280387-280689 Small, acid-soluble spore protein, beta type SASP

TTATTGTTGTCCTTTACCTGCCATTTGTTGTTCTGCCATTTCGACTAATTTTTTAGTCATGTATCCACCA
ACATAACCATTTTGTCTAGCTGTTAAGTTTCCTTTATCAACTTGTTGATAATCAGCCATACCTAGTTCAT
TAGCTATTTCTAATTTCATTTGATTTAATGCTTGTTTAGCTTGTGGAACTGCTGTTTTGTTATTGTTGTT
GTTTGACATATCTAATACCTCTTTTCTATGATTTTTGTTTCTTGGTAGTATTATATGAAATATTTGTTTT
ATAATACATACAATTTGTGACAA

13 299 **4.4**

[Eubacterium] siraeum DSM 15702 E_siraeum-3.0.1_Cont322, whole genome shotgun sequence GenBank: ABCA03000045.1: c37855-37667 Small, acid-soluble spore protein, alpha/beta type SASP

ATGGCTAGTACCAATAAAAAGAACAAGGCAGCATTAAACAGCATCAAGATGCAGGCTGCAAGCGAAGTAG
GCGTACCCCTTAAGGATAACGGCTACAACGGCGACCTTACTTCAAAGCAGGCTGGTTCAATCGGCGGTCA
GATGGTCAAGAAGATGATCGAATCCTACGAAATGTCAAACAAGCAGTAA

13 185 **7**

[Eubacterium] hallii DSM 3353 E_hallii-1.0_Cont439.1, whole genome shotgun sequence GenBank: ACEP01000143.1: 6905-7126 Small, acid-soluble spore protein, alpha/beta type SASP

ATGGCAAGTAATAAAAATTCTTCATCTAACAGAGTAGAAGTACCGGAAGCAAGAGACGCAATGGACCGTT
TTAAAATGGAAGTAGCCAACGAACTTGGTGTGAATTTAAAACAGGGATATAACGGAGATATTACTGCAAG
AGATGCCGGCTCCATCGGAGGAGAAATGGTTCGTAAGATGATCAAGAGACAGGAAGAGGAAATGGCTGGT
CAGAGCCGCTAA

16 218 **7.3**

[Clostridium] scindens ATCC 35704 C_scindens-2.0.1_Cont260, whole genome shotgun sequence GenBank: ABFY02000013.1: 132098-132310 Small, acid-soluble spore protein, alpha/beta type SASP

ATGAC**AATGG**CAAGTCGTTCATCAAACAGAGCAGCCGTACCTGAAGCAAAGGGTGCATTGGACAA**ATTCA**
AAATATGAGGTTGCAAACGAATTAGGAGTACCGTTAACAGACGGATACAACGGAGACCTGACATCTAGACA
GAATGGTTCTGTCGGCGGATAT**ATGGT**TAAGAAAATGATCGAACAG**CAAGA**GAAAC**AGATG**GCTGGCAAATAA

9 199 **4.5**

Coprococcus eutactus ATCC 27759 C_eutactus-2.0.1_Cont262, whole genome shotgun sequence GenBank: ABEY02000018.1: c65614-65408 Small, acid-soluble spore protein, alpha/beta type SASP

ATGGCAAG**TTCAA**ACTCTAACAAGGCAAGTGTCCCAGAGGCAAAGAGTGCT**ATGAA**CAAG**TTCAAGATG**G
AGGTAGCTAAGGAGATCGGTGTAGATCTTAAGGACGGCTAC**AATGG**AGATTTAACTTCAGCTCAGTCTGG
ATCGATCGGAGGAG**AGATGGT**TCGTC**AGATGATCAAGA**GACAGGAAGAGC**AGATG**TCAGGCAGATAA

15 203 **7.4**

Blautia obeum ATCC 29174 R_obeum-MSIQ_Cont262, whole genome shotgun sequence GenBank: AAVO02000003.1: 172483-172710 Small, acid-soluble spore protein, alpha/beta type SASP

ATGACAAAAAACAGTTCTTCTAACAGAGCAGTTGTACCTGAAGCCAAAGGAGCCCTGGATCGTTTTAAAT
TCGAAGTTGCAA**ATGAA**CTGGGCGTTCCGCTTACCGATGGCTACAACGGAAACCTGACATCCAAACAGAA
CGGAAGTGTCGGCGGATAT**ATGGT**CAAAAAAATGATTGAACAGCAGGAAAGAC**AGATG**TCCGGCAATTCT
CA**GAATGAA**CA**GTACTG**A

7 224 **3.1**

Blautia hydrogenotrophica DSM 10507 R_hydrogenotrophicus-1.0_Cont1.23, whole genome shotgun sequence GenBank: ACBZ01000017.1: 52449-52661 Small, acid-soluble spore protein, alpha/beta type SASP

ATGGCTAGAAATTC**TTCAA**ATTCCGCAGCAGTGCCGGAAGCAAAGAGCGCATTAGACCGTTTTAAATTTG
AGGTTGCAAACGAGTTGGGAGTACCTCTGACTGACGGTTATAACGGAAACCTGACTTCCAAACAGAACGG
AAGCGTAGGCGGTTAT**ATGGT**TAAG**AAGATGA**TTGAAGCCCAGGAAAGAC**AGATGA**CTGGCGGACA**GTACT**AA

8 209 **3.8**

[Ruminococcus] torques ATCC 27756 Ruminococcus_torques_2.0_Cont64, whole genome shotgun sequence GenBank: AAVP02000003.1: 123659-123898 Small, acid-soluble spore protein, alpha/beta type SASP

ATGTCGTTAAAATGCAAAAGGAGGAAATTGATCATGGCAAATCGTTCATCTAACAGAGCAGCAGTACCGG
AGGCAAAGGGCGCTCTGGACAA**ATTCAAG**T**ATGAA**GTGGCAAGTGAGTTAGGAGTACCGTTGACAGACGG
TTATAACGGAGACCTCACATCGAAACAGAACGGATCTGTTGGCGGATAT**ATGGTCAAGA**AAATGATCGAG
CAGCAGGAAAAAC**AGATG**GCAGGCAAATAA

8 236 **3.4**

Flavonifractor plautii ATCC 29863 F_plautiiATCC29863-1.0_Cont510.3, whole genome shotgun sequence GenBank: AGCK01000277.1: 763-972 small, acid-soluble spore protein C2 SASP

ATGTCTAGCTCTAACAATTCCAATTCCAACCAGCCCGTGGTTCCCAGCGCCCGTGAGGCCCTGAACAAG**T**
TCAAGATGGAGGCCGCCCAGGAGGTTGGCGTCAACCTGAAGCAGGGCTAC**AATGG**CGATCTGACCAGCCG
TGAGGCCGGTTCCGTTGGCGGCC**AGATGGTCAAGAAGATGA**TCCAGGCGTACGAGAACAGCATGCAGTAA

13 207 **6.3**

Desulfitobacterium hafniense DP7 D_hafnienseDSM13498-1.0_Cont144.1, whole genome shotgun sequence GenBank: AFZX01000039.1: c7693-7475 Small, acid-soluble spore protein, alpha/beta type SASP

ATGAGCAATAAAACGCC**AATGGT**TCC**TGAAT**CCGAAAACAAGTTGGATCAACTTA**AATGG**GAAGTTGCTG
AGGAACT**CCATT**TAGACGACGACATCCAGGAAAGAGGTTTTGAAAACATGACCACCCGGGAAGTCGGACA
AATCGGCGGCAAT**ATGGT**CAAAAA**AATGGT**GGCATTTGCCGAAGAGC**AGATG**GGTAAAGGGGCCGACATT
AAGGATTAA

9 215 **4.2**

Clostridium celatum DSM 1785 C_celatum-1.0_Cont521.1, whole genome shotgun sequence GenBank: AMEZ01000133.1: c61494-61312 Small, acid-soluble spore protein, alpha/beta type SASP

ATGTCAAAAGCATTAGTTCCAGAAGCAAAACAAGGATTAAGCG**CATTCAAGA**ACGAAGTAGCAGCGGA**AA**
TGGGAGTA**CCATTCA**CAGATTAC**AATGGTA**ACCTAACATCTAGACAATGCGGTTCCGTAGGTGGTGA**AAT**
GGTTAAGA**GAATGGTA**GAACAATACGAAAGTGGATTAAAGTAA

18 179 **10**

Marvinbryantia formatexigens DSM 14469 B_formatexigens-1.0.1_Cont7.1, whole genome shotgun sequence GenBank: ACCL02000008.1: 188424-188837 Small, acid-soluble spore protein, alpha/beta type SASP

TTTACATTA**ACTGCCA**GTTTATATTCCGGAGAATTTCATATACTATC**ACTGC**AATCGAAAAACACACAAA
AAAACAAACGAA**AAGAT**TGCAGATAAAATGCAAAGGAGGCGTAAATTTTATG**TCAAG**TAAATCTTCTAAC
ACAGCAGCAGTACCGGAGGCGAAGGGAGCACTGGACCGT**TTCAA**ATTTGAAGTTGCGAGCGAGCTCGGCG

6 206 **2.9**

[Bacteroides] pectinophilus ATCC 43243 B_pectinophilus-1.0.1_Cont749, whole genome shotgun sequence GenBank: ABVQ01000035.1: c417970-417746 Small, acid-soluble spore protein, alpha/beta type SASP

ATGGAGGTGGACAGT**ATGAA**CAG**TTCAA**ATTCTAATTCTAAGACAAAGAAACAGGCACAGGATGCTCTTG
ACAGATTT**AAGATG**GAAGTTGCATCAGAGATTGGTGTACCTCTTAAGGAAGGATACAACGGAGATCTTAC
AACAAGCCAGGCCGGTTCAGTCGGTGGTG**AGATGGTA**AGA**AAGATGA**TTAAGAGACAGGAAGAGC**AGATG**
TCTTCAGGCAACTAG

11 221 **5**

Ruminococcus lactaris ATCC 29176 R_lactaris-2.0.1_Cont378, whole genome shotgun sequence GenBank: ABOU02000017.1: 17538-17744 Small, acid-soluble spore protein, alpha/beta type SASP

ATGGCAAGTCGTTCATCTAACAAAGCAGCAGTGCCTGAAGCAAAAGGCGCACTCGACAAG**TTCAAG**TACG
AGGTAGCAACAGAGCTGGGAGTACCGTTGACAGACGGATACAACGGCGACCTGACATCCAAACAGAACGG
ATCTGTCGGTGGATAT**ATGGTA**AAGAAAATGATCGAGCAGCAGGAAAAGC**AGATG**GCAGGAAAATAA

5 203 **2.5**

Coprococcus comes ATCC 27758 C_comes-1.0.1_Cont2253, whole genome shotgun sequence GenBank: ABVR01000041.1: 516733-516945 Small, acid-soluble spore protein, alpha/beta type SASP

ATGAATATGGCAAGTCGTTCATCAAACAGAGCAGCCG**TGCCA**GAAGCAAAAGGTGCACTGGACAAATTTA
AATACGAGGTTGCAAGCGAACTGGGAGTACCGTTATCAGACG**GGTAC**AACGGAGACTTAAC**TTCAAGA**CA
GAACGGATCTGTCGGAGGTTAT**ATGGTCAAGA**AAATGATCGAAGAACAGGAAAAAC**AGATG**TCCGGTCGATAG

11 209 **5.3**

Clostridium sporogenes ATCC 15579 C_sporogenes-2.0.1_Cont478, whole genome shotgun sequence GenBank: ABKW02000003.1: 1295759-1295986 Small, acid-soluble spore protein, alpha/beta type SASP

ATGAAAAATAAACC**AGATGA**TAGAAGAGATAATGTAGATAAA**ATTCAA**TATAATATTACTAAGACT**ATTC**
AAAACTGTGAGCTTGC**AGATGAA**ATGATTGCAAAAAC**AGATGATGAA**AAAACGAAAAAAACTCTAATAGA
AAAAAACCAAAGAAGAAGAGAAGCACTTG**ATGGTA**TGAGAGAAGAAATTA**AAGATGAA**G**CAAGA**GATAAG
AAA**AATGG**ATATATGT

22 222 **10**

Clostridium sp. M62/1 C_sp_M621-1.0.1_Cont5.1, whole genome shotgun sequence GenBank: ACFX02000031.1: 96192-96377 Small, acid-soluble spore protein, alpha/beta type SASP

ATGAATGTACCAGAGGCTAAGGAAGCA**ATGAA**CAG**ATTCAAGATG**GAGGT**TGCCA**ATGAGCTTGGTGTTC
CGTTAAACAGCAACGGATACAACGGCAACTTAACTTCTGCTCAGAACGGTTCTGTAGGCGGATAT**ATGGT**
TAAG**AAGATGA**TCGAGGCTCAGGAAAGAC**AGATG**GCCGGCAAATAA

16 182 **8.8**

Anaerofustis stercorihominis DSM 17244 A_stercorihominis-2.0.1_Cont258, whole genome shotgun sequence GenBank: ABIL02000005.1: 428678-428869 Small, acid-soluble spore protein, alpha/beta type SASP

TTATTTCAT**TGAAT**TTTCGT**AAGATTCAACCATTC**TTTTAA**CCATT**TCACCACCGATAGAACCGGCTTCT
TTACTAGTTAAATTA**CCATT**ATATCCATCTTG**AAGAT**TAATGCTAAGTTCGCTAGCAACTTGTTTCTTAA
ATTGGTCTAAGCCTTTTTTAGCTTCTCTGTTGTTCATCTGTTTCACCTCCAT

9 188 **4.8**

Clostridium sp. SS2/1 Clostridium_sp_SS21-1.0.1_Cont14.1, whole genome shotgun sequence GenBank: ABGC03000004.1: c56363-56142 Small, acid-soluble spore protein, alpha/beta type SASP

ATGGAAGAACGAACTTCAG**CAAGA**AGCAAAGGAACAAACAGAGTCGA**GGTAC**CAGAAGCGAGAGGAGCCT
TAGACAGATTTAAGTTCGAAGTTGCAAATGAGATCGGTGTGGATTTAAAAGAAGGATATAACGGACACTT
AACA**ACTGC**TCAGGCAGGATCTGTCGGAGGAG**AGATGGT**TCGTAAAATGATCCGCAGACAGGAAGAAGAA
ATGTCTCGCTAA

5 218 **2.3**

Anaerotruncus colihominis DSM 17241 A_colihominis-0.2.1_Cont303, whole genome shotgun sequence GenBank: ABGD02000027.1: 56025-56219 Small, acid-soluble spore protein, alpha/beta type SASP

ATGACCAGTTCCAACAATCCTGTTGTCCCGCAGGCCCGCGAGGCTCTTGATAAG**TTCAAAATGG**AAGCCG
CCCGTGAAGTCGGCGTCAACC**TCAAG**CAGGGCTACAACGGCGATCTGACCTCTCGTGAAGCCGGTTCTGT
CGGCGGCC**AGATGGTCAAGAAGATGA**TCGAGGCGTACGAGAACTCC**ATGAA**GTAA

11 191 **5.8**

Anaerostipes caccae DSM 14662 A_caccae-4.0.1_Cont13, whole genome shotgun sequence GenBank: ABAX03000002.1: c118067-117843 Small, acid-soluble spore protein, alpha/beta type SASP

ATGGAAGAAC**GTACT**TCAGCAAACAGCAAAGGAAACAACAGAGTGGAAGTTCCAGAGGCTAGGGAAGCAC
TGGACAG**ATTCAA**ATTCGAGGTTGCTA**ATGAA**ATCGGTGTAGACTTAAAAGAAGGATACAACGGACATTT
AACA**ACTGC**TCAGGCAGGTTCTGTTGGAGGAGA**AATGGT**TCGTAAAATGATCCGCAGACAGGAAGAGGAA
ATGTCTGGCCGTTAG

7 221 **3.2**

Pseudobacteroides cellulosolvens ATCC 35603 = DSM 2933 ctg1, whole genome shotgun sequence GenBank: LGTC01000001.1: 1240722-1240946 Small, acid-soluble spore protein, alpha/beta type SASP

TTACTCAGAAATCATACTTT**CTGCCATTTGAATCATTC**TCTTTACCATCTGACCGCCTATAGGGCCGCCT
TCCAAACCGTTTACTTTGGCCGGAACATCTCCCTTGTAGTGGTCATTGTTTTCCTTTATATATTGAAGGT
GTCCAATTTCTTGTGCACACTCTATTT**TGAAT**CTTGTAAGTGCTTCTCTGCTTTCAGGTACCAGTGGTTT
TCTTGGATTTGACAT

7 221 **3.2**

Bacillus mycoides strain AH603 chromosome, whole genome shotgun sequence GenBank: CM000737.1: 444037-444333 Small, acid-soluble spore protein gamma-type SASP

GTGGAGGTGAGA**AAGATG**AGTAAAAAACAACAAGGTTATAATAAGGCAACTTCTGGTGCTAG**CATTCAA**A
GTACAAATGCTAGCT**ATGGTAC**AGAGTTTGCAACTGAAAC**GAATG**TACAAGCAGTAAAACAAGCGAACGC
ACAATCAGAGGCACAGAAAGCACAAGCTTCTGCTGCTCAAAGTGCAAATGCTAGTT**ATGGTAC**AGAGTTT
GCAACTGAAAC**AGATG**TGCATGCAGTGAAAAAACAAAATGCACAATCAGCTGCAAAACAATCACAATCTT
CTAG**TTCAA**ACGAGTAA

14 293 **4.8**

Bacillus thuringiensis serovar kurstaki str. T03a001 chromosome, whole genome shotgun sequence GenBank: CM000751.1: c349955-349659 Small, acid-soluble spore protein gamma-type SASP

GTGGAGGTGAGA**AAGATG**AGTAAAAAACAACAAGGTTATAACAAAGCAACTTCTGGTGCTAG**CATTCAA**A
GTACAAATGCTAGTT**ATGGTAC**AGAGTT**TTCAA**CTGAAAC**AGATG**TACAAGCTGTAAAACAAGCAAACGC
ACAATCAGAAGCAAAGAAAGCACAAGCTTCTGGTGCACAAAGTGCAAACGCTAGTT**ATGGTAC**AGAATTT
GCAACTGAAACAGACGTG**CATTC**TGTGAAAAAACAAAATGCTAAGTCAGCTGCAAAACAATCACAATCTT
CTAGCTCAAATCAGTAA

14 293 **4.8**

Clostridium botulinum A str. ATCC 3502 complete genome GenBank: AM412317.1: 1907279-1907482 small, acid-soluble protein alpha SASP

A**TGCCA**AGTAATAAAAATTCTAATAATTTAGTAG**TGCCA**GAAGCACAACAAGGATTAAACCAACTAAA**AA**
TGGAAGTTGCAAACGAAGTAGGAATAGCAAACTATG**ATTCAATGG**ATAAAGGAAACTTAAC**TTCAAGA**CA
A**AATGG**ATATGTAGGCGGAAAC**ATGGT**TAAA**AAGATGGTA**GAAGCTTACGAAAGAAATCTATAA

15 200 **7.5**

Clostridium botulinum A str. ATCC 3502 complete genome GenBank: AM412317.1: 1907543-1907743 small, acid-soluble spore protein beta SASP

ATGGCAAATAGAAATTCTAATAATTTAGTAG**TGCCA**GAGGCAAAACATGGATTAAATCAATTAAA**AATGG**
AAGTTGCAAACGAAGTAGGAATAGCAAACTATGATGCTGTGGATAAAGGAAATTTAAC**TTCAAGA**CAAAA
CGGATATGTAGGCGGAAAT**ATGGT**TAGA**AAGATGGTA**GAAGCTTACGAAAGAAATCTATAA

10 197 **5.1**

Clostridium saccharoperbutylacetonicum N1-4(HMT), complete genome GenBank: CP004121.1: 3458976-3459185 small, acid-soluble spore proteins, alpha/beta type SASP

ATGTC**TTCAA**ACAATAGTGGAAGAAACAGAACATTAGTACCAGAAGCAAAGCAGGGATTAAGCAGATTAA
AAACTGAGGTTGCTTCAGAAGTTGGTTTAAGTGACT**ATGAA**AA**TCAAGAT**AAAGGTAACCTTTC**TTCAAG**
ACAA**AATGGTA**GCGTTGGCGGTTAC**ATGGTA**AAACACATGATCGAAAGCTACGAACAAGGCTTAAAGTAA

13 206 **6.3**

Bacillus subtilis subsp. subtilis strain SRCM101441 chromosome, complete genome GenBank: CP021507.1: c3351111-3350971 Small, acid-soluble spore protein J SASP

ATGGGTTTCTTTAATA**AAGAT**AAAGGAAAACGTTCCGAAAAAGAAAAAAACGTAATCCAAGGAGCTCTTG
AAGATGCTGGTTCAGCTCTAA**AAGATGA**TCCGC**TTCAAGA**AGCTGTGCAAAAAAAGAAAAATAATCGATAA

9 137 **6.6**

Paenibacillus sp. JDR-2, complete genome GenBank: CP001656.1: 1350163-1350387 SASP

ATGGCTAACCAATCCAACAATTTAGTTGTACCGCAAGCGAAAGAAGCTTTGAACCGC**ATGAA**ATACGAAA
TTGC**TACTG**AACACGG**CATTC**CGCTCTCCGAAGGTTATAACGGCAATCT**GATGA**CTCGCGAAGCGGGTAC
GATTGGCGGCAACATTACGAGACGCCTTG**TTCAAATGG**CAGAACAATCTTTAAGCGGAGCACAATTCTCC
AATA**CAAGAAGAT**AG

8 221 **3.6**

Paenibacillus polymyxa M1 main chromosome, complete genome GenBank: HE577054.1: 1395028-1395207 SASP H

ATGAACGTGGAACGAGCAAAA**GCCATT**TATG**ATTCA**TCCGAAACGATTGCCGTCCAATTGGATGGCAAAC
CGATTTGGATTGAGCATGT**AGATGAA**GC**GAATGGTA**TGGCAAC**GGTAC**AGATCGGCAGCAGACCGGGTGA
TACACAAACCGTTCGTGTAGATCGATTGGAAGAACAATAG

12 176 **6.8**

Butyrate-producing bacterium L2-50 GenBank: AY357288.1: 3119-3301 small acid-soluble spore protein (SASP) gene

ATGAGTGTTCCGGAAGCTAAGGACGCT**ATGAA**CAAG**TTCAA****AATGG**AGGTTGCTAAGGAAATTGGTGTAA
ACC**TCAAG**G**ATGGT**TAC**AATGGT**GACTTAACATCTGCTCAGAGCGGATCAATCGGTGGCG**AGATGG****T**TCG
TC**AGATGA****TCAAGA**GACAGGAAGAAC**AGATG**TCAGGCAGATAA

14 179 **7.8**

Bacillus cereus Q1, complete genome : CP000227.1: c2872211-2871999 SASP C2

ATGGCAAACAATAATAGTGGAAGTCGTA**ATGAAT**TGTTAGTTCGAGGTGCTGAACAAGCTCTTGATCAA**A**
TGAAAT**ATGAA**ATTGCA**CAAGA**GTTTGGTGTACAACTTGGTGCGGATACAACAGCTCG**TTCAA**ACGGATC
TGTTGGTGGTGAAATTACAAAACGTTTAGTAGC**AATGG**CTGAACAACAACTTGG**TGGTA**GAGCGAACCGTTAA

8 209 **3.8**

Oceanobacillus iheyensis HTE831 DNA, complete genome GenBank: BA000028.3: 1185411-1185635 SASP

GTGCAGACAATAGTCTTACAGGAGGTGATATT**AATGG**CTAACAACAG**TTCAA**ATCAATTAGTAGTTCCAG
GAGTACAACAAGCTCTAGATCAA**ATGAA**ATATGAGATCGC**TCAAGA**ATTTGGTGTGTCTTTAGGTGCTGA
TACAACTTCTCGTGCAAACGGTTCTGTAGGTGGAGAGATTACTAAACGTCTTG**TTCAA****ACTGC**TCAACAA
CAATTGCATGGCTAA

7 221 **3.2**

Clostridium saccharoperbutylacetonicum N1-4(HMT), complete genome GenBank: CP004121.1: 3865590-3865796 SASP

TTAGAGTCCTTGTTCGTAACT**TTCAA**T**CATTC**TTTTAA**CCATT**TCTCCACCAACACTT**CCATT**TTGTCTT
GAAGAAAGGTTTCCTTTATCAGTATTTTCATAATTGCTTAATCCTATTTCTGAAGCAACCTCAGTT**TTCA**
ATCTGTTTAAAC**CTGCC**TTTGCCTC**TGGTACT**AAAGTTCTATTGCTTCCACTATTACTTGA**TGCCAT**

11 206 **5.3**

Gottschalkia acidurici 9a chromosome, complete genome GenBank: CP003326.1: c1744362-1744150 SASP

A**TGCCA**AGTACAAACAGTAGTAATAAAATAGTTGTTCCTGAAGCTCG**TCAAG**CTTTAAATCAA**ATGAA**AA
CTGAGATTGCAAGTGAGTTAGGATTAAGTAACTATGAGTCAATAGACAAGGGAAACCTATCTGCTAGACA
A**AATGGT**TACGTTGGTGGATACATGACAAAAAGACTAGTAGAGCAAGCTCA**AAGAT**CATTATCTGGAAAATAG
6 209 **2.9**

Anoxybacillus flavithermus TNO-09.006 genomic scaffold chrAF6, whole genome shotgun sequence GenBank: KB205935.1: 356441-356587 SASP

ATGAAACCAAACAAAACAGCAGCAGGAACAAA**CATTCAA**CATGTAAAACAACAAAACGCTGGCCAGTATG
GGACAGAGTTTGCGACTGAAACTGATG**TTCAA**CACGTGAAGCAACAAAATGCAAAATCAGAAGCAAAGAA
AAGCTAA

4 143 **2.8**

Parageobacillus thermoglucosidasius TG4 DNA, scaffold01, whole genome shotgun sequence GenBank: BHZK01000001.1: 2683000-2683209 SASP

ATGG**CAAGAT****CAAG**CAATAA**ACTGC**TTGTTCCGGGAATCGAACAGGCATTGGAACAAATAAAAT**ATGAA**A
TCGCC**CAAGA**ATTTGGCGTACAATTAGGAGCAAATACCGTTTCCCGGGCAAACGGCTCTGTCGGCGGCGA
AATTACGAAACGGCTCGTTTCCCAAGCGCAAAGCCAGCTGGCCGGATCTGCTT**TTCAA**AAAACAGAATAA

7 206 **3.4**

Bacillus megaterium DNA, transposon TnMERI1, complete sequence, strain: MB1 GenBank: LC152290.1: c684-519 SASP

AT**ATGGT**GCTCAACAAGCAATTGACCAA**ATGAA**AT**ATGAA**ATTGCTAGTGAGTTTGGTGTTAACCTAGGT
CCTGATGCA**ACTGC**TCGTGCAAACGGATCTGTAGGTGGAGAGATTACAAAACGTC**TGGTAC**A**AATGG**CTG
AGCAACAACTTGGCGGCGGACGCTTC

7 162 **4.3**

Thermoactinomyces sp. CDF CotJC protein gene, partial cds; and CotJB protein, CotJA protein, alpha/beta-type SASP, GenBank: GQ202245.1: 1287-1499

ATGGCACAA**CAAGAT**CGCACTCGCAGTTCTAGTCAATTGCTGGTGGCTGGTGCTGC**TCAAG**CAATCGATC
AA**ATGAA**ATATGAGATTGC**TCAAGA**GTTTGGCG**TTCAA**TTGGGAGCTGACACCACTTCCCGCGCAAACGG
CTCTGTCGGGGGAGAAATCACGAAACGTCTGGTTTCCATGGCTCAACAACAGCTTGGAGGAAGCAACTTCTAA

7 209 **3.4**

B.megaterium SASP B gene encoding small, acid-soluble spore protein B GenBank: M14161.1

AGCTAGTCGTATGGAGGAACCAAAAGAC**ATGGTA****GATG**CAGTTATGTTCTTAGTGTCCGA**TCAAG**CAAAT
ATGATTTGCGGTCAAACGCTTATTATTGATGGAGGGCGTTCACTTTTAATGTAAGGTAAATGTATTTTGG
GCTTGCGCCTTAAAA**TACTG**AAAAAAGTTTGCATACCCTATTTACCTCCCGGGCATATTAATTTTCGTGG
AGGTGATTAC**AATGG**CAAAGCAAACAAACAAAACAGCATC**TGGTAC**AAGCACACAACACGTGAAACAACA
AAATGC**TCAAG**CATCTAAAAACAACTTC**GGTACTG****AAT**T**TGGTA**GCGAAACAAATGTA**CAAGA**AGTGAAA
CAACAAAATGCACAAGCAGCGGCAAACAAAAGCCAAAATGC**TCAAG**CATCTAAAAACAACTTC**GGTACTG**
AATTTGCTAGCGAAACAAGTGC**TCAAGA**AGTGAGACAACAAAACGCACAAGC**TCAAG**CAAAGAAAAACCA
AAACTC**TGGTA**AATACCAAGGTTAATATCAAAAGCGAACTTAACGTCTTTCATACGGCGATGTTAAGTTC
GCTTTTTCTTATATAAAAAGCAACAAAGTATGACAAAACTTCTTGAAACTTTACACAACTCGTCAAAGGAATCT

24 630 **3.8**

B.megaterium small, acid-soluble spore protein (SASP) C-1 gene GenBank: M10919.1

ATATCC**ATGAA**ATTTGCAGAGGTTTACAATCAGGTGAAATCAGCG**TGAATG**ATTTGAAAGAAAAAGACCC
TTTTGTTGTAAATGCAG**TTCAAGA**AGC**AATGG**ATAGAATAACTAAACACAATTCTTAAAATAGCTATAAA
AAGCAATCATTTTTATTAAGCCAAAGACTAACTCTTTACAGAGTTAGTCCTTTGT**CATTCAA**ATAGTATA
CC**ATGAA**CGCGAAAAAA**GAATG**ACAAACGAGTTGT**CATTC**TCTCTCCTACACATGGCAAGCGATTAGTTT
TTAGTCACAAGGAA**ATGAA**ACATCTAATCCCACTTTTTACAGTGTCGGTTGTTTCTAAAAATTAGTATGT
CATCCTAACAAGGAAATATACAT**CCATT**ATTTACCACGAATACATTTCTTCCACAATAGGAAACTTAAGA
AAAACAAGGAGGTGACACAACATGGCAAACAACAACAGCAGCAACAACAATGAGTTATTAGTATACGGTG
CTGAACAAGCAATCGATCAA**ATGAA**AT**ATGAA**ATCGCTAGCGAATTTGGTGTAAACCTTGGTGCTGATAC
A**ACTGC**ACGTGCA**AATGG**ATCAGTAGGTGGCGAAATCACAAAGCGTCTTGTGCAATTAGCTGAGCAACAA
CTTGGCGGCGGACGTTTCTAAGACAACTTTATAATTGATGGCTGAAGGAA**GAATGG**GTCC**CCATTC**TTCC
TTTTTGTACGAATAAAAAAGGAAGGAGAGAGTATATGCAGCAACATCAATTAGCT

23 761 **3**

[Bacillus thuringiensis] serovar konkukian str. 97-27 chromosome, complete genome NCBI Reference Sequence: NC_005957.1: c2943911-2943699 SASP

ATGGCAAA**CAAGA**ACAGTGGAAGTCGTA**ATGAAT**TGTTAGTGCGAGGCGCTGAACAAGCTCTTGATCAA**A**
TGAAAT**ATGAA**ATTGCA**CAAGA**GTTTGGTGTACAACTTGGTGCAGATACAACAGCTCG**TTCAA**ACGGATC
TGTTGGTGGTGAAATTACAAAACGTTTAGTAGC**AATGG**CTGAACAACAACTTGG**TGGTA**GAGCTAATCGCTAA

9 209 **4.3**

Bacillus amyloliquefaciens subsp. plantarum YAU B9601-Y2 complete genome GenBank: HE774679.1 SASP

TTGGGCAGACGTCGTGGAGTTATGTC**AGATGA**G**TTCAA**ATATGAGCTGGCGAAGGACCTTGGTTTTTACG
ATACGGTGAAAAACGAGGGCTGGGGAGG**CATTC**GGGCGCGTGACGCCGGGAAT**ATGGTA**AAGCGCGCGAT
TGAGATTGCCGAACAGC**AGATG**GCGGCTCAGAACCAAAATAACCGATAA

7 185 **3.8**

Bacillus velezensis strain SRCM101413 chromosome, complete genome GenBank: CP021890.1: 229419-229670 SASP

ATGATGGACAGGACACTCGGATATTTGCGCGAGTCGCTTTCGAACCACTTGGAGCATGATGTGTGCCGAA
GGATTTATAAAAAGCTTCAGGTGAAGCATT**ATGAA**AATGAGGGCGAGTTTGTGA**AAGAT**CTAAATGATTT
GGA**AATGG**ATGCGC**TGAAT**CAGGTGCTTGAAAAGGAAATCAAATATGCTGAGAACGAGCAG**GATGA**GAAG
CGCACGATGGAAT**TGAATGAA**GTATATGAGCTCCTGTTTTAA

8 248 **3.2**

Bacillus licheniformis strain SRCM100027 chromosome, complete genome GenBank: CP021677.1: 213006-213254 SASP

ATGGACACAACATTGGGCTACCTCCGTGAGTCACTGTCGAATCACCTTGAACACGGCATAGGACAAAACA
TTTACAGAAAAATCGTTTCCGGGCGATA**TGCCA**ATGAGGAAGAGTTTGTCGAGCACTTGGAGGAGCGGGA
AATGGAATTTC**TGAATCAAG**TGCTTGAACATG**AGATGAA**ATATGCGC**TGAATGAA**CAGGACCATAAACGG
A**CAAGA**GAATTAAACGAAGTGT**ATGAACTGC**TGTTTTGA

13 245 **5.3**

Bacillus atrophaeus strain SRCM101359 chromosome, complete genome GenBank: CP021500.1: 227782-228030 SASP

ATGGATAAAACCTTGGGTTATTTACGGGAATCGCTTTCCAATCATCTTGATAACGATGTA**TGCCA**GCGAA
TTTA**CAAGAAGATG**TTAACTCGGCATTATACGGGGGAAGGCAACTTTGTAAAGGATTTAAAT**GATGATGA**
AATGTCTTATCTGAACACTATATTAGAAAAAGAAA**TCAAG**TACGCTCAAA**ATGAACAAGA**CCAACAGCGG
GCAAGCGAGTTAAACGAGGTTT**ATGAAT**TGCTGTTCTGA

12 245 **4.9**

Bacillus subtilis subsp. subtilis strain SRCM100757 chromosome, complete genome GenBank: CP021499.1: 221169-221417 SASP

ATGGATGTTACACTCGGTTATTTGCGCGAATCACTTTCGAATCATCTAGAAA**ATGAA**GT**CTGCCA**GCGTA
TTTG**CAAGAAGATG**CTGGCGAAGCGTTATGCAA**ATGAA**GGGGAATTTGTAAAAGACTT**AGATGA**T**AATGA**
AATGTCTTTTTTGAACCATGTATTAGAAAAAGAAATTAAGTATGCCCA**GAATGAA**CAGGATCAAAAACGG
GCCAAGGAATTAAACGAGGTGT**ATGAATTACTGC**TTTGA

17 245 **6.9**

Bacillus subtilis subsp. subtilis strain D12-5 chromosome, complete genome GenBank: CP014858.1: 2244206-2244352 SASP P

ATGACCAATAAGAATACAAGTA**AAGAT**ATGCATAAAAACGCCCCTAAAGGACACAATCCCGGCCAACCTG
AGCCTCTAAGCGGAAGCAAAAAAGTAAAGAACCGAAACCATA**CAAGA**CAAAAGCACAAC**TCAAGCCAT**GA
TATGTAA

4 143 **2.8**

Bacillus coagulans 2-6, complete genome GenBank: CP002472.1: 31304-31471 SASP

ATGTCTGAACGTTTTAAAGAAGAACTGGCGA**AAGAT**CTCGGCTTTTATGATGTCGTCCAGGAAGAAGGCT
GGGGCGGCATTAAAGCCCGGGATGCCGGGAAT**ATGGTA**AAGCG**TGCCATT**GAACTGGCTGAAGCGCACCT
TGCTTCGGAAAAACAATCGGGCAACTGA

6 164 **3.7**

Geobacillus sp. Y412MC61, complete genome GenBank: CP001794.1: 1747465-1747665 SASP

TTATTCCGTTTTTCGGC**CTGCCA**ATTCGCTTTGTGCTTGAGCAATGAGCCGTTTCGTAATTTCTCCGCCG
ACGGAGCCGTTGGCGCGCGAAACCG**TGCCA**GCCCCGAGCTGTACGCCAAACTCTTGGGCGATTTC**GTACT**
TAATTTGTTCCAACGCTTGTTCGATGCCTGGAACAAGCAGTTTGTTGCTTGAACGA**GCCAT**

5 197 **2.5**

Thermoanaerobacter tengcongensis MB4, complete genome GenBank: AE008691.1: 792422-792740 SASP

TTAAGACCTTCTTTGCATATACTCTG**ATTCACTGCC**TGCAAGGCCTTGCTGACCGCCTTGTGCAGGGGGA
GAACCATATATGCTGATGCCTCTA**GCCATT**GCCGATTCTGCGAA**ATGAACCATTC**GCCTTAACATTTGTC
CTCCTACTTTTCCGCAGTCTC**TACTGC**TTATATGTCCCCAGTAATCTTCAGACCCTTGTTTTACAGGCAA
ACCTAATTCTGAGGCGATTTCGTATTTCATGTTG**TCAAGA**GCTTTATGAGCCTC**TGGTACCATT**TTTGGC
CTGT**CATTC**CAGCTTCCTCT**TGCCATTTCAA**TCCCTCCT

20 315 **6.4**

Bacillus methylotrophicus strain B25 genome assembly, chromosome : BAMMD1 GenBank: LN999829.1 SASP

TTGGGCAGACGTCGTGGAGTTATGTC**AGATGA**G**TTCAA**ATATGAGCTGGCGAAGGACCTTGGTTTTTACG
ATACGGTGAAAAACGAGGGCTGGGGAGG**CATTC**GGGCGCGTGACGCCGGGAAT**ATGGTA**AAGCGCGCGAT
CGAGATTGCCGAACAGC**AGATGG**CGGCTCAGAACCAAAATAACCGATAA

8 185 **4.3**

Bacillus bombysepticus str. Wang, complete genome GenBank: CP007512.1: c3335004-3334870 SASP N

ATGGGTAATCCGAAAAAAA**ATTCAA**AAGACTTTGCACCGAATCATATTGGAACACAATCAAAAAAAGCTG
GTGGAAATAAAGGAAAGCAAATG**CAAGAT**CAAACAGGTAAACAACCGATTGTTGAT**AATGGT**TAA

6 131 4.6

Bacillus halodurans C-125 DNA, complete genome GenBank: BA000004.3: 77286-77471 SASP

ATGAGTCGAAGACGTGGAATCATGTCAGATCGCTTAAAGGAAGAAATTGCGAAAGAGTTAGGGTTCTACG
ATACCGTCCAA**CAAGA**AGGTTGGGGAGG**CATTC**GTGCACGTGATGCAGGAAAC**ATGGT**GAAGCG**TGCCAT**
TGAGCTTGCTGAGCAGCAATTAGCAGAGAGAGAATCTTCACGTTAA

7 182 **3.9**

Bacillus anthracis strain PR09-4, complete genome GenBank: CP012727.1: 3383781-3383930 SASP

ATGGGTAAAAGAAAAGCAAATCATACTATTTCAG**GAATGAATG**CGGCATCTGCACAAGGACAAGGTGCTG
GTTATAACGAAGAGTTTGCAA**ATGAA**AACTTAACTCCTGCAGAACGACAAAATAATAAGAAACGCAAAAA
GAACCAGTAA

5 146 **3.4**

Clostridium beijerinckii strain NCIMB 14988, complete genome GenBank: CP010086.2: 4894410-4894598 SASP

ATGGATAAAGAAAGAGCACAGGAAATTGTTTCATCATCAGT**AATGG**CAAATGTAACACTTAACGGAACTC
CAATTTACATCGAAAGCGTAAATACTAATACAGAA**ACTGC**TAATAT**CCATT**TTTTTAAAAATCCAAAAA**A**
TTCACGCGAAGTGCCTTTAACTAGTTTA**CAAGA**ACATAATAAAAAATAG

5 184 **2.7**

Bacillus mycoides strain M2E_15 contig_238, whole genome shotgun sequence GenBank: LLWA01000150.1: 1332088-1332480 SASP

ATGAACAAAAAACAACAAGGTTACAATAAGGCAACTTCTGGTGCTAGCATCCAAAGCACAAATGCTAGCT
ATGGTACAGAATTCGCAACTGAAACAAATGTACAAGCAGTAAAACAAGC**GAATG**CACAAGCAGAGGCAAA
GAAAGCACAAGCTTCAGGTGCACAAAGTGCAAATGCTAGTT**ATGGTAC**AGAATTCGCAACTGAAACAAAT
GTACAAGCAGTAAAACAAGC**GAATG**CACAAGCAGAGGCAAAGAAAGCACAAGCTTCAGGTGCACAAAGTG
CAAATGCTAGTT**ATGGTAC**AGAATTTGCAACTGAAACGGATGTGCATGCAGTGAAAAAACAAAATGCACA
ATCAGCTGCAAAAAAATCACAATCTTTTAGCTCTAATCAATAA

12 389 **3.1**

Geobacillus sp. WCH70, complete genome GenBank: CP001638.1: c900227-900018 SASP

ATGG**CAAGATCAAG**CAATAAATTGCTTGTTCCTGGAATTGAACAAGCGTTAGATCAAATAAAAT**ATGAA**A
TTGCC**CAAGA**ATTTGGCGTACAATTAGGAGCAAATACGGTTTCTCGCGCA**AATGG**CTCTGTCGGCGGCGA
AATTACAAAACGGTTAGTCGCACAAGCACAAAGCCAACTTTCCGGATCTGCTTTCCAAAAAAACCGAATAA

6 206 **2.9**

Parageobacillus toebii NBRC 107807 strain DSM 14590 Ga0265171_11, whole genome shotgun sequence GenBank: RCWX01000001.1: 279784-279993 SASP F

TTAAAATTGTTGTTGAGTACCGCCTAATTGTTGTTGC**GCCAT**AGCAACAAGGCGTTTTGTGATTTCTCCC
CCAACAGAACCGTTAGCGCGAGAAGTAGTATCAGCGCCTAATTTTACGCCAAATTCTTGAGCGATTTCGT
ATTTCATTTG**TTCAAGA**GCTTGTTGTGCTCCTGGAACAAGCAATTGATTCGAATTGTTGTTGCG**TGCCAT**

6 206 **2.9**

Parageobacillus toebii NBRC 107807 strain DSM 14590 Ga0265171_11, whole genome shotgun sequence GenBank: RCWX01000001.1: 1356834-1357019 SASP

ATGGCAAAACAACAACCAAACAAAACAGCTGCAGGCACTAA**CATTCAAGA**AGTAAGACAACAAAACGCAC
AATCTGCACAAGCAGGTCAATTCGGCAC**TGAAT**TTGCTGCAGAAACTAATGTGCAACAAGTAAAACAACA
AAACGCGCAAGCAGAAGCTCGCAAAGCGCAAA**ATTCAAG**CAAATAA

9 182 **5**

Geobacillus thermodenitrificans NG80-2, complete genome GenBank: CP000557.1: 895067-895318 SASP

TTATTCTGTTTTTCGGC**CTGCCA**GCTGGCTTTGGGCTTGAGCAACAAGCCGTTTTGTAATTTCCCCGCCG
ACGGAACCGTTGGCGCGCGAAACAG**TGCCA**GCCCCGAGCTGTACGCCAAATTCTTGAGCGATTTCGTATT
TAATTTGTTCCAATGCTTG**TTCAA**TGCCTGGAACAAGCAGTTTGTTGCTTCCACG**TGCCATCATTC**TTGC
CTCCTTTGTCATGTTTAAGTGTGGCATCATTAGTATGAGCAA

7 258 **2.7**

Carboxydothermus hydrogenoformans Z-2901, complete genome GenBank: CP000141.1: c1047458-1047186 SASP

GTGAAATCA**CCATT**GGAAAAACTTAAATATGAGGTTGCCGGGGAACTGGGCATCGGCACCGACGACACCA
CCTACAGACAAAACTTAGAAAAA**ATGAA**AATCGAAGCCGCCAGGGAAATCGGTATTTATGACCAAGTTAA
AGACGGC**TACTG**GGGTGAAGTTCCATCCCGG**GAATG**CGGCCGGGTTGGTGGAAGGCTTGGTGGAAAAATT
GG**TGGTA**AC**ATGGTA**AAAAAATTAATTGCCTTGGCAGAACAGCAATTACAACAGAAG**TGGTA**G

8 269 **3**

Paenibacillus alvei isolate Paenibacillus B-LR1 genome assembly, chromosome: PBLR GenBank: LS992241.1: 460538-460756 SASP

ATGTCTAATCGTTCTGGTTCCAACAACTTGGTTGTTCC**TCAAG**C**TACTGC**TGCATTGGAGCAA**ATGAA**AT
TCGAAATTGCTCAGGAATTGGGCGTTCCAATTCCA**CAAGA**CGGCTACTACGGGAACTACACTTCCCGTGA
TACCGGTTCTATCGGGGGATACATGACGAAGCGTTTGGTGCA**AATGG**CACAACAATCGCTTTCCGGCGGT
TCCCGCTAA

6 215 **2.8**

Paenibacillus alvei isolate Paenibacillus B-LR1 genome assembly, chromosome: PBLR GenBank: LS992241.1: c457500-457273 SASP

A**TGCCA**AATCAACAATCAGGAAGCCGCTCCAATAATCTAGTTGTTCC**TCAAG**CAACAGCAGCATTGCAAC
AA**ATGAA**GTTTGAGATTGC**TCAAGA**ATTGGGTATTCCAATTTC**TCAAGATG**GTTACTACGGTAACTATAC
TTCTCGCGATATGGGCTCCATCGGAGGCTACATCACGAAGCGTCTTGTACAAATTGCTGAACAGCAATTG
GCTGGCCGCGCTCGCTAA

9 224 **4**

Bacillus filamentosus strain Hbe603, complete genome GenBank: CP011974.1 :2734256-2734435 SASP H

ATGGATGCGCAAAGAGCA**CAAGA**AATTTCTA**ATTCAA**CGGACATGGCAAATATAATGTATAATGGAAAAA
GTGTTTATATTGAACATGTAGAC**CAAGA**A**AATGG**GACAGCTACA**ATTCA**TAATATTGACGAACCAAATAA
TAAACAAAGTGTTTCTATATCAGATTTAACAGAACAATAA

6 176 **3.4**

Bacillus anthracis strain Ames chromosome, complete genome GenBank: CP009981.1: 3625253-3625456 SASP C

ATGGCAAACCAAAATTC**TTCAA**ATCAATTAGTAGTACCAGGAGCAACAGCTGCAATCGACCAA**ATGAA**GT
ACGAAATCGC**TCAAGA**ATTTGGTGTACAATTAGGAGCAGATTCTACAGCTCGTGCTAACGGTTCTGTTGG
TGGCGAAATCACAAAACGTCTAGTTGC**AATGG**CTGAGCAAAGCCTTGGCGGATTCCACAAATAA

5 200 **2.5**

Bacillus paralicheniformis strain BL-09, complete genome GenBank: CP010524.1: 57263-57448 SASP

ATGGGCAGACGTCGAAGCATCATGTCAGAGGA**ATTCAA**AA**ATGAAT**TGGCTAAGGATCTTGGATTTTACG
ACACGGTAAAAACCGAGGGCTGGGGCGGAATTCGGGCCAGAGACGCCGGTAAC**ATGGT**GAAGAGG**GCCAT**
TGAATTAGCCGAACAGCATATGGCTCAAAACCAGCAAAATCATTAA

7 182 **3.9**

Bacillus paralicheniformis strain BL-09, complete genome GenBank: CP010524.1: 227024-227272 SASP

ATGGACACAACATTGGGCTACCTCCGTGAGTCACTGTCGAATCACCTTGAACACGGCATAGGACAAAACA
TTTACAGAAAAATCGTTTCCGGGCGATA**TGCCAATGAA**GAAGAGTTTGTCGAGCACTTGGAGGAGCGGGA
AATGGAATTTC**TGAATCAAG**TGCTTGAACATG**AGATGAA**ATATGCGC**TGAATGAA**CAGGACCATAAACGA
A**CAAGA**GAATTAAACGAAGTGT**ATGAA**CTGTTGTTTTGA

13 245 **5.3**

Sporosarcina globispora strain DSM 4 scaffold1, whole genome shotgun sequence GenBank: LGUF01000007.1: 3756751-3756900 SASP O

ATGGCTAAAAGAAAGGCTAATCATATTATCGAGG**GAATGAATG**CTGCAAAAGGCCAGGGCATGGGCGCGG
GCTACAATGAGGAATA**TGCCA**ATGA**GCCATT**AACTGAAGCACAAAAACAGAACAATAAAAAGCGGAAAAA
GAATCAGTAA

7 146 **4.8**

Bacillus infantis NRRL B-14911, complete genome GenBank: CP006643.1: c2178064-2177921 SASP P

ATGAACAAAAACGACAGCA**AAGAT**ATGCACAAAAATGCCCCAAAAGGAAATAACCCGGGTCAGCCTGAAC
CGTTAAGCGGCTC**CAAGA**AAGTAAAAAAACCGCAACCATA**CAAGA**CAAAAGCATA**ATTCA**GGGCACGACATGTAA

5 140 **3.6**

Bacillus firmus strain NCTC10335 genome assembly, contig: ERS1247816SCcontig000001, whole genome shotgun sequence GenBank: UFTC01000001.1: 1286847-1287047 SASP A

CTAGCGTGCTCCGCCTAGCTGCTGCTCC**GCCATT**TGCACAAGGCGCTTCGTAATTTCTCCGCC**TACTG**AT
CCATTTGCTCTTG**AAGATG**TTTCCGGGCCAAGGTTCACACCGAATTCTGTTGCAATTTCATACTTCATTT
GATCAATTGCCTGACTGACTCCTGATACTAAAAGCTGATTAGAATTGCTGTTTCT**TGCCAT**

8 197 **4.2**

Bacillus krulwichiae strain AM31D chromosome, complete genome GenBank: CP020814.1: 642664-642843 SASP B

ATGGATAGACAACAACAACAAGCGTCTAAAACAAACGCACAACAAGTTAGAAGACAAAACGCTGCTTCTG
CACAAGGTGCAGATACAGAATTCGCGAGCGAAACTGATGTA**CAAGA**AGTACGTCAGCAAAATGCTCAGTC
TCAAGCTCGCAAGCAGCAAAACTC**TTCAAGA**CAACAGTAA

5 176 **2.8**

Bacillus krulwichiae strain AM31D chromosome, complete genome GenBank: CP020814.1: 1113182-1113388 SASP C1

ATGGTAAACAACAACAACAACAGTAATCAAT**TACTG**GTTCCAGGAGTACAGCAAGCATTAGACCAA**ATGA**
AATACGAGATTGCTTC**TGAAT**TCGGTGTTGAACTTGGTGC**AGATG**CAACAGCACGTGCAAACGGTTCTGT
TGGCGGAGAAATCACCAAGCGTCTCG**TTCAA**CAAGC**TGAAT**CTCAGCTTGGAGGTTCACAATTTTAA

8 203 **4**

Bacillus krulwichiae strain AM31D chromosome, complete genome GenBank: CP020814.1: 2324595-2324801 SASP C2

ATGGCAAGCAACAACAATAG**TTCAA**ATCAATTACTAGTTCCTGGAGTACAACAAGCTTTAGATCAA**ATGA**
AGTATGAGATTGCA**CAAGA**ATTTGGTG**TTCAA**CTTGGCGCTGATACAACCTCTCG**TGCCA**ACGGTTCAGT
TGGTGGCGAAATCACAAAACGTCTTG**TTCAA****ATGG**CTGAACAACAATTAGGCGGTTCACAATTCTAA

7 203 **3.5**

Bacillus krulwichiae strain AM31D chromosome, complete genome GenBank: CP020814.1: c3432677-3432468 C3 SASP

ATGGCAAACAACAATAGCAACA**ATTCAA**ATCAACTTCTTGTTCCTGGTGTACAACAAGCACTAGATCAA**A**
TGAAGTATGAGATCGCTTCTGAGTTTGGTG**TTCAA**CTTGGTCCTGACGCAACTTCTCGTGCTAACGGTTC
TGTTGGTGGAGAAATCACTAAGCGTTTAGTACA**AATGG**CTGAACAACA**AATGG**GTGGTT**TTCAA**CAATAA

7 207 **3.4**

Geobacillus subterraneus strain KCTC 3922, complete genome GenBank: CP014342.1: 1252984-1253166 SASP H

TTAC**GAATG**CTCTTCTAAGCTGGCGACCGGCACGCTTTGTTTTTGATTCGGATTG**TCAAG**CGGATGGATG
GT**TGCCAT**CCCTTTTTGCTGATCGACATGCTCAATGTAAATGCGCTGGCCGTTGTACGTGA**CATTC****GCCA**
TCACTGGC**GATGA**GGCGATTTCTTGCGCCCGACGAGCATCCAT

7 179 **3.9**

Bacillus toyonensis BCT-7112, complete genome GenBank: CP006863.1: 3120802-3121083 SASP

ATGAGTAAAAAACAACAAGGTTATAACAAGGCAACTTCTGGTGCTAG**CATTCAA**AGTACAAATGCTAGTT
ATGGTACAGAGTTTGCGACTGAAACAAATGTACAAGCAGTAAAACAAGCAAACGCACAATCAGAAGCAAA
GAAAGCACAAGCTTCTGGTGCACAAAGTGCAAACGCTAGTT**ATGGTAC**AGAATTTGCAACTGAAAC**AGAT**
GTGCATGCTGTGAAAAAACAAAATGCAAAATCAGCTGCAAAACAATCACAATCTTCTAG**TTCAA**ACGAGTAA

11 278 **4**

Thermoactinomyces vulgaris strain DSM 43016 Ga0070019_102, whole genome shotgun sequence GenBank: REFP01000008.1: 146200-146412 SASP

ATGGCACAA**CAAGAT**CGCACTCGCAGTTCTAATCAATTGCTGGTGGCTGGTGCTGC**TCAAG**CAATCGATC
AA**ATGAA**ATATGAGATTGC**TCAAGA**GTTTGGCG**TTCAA**TTGGGAGCTGACACCACTTCCCGCGCAAACGG
TTCTGTCGGGGGAGAAATCACGAAACGTCTGGTTTCCATGGCTCAACAACAGCTTGGAGGAAGCAACTTCTAA

7 209 **3.4**

Thermoactinomyces vulgaris strain DSM 43016 Ga0070019_102, whole genome shotgun sequence GenBank: REFP01000008.1: c152962-152672 SASP

AT**GATGAA**CAACAAAAACTTTGGACCG**CAAGA**ATCCAAAACCAATGCACAACAAGTTCGTCAGCAAAATC
AGCAATCCGCTCAACGTCAAACCGGTAAAT**ATGGTAC**CGAGTTTGCTTC**TCAAG**CTGGTGCAGCTCAACA
ACAACAACAGCAACAACAAG**CTCGCCA**AGCTCAACGCCAAGGGCAACAAGC**TGGTA**AATTCGGAACTGAG
TTTGCTTCCGAAACCAATGCACAACAAGTTCGTCAACAAAATCAGCAATC**TCAAG**CCAAAAAAGGCCAAC
AACAACAATAA

12 287 **4.2**

Thermoactinomyces vulgaris strain DSM 43016 Ga0070019_102, whole genome shotgun sequence GenBank: REFP01000008.1: 290944-291132 SASP

GTGGCCAACCGTTCCAACCGATTGCTCGTTCCCGGTGCCCGGCAGGTCTTAAACCAG**TTCAA**AGAAGAAA
TTGCTTCGGAATTCGGAGTGAAGTTGGGTTCTGACACGGCGGCCCG**TGCCAATGGT**TCCGTCGGCGGTGA
GATCACGAAACGGCTCATTTCGCAAGCGCAAAAACAAT**TGAATGAAT**AA

8 185 **4.3**

Bacillus subtilis subsp. spizizenii strain SW83 chromosome GenBank: CP030925.1: 192171-192419 SASP

ATGGATGTTACACTCGGTTATTTGCGCGAATCACTCTCGAATCATCTAGAAA**ATGAA**GT**CTGCCA**GCGTA
TTTG**CAAGAAGATG**CTGGCGAAGCGTTATGCAA**ATGAA**GAGGAATTTGTAAAAGACTT**AGATGA**TA**ATGA**
AATGTCTTTTTTGAACCATGTATTAGAAAAAGAAATTAAGTATGCCCA**GAATGAA**CAGGATCAAAAACGG
GCCAAGGAATTAAACGAGGTGT**ATGAATTACTGC**TTTGA

16 245 **6.5**

Bacillus simplex strain SH-B26, complete genome GenBank: CP011008.1: 2256208-2256360 SASP O

TCATTGGTTTTTCTTTCTTTTTTTATTATTTTGCCTTTGCATTTCCGAT**AATGG**CGGTTCATTTCCCAGT
TCATCATTATACCCAGCT**CCATT**ACCTTGCGCTTTTGCAG**CATTCA**TACC**TGGTA**TAACATGATTTGCCT
TTTGTTTTGTCAT

5 149 **3.4**

Aneurinibacillus soli DNA, complete genome GenBank: AP017312.1: 1462925-1463125 SASP A2

TTATGCACGACCGCCAAGCTGTTGTTCA**GCCATT**TGTACAAGGCGTTTTGTAATCTCGCCACCTACAGAA
CCGTTCTGGCGAGAAGTTGTGTCCGGCCCGAGTGTTACACCGA**ATTCA**GAAGCGATCTC**GTACT**TCATCT
GATCCAGCGCGTTTGCTGCTTG**TGGTAC**GACAAGCTTGTTTGTGTTCGAATTTTGA**GCCAT**

7 197 **3.6**

Aneurinibacillus soli DNA, complete genome GenBank: AP017312.1: 2346698-2346907 SASP C1

ATGTCACAGCAACAAAATAGAAATCGTAATAACA**ATGAA**CTGGTTGCTCCACAAGCACTGGATAT**GATGA**
AGT**ATGAA**ATAGCTTC**TGAAT**TCGGTGTACAACTCGGACCGGATACAACTTCTCGCCAGAACGGTTCTGT
CGGAGGCGAAATTACAAAGCGCCTGGTTCAGATAGCAGAGCAACAATTAAG**TGGTA**AATACGTGCAATAA

6 206 **2.9**

Aneurinibacillus soli DNA, complete genome GenBank: AP017312.1: c3048746-3048531 SASP A3

A**TGCCA**CAACAAAA**TCAAG**GCCGTAATACTAACCAAC**TGGTA**GTACCACAAGCGACTAATGCACTCGATC
AA**ATGAA**GTATGAGATTGCGGCGGA**ATTCA**GTGTACAACTTGGACCAGATACAACTTCTCGCCAGAACGG
TTCTGTCGGGGGCGAGATTACAAAGCGTCTTGTTC**AGATG**GCTGAACAACAACTAAG**TGGTA**AATACGTG
CAATAA

7 212 **3.3**

Aneurinibacillus soli DNA, complete genome GenBank: AP017312.1: c3353234-3353034 SASP C3

ATGGCTAACAACAACAGCTCAAACAAGCTGGCAGTACCACAAGCGGCTAACGCGCTCGATCAA**ATGAA**GT
ATGAAATTGCTTCTGAGTTCGGCGTACAGCTTGGACCGGATTCTACATCTCGTGC**GAATGGT**TCTGTAGG
CGGCGAGATCACGAAGCGTCTTG**TTCAAATGG**CTGAACAACAGCTCGGTGGCCGTGCATAA

7 197 **3.6**

Lysinibacillus sphaericus C3-41, complete genome GenBank: CP000817.1: 70145-70306 SASP

ATGTCACCTCGATTAAAAGAAGAGATCGCAAAAGAACTTGGATTTTATGATGTTGTGCAAAAGGAAGGCT
GGGGCGGCATAAAAGCTCG**AGATG**C**TGGTA**AT**ATGGTCAAG**CG**TGCCATT**GA**AATGG**CTGAGCGAGCAAG
TAATGAG**CAAGA**GCGTAAGTAG

9 158 **5.7**

Lysinibacillus sphaericus C3-41, complete genome GenBank: CP000817.1: c4019787-4019569 SASP

ATGGC**TTCAA**ACAACAACCGCAG**TTCAA**ACCAGCTTGCAGTACCTGGTGTACAACAAGCACTTGATCAA**A**
TGAAATACGAAATTGCA**CAAGA**ATTTGGTG**TTCAA**TTAGGGGCTGACGCTTCAGCTCGTGCTAACGGTTC
CGTTGGTGGTGAAATCACTAAACGTCTTGTACA**AATGG**CAGAATCTCAATTAAAGGGTA**TGCCA**AACAAC
AACCAATAA

7 215 **3.3**

Edaphobacillus lindanitolerans strain MNA4 genome assembly, contig: Ga0116976_13, whole genome shotgun sequence GenBank: FTPL01000003.1: c332939-332733 SASP

A**TGCCA**AACAACGACAACAACTCGAACAAGCTTCTCGTTCCCGGCGTCCGCCAGGCACTCGACC**AGATGA**
AGGAAGAGATCGCCCGCGACTTCGGCGTCCAGCTTGGCGAAAACACGTCAGCGCGTGCCAACGGATCCGT
CGGCGGAGAAATCACAAAGCGCCTCGTTCGCCAAGCCCAGCAGC**AGATGAA**GGGCCCG**CAAGA**ATAA

8 203 **3.5**

Edaphobacillus lindanitolerans strain MNA4 genome assembly, contig: Ga0116976_13, whole genome shotgun sequence GenBank: FTPL01000003.1: c332939-332733 SASP

A**TGCCA**AACAACGACAACAACTCGAACAAGCTTCTCGTTCCCGGCGTCCGCCAGGCACTCGACC**AGATGA**
AGGAAGAGATCGCCCGCGACTTCGGCGTCCAGCTTGGCGAAAACACGTCAGCGCGTGCCAACGGATCCGT
CGGCGGAGAAATCACAAAGCGCCTCGTTCGCCAAGCCCAGCAGC**AGATGAA**GGGCCCG**CAAGA**ATAA

8 203 **4**

Parageobacillus thermoglucosidasius strain DSM 2542 chromosome, complete genome GenBank: CP012712.1: 1972688-1972837 SASP O

CTATTGATTCTTTTTTTCTTTTCTTGTTGTTTTGGCGCTGCGCTTCTGTC**AATGG**CTCGTTGGAAAATTCT
TCGTTATAGCCGGCTCCCTTTCCTTGCGCGTCCGCCG**CATTCA**TGCCGG**GAATG**ACATGATTGGCTTTCC
GTTTGCCCAT

4 146 **2.7**

Bacillus aryabhattai strain AB211 scaffold11, whole genome shotgun sequence GenBank: MCAN01000003.1: 1288271-1288450 SASP H

ATGGATGCACAAAGAGCA**CAAGA**AATTGCTG**ATTCA**GTCGACATGGCCAATGTAATACAT**AATGG**GAAAA
GCATTTACATTGAGCATGTAGACCAACAA**AATGG**GATAGCTACA**ATTCA**TAACCTC**GATGAA**CCAAGTAA
TAAGCAAAGTGTTTCTATATCTGAACTAGCAGAACAATAA

7 176 **4**

Bacillus loiseleuriae strain FJAT-27997 Scaffold1, whole genome shotgun sequence GenBank: LFZW01000001.1: 1923819-1923965 SASP O

T**TACTG**GTTTTTCTTTCGCTTTTTATTATTTTGTTTTTGTTCCTCTGTTAAAGGTTGAGAAAATTCCTCG
TTATATCCTGCACCTTTCCCTTGTGCGCTAGCTG**CATTCATGCCA**GGAATTAT**ATGGT**TTGCCTTACGTT
TA**GCCAT**

6 143 **4.2**

Geobacillus stearothermophilus 10, complete genome GenBank: CP008934.1: 325050-325199 SASP O

ATGGCAAAGCGAAAAGCAAACCACGTAATCCCTGGC**ATGAA**CGCCGCCAAAGCCCAAG**GAATGG**GAGCCG
GCTACA**ATGAA**GAGTTTTCCA**ATGAA**CCGCTGACAGAAGCACAGCGCCAAAACAACAAAAAACGAAAAAA
GAACCAATAG

5 146 **3.4**

Bacillus rhizosphaerae strain SC-N012,DSM 21911,NCCB 100267 genome assembly, contig: Ga0075195_103, whole genome shotgun sequence GenBank: FRBS01000003.1: 254907-255107 SASP

TTATCTTTGAAATCCGCCTAATTGTTGTTCAGCTTGAGCAACCAAACGTTTCGTGATTTCGCCACCTACA
GAACCGTTTGCACGAGAAGTCGTGTCAGCTCCAAGGTTTACGCCAA**ATTCA**GAAGCGATTTCGTATTTCA
TTTGA**TCAAG**TGCTTGTTGTACGCCA**GGTACT**ACCAAGTTATTTGAGTTTGAGTTA**GCCAT**

5 197 **2.5**

Virgibacillus chiguensis strain CGMCC 1.6496 genome assembly, contig: Ga0104465_104, whole genome shotgun sequence GenBank: FQXD01000004.1: 159098-159298 SASP

ATGGCTCGACGAAACCGTAATCAGTTAGAAG**TGCCA**GGTGCAGAAAAAGCAGTCGACTCCCTAAAGTATG
AGATCGCTA**ATGAAT**TTAATGTCGATCTTGGCGCCGATAC**TACTGCAAGA**GAA**AATGGT**TCCGTGGGTGG
CG**AGATGGT**GAAGCGTATGATAAA**AATGG**CAGAA**CAAGAT**CTCGCAAATAGACAGAAGTAG

13 197 **6.9**

Observed AL pentamer frequency: 4.49
Expected: 22/1024 ± 1.65x(22x1002/1024^{2}x100)$^{1/2}$ ≈ 2.15% ± 0.25%*

4.49% ≈ 2.15% + 15 s, where s ≈ 0.15%

P(Observed ≥ 4.45%) ≤ exp(-225) ≈ $(e^{-2.25})^{100}$ ≈ 10^{-100}

AL-pentamer proximity for the 20 aminoacid–tRNA ligases of Deinococcus radiodurans R1

Deinococcus radiodurans R1 chromosome 1, complete sequence GenBank: CP015081.1: c651235-649715 glycine--tRNA ligase

ATGCCCGCAACCTC**AATGG**AAGAACTCGTCAGCCTGTGCAAACGCCGGGGCTTTATTTTTCAGGGCAGTG
AGATTTACGGCGGCCTGCAAGGCTTTTACGACTACGGCCCCCTCGGCGTGGAGCTGAAGAACAACA**TCAA**
GGCGGCGTGGTGGCGCTCCAACGTCTACGAGCGCGACGACATGGAAGGGCTGGACGCCTCCATCATCATG
CACCGC**ATGGT**GCTGCGACACTCGGGCCACGAGGCGACGTTCTCCGACCC**GATGA**TCGACAA**CAAGA**AGA
ACAACAAGCGCTACCGCCTCGACCACCTTCTCAAAGACCAGAAAGCCGACGTACAGGCCAAAGTCGCCGA
AATTATGGGTGAGAGTGCCGACAACTTTGCCGCGCTGGTGGCTGCGCTGAACGCCAAGCCCGCCCAGGCC
TCCGCCG**CATTCAAG**GAAGCGGGCGTGCGTGACCCCTTCTCCGGCGAGGTAGGCGAGTGGACCGACCCCA
AGCCG**TTCAA**CATGATGTTCCGCACGACCATCGGCCCGGTGGCCGACGAGGAAAGTTACGGCTACCTGCG
TCCCGAAACCGCGCAGGGCATCTTCACCAAC**TTCAAGA**ACGTGGTGGACTCGACCAGCCGCCGC**CTGCC**C
TTCGGCATCGCGCAGATCGGCAAGGCGTTCCGCAACGAGATCACGCCGCGCAACTTCATTTTCCGGGTGC
GTGAGCTAGAGC**AGATG**GAAATTGAGTTCTTCGTGACGCCCGGCACCGACGAGGAGTGGCACGAGCACTG
GCTGGAAAAGCGCCTGA**AATGGT**GGGAAGACCAGGGCGTGCCGCGCGAG**AAGAT**CGAGATTCTGGATGTG
CCGAAAGAAGACCTCGCGCACTACTCCAAGCGCACCTACGACCTGATGTACGACTACCCCACGCTGGGGC
ACGAGGAAATCGAGGGCATCGCCAACCGCTCCGACTACGACCTCGGCTCGCACACCAAGTCGCAGAGCGA
GCTGGGCCTGGTGGCGAAGGTGGAGGAAAACAACGACTCCATCGCCAAGCTGACCATCCCCCACCCCGAA
ACGAACAAGCCGGTCGTGCCGTTCGTGATCGAGCCGTCGGCGGGGGTAGACCGGGCGATGCTGGCGGTGC
TGAGCGAAGCGTTTACCAAGGAAACGCTGGAAAACGGCAACGAGCGGATTGTGCTGAAGCTGAAGCCGCA
CCTCGCGCCGATTAAAGTGGCGGT**CATTC**CGCTGGCCCGCAACCGGGAAGAAATCACGGACGTGGCCAAA
GCCATCAAGGCCGAGCTTCAGGGCCTCGGCCTGGGCCGGGTGCTGTACGAGGACTCCGGCAACATCGGCA
AGGCGTACCGCC**GCCAT**GACGAGGTGGGCACGCCC**TACTGC**GTCACCGTGGACTTCGACACCGTCGGGCT
GGGTGAGAACACGGACGAGAGCCTGAAAGACACCGTGACCGTCCGTGACCGCGACACGCTCGCGCAGGAG
CGCGTGAAAATCAGCGAACTGGCGGGGTGG**ATTCA**GGCGAAGTTGCGCTGA

25 1517 **1.6 observed, 2.15 ± 0.6* expected (* 90% confidence interval)**

Deinococcus radiodurans R1 chromosome 1, complete sequence GenBank: CP015081.1 :1441515-1442873 asparagine--tRNA ligase

ATG**ACTGC**TTCATCCCCAGTGACCCGTTCGAGT**ATTCAAGA**CCTGAAACAGCACATCGGGCAGTCCGTCG
AGGTGCTGGCCTGGCTG**CAAGA**CAAGTCCGGCAAGGG**CAAGATTCAA**TTTC**TCAAG**CTGCGCGACGGCTC
GGGCTTCGTGCAGGGCACCGTG**TTCAAG**GGCGACGTGGCCGAGGACGTGTTCGAGGCCGCCAAGCGCCTG
ACCCAGGAACAGGCCGTGCGCGTGACCGGCGAAGTCCGCGCCGACGAGCGGGCTCCCGGCGGCGTGGAAC
TCAGCGTGCGTGACCTCAGCGTCATTTCCGAAAACGTGGGCGAGTACCCGATCACGCCCAAGGAGCACGG
CATCGAGTTCCTGATGGACCACCGCCACCTCTGGCTGCGCCACCGCCGCCCCTGG**GCCAT**CATGCGGGTG
CGCG**ACTGC**GTGCAGCGCGGCATCGTCGAGTTTTTCCACGGCGAGGGCTTCGTGCGCTTCGACGCGCCGT
TCTTCACGCCCAACGCCGCTGAAGGCACCACCGAACTTTTTGAAATCGATCTCTTCGGCGAGGACAAGGC
GTACCTCTCGCAGACCGGGCA**ACTGC**ACGCCGAGGCGGGCGCCTTCGCCTTCGGCAAGGTCTACACCTTC
GGCCCCACCTTCCGCGCCGAGAAGTC**CAAGA**CGCGGCGTCACCTGCTCGAATTCTGG**ATGGT**CGAGCCCG
AAGTCGCGCCGAGCAGCCACGCCGAGAACATGGCGCTG**CAAGA**GCGC**ATGGT**GAGCCACCTCGCCCGCAA
AGTCCTGAGCGAGTGCGCCGCCGAACTCG**AGATG**CTGGGCCGCGACGTAAGCAAGCTCGCTGGGGCCGCC
GAGGGGAACTACCCCCGCGTGACCTACACCGAGGCGCTGGAGAT**CATTC**GGCAGCACATCGAGGACAAGG
AC**CTGCC**GCCCAACGTGCAGGAGGACGTGCAGCCCGTCGAGTGGGGCGACGACCTCGGGGCGCCGCACGA
AACGATTCTGGGCCACCACTTCGACCGTCCGGTG**ATGGT**CGAGAAGTACCCGGCG**GCCATCAAG**GCGTTC
TACATGCAGCCCGACCCCGAAGACCCCCGCGTGGCCCTGTGCGACGACATGATTGCGCCCGAAGGCTACG
GCGAAATTATCGGCGGCTCGGAGCGT**ATTCA**CGACTACGAGCTGC**TCAAG**TCGCGCATCGAAGAGCAGGG
ACTGCCGCTCGAAGCCTTCGAC**TGGTAC**CTCGACCTGCGGCGGGTGGGCAGCATGCCGCACGCGGGCTAC
GGCATGGGCCTGGAGCGCTTCATCGCCTG**GATGA**CCGGCATCGACCACATCCGTGAGGCGATTCCCTTTC
CCCGGATGCTGACGCGC**ATGAA**GCCCTAA

34 1353 **2.5 observed, 2.15 ± 0.6* expected**

Deinococcus radiodurans R1 chromosome 1, complete sequence GenBank: CP015081.1: 80923-82152 tyrosine--tRNA ligase

TCAGCCTTCCAGCACCAGCCGGGCGAACTTGTCCTTGCCCTTCTG**GATGA**CCACGCCTTCCGTCAGTTGC
TCGCGGGTGAGCTGGCCTTGCGGGTCGCTGTAAGTCTCGCCGCCGAGCTTAAGACCCCGGTTC**TGAAT**CA
GTTTGCGGGCGGCGCCGTTGCTGGGTTCCAGGCCCGCCAGCACGACCAGTTTC**GCCAT**GCTGACGTGGCC
CTGTTCGTTCAGCTCACTTGCCGGGACGCTGACGGCGG**GAATG**TTGTCGG**GAATGCCA**CCCTTCGCCACC
GAGCGGAAGCGCGCCTCGGCAGCGTCGAGGTCGGCATCGGGGTGGAAGGCTCGCACCACTTCGCGGGCGA
GTTCGCGGTGGGCGGCGACCGGGTGCCCGGCGAGCAGTTCCTCGATGCGCTCACGCGGCAGGTCGGTCAG
CAGCGTGAAGTAGTTGTCCAGCAGCGGGTCGGGCACCTTCATCAGCCCGGCGAACATGGCGTGCGGCTCG

TCGGTCAGGCCGATATAGTTGTCCAAGCTCTTGGACATCTTCTCGGTGCCGTCCAGGCCGACGAGCAGTG
GCAGCGTCAGCACGACCTGCGCTTCTTGCCCGTAGTCGCGTTGCAGGGCGCGGCCCACCAGGTTGTTGAA
CAACTGGTCGGTGCCCCCGAGTTCCACGTCGGCGTGCAGCGCCACCGAGTCGTAGCCCTGGGTGAGCGGG
TAGAGCAGCTCGTGCATGGAAATCGGGGTGCCGGCGCTGAGGCGCT**TGGTA**AAGTCGTCGCGTTCCA**GAA**
TGCGCGCCACGGT**GTACT**TCGCCGCCAGGCC**GATGA**TGTCCTTGTAGCCAAGCTGTTCGAGCCACTCGGA
GTTGTACCTGAT**TTCAAGA**GCTTCGGGTTCCTGGCGCAGAATCAGGCGGC**ACTGC**GCGAGGTAGCTCTCG
GCGTTGGCGCGCGCTTCTTCCAGGCTCAGCGGCGGGCGGGTCTTGGATTTGCCGCTGGGGTCGCCG**ATGG**
TCGCGGTGAAGTCGCCGATCAGCAT**GATGA**CCTTGTGCCCGAGGTCCTGAA**ACTGCC**GCATTTTGCGCA**G**
AATGACGGCGTGCCCGAGGTGCAGGTCGGGGCGGGTGGGGTCGGCGCCGAGCTTGACGCGCAGGGGCTCG
CCTTTCTCAATCTTGCGCTTGAGGTCTTCTTCGGACACGAGGTCCACCACGCCGCGCTTGAGAAGCTGGA
TTTGCTCATTGACGGGAACATTTCTGCGGATTTCGGACAT

19 1226 **1.5 observed, 2.15 ± 0.6* expected**

Deinococcus radiodurans R1 chromosome 1, complete sequence GenBank: CP015081.1: 104401-106887 glutamine—tRNA ligase

ATGACCGATGCCCCCCGCCCAACCGCCGGGGCCGACGCCCCAGCCCGGCCCCCCG**CTGCC**CCACTCGTCG
CGCCCAACTTCATCACCGAAATCATCGAGCGCGACCTTGAGGCCGGCAAGTACCCCCGCGTCGTGACCCG
TTTTCCGCCCGACCCCAGCGGCTACGCGCACCTGGGGCACGTCTT**TGCCA**GCCTGCTCGAC**TTCAA**CACG
GCGCGGCAGTACGGCGGGCAG**TTCAA**CCTGCGCATGGACGACACCAACCCCGAGCTGGCGCGGCAGGAGT
ACGTGGACTCCATCGCCGACGACCTGAAGTGGTTGGGGCTCGACTGGGGCGAGCACTTCTACTACGCCAG
CGACTATTTCGACCGCTACTACGCCTATGCCGAGCAACT**CATTC**GCCAGGGCGACGCCTACGTGGAAAGT
GTAAGCCCCGAGGAACTGTCGCGGCTGCGCGGCAACGCGACCACGCCCGGCACGCCGAGTCCTTACCGTG
ACCGCTCGGTTGAAGAAAATCTCGACCTGCTGCGCCGC**ATGAA**GGCCGGCGAATTTGCCGACGGTGAGCA
CGTCCTGCGCGC**CAAGAT**TGACCTCACCGCCCCCAAT**ATGAA**GCTGCGCGACCCGGTGCTGTACCGCATC
GTCAACAAGCCGCACTTCCGCACCAGCGACGAGTGGCACATCTACCCCGCCTACGACTTCGAGCACCCCT
TG**CAAGA**C**GCCAT**CGAGGGCGTGACCCACTCCATGTGCAGCCTGGAATTCGTGGACAACCGC**GCCAT**CTA
CGACTGGCTGATGGAAAAACTGAACTTCGACCCGCGCCCCCACCAGT**ATGAAT**TCGGGCGCCGGGGGCTG
GAATACACCATCACCAGCAAGCGCAAGCTGCGCGAACTGGTGCAGGCGGGCCGGGTGAGCGGCTGGGACG
ACCCCCGCATGCCCACCCTGCGTGCCCAGCGCCGCCTGGGGGTCACGCCGGAAGCGGTGCGGGCCTTCGC
CGCCCAGATCGGGGTGAGCCGCACCAACCGCACGGTGGACATCGCCGTGTACGAAAACGCGGTGCGCGAC
GACCTCAACCACCGTGCCCCCCGCGTGATGGCGGTGCTGGACCCAGTGAAGGTCACGCTGACCAATTTGG
ACGGCGAAAAGACGCTCTCGCTTCCC**TACTG**GCCGCACGACGTGGTGCGCGACTCGCCCGACGGTCTGGT
CG**GAATG**CCCGGCGGCGGGCGGGTGGCCCCCGAGGAAGCTGTGCGCGACGTGCCGCTCACCCGCGAGCTG
TACATCGAGCGCGACGACTTCAGCCCCGCGCCGCCCAAGGGC**TTCAAG**CGCCTGACCCCCGGCGGCACGG
TCCGGCTGCGCGGCGCCGGCAT**CATTC**GCGCCGACGACTTCGGGACCGACGAGGCGGGGCAGGTCACCCA
CATCCGCGCCACCCTGCTGGGTGAGGACGCCAAAGCGGCGGGCGTCATCCACTGGGTGAGCGCCGAGCGT
GCC**CTGCC**GGCCGAGTTTCGCCTCTATGACCGCCTGTTCCGGGTGCCGCACCCCGAGGGCGAAAACGCGG
ACGTGGAGGACGACAGCGCCGGCCCCGCCGAACACGAGGCCGAGCCGGGCGCCGGGCAGGAAACGGCGCC
GGTCAGCCAGGGCTTCATGCGCTACCTCACCCCCGATAGCCTGCGCGTGCTGCGCGGTTACGTGGAGCCG
AGCGTGGCCGGCGACCCGGCAGACACCCGCTACCAGTTCGAGCGTCAGGGCTACTTCTGGCGCGACCCTG
T**TGAAT**TGGAACGGGTAGACAGCCGCGAGGACGCGCTGGTGTTCGGGCGCATCATCACCCTGAAAGACAC
CTGGGGCAAGCAGGGCGGCGGCACCCAGCAGAAGGCGGAAGG**CAAGA**AGCGACCCTCTACCAAGGGGAGA
GGGCCTGACGAAGTCAGGGGTGAGGGGTCTTCTTCCCCAGCTAAAGCCCATGCGCCCAAAGCCCAGCCCC
TCACCCCCGAACAGGACGCCGA**ATTCA**CCCGGCTGCTCGGCCTCGGTGCCTCGGAGGGGGACGCCCGCAC
CATCGCCCGTGACCCGGCGCTGCTCGCCTTCGTGGGGGGGGCGGCGCCGGGGGACACCTTCGCTCAGGTC
GCCTCGTGGACAGTCAACGAACTCGTCGCGGGCCTGCGTGCCGGGGAGGTGAAGGTGCGGGCCGCCGACC
TCGCGCCGCTCGCCGAGGGGGTGGCGAGTGGGCAACTCAGCGCGCGCATTGCGCGTGAGGCCCTCGCCCG
CGCCGCCGCCAGCGGGGACGCGCCGCTCACCATCATCGAGCGCGAGGGTC**TGAATG**CCGGCCTGAGCGCC
GAGGCCCTGCAACAGGTAGTGGCCCAAGTCATCGCCGCCAATCCCGACAAGGCGGAGGCCTACCGCGGCG
G**CAAGA**CCGCGCTGCTCGGTTTTTTCACCGGGCAGGTCATGCGCGCGACCGCCGGCAAGGCCGACCCGCA
GGCGCTCGCGGCGGCGCTCAAAGACGCTCTCGCCTGA

26 2483 **1 observed, 2.15 ± 0.5* expected**

Deinococcus radiodurans R1 chromosome 1, complete sequence GenBank: CP015081.1: c156062-154233 arginine--tRNA ligase

ATGGATTTGAAGGCCCAACTCAAAGCAGCCGTCGAGCAGGCCGCGCACC**AGATG**G**GAATG**CCCGTCGACG
CC**GCCATTCA**GGAAACGCCCGCCAATAAACCCGGCGACTACGGCACGCCCGCCGCCTTCC**AGATG**GCGAA
GGCGGCCGGGGGCAACCCCGCCCAGATCGCCGCGCAGCTCGCGCAGACGGTGGTG**CTGCC**CGCCGGCATC
CGCAGGGTGGAGGCCACGGGGCCGTTCCTGAACTTTTTCCTCGACGCGGGCGCTTTCGTGCGCGGCGTGG
TCGAGCGTCCCTTCGAG**CTGCC**CAAGCGCGAGGGCAAGGTCGTCATCGAGCACACCTCGGTCAACCCCAA
CAAAGAGCTGCACGTCGGGCACCTGCGTAACGTGGTGCTGGGCGACTCCATGGCGCGGATTCTGCGGGCG

GCGGGGCACACCGTCGA**GGTAC**AGAACTACATCGACGACACCGGGCGACAGGCCGCCGAGTCGCTGTTCG
CCACCCAGCATTACGGCCGCGTCTGGGACGGCGTGCAGAAGTACGACCAGTGGCTCGGTGAAGGCTACGT
GCAGCTCAACGCCGACCCCCAGAAGCCCGAGCTGGAAAGCGGCATCATGGAGATCATGCACAAGCTCGAA
GCCGGGGAGCTGCGGCCCCTGGTCGAGCAGACGGTGAAGGCGCAGCTTCAGACCTGCTTTCGCCTCGGCG
CGCGCTACGACCTGCTGAACTGGGAATCCGACGTGGTGGGCAGCGGGTTTCTGGCGCAG**GCCATGAA**CAT
TCTGGAAGGCAGCA**GGTAC**ACCTCGCGCCCCACCGAGGGCAAGTACGCCGGGGCGTTCATCATGGACGTG
TCGGAATTTATGCCGGGGCTGGAAGAACCGAACGTGGTCCTCGTGCGCTCGGGCGGCACC**GCCAT**GTACG
CCGCCAAGGACATCGGCTACCAGTTCTGGAAATTCGGCCTGTTCGAGGG**GATGAAATTCAAG**CCCTTCAT
GCAGGACCCCGAAGGCAACACCATCTGGACGAGTGCCCCGGACGGCCAGCCCGAC**GATGA**GCGGCGCTTC
GGGCACGCGCAGGAGGTCATCAACGTGATCGACTCGCGCCAGGACCACCCGCAGACGGTGGTGCGTTCGG
CGCTGGGTGTGGCGGGCGAGCAGGAGAAGGAGGAGCGCAGCATCCACCTCTCCTACGCCTTCGTGACGCT
GGAAGGCCAGACCATCAGCGGGCGCAAGGGCATCGCGGTCAGTGCCGACGACGCGATGGACGAGGCCCAG
AAGCGCGCGCTGAGCGTCTTGCAGGGCATCAATCCCGACCTGGCCGCGCGCGAGGACGCCGCCGAAATCG
CCCGGCGCATCGGCCTGGGC**GCCAT**CCGCTTC**GCCAT**GC**TCAAG**GCCGAGCCGACCCGCAAAATCGACTT
CCGCTGGGAGCAGGCGCTGGCGCTGAACGGGGACACCGCGCCCTACGTGCAGTACGCCGCCGTGCGCGCC
GCCAACATCCTGAAAAAGGCCGAGGAAGCCGGTTA**TGCCA**CCGACGGCACCGGGGCCGACTGGGACGCG**C**
TGCCCGACATCGACCTCGTGCTCGCCAAGCAGATCGCCAAG**CTGCC**CGAAGTCGCCGCGCAGGCCGCCCG
CATCCACTCGCCGCACGTGGTGGCGCAGTACGCGCTCGATCTCGCCACATCC**TTCAA**CGCG**TGGTAC**AAC
GCCAAAACCAAGCAGGGCAAACCCGCCACCAACGTCCTGCAATCGGAAGAAGGGTTGCGGGAAGCGCGGC
TGGCGCTGATCGTGCGGCTGCGCAAAGCCTTCGAGGACACCCTGGACCTCATCGGCATCGAGATTCCGGC
GGCGATGTAA

29 1826 **1.6 observed, 2.15 ± 0.55* expected**

Deinococcus radiodurans R1 chr 1, complete sequence GenBank: CP015081.1:c371799-369337 phenylalanine--tRNA ligase subunit ß

ATGAAACTTCCCTACTCCTGGCTCAAAGAACTGATTCCCGACCTCCCGCCCGTCGCTGACCTCGAACCCA
CCTTCGCGCATCTCGGG**CTGCC**GCTCGAAGGCGTGGAGGACGTGCCCGCGCCGGTGGGCGGCGTGCTGCT
GGTGGCCGTGAAAGCCGCCGAACCGATGGAGGGCACGCAGCTCACCAAACTGACGCTCGACACCGGCGA**G**
AATGGTGAAAAGACCATCGCCAGCGGTGCCCCCAACGCGGTGGGT**CTGCC**GGCGGGCACG**ATGGT**CGCGC
TGGTCACGCCCGGCACCACGCTCGGCGGGATAACCTACGGCGTGCGGCCCATGCAGGGCGTCGAAAGCTG
GGGCATGGCCGCCAGCGCCAAGGAACTCGGCATCGGCGAGAGCAACGCGGG**CATTC**TGACCTTCCCGGCA
GGCACGGCGGCGCCCGGCACACCCCTGCGCGAGCTGTGGCCCGCCGACAGCGTGCTGGACGTGGAAGTCA
CGCCCAACCGCGCCGACGTGCTGAGCGCGCTGGGCCTCGCCCGCGACCTCGCGGCGTACCTGAACCTGGA
GCTGAAAGAACCCCAGATTC**CTGCC**GCACCCACCCAGCCGGGCGAGATCCGCGTCAGC**CTGCC**TGACCGG
GGCCGGGTGCTGGACCGCGACCCGCAGGGCAA**ACTGC**GCTTCGGCTGCGA**CCATT**TCGCCGCCCGCGCCG
TCAGCGGCCTGCAAAACGGCCCCGCGCCGCTGTGGATGCAGCGCCGGGTCAGCCTCGCGGGGATGCGCTC
CATCGACCTGATCGTGGACACCTCCAACTACGTGATGCTCGAACTCGGTCAGCCCACCGCGCTGTACGAC
CGCCGCGACGTGGCCGGGGACGGCCTGGTGGTGGCCTTCGGTCTGCGCGAGGGCGAGACTG**TCAAG**GACC
TGCTGGGGAATACGCATCAGGTCGGCCCGGAGGACCTGCTGATTCTAGACGCGG**GAATG**AGCGACGAACC
GGT**GATGA**CGGTAGCCGAGGCTTT**TGCCA**GCGCCGGGCAGCCCAAGGAAGGCTCGCACGTGCTGGGCATC
GCGGGCATCATGGGGGGCGACCACGGCCACGTGCGTGCCGACACCCGCGACGTGGTGATCGAGTCGGCGC
ACTTCGACCCCGTGTTGCTGCGCCGGACCTCCACCCGGCTGGGCC**TCAAGA**CCGACGCCGTGTACCGCTA
CGAGCGCGGCGTGGACCCGCTGCTGGCGCCCAAAG**CTGCC**GTGCGGGTGGCCGAGCTGCTGCGCGCGGCG
GGCGGCACCCCCGAAGCCGGGCAGACGGTGGTGGGCACCCCCGAAGTGCCGCAGACCATCACCACGACCG
GCGAGCAGATTCGCGCCCTGCTGGGGATGCACATCGGCACGGCGGAAATGCGCGAGAGCCTCACTCGCCT
GGGCTGCACAGTGACGGGCGACGGCGACAGCCTGACCGTCACCCCGCCTTCGTGGCGGGTGGAC**ATGGT**C
ATCTGGCAGGACCTCGCCGAGGAAGTCGCCCG**ACTGC**ACGGCTTTACCGAGTTGCCCGAAACG**CTGCC**CA
CCCTGCGCGTGCACGAGAGCAACATCGGCGCCTCGGCCCAGAGCGAAGCCCGCGCGGA**ACTGC**GGCGCAC
CCTCGCCGGGCTGGGCTTTCAGGAGGTCGTGACTTACACCTTCACCAGCGACGAGGAAGCGCAGAAGGCG
CGGGCCGAGGCGCCCGGCGTGCGCCTGCGTAACCCCATGACCACCGACCGCACTGGGATGCGCACGGCGC
TGTACCCCTCGCTGCTGCGGGCGGCGGGCGCACACCCGAAAGGCGAGCGGGCGC**TACTG**TTTGAAATCGG
GCGGATTTTCCCGGCGGCGGGCGAGCAGGAACGCCTGGGCCTGCTGATGCGCGGCGACCTCGCGGCCCGC
ACCTATCAGGACGGCGTGCGCGGCGATTTCAGCGTG**TTCAAGG**GACTGGTGCAGGGCTTTGCCGGAGCGG
TCGGCGCCAGCTTTGCGCTGGAGCA**ACTGC**GCGGCGACGACGTGCCCGCCGCCCTGCACCCCGGCGTCGC
GGGCGCGGTGGTCTGGAACGGCGAGCGGGTGGGCTGGTTGGGCGCGCTGCATCCCGAAATTGCCCAGGAA
TTTGGCTTGAAAGGCGACACTTTCCTGATGGAAGCCGCG**CTGCC**G**CTGCC**GGGCCGCGACTGGGCCTTCC
GCGACCCCAGCCGTGCCCCCGCCGCGTGGCGTGACCTCGCGGTGATTGCGCCGCAGGGCGTGAGCTACGG
CGACATCGTCGGGGTGCTGAAAGGCGCGGGCGGCGA**ACTGC**TGGAGAGCGTGGAGCCCTTCGACGTGTTC
ACCGGCGAACAGGTGGGCGCGGGCAACCGCTCGGTGGCGGTGCGCCTGACCTACCGGGGCGCCAAAACCC
TGACCGACGAGGAAGTGGACCCGGTG**TTCAA**CGCGCAGATTGACGCGGTGAAGGCGCGGGGCTGGGCGAT
TCGGGAGAAGTAA

32 2459 **1.3 observed, 2.15 ± 0.5* expected**

Deinococcus radiodurans R1 chromosome 1, complete sequence GenBank: CP015081.1: c373751-372732 phenylalanin--tRNA ligase subunit alpha

ATG**CAAGA**CACCGCG**ATTCA**GGAA**ATTCA**GGGGGCCGAGACGCTTGAGGCGCTGCAGGCCG**TCAAGA**CGA
AGTACGTCGGCAAGTCCGGCCTGGTGACGAAAGAACTTGGGAGCCTCGGCAAG**CTGCC**GCCCGAGGAACG
CAAGGCGTGCGGCGCCGAAATCAACGTGGTGCGCGCCGCG**ATTCA**GGCCGCGCTGGACGAGAAAGAGTCA
GCCC**TCAAG**CGCGCGGCACTCGACGCTAAACT**TGCCA**GCGAA**GCCAT**CGACGTGACG**CTGCC**GGGC**CTGC**
CC**CTGCC**GGCGGGCGGGCTGCATC**CCATT**TCCCGCGTGCTCGACGACCTGATCGGCATCTACCGCC**AGAT**
GGGCTACGCGGTGATCGAGGGGCCGGAAGTCGAGGAAGAGCACTACAACTTCGAGGCGCTCAACGTGCCG
TGGTATCACCCCGCGCGCGACCTG**CAAGA**CACCTTCTGGCTGGAAGACGGACGCCTGCTGCGCACCCACA
CCTCGCCCATGCAGATTCGCTAC**ATGGT**GGACCACGAGCCGCCC**TTCAAGAT**CGTGGTGCGCGGCAAGGT
CTACCGCTACGAAGCCACCGACGCCACCCACGAGGCGATGTTTCACCAACTGGAAGGGCTGGTGGTGGGC
GACGGCATCAGCATGAGCGACC**TCAAG**GGGA**CCATT**GCCGA**AATGG**CGCGCGGGCTGTACGGCGCGAGCG
CCAAGGCGCGCTTTCAGCCCAGCTATTACCCCTTCGTGGAACCCGGCGCCGACTTCGCCGT**GTACTG**GGA
AAACCCGCGCGGCGAGAGCAAGTGGCTGGAACTGGGCGGCTGCGGC**ATGGT**GCATCCCAACGTG**TTCAAG**
GCGGTGGACGACCTGCGCGAGGCGGCGGGCAAGGACCGCGTGTACGAGGGCAAAACCGGCTTCGCGTTCG
GGCTGGGGCTGGAGCGCATC**GCCAT**GCTGAAGTACGGGATTCCCGACATCCGCTACTTCTACGCCAACGA
CCCGCGCGTCATCGGGCAGTTCCGGGGGGAACTGG**GATGA**

32 1016 **3.2 observed, 2.15 ± 0.75* expected**

Deinococcus radiodurans R1 chromosome 1, complete sequence GenBank: CP015081.1: c427261-424589 alanine—tRNA ligase

ATG**ACTGC**GCCGCTCACCACCGCCGAGATTCGCGAGA**GGTAC**CTGTATTTCTTCGAAACCAAGGGGCATC
TGCGC**CTGCC**CTCGCACTCCA**CCATT**GCCCCCGACCCCACCACGCTCTTTACGGTCGCTGGGATGCAGCC
C**TTCAAG**GAAAACTTCATGGGCGCGCCCGCCGTGTTCGATGGCCAACCCAGCAAGCGCGTCACCACCGCG
CAAAAGTGCGTGCGCGTGGGCGACATCGAGAACGTGGGCCGCACCCGCCGCCACCTCTCGCTGTTCG**AGA**
TGATGGGCAACTTCTCCTTCGGGGACTAC**TTCAAG**CGCGACGCG**ATTCA**CTGGGCCTGGGAGTTCCTGAC
CGGCCCGGAGTGGATGGGCATGGACAAGGA**CAAGATG**TACGTCACCGTCTACAAGGACGACGACGAGGCG
TTCGGC**TACTG**GACGCAGGACATCGGTTTGCCCGCCGAGCA**CATTCA**CCGCTTCGACGCCGACGAGAACT
TCTGGCCCGCCAACGCGCCGCTCGAAGGCCCCAACGGGCCGTGTGGCCCCTGCTCGGAGATTTACTACGA
CCGTGGCGAGAACTACGGCGACGACACCTGGGGCGACTACTACCAGACCCGCGAGAGCGCCCGCTTTCTG
GAGGTCTGGAACCTGGTGTTCCCGCAGTACGACCGCCAGGACTTGGACGCCAGCGGTCAGCCCGTGC**TCA**
AGGACTTGCCT**TTCAAGA**ACATCGACACCGGGATGGGCCTGGAGCGCGTCGCCAGCGTGGTGCAGGACGT
GCCCGACTTCTATTCCAACGACGTGTTTAAGCCCATCGTCGAGCGGGTGGCCGAGCTGAGCGGCAAGCCT
TACGAGGGCGAAACGAGCGTCTCGCACCGCGTGGTCGCCGAACACATCCGCTCGGTGAGCATGATCGTGG
CCGACGGCACCGCCTTTTCCAACACGGGCCGGGGCTATACCGCCCGCAAAATCATGCGCCGC**GCCATTC**G
TCACG**GGTAC**ATGCTGGGCCTGCGCGAGCCGCAGCTCTACCGGTTGGTCGAACTGGTGGTGGACAGCATG
GGCGGCGCTTACCCCGAGTTGCAGCACAACCAGAGCCGGGTCGAAGCGAGCGTGAGGGCGGAAGAAGAGC
AGTTCC**TCAAGA**CGCTCGAAGGCGGCA**TCAAG**CGCCTGGGCGGGCTGCTGAGCGGCATGGAAAAGGGCAG
CACGCTGGTGGGCAAAGACGCCTTCGAGCTGTACGACACCTACGGCTTCCCGGTGGACCTGACCAAGGAA
ATCGCCGAGGAATACGGCGTGAGCGTGGACGAGGCCGGCTACGCCGAGAGCCTGGAACACGCGCAGGAAA
TCGCCCGCGCGGGCAGCAAGTACGGCAAGTCCGAACTGTTCGGCG**GCCAT**CAGGAAGCGCTCGACGGC**CT**
GCCCGCCACGCAGTTTGTCGGCTACGACCAGACCTCCGGGGACGGTCAGGTGCTGGCGCTGCTGAGCGGC
GGCGAACGCCTGAGCCAC**CTGCC**GGCGGGCAGCGAGGCCACCGTCGTTCTCTCGCAGACGCCCTTTTACG
GCGAAGGCGGCGGCGAGGTGGGCGACACCGGGCGGCTGGAGTGGGACGGCGGGGCCGGTATCGTGCGCGA
CACCCAGAAGACTAAGCAGGGCGTGTTCCTGCACGACGTGCTGGTGGAGCAGGGTGAGCTGAAAGAAGGC
ACCCGCGTGCGCGGCGTAGTCAGCCCCGAGCGGGCG**GCCATTCA**GCGCCACCACACCGCCACCCACCTGC
TGCATGCGGCACTCCGGGCGGTGCTGGGGGGCGGCGTGCAGCAAAAAGGCTCGCGGGTGGCTGCTGACCA
ACTCCGCTTCGACTTCTCGCACGGGGCC**GCCAT**GAGCGCCGAGGAAATCGCCCAGGTCGAAACGCTGGTC
AGCCGCTGGGTGAG**TGCCA**ACTTCCCGGTGTCGTGGCGCGAAATGCCGATTGCCGAGGCGAAAG**CTGCC**G
G**TGCCA**CCGCGCTGTTCGGTGAGAAGTACGGCGACGTGGTGCGCGTGGTGCGGGTGGAAGGCGACGTGGA
CTTTGGCGGCCACGCGGTCTCCAGCATGGAGCTGTGCGGCGGGGCGCACGTCTCGCGTACCGGCGACATC
GGCGCCTTCGTGATTGTCTCCGACGAGAACGTGGCGGCGGGCGTGCGGCGTATCGAAGCGCTGGCGGGCG
AGCAGGCTACGGCGTGGCTGCGCGGGCGCCTGAACGCCTCGGCCAAGGCGGCGGCGCTGCTCAATACCTC
GCCCGAGGGCCTGGAAGAGCGTGTCAGCGGCCTGCAGGGGCAAC**TCAAG**GCCGCCGAGAAGGAAACGGCC
CAGGCGCGCCGCCAACTCGCCGAAGCGC**AGATG**GGCGGGGGTGGCAGCGCGGCCCAGACCCGCGAATTGG
GCGGC**TTCAAG**GTCGCCAGCCTGAAGCTCTCGGGCATCGAGGGCAACGA**ACTGC**GCGGCGCCGCCGACAA
ACTGCTCGACCAGAGCGGCGCGGACCTCGTGGTGATCGCCTCCGACAAGGGCCTGGTCG**TCAAG**GCGACC
AAGGACGCCGTGGCGAAGGGCGCGCACGCCGGACAACTGGTGAGCAAGCTCGCGGCGGCGGGCGGCGGCA
AGGGCGGCGGACGCCCTGACATGGCGCAGGCGGGGATTACGGACGCGGCGGGCGCGTTGGGAGCGCTGGA
CACGGCGTTTTGA

46   2669 **1.7 observed, 2.15 ± 0.45* expected**

Deinococcus radiodurans R1 chromosome 1, complete sequence GenBank: CP015081.1: c554453-551982 leucine—tRNA ligase

ATGACCCAGGAAGCCACTAAACCCAA**CATTCA**GGAACCCCGCGCTGAGCGTTATAACCCGCAC**GCCAT**CG
AACAGAAGTGGCAGGGGCAGTGGCAGGAAAGCGGCCTGTACAAATTCGACGAGAACGCCCCCGGCGAGAA
GTTCTACGCCCTGACGATGTTCCCGTACCCCTCGGGCAACCTGCACATCGGCCAC**TGGTAC**GCCAACGTG
GCCCCCGACGCCCGCGCGCGCTGGCTGCGGATGCGCGGGTATAACGTGCTGTTTCCGATGGCTTTCGACG
CCTTCGGG**CTGCC**CGCCGAGAACGCC**GCCATCAAGA**ACAACACCAACCCGGCGACCTGGACCTACGCCAA
CATCGAGCGCATGACCGGGCAGTTCTCGCGCATGGGCAC**GATGA**TCGACTGGTCGCGCAAGTTCGCCACC
TGCGACCCCGA**GTACT**ACCGCTGGAACCAGTGGTTTTTCATCGAGTTCTGGAAGCGCGGGCTGGCCTACA
AGAAGGGCGGGCTGGTCAACTGGTGCCCCAAGGACCAGACGGTGCTGGCGAACGAGCAGGTCGTGAACGG
CC**ACTGC**GAGCGCTGCGGCACGGCGGTCGAGCGGCGCAACCTGAGCCAG**TGGTAC**CTGAAAATCACCGAC
TACGCCGAGGA**ACTGC**TGGACTTTAGCGCCACCGACATGCCCGAAAAGGTCCGC**GCCAT**GCAGACCAACT
GGATTGGCAAGTCGGTGGGCGCCGAGGTCACCTTCGACACGCCCGCCGGCCCCGAGACGGTCTTTACCAC
CCGCCCCGACACGCTGATGGGCGCGACGTTC**ATGGT**GCTGGCGCCCGAGCA**TGCCA**AGGTGAAGGAATTG
ACCACCGACGAGCAGCGCGCCGAGGTGGAAGCTTACGTCG**CTGCC**GCCGGGCG**CAAGA**CCGACGTGGAGC
GCCAGCAGGAAGGCGAAAAGACCGGCGTGTTCACCGGCAGTTA**TGCCA**CCCACCCCATCAGCGGGCACCA
GTTGCCCATCTGGGTGGCCGACTACGTGCTGGTCACCTACGGCACCGGCTCCATCATGGCCGTGCCCGCG
CACGACGAGCGCGACTTTGCCTT**TGCCAAGA**AGTTTGAC**CTGCC**TATCAGGGAAGTCATCCGCGCCGAGG
GGTCCGAGGGCATGGGCGACCAGCCGAGCGAGCC**GTACT**CGGGCGAGGGCCAGATCGTCAATTCCGGCGA
GTTCGACGGCATGCCCGGCGGCAAGGCGAGCATCGCG**GCCAT**CATTGCCCGGCTGGAGGAACGCGGCGTG
GCGAAGGCCAAAACGACCTACCGCCTGCGCGACTGGCTGTTTGCCCGCCAGCGC**TACTG**GGGCACGCCGA
TTCCCTTCGTCC**ACTGC**GAGAAGTGCGGGATGCAGCCCGTTCCCGAGGACCAGTTGCCGGTGAAGTTGCC
CGAGAACGTGGCCTTCACCCCGACCGGCCAGAGCCCGCTGAAGCTG**GATGAA**GAGTGGAAGACGACCACC
TGCCCCTGCTGCGGCGGCCCCGCCGAGCGCGAAACCGACACCATGGACACCTTCGTGGACTCGAGC**TGGT**
ACATGTACCGCTACCTGTCGCCCAACTACGACGGCGGCCCCTTCGACCCCAGTAAGGCGGGCCTG**CTGCC**
GGTGGACCTGTACACGGGCGGCATCGAGCAC**GCCATT**TTGCACCTGCT**GTACT**CGCGCTTCTGGACCAAA
GTGATGCGCGACATGGGCCTGACGACGCAAAGCGAACCGTTTGCCCGGCTGCGCAACCAGGGC**ATGGT**GC
TGGGCGAGGACGGCGAG**AAGATG**TCCAAGTCGCGCGGCAACGTGGTCGACCCCGACGACCTGGTGCGCGA
GTACGGCGCCGACACGGTGCGGGCGTTCCTGATGTTCATCGCCCCCTGGGAACTGGGCGGCCCCTGGGAT
CCGCAGGGCATCAACGGCCCGAGTAAGTGGCTCTCGCGCGTCTGGAACGTGTTCTTCGAGGAGAAGGTCA
GCGGCCCCGAGGAA**AAGATG**CAAGGCGCCGACGTGCGCTTCGCCGTTCACTCCGCGCTGAAGAAGGTAAA
CGACGACTTCGAGCGCATGAGC**TTCAA**CACCATCATTTCGACCCTGATGGAGCTGACGAACGCGCTGGTC
AAGGCCAAGCGCTCGCCGGTCTTCGGCACGCCCGTCTGGGAGGAAGCGCTGGACATC**TTCAA**CCGCATGC
TGGCCCCCGTCGTGCCCCACATCGCCGAGGAAATCTGGCATGAGCGTGGGCAAAAGGGCAGCGTGCACAC
CGCGCAGTGGCCGCAGGTGGACGAGGCGGCGGCGGTGCGCGACACCGTGACCATCGGCGTGCAGGTGTCG
GGCAAGGTGCGCGGCGAGGTCAGCATCTC**CAAGAC**CGCCAGCCAGGAAGAAGCCCTGAGCGCGGCGCGGG
CCATCGCCGAGGTGCAAAAGCATCTGGAAGG**CAAGA**CCGTCG**TCAAG**GAGATCTACGTGCCGGGGCGGAT
TATCAACATCGTGGCGAAGTAA

47 2469 **1.9 observed, 2.15 ± 0.5* expected**

Deinococcus radiodurans R1 chromosome 1, complete sequence GenBank: CP015081.1: 628397-630346 threonine--tRNA ligase

ATGCACGTCACCCTTCCCGACGGTAAACAACTCGACC**TTCAA**CCCGGCGCCACGGCGCTCGACGCCGCCA
AA**GCCAT**CGGCCCGCGCCTGGCG**CAAGA**CGCCCTGGGCGCCACCGCC**AATGG**CGAACTTACCGACCT**GAT**
GACGCCGCTGAGTGATGGCGCCAGCATCACCCTGATCAC**CAAGA**AGAATCCCGGCGACGCCGCGCCGCTG
TTCCGGCACTCGCTGGGCCACGTCATGAGTCAGGCGGTGGGCGAATATTACAAGGCCAAAGGCTACGGCC
CCGAC**GCCATCAAG**CGCGGGGTGGGGCCGTACATCGAAAACGGC**TGGTA**TCAGGATTTCGAC**CTGCC**CGA
GCCGCTGAAAGAAGAGGAC**CTGCC**CGAGATCGAA**AAGAT**CATGCGCGACATCATCGGGCGCGGCCTGGAC
ATCACCCGGCGCGAAATCAGCAAAGACGAGGCGCTGGCGCAGTTTCCCCACGACCCCTACAAGGCTGAGC
TGATCGAGGGTTTGCCGGACGACGAACCCATCACCTTCTACAGCCAGGGCGACTACACCGACCTGTGCCG
GGGGCCGCACTTCCCTAGCACTGGCAAG**CTGCC**GCAGAGC**TTCAAG**CT**GATGA**GCACCAGCGGCGCGTAC
TGGCGCGGCAACGAGAAAAACCCCATCCTCCAGCGCATCTACGGCGTGGCGTTCGCCACCCAGAAGGAAC
TGGACGAGTACCTGTTTCAGCTCGAAGAAGCCAAGCGGCGCGACCACCGCAAGCTGGGCAAGGAACTTGA
ACTGTTCACCATCGACCCGCTGGTGGGCAAGGGC**CTGCC**GCTGTGG**CTGCC**CAACGGCACGGTGCTGCGC
GAAGAGCTGACCAACTTC**ATGAA**AGAGCAGCAGTTCCAGCGCGGTTACCAGGGCGTGGTGACGCCCAACA
TCGGGAACCTCGACCTGTACCGCACCTCCGGGCACTACCC**GTACT**ACTCCGAGTCTCAG**TTCAA**CCCGAT
TCAGGTGGACGAGGAAGAGTACATGC**TCAAG**CCC**ATGAACTGCC**CGCATCACGTGCGAATTTACGCCAGC
AAGCCGCGCAGCTACCGCGAC**CTGCC**GGTGCGGCTGGCCGAGTTCGGCACGGTGTACCGCTACGAGCAGT
CGGGCGAACTCAACGGCCTGACCCGCGTGCGCGGCTTCACGCAGGACGACGCCCACATCTT**CTGCC**GCCC
GGACCAGC**TCAAG**CGCGAGTTTCTGGACGTGCTCGACCTGACGGTGCTGGTGC**TCAAGA**CCTTCGGCATG
ACCGACGTGCGTTTCCGCGTGGGCGTGCGCGACCCCGAGTCCGACAAGTACGTGGGCGACGAGCAGAACT
GGGCGCTGGCTGAGCGCCAGATTATCGAGGCGGTGGAGGAAGTCGGC**CTGCC**CTACACCATCGAACCCGG
CGACGCAGCCTTCTATGGCCCCAAGCTCGACTTCGTGG**TCAAG**GACGTGCTGGGGCGCGAGTGGCAGCTC

GGGA**CCATTCA**GGTGGACTACAAC**CTGCC**CGAGCGCTTCGACATCTCCTACACCGGCGAGGACGGGCAGG
AGCACCGCCCGATCATG**ATTCA**CCGCGCGCCCTTCGGGTCCATCGAGCGCTTCACCGGCATCCTGATCGA
GCACTACGCGGGCGACTTTCCGCTGTGGCTGGCGCCCCGGCAGGTCATGATCATCCCTATTGCCGACCGC
CACAACGCCTACGCTGAGGAGCTGCGTGAGGA**ACTGC**ACCGCGCCGGGTTGCGCGCCGAGGTGGACGATT
CCTCCAACCGCATGCAGGCCAAGGTCCGCGACGCCGAGCTGCA**CAAGAT**TCCGGTGATGCTGATCGTGGG
CGACAAGGAGCAGGAGGCCCGCGAGGTCAGCGTGCGCGAGCGCACCGGCGAAGGCACCAAGGAGCGCAAG
GGCGTGAAGTTTGACGAGCTGAAGGCGGAATTGCTGGAGCGGCGGAAGAACCGGTCTTAA

40 1946 **2.1 observed, 2.15 ± 0.5* expected**

Deinococcus radiodurans R1 chromosome 1, complete sequence GenBank: CP015081.1: 1028561-1030057 cysteine—tRNA ligase

ATGACCCAGCCCACCATCACCCAGGACACCGCCCGCCAGCCCAACCCCAACATCACCCTCTACGACACCA
TGCAGCGCCAGAAGGTGCCTTTCGTGCCCGGCACGCCCGGCTACGTGGGCATGTACCTGTGCGGCCCGAC
CGTCTACTCCGACGCGCACCTGGGTCACGC**CAAGA**AGGAAGTCGCCTTCGACGTGATTCGCCGCGCCCTG
ACACATTTCGGGTATCAGGTGCGCTACGTCTCCAACATCACCGACGTAGGCCACCTGCTGAACGACGCCG
ACGAGGGCGAGGACAAGTTGCAGGCGCGCGCCCGGCTGGAGCAACTGGAGCC**AATGG**AAGTTGCCGACAA
GTACTTCTGGTCGTTTTTCCGCGATATGGACGCCC**TGAATG**TCCTGCGCCCCAGCATCAACCCGCGCGCC
ACCGGGCA**CATTCA**GGAGCAGA**TCAAG**CTGATCGAGGAACTCATCGAGAAGGGCCACGCCTACGAGTCGG
CGGGCAGCGTCTACTTCGACGTTCGGAGTTGGCCCGAGTACGGCAAGCTCTCGGGCCGCAAACTCGACGA
CCAGGAGGAAGGCACCCGCGAGGCCGTGCGCGACGAGAAACGCGACCCCCGCGACTTCGCGCTGTGGAAG
AAGGCCGAGCCCGAGCACCTGATGCGCTGGGACTCGCCCTGGAGCGTGGGGTTTCCCGGCTGGCACATC**G**
AATGCTCG**GCCAT**GAGCCTGAAGTACCTCGGCGAGGGCTTCGA**CATTCA**CGGCGGTGGCCTCGACCTGCA
ATTTCCCCACCATGAGGCCGAAATTGCCCAGGCGGAGGCGGCGGGGCACCACTTCGCGCGC**TACTG**GATG
CATAACAACATGCTGACCATCGGCGGCGAG**AAGATG**TC**CAAGA**GCAAGGGCAACTTCACGA**CCATTCA**GG
ACATCCTGAAAAAATACGACCCG**ATGGT**GGTGCGCTTCCTGCTGGTGAGCAGCCACTACCGCAGCGTGAC
CG**AGATGAA**CGAGGAAGCCTTTGCGAGCGCGGCGAACGGCTACCGCCGCCTCAGCGAAACGCTGCACGAA
ATCGAGCGCCGCTTGAAGGACGCGCCTGCTGGCTCGGAC**ACTGC**GCTGGACAGCAAAATCGCCGCCCGCG
TGACCGAGTTCGAGGAC**GCCAT**GCGCGACGAC**TTCAA**TACGCCCAAAGCGGTGGCCTCACTGTTCGGGCT
GACCGGCGAGCTGAACACCGCGCTCAATGCCGGGCCAGTGGGACGCGATACCCTGGAGCGCGCGCGCGAC
GCCTACCGCAGCCTCGGCGGCGACGTGCTGGGCCTCTTTGCCGAGACAGGCAGCGCGGCTTCGGTCGCCC
AGGACGACGCCTCAGTGATTGACGCCCTGATGGACCTGGTCCTCAAAGCCCGCCAGAACTACCGCCTGCA
AAAGCAGTACGCCGAGGCCGACGAGCTGCGCGAAACGCTGGGCAAGGCGGGCATCACGGTGGAAGACACC
AAAGACGGCGCGCGCTGGAAACGCTGA

26 1493 **1.7 observed, 2.15 ± 0.6* expected**

Deinococcus radiodurans R1 chromosome 1, complete sequence GenBank: CP015081.1: c1288720-1286675 methionine—tRNA ligase

GTGCAAAATCCCCCCCAGCACCCTGAGGCCCAGTCCCCCGAGACCCGGGACCGTGAGTTTTTCATCACCG
CC**GCCAT**CGACTA**TGCCA**ACGGCACCCCGCACATCGGGCACGTCTACGAA**AAGAT**TCTGGCCGACGCGAT
TGCCCGTTACCAGCGCCTCGCCGGGCGCGACGTGACGTTTGTGATGGGCACCGACGAGCACGGCGAAAAA
ATCAGCAAGGCGGCGGCCAAAGGGGGCGTGACCCCGCAGGAGCTGGTGGACGACCTCTCGGAGCGGGCCT
TTCAGGGCCTGTGGAAAAAGCTCGGCATCAGCTACGACTTTTTATCCGCACCACCTCGGCCAAGCA**CAA**
GAAATATGTGCAGGACGTGCTTCAGCGGGTCTACGATGCGGGCGACATCTACTTTGCCGAGTATGAGGGC
CTCTACTCGGTGGGCGCCGAGCGCTACGTGACCGAGAAGGAACTGGTGGAAGGCCCCGACGGCGTGCGGC
GCTTCCCCGGCGACAAGGACCCGCCCGAGCTGCGGCGCGAGGCCAACTACTTC**TTCAA**CATGCAGAAGTA
CCAGCCCTGGCTGCTGGAGACCTTGCAGCAAAACCCCGACCTG**ATTCA**GCCCGCCGGCTACCGCAACGAG
GTGCTGG**AGATG**CTGAAAGAGGACATCGGCCCACTGAGCATCAGCCGCCCCAAGGCGCGGGTGCCGTGGG
GCATCGAG**CTGCC**CTGGGACACCGACCACGTGACCTACGTGTGGTTCGATGCGCTGCTGAGCTACCTGAC
GCCGCTGGTGAGCCAGGGGCAGGACGCCAGCATGAGCGGCAAGGCGTGGCACGTCATCGGCAAGGACATC
C**TCAAG**CCGCACGCGGTGTTCTGGCCGACCATGCTGCGCGCCGCCGGG**CTGCC**GCTCTACCGCCGCTTGG
TGGTGCACAGCCACATCCTCGCCGAGGACGGGCG**CAAGATG**GG**CAAGA**GCCTGGGCAACGCGATTGACCC
CGAAGAACTGGTTGCCGCGTGGCCGGTGGAC**GCCATTC**GGTATGCGCTGCTGCGCGAGGCGTCGCTGGGG
GCCGACAGTCCCTTCGGTGAGGGC**GTACTG**GTTAGCCGCCTGAACTCGGACCTCGCCAACGACCTCGGCA
ACCTGCTTTCGCGCACCGTGAGCATG**ATTCA**GAAGTACAGAGGCGGCGTGATTCCGGCAGCGACCGAGCC
GACCGACCGCGAACGCGAGATTGAGGCGGCGGCGCGCGCCCTCCCAGACGAAGTGCTGCGGCTGGTGGAC
GAACTGAAAATCAATATGGCGATCGACGCCGC**GATGA**GCTTCGTGCGCGACCTCAACCGCTACATCGCCG
AGTCCACGCCCTGGACCCTCGCCAAGTCGCCGGAGACGCAGGGCCGCCTCGACACCGTGCTCTACACCGC
CGCCGAGGGGCTGCGCGTAGCGAGTGTGGCGCTCGAAGCCGTGATTCCGACCAAGGCGAAGGA**ACTGC**GC
GAGCAGCTCGGCCTGGGCCGCCAAGGCTACCCCCTGCAAGCCGCTTGGGGCCTGACCCCCGCCGGCACCC
GCGTGCAGGGCGGC**GCCAT**CCTGTTTCCCAAGCCCGAGCCGAAGGCGGACGAAACGAAAAACGCCGAGGC
CAAGCCCCCCAAGCCCCAAGC**CAAGA**AAGAGAAAAAGACCGTGACCGAC**ACTGC**TCCCGC**CAAGA**CCACC
GAACAGAAGCCCGAAG**CTGCC**GCCC**CTGCC**CAGAACGACGGCCTGATTTCGATCGACGACTTCGC**CAAGA**

TCGACCTGCGAATCGCCGAAGTCGTGGCCTGTGAAGCGGTGGAAAAGGCCGACAAGCTGC**TCAAG**CTGAC
CG**TCAAG**CTCGGCGACGAGACGCGCACGGTGGTCAGCGGCATCCGCAAG**TGGTAC**GAACCCGAAGCGCTG
GTGGGCCGCAAGGTCGTGCTGGTCGCCAATC**TCAAG**CCCGCCAAGCTGCGCGGCATCGAGTCGCAGGGCA
TGATTCTGGCAGCGGAGGACGACGCCGGGAACCTCGACCTGGTGGGAACCGAGCTGGAC**CTGCC**GAGCGG
GACCAAAGTTCGTTGA

36 2042 **1.8 observed, 2.15 ± 0.5* expected**

Deinococcus radiodurans R1 chromosome 1, complete sequence GenBank: CP015081.1: 1365358-1366641 histidine—tRNA ligase

ATGGCG**ATTCA**GCGTCCCAAAGGCACCAACGACCTC**CTGCC**GAGCGGCAGTCC**CAAGA**CCGAAGCGT**TTC**
AACGGGCCGCGGCCCACGAGTGGCTGATCGCGCAGGCCCGTGAAGTGCTCGAACGCGCCGGGGCGCAGCG
CATCGACACGCCGATGTTCGAGGAAGCCGAACTCG**TCAAG**CGCGGGGTGGGCGGCTCGACCGACATCGTG
CGCAAGG**AGATG**TTTACCGTCTACTACTTCGGCGACCACGGCGGCTACGTGCTGCGTCCCGAGGGCACGG
CGAGCATCGTGCGGGCGTATCTGGAGAACGGGC**TCAAG**CA**ACTGCC**CGCGCCGC**TCAAG**CTCTGGACCTC
GGGGCCGATGTTCCGCGCCGAGCGGCACCAGAAGGGCCGCTACCGCCAGTTCACGCAGGTGGATTACGAG
GTGCTGGGCTCCGCCGACGCGCTCGTCGACGCCGAGGCGATTGCGCTG**ATGGT**GGCGGTGGTGCAAAAGC
TGGGGCTGCGGGGCGTGCGCG**TCAAG**CTCGGGTCCATCGGCGACCCGGAGGACCGCGAGCGCTACAACGC
GTACCTGCGTGATTTGTTCTCGCCGCACTTGGGGCGCCTCTCCGACGATTCCAAAGACCGCCTGGAGCGC
AACCCGATGCGGATTCTGGATTC**CAAGA**GCGCGAGTGACCAGGA**ACTGC**TGGCCGAGCTCAGCGTGCGGC
CCATGCTCGACTTCCTCGGCGAAGGCGCCGCCGCCGACTTCCGGCAGGTGCAGGCCTACCTGAGCGACTG
GGGCGTGCCGTTCGACCTCGACCCGAGCATCGTGCGCGGGCTGGACTATTACCGCCGCACCGCCTGGGAA
CTCCACTACGAGGGCATCGGCGCCAAGTCGGCCCTGGGCGGCGGCGGGCGCTACGACGGCCTGAGTGAAC
AACTCGGCGGCGGCAGCGTGCCGGGCATCGGCTGGGCGTTCGGCGTCGAGCGGCTGTTGCTGGCGCTCGA
GCAGGAAGGCATCGAGATTCCGGGCAGCGGCGGGCCGCAGCTCTACGTCGCGGCGCTGGACGAAGAAAAC
GTGCCGCTGGCGGCGCGGGTGGCGCTCGCGGCCCGCGCGTCTTGCCGCGCCGAGTTCGCCTACCGC**GCCA**
TGAAACCGGGCAGCGCC**TTCAAG**GACGCCGAGCGCCGGGGCGCCGTGTGGGTGGGCCTCATCGGGTCGGA
GGAAGCCGCCGCCGGGACCCTGAGCCTGAAAAACCTGCACACCGGCGAGCAAAAGACCGTGCCGGTCGGG
GAGCTGGGACAGGCCCTTGCTTGA

18 1280 **1.4 observed, 2.15 ± 0.65* expected**

Deinococcus radiodurans R1 chromosome 1, complete sequence GenBank: CP015081.1: 1367030-1368760 aspartate—tRNA ligase

ATGAAACGCACGTCCCTGATCGGCCAGCTCGGCCAAGCGCAGCAACAACAAACCGTCACCCTGCAAGGCT
GGGTCAGCCGCCGCCGCGACCTGGGCGGCCTGATTTTTCTAGAGCTGCGCGACCGCTCCGGCACCGTGCA
GGTGCAGGTCGAGCCCGATTCTCCCGCTTTTGCCGAGGCCGACCGCCTGCGCGCCGAGTACGTGGCCGAA
ATCGAGGGCACCT**TTCAA**CCCCGCCCCGAGTCGCAGCGCAAGGGGGGCCTGGCCGACTTCGAGGTCAT**TG**
CCAGCCGGG**TCAAG**GTGTTGAACGCCGC**CAAGA**CGCCGCCTTTTGAACTCGACAAGGGCGAGAGCGTGGC
TGAGGATATTCGACTGAAGTACCGCTACCTCGACCTGCGCCGCCCGG**AGATG**CAGCGCGCCCTGATGCTG
CGCTCCAAGGCCGTGACCGCCGTCACCGAATTTCTCGACGCCGAGGGCTTT**ATTCA**GGTCGAAACGCCGA
TGCTCACCAAGTCCACGCCCGAGGGCGCGCGTGACTTTCTGGTGCCCTCGCGCC**TGAAT**CCCGG**TGAAT**T
CTACGCG**CTGCC**GCAGAGCCCGCAGCTG**TTCAAG**CA**ACTGC**T**GATGAT**CGCGGGCTTCGACCGCTACTAC
CAGCTCGCCCGCTGCTTCCGCGACGAGGACCTGCGCGCCGACCGCCAGCCCGACTTCACGCAGCTCGATA
TGG**AGATGA**GCTTTGTCGAGCAGGACGACGTGCTCGAAGTGCAGGAGCGGCTGCTCGCGCACGTGTTCAG
GCGGGTGCTGGACGTGGAGTTGCCTCTCCCCTTTCCGCGCATGAGCTACTTCGACGCGATGGACCGCTAC
GGCTCCGACAAGCCGGATTTGCGCTTCGACTCGGCCTTTACCGACGTGACCGGGCTGTTCCGGGGCGGGG
AATTTGCGGCGTTTGCAAGTGCCCCGAGCGTCAAAGTCCTCGTCGCGCCCGAGCTGACGCGCAAGCAGAT
CGACGAACTCGAGCGCGTCGCCAAGCAAAACGGCGCGGGCGGTCTGGCCTGGCTGCGGCGTGACGGTGAA
GGGTTTACGGGCGGCATCTCCAAGTTCGTGGGCGGGATTGCCCCGCAGCTGATCGAGCAGACCGGCGTCG
CGCAGGGCGGCACGCTGCTGTTCGCGGCGGGCGAGTGGAAAAAAGCGGTCACGGCGCTGGGAGCGGTGCG
CCTCGCGCTGCGGGACCTGTTCGACCTGGCGGCGGGCGGGCCGCAGTTTCATGTGTCCTGGGTGGTGGAC
TTTCCGCAGCTCGAATTCGACGAGGACTCGCAGAGCTGGACCTACATGCACCACCCCTTCACCGCGCCGC
ACCCGGGCGACGTGGCGCTGTTCGGCACCGAGCGGCAGGGCG**AGATG**CGCGCGCAGGCCTACGACCTCGT
GATGAACGGCTTTGAAATCGGCGGCGGCTCGGTGCGT**ATTCA**CGACCCCGAGGTGCAGGCG**AAGATG**TTC
CAG**GCCAT**CGGCTTTTCCGAGGAGGCTGCGCGCGAGAAGTTCGGCTTCTTTCTCGACGCGTTGGAATACG
GCACGCCTCCGCACGGCGGCATCGCCTGGGGCTTTGACCGCCTGCTGATGCT**GATGA**GCGGCGCGGGCAG
CATCCGCGAAGTCATCGCCTTCCCCAAAAACAACCGCGGCGCCGATCTGATGGCGCAGGCCCCCTCCCCC
GTCGAGGATGCGCAGCTCGCCGAAGTGGGCGTGCAGGTTCGGGGCGAATAA

25 1727 **1.4 observed, 2.15 ± 0.6* expected**

Deinococcus radiodurans R1 chromosome 1, complete sequence GenBank: CP015081.1: 1378406-1381642 isoleucine—tRNA ligase

ATGACCAAACGTACCTTCGCCCCTGTCCCCACGCAGCCTAACTTCCGCCAATTGGAAGCCGACATCCTCC
AGA**AATGG**AAG**GATGA**GCAGGTCTTCGAGCAGACGCAGACCCGCCCCGCTCCGGCAGGCGAGTTCGTGTT
CT**ATGAA**GGGCCGCCCACCGCCAACGGCAAGCCCGCCCTCCACCACGTGCTCGCCCGCTCG**TTCAAG**GAC
CTCTTTCCCCGC**TTCAAG**GTGATGCAGGGCCACCACGTCACCCGCAAGGGCGGCTGGGACACCCACGGC**C**
TGCCGGTGGAAATCAGCGTGGAAAAAAAGCTCGGCTGGCTGGGCCGCAACCACGGGGCGAGCCGCGAGGA
ACTCGAGGA**ATTCAA**CCGCCTGTGCCGCACGTCCGTCTGGGAAA**CCATTCA**GGACTGGAACGAACTCACC
GAGCGCATGGGC**TACTG**GCTCGACCTGGGCGACCCCTACATCACCTACCAGAACAGCTACGTGGAGTCGG
TGTGGAACTTGCTGCGGCGCCTGCACGAAAAGGGTCTGGTCGCG**CAAGAT**TACAAAGTGGTGCCGCTCTC
GCCGCGCATCTCCTCTACCCTGTCGCGTGCCGAGCTCGGTGAAGTGGACTCTTACCGC**ATGGT**GGACGAC
CCGAGCGTGTATGTTCGCCTCCCCATCGTCTGGGACACGTTGCCCGAGCGGGCACACGCCGCACTCAGCA
GTCTCAGTGGTGAGCAGCGCCAGGGCCTGAGCCTGCTGGTGTGGACGACGACCCCCTGGACG**CTGCC**GAG
CAACACCCTGG**CTGCC**GTCAACCCGGATCTCGACTACGTGGTCGCCGACAGCCCGAGCGGGCGCGTCATC
GTCGCCGAGGGCGCGGTGGAACGGCTCTCGGCGCTGCACAAGGACGCGGCTCCGCTGGAAGTCCTCGCGC
GT**TTCAAG**GGTCGCGATCTG**GAATGG**GTGGAATACGAACCGCCCTTCCCCGAAGTAGCGTCTCAACTCGG
CGTCGTGTCGGAGCTCCACGAGCGGCGTGACGGCAAGCCGGTCCTGCACTTCGTGGTGATGGCCGACTTC
GTGAGCGATGTGGACGGCTCCGGGGTGGCGCATGAGGCGCCCGCTTACGGCGCGGAGGACTTGGAGGTCG
CCCGCGCGTACGGCGTGCCGCTGATGTTCGGGGTGGACGACCACGGCATCCTGCAAGTCACGCACGAGCA
AGGCAAGTTC**TTCAAG**GACGCCGACAAGGGCCTCATCGCCGAC**ATGAA**GGCGCGCGGCCTGATGTTCTGG
GCC**GGTAC**CTTGAAACACCGCTATCCCTTCCACGACCGCACCGGCGACCCCATCCTCTACTTCGC**CAAGA**
AGGGC**TGGTAC**ATCCGCACGTCGCAGGTGGCGGGCG**AGATG**CTGGCGCAGAACGAA**AAGAT**CAACTGGGT
GCCGGGCAACA**TCAAG**CACGGGCGCTTCGGCAACTGGCTGGAGGGCAACGTGGACTGG**GCCAT**CTCCCGC
GAACGC**TACTG**GGGCACGCCG**CTGCC**CTTCTGGCAGTCCGAGAGCGGCCAGCTGCGCGTGATCGGCAGCG
TGGCGGAACTCTCCGAGCTGGCGGGGCGCGACCTGAGCGACCTCGACCTTCACCGGCCTTACATCGACGA
CATCACCTTTACGCTCGACGGTGAGGAGTACCGCCGCGTGCCCGAGGTGCTCGACGTGTGGTTCGACTCG
GGGTCGATGCCCTACGCGCAGTGGGG**ACTGC**TGCTGAACGAACAGGGCGAAGCGGTGCGCGGCGCCGAGC
AATT**TGCCA**AGCACTATCCCGCCGACTACATCTGCGAGGCGATCGACCAGACGCGCGGGTGGTTCTACTC
GCTGCAC**GCCAT**CTCGACGATGCTCTACGACCAGCCGGCGTA**CAAGA**ACGTCAT**CTGCC**TGGGGCACATC
GTGGACGAGAAGGGCCTG**AAGATG**TC**CAAGA**GCAAGGGCAACGTGGTGGCGCCG**CTGCC**GCTCTTCGACC
AGTACGGCGCGGACTCGGTGCGC**TGGTAC**ATGTTCATGGCGTCGGACCCCGGCGACCAGAAGCGCTTTTC
CGAGCGTCTGGTCGCCGAGGCGCAGCGCAGCTACGTGAACACGCTGTGGAACGTCTATTCGTTCTTCGTG
CTGTACGCGAACCTCGACCAACCTGACCTCGCCGCTGCTCCCGCCGTGGCCGAGCGGCCCG**AGATG**GACC
GCTGGCTGCTTGCCCGCCTGGAAGAAACGGTGCGCGACGTGACGGCGGCGCTCGAAAGCTATGACGCCCG
CAGTGGGGGCCGCGCCCTGGAAGGGTTCGTGGACGACCTGAGCAAC**TGGTAC**GTGCGCCGCAGTCGCAGC
CGCTTCTGGGGTGAGGGCGGCACCGTGGACACCGCCGCCTACGCCACGCTGCACGAGGCGCTGCTGGTGG
TGTCGCAACTGACCGCGCCGTTCACACCTTTCCTGGCCGACGCGCTCTACCGCAACCTGTCGGGCGAGGA
GTCGTCGGTGCACCTGACGCCGTGGCCGACCGTCCGCGCCGAGCGGCTCGACCGCAAGCTGACCGCCGAC
ATGGCCGCCGT**GATGAA**AGTCGTGGAACTGGGCCGCGCCGTGCGGGGGGCGCACAACC**TCAAGA**CCCGGC
AGCCGCTCGCCGGGGTGCA**GGTAC**GCGCCGCCTCGCCCGAAGCGCTGGACGCGCTGAAGCGCTCGCAGAC
GCAGATCATGGAAGAACTGAACGTCAAAGCCGTGACCTTCCTAGAAGGCGACACCGACCTCGTGCAGTAC
AGCCTGCGCCCGAAC**CTGCC**GGTGGTGGGCAAGCAGTACGGCAAGCAGCTTCCGGTGCTGAAAAAGGCCC
TGACAGAGGCGGACGCCCGCGCCGTCGCCACGGCGGTGCAGGCCGGTCAGGGCTTCAGCGTGCAGGCCGA
CGGCGTGACCTTCGACCTGACGCCGGGGAGCGTGCTGGTGGACGCCAAGGCGCCCGAAGGTGTGGCGG**CT**
GCCGAGGACGCTGGGTATCTGGTGGCCTTCGATACCGCCCTGACCCCTGAAC**TGGTAC**GCGAGGGGCTGG
CCCGTGATCTCGTTCGC**GCCATTCA**GGAGGCCCGCAAGGCGGCGGGCTTCGAGGTGCAGGACCGGATTGC
CCTCGCGCTGGAACTGGATGGCGAGGCGCTGGAAGCGGCGCAGGCGTGGCAGGACTTCATTGCCGGTGAG
GTGCTGGCGGAGCAGGTGGCCTACGGCTCCGGCGAAGGCTTCCGCGCCGAGGTGGAAGGCGGAGCGGTGA
CGC**TCAAGA**AGCTTTGA

65 3234 **2 observed, 2.15 ± 0.4* expected**

Deinococcus radiodurans R1 chromosome 1, complete sequence GenBank: CP015081.1: c1620566-1619541 tryptophane—tRNA ligase

ATGACCACCCCGACCC**CTGCC**GCTACAC**CTGCC**CGCCCCCGCGTCCTGACCGGGGACCGGCCCACCGGGG
CGCTGCACCTCGGGCACCTCGCCGGGTCGCTGCAAAACCGGGTGCGCTTG**CAAGA**CGAGGCCGAGCTGTT
CGTGCTGCTTGCCGACGTGCAGGCGCTCACCGACCACTTCGACCGGCCCGAGCAGGTGCGCGAGAACGTG
CTGGCGGTGGCGCTCGATTACCTCGCCGCCGGGCTCGACCCGCAGAAGACGACCTGCGTGGTGCAGTCGG
CGGTGCCCGAACTCGCCGAGCTGACGGTCTATTTCCTCAATCTGGTCACGGTGTCGCACCTGCGCCAGAA
CCCCACCG**TCAAG**GCCGAAATCGCGCAAAAGGGCTACGGCGAGCGGGTTC**CTGCC**GGGTTTTTCGTGTAT
CCGGTGTCGCAGGCCGCCGACATCGCCGCGTTCGGGGCCACGCTGGTGCCGGTGGGCGACGACCAGTTGC
CGATGCTCGAACAGACCCGCGAAATCGTGCGGCGC**TTCAA**TGCGCTGTATGCCCCGGTGCTCGCCGAGCC

GCAGGCGCAGCTCAGCCGGGTGCCGCGTCTGCCGGGGCTCGACGGGCAGGCCAAGATGAGCAAGTCGCTC
GGCAACGCGATTGCGCTCGGCGACTCTGCCGACGAGGTGGCGCGCAAGGTCATGGGCATGTACACCGACC
CTGGCCACCTGCGGGCAAGTGATCCCGGACGGGTGGAGGGCAATCCGGTCTTCACCTTCCTCGACGCCTT
CGACCCCGATCCGGCGCGGGTGCAGGCGTTGAAAGACCAGTACCGCGCGGGCGGCCTCGGTGACGTGAAG
GTCAAAAAGCACCTGATTGACGTGCTCAACGGGGTGCTGGCTCCCATCCGCACGCGGCGCGCCGAGTATG
AGCGCGACCCCGACGCCGTGCTCCGGTTCGTCACGGAGGGCACGGCGCGGGGCCGGGAGGTGGCGGCGCA
GACGCTCGGGCAGGTGCGGCGGGCGATGCGGTTGTTTGGGCACTGA

12 1022 **1.2 observed, 2.15 ± 0.75* expected**

Deinococcus radiodurans R1 chromosome 1, complete sequence GenBank: CP015081.1: c1446067-1444568 proline—tRNA ligase

ATGACGAAAGATGGCGGCAAGAAAGACAACCAGGGCCAGGACAAGAAGGCGCAGCAGTACGGCGTCACCC
CCCAGAGCGTAGATTTCAACGACTGGTACAACGAAGTGGTCAAAAAGGCCGACCTCGCCGACAACTCGCC
GGTGGCGGGCGCGATGGTCGTGCGGCCCTACGGCTCGGCGCTGTGGGAAAACATTCAGCGCTGGCTCGAC
GACCGCTTCAAGGCGAGCGGGCACGAATCGCTGATCTTCCCGACCCTCATTCCCATGAACTTCATCATGA
AGGAGGCCGACCACGTCGAGGGCTTCGCGCCCGAGCTGTTCACGGTGAACCAAGATCGGCACCGAGGAACT
GGCCGAGCCCTACGTGATGCGCCCGACCTCCGAGACGATCATCGGGCACATGTGGAGCGGCTGGCTCAAC
TCCTACCGCGACCTGCCCTTCCTGCACTACCAGTGGGGCAGCGTGTTTCGCGCCGAGCTGCGGACCAAGG
CGTTTCTCCGCACCTCCGAGTTTTTCTGGCACGAGGGCCACACCGCCCACGCCGACGAGGCCGAGGCCCG
CGCCGAAGTGCGCCAGCAACTCGACCTGTACCACGAGTTCTGCCGCGACGTGCTGGCCCTGCCGGTGGTG
CGCGGCGAAAAAACCGCCTCCGAGCGCTTTGCCGGGGCGGTCGCCACCTACTCCATCGAGGGCATGATGC
GCGACGGCAAGGCGCTGCAATCGGGCACCTCGCACTACCTCGGCCAGAACTTCAGCCGGGCCTTCGATGT
GAAGTACCAGACCCGCGAACAGAAAGAGGAATTCGCGCACACGACCTCCTGGGCCATCTCCAGCCGCATC
ATCGGGGCCATCATCATGACCCACGGCGACGACTCCGGGCTGATGATGCCGCCCCGCATCGCGCCCATTC
AGGTGGTTGTGATTCCGGTGGGCCGCAAGGACAACTTCGACCAGATGGTGCAGGAAGGCGAGAAACTGGC
CGCCGAGCTGCGCGCCCAGGGCCTGCGCGTCAAGGTGGACGGGCGCGACGGCGTGACCAACGGTTTCAAG
TACAACGACTGGGAACTCAAGGGCGTGCCGGTGCGCATCGAACTCGGCCCGCGCGATCTGGAAAGCGGTG
TGCTGGTCGTCAAGAACCGCCACAGCGAGGACAAAGAAACCCTGCCGCGCGCCGAAGCGGTCAGCGGCAT
GAGCGCCCGTCTGGACACCATCCACGACTTCCTGATGAAGCGGGCGACGGATTTCCTGCTGGCGAACACC
GCCGAAGTGGACAGCTACGACGCCTTCCAGCGCGAAATCGAGGCCGGTCACTGGGTCCGCGCTTACCACT
GCGGCGAGCCGGCGTGCGAAAAGAGCATCAAGGAAGACACCAAGGCGACCGCCCGCAATGTGCCCTTCGA
CGACGCCGAGTTCTTTGCCGAGCGCGGCGAGGGTCAGTGCGTGAAGTGCGGCCAGCCGAGCGCGTATGGC
AAGCGGGTGCTGTTCGGGCGTCAGTATTGA

38 1496 **2.5 observed, 2.15 ± 0.6* expected**

Deinococcus radiodurans R1 chromosome 1, complete sequence GenBank: CP015081.1: c2237914-2236463 glutamate—tRNA ligase

ATGTCTGCTCCCAGCTCCCCGCGCGTGACCCGCATTGCCCCCAGCCCCACCGGCGACCCGCACGTCGGCA
CGGCGTACATCGGGCTGTTCAACCACACCCTCGCCCGCCAGAGCGGAGGCCGCTTCATCCTGCGGGTGGA
GGACACCGACCGCAACCGCTACGTCCCCGACAGCGAAAAACGCATTTTCCAGATGATGCAGTGGCTGAAC
CTCACCCCCGACGAAAGCCCGCTGCAAGGCGGTCCCAACGGGCCTTACCGCCAGTCCGAGCGCTTCGACC
TCTACGGCGACTACGCCCGTCAGCTCGTGCAGAGCGGGCACGCCTACTACGCCTTCGAAACGTCCGATGA
ACTCGCCGCCCTGCGTGAGGAAGCGCAGAAGGCCGGGCACGTCATCGCCATTCCCAGCCGTGACTTGGGC
GCGGCGCAGGCGCAGGCGCGGGTGGACGCGGGCGAGCCGGCGGTCATTCGCCTGAAGGTGGACCGCGACG
GCGAAACGGTGGTCAACGACCTGCTGCGCGACCCCATCCACTTTGCCAACAAGGAAATTGACGACAAGGT
GCTGCTCAAGGCCGACGGCTTTCCCACCTACCACCTCGCCAACGTGGTGGACGACCGCTTAATGCAAGTC
ACCCACGTGGTCCGCGCCGAGGAGTGGATTACCTCCACGCCTATTCACGTACTGCTTTACCGCGCCTTCG
GCTGGCCGGAGCCCGTGTTCGCGCACATGCCGCTGCTGCGCAACGCCGACAAATCCAAGATTTCCAAGCG
CAAGAATCCCACCAGCGTGGAGTGGTACCAGAACCAGGGTTTCCTCCCCGAAGCGATGCTCAACTTTCTG
GCGACGATGGGCTGGACGCACCCGGACGGCCAGGAAATCTTCGACCTCGCCGAGTTCGAGCGGGTCTTTC
GTCTCGAGGACGTGACGCTCGGCGGCCCGGTCTTCGACCTCGCCAAGCTGCGCTGGTACAACGGCAAGTA
CCTGCGCGAAGTACTGAGCGAGGACGACGTGGCGCGGCGACTGCACGCCTTCCTGATGCAGAACAAAGTC
ACGCTGCCCAGCGTAGACGGCCCGCAGGACCCTTACTTCCGCGCCGTCACCCACCTGATGATTCCCCGCC
TGGAAGTCTTCGCCGACTTCATGGACAAGACGCTCTACTTCTGGTCCGAGGATTACCCGGTGAACGAGAA
GGCGCAGAAAGCCATCGACGCGGGCAAAGAGTTGCTGCCTGAGCTGGCCGCCCGCCTCAAGAACCTGCCG
ACCTTCGATGCCGCGAGCATCAAGGAGATGTTCCACGCCTACGCCGAGGAAAAGGGCCTGAAGATGGGTA
AAGTGATGCCCCCCATCCGCGCCGCCGTCGCGGGCACGATGGAAAGCCCCGACCTGCCGGAGATGCTTGA
AGCGCTGGGCCGCGAGCGCGTGCTGGCGCGGGTGGAAAAAGCCGCCCGCTGA

38 1448 **2.6 observed, 2.15 ± 0.6* expected**

Deinococcus radiodurans R1 chromosome 1, complete sequence GenBank: CP015081.1: c2354014-2352473 lysine—tRNA ligase

CTGCACGAACAGACCATCGCCCGCC**TGAAT**AACCTGGACGCGCAGGTTGACGCCGGCTTCGAGGCCTATC
CCTACAGTTACCCCCAGACCCACCACGCCCGCGACGTGTTCGCCGCTCACC**CTGCCA**GAGAAGAGGGCAC
AGGGGAAGGGGGAACGCTGGAACCCGGCCAGAAGTGGGAAGAAGAAAGCTACTCGCTCGCGGGCCGCGTG
ACCCTGATGCGCCACATGGGCAAGGCCGCCTTTGCCGACCTGAGCGACGAGGACGGCAAAATCCAGCTGT
TTTTCTCCAAGCAGGACACCGCCGGCTTCGACGCCAC**CAAGA**AAATCGACCTCGGTGACATTATCGGCGT
GAAGGGTCACCCCTTCGTGAC**CAAGA**CCGGGCAACTGACCCTGCATGTGACCGAGTGGCAGCCGCTCG**TC**
AAGAGCCTGCACCCG**CTGCC**GAGCAAGTTTCACGGCCTG**CAAGA**CGAGGA**ACTGC**GCGCCCGGCGCCGCT
ACGTAGACCTG**ATGGT**GACCGAGGGCGCCCGCGAGAAGTTCCAGGCCCGCAGCCGCATGATTC**GGTAC**AT
CCGCAACGAACTGGACGAACGCGGCTTCATGGAAGTGGAGGGGCCCACCCTTCAGGTCACGGCGGGCGGC
GCCGAGGCCCGGCCCTTCATGACGCACCACAACGCGCTCTCGTACGAT**TTCAAG**TTGCGCATCAGCCTGG
AACTGTACC**TCAAG**CGGCTGCTCGTCGGCGGCTTCGAGCGGGTGTACGAGATCGGGCGGGTCTACCGCAA
CGAGGGCATCGACCGCACCCACAACCCCGA**ATTCA**CCATGCTGGAACT**GTACTG**GGCCTACGCGGACTAC
AGCGACATCGCCAAGTTGGTCGAAGACCTGCTGAGCGGGCTGGCAAAGGAAGTGCACGGCTCTTATCAGT
TCGAGTACCAGGG**CAAGA**CGCTGGACTTTACCCCGCCGTTTGCCCGTGTGGATTACCTCGGCGGCCTGCG
CGAGCACGTGCCGGGCCTCGACTTCGACCCGCTGGACCTAGACCGGCTGCGCGCTTTCTGTGACGAGCGC
TTTCCGCAGTGGAAGGGCGTGCCGAGCTACAAGCTGCTGGACAAACTGTTCGGTGAGTTCGTGGAGCCGC
TGCTGAGCAATCCGACGTTCGTGATGGACCACCCCGCCGTGATTTCGCCGCTCGC**CAAGA**AGCACCGTTC
CCGCCCCGAGGCCGTCACCGAGCGTTTCGAGGTGTTCTGCTCGGGCTTCGAACTGGC**GAATG**CGTTCTCC
GAACTCAACGACGCCTTCGACCAGCGCGAACGCTTTGAGGCGCAG**ACTGCC**CGCCAGGCCGCCGGAGACG
ACGAGGCGCACCCGCAGGACGAGGACTTCCTGCTCGCGCTGGAATACGGGATGCCGCCCGCCGGGGGCCT
GGGCATCGGCATAGACCGGCTGGCGATGCTGCTCACCGACTCGGATTCCATCCGCGACGTGCTGCTGTTC
CCGCTGCTGCGGCCCGAAGGCGGCGAGGCGGAAGAAGCTGACGACACCGCGCAGGAAAACACCGCCGGCTGA

23 1538 **1.5 observed, 2.15 ± 0.6* expected**

Deinococcus radiodurans R1 chromosome 1, complete sequence GenBank: CP015081.1: 2574688-2577429 valine—tRNA ligase

ATGACCGACCCCCACACCACCACCCTCCCCACGCAGTTCGACCCCACCGGCATCGAGCCGA**AATGG**GCCG
CCCGCTGGCGCAGTGAACCGTTCCGCGCTGACGCCACCAGCGGCAAAGACCCCTTTACCATCGTGATTCC
GCCGCCCAACGTGACCGGCAACCTGCACCTCGGACACGCGCTCGACAACACGCTCATCGACACCCTGATT
CGCTACAAGCGCATGGCGGGCTTCGAGGCGCTGTAC**CTGCC**GGGCATGGACCACGCGGGCATCTCCACGC
AGGTGGTGGTGGAGCGGCAGC**TCAAG**GACGCCGGGACCAGCCGCCACGACCTCGGGCGCGAGGCGTTCCT
TGAAAAAGTCTGGGAGTGGAAGGGCAAATCCGGCGGCATGATTCTCGATCAGCTCACCCGCCTGGGCGTG
AGCGCCGACTGGACCCGCGAGCGCTTCACGATGGACGAGGGCCTCTCGCGCGCCGTCCGCACCCAGTTCG
TGAAGCTCTACCACGACGGCCTCGCCTACCGGGGCGAGCGCATCGTGAACTGGGACCCGGCCTCACAGAC
CACGCTCTCCGAACTGGAAATTGACCGCGAAGTGCGCAAGGG**CAAGATG**TACACCCTGTCGTACAAGCTG
GAAAACTCCGCCGAGCGCGGGAGCAACGGCGAAACTGGCGAAATCCGCATCGCCACCGTGCGCCCCGAAA
CCATCTTCGCCGACCAGGCGATTGCTGTGCATCCCGAGGACGAACGCTTCCGGCATCTGGTGGGCCAGAA
GGCGCG**CATTC**CGCTGACCGACCGCTGGGTGCCCATCATCGCCGACGAAGCGGTGG**AGATG**GAATTCGGC
GTCGGGGCACTGAAAATCACGCCCGCGCACGACCCCACCGACTTTGAAGTGGGCGAGCGGCACGGCCTGG
AGCGCCCCAGCGTGATTGATCTCGACGGCAACCTCACCCGTGACGAGCTGGTGCCCGCCGAGTTTCAGGG
TCTGGAACGCTTCGCCCGCGCGCAAAGCGGTGGTGAAGGCGCTGGAAGAATCCGGCGATCTGCTGGAGCAA
AAGGACCACGACACC**GCCAT**CGGCCTCTCCGAGCGCACCAAAGTGCCGGTGGAGCCCATCATTTCCGAGC
AGTGGTTCGTG**AAGATGAA**ACCCTTTGCCGACCAGGTCTTGGAAGGACTGGATAAGGGCGAGA**TCAAG**CT
GGTGCCCGAGCGCTACACGAAGGTCAACCGTGACTGGCTGGAAAACATCCGCGATTGGAACATTTCCCGG
CAGTTGTGGTGGGGCCACCAGATTCCGGCG**TGGTAC**GACAAAGAGGGCAACATCTACGTGCCCGACCCGG
AAAACCCCGAACTCG**ACTGC**GACCAGGACCCCCGCTACGCGCACCTCAATCTGCGCCGCGATCCCGACGT
GTTCGACACCTGGTTTTCGAGCAACCTGTGGCCCTTTTCCACCCTCGGCTGGCCTGACACCGACAGTGAG
GACTTCCGCAAGTTCTACCCGACGCAGGTGCTGGTGACCGGCTACGACATCCTCTTTTTCTGGGTGGCGC
GCATGC**AGATG**GCGGGCTACGGCCTGACCGGACAAGCACCCTTTTCCACCGTGATGCTGCACGGCCTGTA
TCTGGACGCCAAGGGCCAG**AAGATG**TC**CAAGA**GCAAGGGCAACGGCATCGACCCGCTGGAACTGTTTGGG
CAGTACGGGGTGGACGC**CTGCC**GCTTCGCCTTCACGTTCCTCTCGACCGGCGGGCAGGACA**TCAAG**CACG
ACGCCCGGCGCTTCGAGCAGGGCCGCAACTTCGCCAACAAGCTCTGGAACGCCACCCGCTTTGCGCTGAT
GCGTCTGGGCGAAGCCCGAATCGAGGGGAGCGACGACCTCTCCGCCTACGTCCGCGCCGCCGTGACGCTG
CCCGATGGCGTGCTGCTGCGGAGCAAGGACGTGTTGGCACAGC**TCAAG**GAGCGCGACGACCTCACCCTCG
CGGACCGCTGGATTATCAGCCGCC**TGAATG**ACGTGACCGCCGAAGCCTCCGCGCAGCTCGATGCCTTCGA
CATCGGCGCC**GCCAT**CCGCACGCT**GTACT**CGTTCACCTGGGACGAGTTCTGCGAC**TGGTAC**ATCGAGGCC
GCCAAGCCCGAACTCGCCAGCGGCAACCTCGGCACCATGGCGACGCTGAAGGTGGTGCTGGAGCACGTGC
TCAAGCTGCTGCACCCCTTCATGCCGTTCATCACCTCCGAGCTGTACGCCGCGCTGGGGCACCGCCGCCA
GATTGCCGTGCACAGCTGGCCGCAGCCCGACGCCGCTCTGCACGACGCGGAAGCCACCAAAGCTTTCGAC

GCGCTGCGCTCGGCGGTGGACAGTGCTCGCAGCC**TCAAGA**GCGAACTGGGCCTCTCGCCGCAGGACCGCC
TGAACGTGGCGGTGGACGGCGACCTCGCTGACGTGGTGCGACAGAACGCCCGCGTGGTGGAGGGCATCGC
CAGGGTGAACCTCGTGCCCGCTCTGGAAGGCCGCACGCTCTCGCAGGTGGCCCCCGGCGTGACCATCCTC
GCGCCGCTGGAAGGCACGGTGGACATCGCCGACTGGGTGAAAAAGCAGCAAAAACGCCTCGCCGAACTCG
ACAAGCAGA**TCAAG**CAGGCGCAGGGCAAGCTGAACAACGAGGGCTTCGTGGCCCGCGCCCCCGCCGAGGT
GATCGAGGAGGAGAAGCGCCGGGTGGCCGACTTCGGGGCGCAGAAGGAGCGGTTGGAAGGAGTGTTGGCT
CAGTTGGGGTGA

34 2738 **1.2 observed, 2.15 ± 0.45* expected**

Deinococcus radiodurans R1 chromosome 1, complete sequence GenBank: CP015081.1: c1437593-1436316 serine--tRNA ligase

ATGCTGGACCTGAAATTTATCCGTGAGAACCCCGACGCCGTGCGCGAG**GCCATTC**GCG**TCAAGA**ACGTGG
CCCTTGACCTG**GATGA**CCTGTTGCAGCGTGACCGCGACCTCGTGGCGC**TCAAG**CAGCGCGTGGAA**GCCAT**
GCAGACCGAGCGCAA**TGCCA**ACGCCAAGCTGGTGCCCAAGGCCAGCCCCGAGGACCGCCCCGGCCTG**ATT**
CAAAAGGGCAAGGACCTGAGCGAAGACC**TCAAG**GCGCTCGAGCCCCA**ACTGC**GCGAGCAGGAAGACGCCC
TGAAGCA**ACTGC**TGCTGCGCGTGCCCAA**CATTC**CG**CTGCC**GGGCGTGCCCGTCGGCAAGGACGAAGACGA
CAACGTGGA**ACTGC**GCCGCGAAGGCGAG**CTGCC**TGGGTTCGACTTCACGCCGCTCGACCAGGTCGAGATT
CTGGAAAAGCAGGGCTGGGCCGACTTCGAGCGTGTCGCCCGCGTCTCGGGCAGCCGCAGCTACCTGC**TCA**
AGGGCGACGCGGCGCTGCTGGA**AATGG**CGGTGCTGATGTTCGCGCTCGATTTCCTGTCGCAGCGCGGCTT
TACTCCACTTTCGACCACGGCGCTCGTCCGCCGCGAAACGCTGGTCAACTCGG**GCCATT**TCCCCGGCGAC
GAGGAATCGGTCTACAAGCTTGAAGGC**GATGA****ACTGC**TGCTCGCTGGAACCGCCGAGGTGCCGATCAATA
GCCTCTACGCGGGCGAGCAGCTCAGCGCCGACGAGTTGCCGCTGACCTTCGCGGGTTTCAGCGCCGCCTT
CCGCCGCGAGGCCGGGAGCGCCGGGCGCGACGTGCGCGGGCTGATTCGCGTCCACGAGTTCCGCAAGGTC
GAGCAGTACGTCCTGTGCCGCGCCGACCAGGAAGAGGGCCTGA**AATGGT**TCGAGCGCCTGCTGAGCAACG
CCGAGGGGCTGTTGCAGGCACTGGAG**CTGCC**CTACCGCGT**CATTCA**GA**ACTGC**ACTGGCGACATGGGCGC
GGGCAAGGTGCTGATGTACGACATCGAAACCTGGGTGCCGAGCGAGCAGAAGTACCGCGAAACCCACTCC
TGCTCGTACCTGGGCGACTGGCAGGCCCGCCGCACCGGCCTGCGCTACCGCGACGAGCACGGCAAGCTGC
TCTACGCCCACACCCTCAACAACACCGGCATCGCCAGCCCGCG**CATTC**TGGTGCCGCTGCTCGAAAACCA
CCAGCAGGCTGACGGCACCGTGCGCGTGCCCGCCGCGCTGCGCCCTTACCTCGGCGGCAGGGAAGTGCTG
GGCCAGCCGGTGCGCTGA

32 1274 **2.5 observed, 2.15 ± 0.65* expected**

Observed mean proximity: 1.8 Expected mean proximity: 2.15 ± 0.55* (* 90%-confidence interval calculated from the mean sample size equal to 1913). Data from https://www.ncbi.nlm.nih.gov/nuccore/CP021507.1?report=genbank

AL-pentamer proximity for CRISPR Cas proteins

Mucilaginibacter paludis DSM 18603 chromosome, whole genome shotgun sequence GenBank: CM001403.1: 2879349-2879678
CRISPR associated protein Cas2

ATGCGTTTAA**ATGAAT**ACCGGATTTTGTGGATATTAGTATTTTTTGAC**CTGCC**GACAGAAACAAAAAAAG
ACCGGCAAATCTACAGTAGGTTTCGTAAGGATATT**ATGAA**GGATGGATT**TGGTA**TGTTCCAATTCTCCAT
CTACCTGCGGCATTGCAGCAGTCGTGAAAACGCTGATGTACACATCAAACGTGTGAAGAAAATACTCCCA
CCTAAAGGTCACGTCGGTATCATGA**CCATT**ACCGATAAGCAGTTCG**GAATG**ATGGAATTGTTCT**ATGGTA**
AGGAAGAGAAGGAGCTTCCAGACACCCCACAGCAATTGGAATTGTTTTAG

9 326 **2.8**

Mucilaginibacter paludis DSM 18603 chromosome, whole genome shotgun sequence GenBank: CM001403.1: c2880706-2879693
CRISPR associated protein Cas1

ATGATAAAACGAACGCTTCACTTCAGTAATCCGGCTTACCTGAGCTTAAAGGAAAGCCAGT**TGGTA**ATTG
ATCTTCCTCACCTTAAAATTTTAGGGGAAAAGGAAAGTAAAAAGTC**GGTAC**CTATTG**AAGAT**ATAGGAAT
TGTT**GTACTG**GATCACCAGCAGATCACCATCACCCATGGCTGCATTGCAGCATTATTATACAACAATTCC
GCGATCATCACCTGCGATCATACACATCACCCCACCGGCATGATGCTACCAATTGACGGACATGATACGC
AAAGCGAGCGCTTCA**GGTAC**CAAATCGATGCTTCACAGCCACTTAAGAAGCAGCTTTGGCAACAAACTAT
CCAAGCCAAAATATTAAACCAAGCGGCGGTTTTGGCAGATCG**TGGTA**TTCCCCACGATAATATGCTTCAC
TGGGCTAAGTCTGTACGTTCAGGCGATCCGGATAACTACGAAGGACGTGCCGCCGCTTAT**TACTGGAAGA**
ATGTATTCCCTAAAAAAGTGGAATTCTTTCGTGGCCGTGAGGGTGACCCGCCAAACAATT**TACTGAAT**TA
TGGTTA**TGCCAT**ATTACGGGCTATTGTAGCGCGCGGGC**TGGTA**TGTTCCGGCCTGATACCTACATTAGGT
ATACATCACAGGAATAAATATAACGCCTATTGCTTGGCT**GATGA**CATTATGGAGCCTTACCGCCCCTATG
TTGATTTGATCGTTCTA**AAGAT**CATCGAT**AATGG**CGAGAATTTTTTAGAGTTAGGCAATTCTATAAAATC
TCAGTTATTAGGCAT**TGCCA**CCGTTGACGTACAATTTGAAAGGAATAGGAGTCCGTT**AATGGT**TGGTGTG
CAAAGCAC**TACTGC**TTCTTTAGCCAGGTGTTACGAAGGAAAAAACAGGAAAATAAACTACCCTATC**ATGA**
AGAATTTTAATCCTAAAAGG**GCCAT**AATCCCTAAGCCGGACGAAAAT**TTCAA**T**GATGA**GGATACAGAAAA
TACTAT**TGAAT**ACAATATTGGC**GATGAA**GAATAG

31 1010 **3.1**

Mucilaginibacter paludis DSM 18603 chromosome, whole genome shotgun sequence GenBank: CM001403.1: 7594207-7594989
CRISPR associated protein Cas6

GTGCGATTTAAATTAACATTGTTGCCTACGGGCAGTAAAACGACCGTT**CCATTCAA**TTATCAGTATGCCC
TTGCGGCGGTAATTTTTAAAAAGGTTGCCG**CTGCC**GACGAGCAATACGCCAACTTTTTACATCAGCAAGG
TTATGCCCTGAACAATTACTCCAAACATTTTAAGTTTTTTACCTTCTCTAACCTGGAAGGTAGGTTTACG
CCACAGCAAAATGCCCTGTTGCTGCAAGGTAATCAAACCGGGTTTGTGCTTTG**CTGCCA**CATGCCCGAAT
TTGCTGCGCATTTAATAACCGGTGTTTTTACCGATCAGTTGATATCAATAGGCGGGAGCGGGGCCGTGGG
CCGGTTTAGGGTTGAGCAAATTGTATCGCTCCCGG**CTGCC**TTTAAG**GATGATGA**CGAAATACACACGATG
CAGTTTAAACCGCTTTCGCCCTTGGTTGTAGGCCGGACCAACGCTAAAGGCAAT**GATGA**TTATTTATCGC
CAG**AAGATGA**GGATTTTATACCCCTGCTACATATTAACCTGGCCGATAAGCTTGCCGTTGCTTATAATGA
TAGTTATAGCGGGCGAAT**TGGTATCAAG**GTAAAGC**ATGAA**CCCGGAAAGCTAAAATCTCGCCTGGTGACG
ATTAAGGAAGGGACAGCGGCCGAAACTAA**GGTAC**GTGGCTTTTTCGGGTTTGCCCTGG**AGATGACTGC**GC
CTGGCAGGGTGATCAATTTTGTGTTGGACGTGGGGCTGGGCGG**GATGAAT**AGTATGGGGTTTGGTTGTGT
GGAGGTGAAATAA

24 779 **3.1**

Streptomyces virginiae strain IBL14 Cas2 gene, complete cds GenBank: KY176009.1

GTGTATGTAGTCGTCGTCTACGACACCCACGCCAAACGAAACGCACGGATCCTGCGTAC**CTGCC**GCAAGT
ACCTCCACCACGTCCAGCGCAGCGTCTTCGAAGGCCACCTCAGCGACGCCCAACTCCGACACT**TTCAAG**C
TGCAGTCG**AAGATG**CCCTCGACTTGGAGTACGACAACGTCCTCGTCTACACGTTCCCAGCCGGCACCG**TG**
CCACAACGCCT**TGAATGG**GGAGCAGTCGAACCCGCCCCCAGTGACATCCTGTGA

9 260 **3.5**

Legionella pneumophila strain L15-118 CRISPR-associated endoribonuclease Cas2 (cas2) GenBank: MF440347.1: 5115-5414

ATGCAAAT**AAGAT**TATACAAGGAATACATAGTTTCCTACGACATCAGGGACAACAGCATCCGACGTACAA
TTTACGAAAGGCTTAAAGACATTGGCTTAACCCCCG**TTCAA**AAATCAGTATTTTGGGGATATTTAAACGA
ACCTGAAAA**AAGAT**ATTTAACGAACTTTCTAAAAAAAGAACT**TGCCA**AA**CAAGAT**ACCGCTTTTATTACA
CCTTGCAAGCTGGC**TCAAGA**ATACGAAGTAAACCTCATTAATCTTGAACTG**GATGAA**CTTAGGAAACCGG
ATGGACACATCATCGTATAA

10 296 **3.4**

Tannerella forsythia 3313 DNA, complete genome GenBank: AP013044.1: 2465356-2465619 CRISPR-associated protein Cas2

ATGTATGTGATTCTGGTCTATG**ACTGC**GGCGAGAAACGGGTTGGGAAAATGC**TCAAG**CTCTGTCGGAAAT
ATC**TGAAT**TGGATACAAAATTCGGTTTTTGAAGGCGAAATTACAGAAGTTAAATTAAAAGAGTTAACTTT
GAAGGCTGAGAAAATA**ATGAA**CAGC**GATGAA**GACTCTCTGATTGTTTT**TTCAAG**TCGA**CAAGA**AA**AATGG**
TTGGATAAGCAAATCTT**TGGTA**AAGAGCGGAGTAGTGTCGATAACTTCTTGTAA

12 260 **4.6**

Nostoc sp. 'Peltigera membranacea cyanobiont' N6 chromosome, complete genome GenBank: CP026681.1: 5862085-5862366 CRISPR-associated endoribonuclease Cas2

ATGTTTTTATATGTGATTGCTTATGA**CATTC**CTTGTGATAAACGACGTAAAAAAGTAGCTGATTTACTTG
AAGGCTATGGACAACGGG**TTCAA**TATTCTGTCTTTGAGTGTCAGTTGACTACCGATAAATA**TCAAGAT**TT
ACGTCGTCGGTTAAGAAAAAAGTTGAAGTTAACTG**AAGAT**AGTCTACGATTTTACCCTTTGTCACGACAT
ACCCTTTC**TCAAG**TAGAATCTTGGGGTGTGGG**GCCAT**CTGTGATTGAACCGCCTAGTTCGATAATCATTTAA

8 278 **2.9**

Saccharolobus solfataricus strain SARC-B chromosome, complete genome GenBank: CP011055.2: 2127278-2128213 type I-A CRISPR-associated protein Cas7/Csa2

ATGTGGATATCCTTCTCCGTTAGGTATT**TGGTA**AACGTAG**AAGAT**CTAAATAACGTAGAGTCTGCAGGGA
ACTACGTAAGGCATAGGAGAGCT**CCATT**AGTTTTTAAGGATAAAGACTCCTACACGGTAACATATG**TGCC**
AGCAGTTAGTGGAGAAATGATAGCACATGGGTATCAA**ATGAAT**CTAGTCGAACTAGCGC**TTCAA**AGAAAT
CTACCAGTAGATAGTTTAGCTAAGCAGGGAATATTGATAAAGAGGGGTTCT**GATGA**TAAGGTTC**ATGAA**G
GCACTAAATGCACT**GATGAA**AAAGGGTCGGATTACGAACTCTGCGTCATTAATGAGGATATAGTCGAGGA
TGTTGCTGG**ATTCATGAAT**CCAAACAAGCTAGTTAAGAGAAC**TTCAA**ATGTAGCCTTTAGTTAC**ATGGT**T
CCTGCAAT**AGATG**CAGTTAAAGC**TTCAA**CAATATCTTCCCAATTTCACGTTAGGTATGCTAATAAAGAAC
T**AATGG**ACAAATA**CAAGA**ACGAGAA**CATTCAA**TCCCTATATAATATCGAAACAGCGAGCGCATCGTAT**GT**
ACTTACGGGTTACTTGAACGTTAACAGTGTAGGAAAGACACAAAACTATCCGGTAAAAGAAGTTGATAAG
AAAAAGGATAGGGAAAAGGCTGCATT**AGATG**CCTTAATGTTAACTTTAACGCAATTCCTATTTGGCGCAA
AGTTGACTAGATTTAAGCCCATAGTGGAGATAGAGGCCTTATTTGTTAGCGCTTCAGAGAAGCCT**TTCAA**
TCTACCACCCGTAACTGGAGACATTAAGAAATACATAGACCTTGTTAACTCTACAACGGATTCCTTCGCT
AAAATTT**TGAAT**ATTAAACGGCCCGTAGTAAA**GTACT**ATCTAAAGGAGGAGAAAGGTAACGTCAATACGC
CAAT**AGATG**CGTTTACGGTAATGTAA

29 932 **3.1**

Saccharolobus solfataricus strain SARC-B chromosome, complete genomeGenBank: CP011055.2: 2120631-2121506 type I-A CRISPR-associated protein Cas4/Csa1

GTGTTCTTTACT**CATTCA**GATATGCTGTTGCT**TTCAA**AGAGGATAAAGAAGTTACCTAAGAACGTG**GATG**
AGGAGCTGAGGGGATGGAATTGGAGCGAACCACCAGTTTACACTAGGTCCCTTTCACAGGTATCAATTTC
CGA**AATGGT**T**TACTG**TA**GTACT**CTGAGGAACGTTTACCTTAAGGTAAAGGGCTTTAGGGGAGAAATAGGT
AGGCAAATAC**TTCAAG**GTTCACTTATCCACACGATTTACGCTATTGGCATTGAAGCAATCAAACGTTTTA
TTT**ATTCAAG**GGAAAGCATTGATGGGA**GTACT**CTGAGAACGCT**AATGGGAGATGA**GTTTTACTCCCTACT
TAAGGACTTAAGGGAGGAAGAGGGGATTTACGCAAAGGTCTTATGGGATCACATTACGAACATTT**ATTCA**
GCTGAACTGGATAGGGTTAGGAGTAAGTTCACTAACTTAACTAGGGATTCCTTGGTCTCCCAAGTTGTGC
CGTTTTACGTTGAGTTTCCAGTTG**ATGGT**TCCCTTCTGGGCTTGACCAATTTAAGGGTCGACGCCTTTAT
CCCTCACCTTCCCCTAATAGCGGAA**ATGAA**GACTGGGAAATACAGATACACTCACGAGTTGTCCCTTGCA
GGATACGCTTTGGCAATTGAGAGCCAGTACGAGATAC**CCATT**GATTTTGGTTACTTGTGTTATGT**TACTG**
TGACTGAAAAGGAGGTTAAGAATA**ACTGC**AAGCTGATTC**CCATT**TCAGATTCCTTGAGGAGTGAGTTCTT
GGACATGAGGGATAAGGC**TCAAGA**CAT**AATGG**ATAAGGGTGTGGATCCTGGGATAGCTAAGGATTGTGAG
AGTGATTGCATGTTTTATAAGGTTTGTCACCCTTAA

27 872 **3.1**

Methanosaeta concilii GP-6, complete genome GenBank: CP002565.1: c2758294-2758007 CRISPR associated protein Cas2

ATGCTGGTCTGGGTGATCTACGACATATCCGAAAACAGATACCGCAGCCGGGTGGCTAAGATCTGCAAGA
ACTACGGCCTGTTTCGGGTGCAGAAGAGCGCATTTCTGGGAGACTTGAACCGGAATGAGTCCGACTCCCT
GACTCTGGAGTGCGAGGCGATTATTGACGAATCGGATTCAGTCTACGTCTTCCCCATGTGCGAGGACTGC
TTTGATAAGATCAAGCTGATCGGAGCAGGGTTCGATCGAGAGCTGGTGAGCGGGATGACCATAACCAAGG
TGTTTTGA

8 284 2.8

Desulfobacula toluolica Tol2 complete genome GenBank: FO203503.1: 1119557-1120546 CRISPR-associated protein, Cas1 family

ATGAAACAATCCTTATATCTTTTTTCAAATAGTACCATCAAAAGAAAAGACAATTCTTTGATGTTTGAAA
TTGACGGAGAAAAGAAATACCGTCCTGTTGAATCAATTTGTGACCTTTTTCTTTTCAATGAACATACTCT
GAATACAAAATTGCTGAATTTTCTGGGGCAACAAAAAATTCCCGTTCATATTTTTAATTATTACGGATTT
TATTCCGGTTCATTTGTCCCAAGAGAGCAATATCTTTCAGGGCATGTCACCATCAGTCAGGCAAAGCATT
ATCTTGACGATAAAAAAAGAATGGTTTTGGCCTATGAATTATTGAATGCCGCAGCTTCAAATATTATCAC
CAATTTAACCTATTATAAAAACAGGGAAAGAGATATCGGTGAAAAAATTTTAAAGATAAAAGAGTTAAAA
AAATCATTAAAAAATGCGGATTCAATTTCATATTTAATGGGCATAGAAGGAAATATCAGGGATTTGTATT
ATTCTGCTTTTAATGAAATTGTTCAAAATAAGGTTGAATTTAAAAAACGTATTAAAAGACCGCCGGATAA
TTTTATAAATTCCCTGATTTCATTTGGTAATTCCCTTGTTTATACAACGGTTCTGTCTGAAATTTATAGA
ACCCAGCTTGATCCTACCGTGAGTTTTCTTCATGAGCCGGGTTACAGGCGGTACTCCCTTGCCCTTGATA
TATCGGAAATTTTTAAGCCCATCCTCCTTGATAGAATGATCTTTACCATGATCAACAAAAAAGAATTAAC
GGAAAAAGATACGGATAAAGACCTTAACTATTGTTATCTGAAAGATCAGGGCCGGATGAAATTTTTAAGA
AAATATGATGAAAGGCTGAAAAAGACTATTAAACATAAGAAACTCAATCGCAATGTTTCTTACAAACGGC
TTATACGGCTGGAGTGTTATAAAATTGTTAAACATATTCTGGGTGAAGAAAAATATACCGGGTTCAAGAT
GTGGTGGTAA

39 986 4

Desulfobacula toluolica Tol2 complete genome GenBank: FO203503.1: 1118078-1118929 CRISPR-associated protein, Cas6 family

ATGATTGCAGGCTTCACTAACCCTGAAGCCTTCCGGAAAGGTATGATTTTGCGTATAAAAATAACAGCAA
TAACGAATCAACCGGCCTTTTTTCCCTATAATTATCAATATGCAGTTCATTCAGCTTTGTACTCTTTAAT
TCAAGAATCATCAGCAAACTATTCAAGTTTTTTACATGATAAAGGGTATGTAAAAGATGGCATCAATAAG
CTTTTTAAACTATTTACCTTTTCAAAGCTCAACTTTAGTCCTAAAAATAATGGGAATGGCGGTTTTTATA
AGGTTTCAAAAATACAATTTGTGTTTTCCACCATCATGGAAAAAAGCATGAGACATTTAATCCTCGGCAT
ATTTTCAAATCAGAAAATGATGTTAAACTTGAGCGGGCAAAGACATTTTTTTGATATTGTAAATGTTGAT
ATTCAGGAAGATTGTCAGTTTACCAATACTGAAAAATTTATTTGTTTGTCGCCCATAACCGTTTCAAGCA
TAATGATCAAAGAAAAAGGTAAAAGGGTGCAACATTTTTTAAACTATATGGTGCCCGCGGAAAGAGATCA
TTTTGTTGAAAATTTAAAGAAAAATCTTATTAACAAATATGAAACCATCAATAATATCCCATATGAAAAT
ATGAAATTTCCTTTTCAGTTTCATTTTGAAAACAACTATATCGTAAAAAGAAATGGCAGAATATCAAAAT
TGATTGAATTTAAAAATAATATTAAAGTCAAAGCCATGGAAGCCCCTTTTTATATAGAGGCAGATCCTGA
GTTAATTAAAATCGGGTATGATTGCGGATGGGGTGAAAAAAACAGCGCCGGTTTTGGTTGTGTTGAAAAA
GCAGCAAAATAA

30 848 3.5

Desulfobcula toluolica Tol2 complete genome GenBank: FO203503.1: 1118938-1119459 CRISPR-associated protein, Cas4 family

TTGAACCGGTTTTTAATGGGAGAAAATATGGATAATATTAATTTTACCGGCACACAGATTAATTATTATT
TTGTCTGTAAGCGAAAACTCTGGTTGTTTACAAGAGCTATTCGGTTTGAAGATGAAAATGAATATGTGCA
GTTGGGAAAACTCATTGATGAAAGCACTTATAAACGCAATAAAAAGCAGATTGAAATCGGCAATATCAAA
ATTGATTTCATTGATAATACGGGGGTTATTCATGAAATCAAGAAATCAAACAAGATTGAAAAAGCCCATA
TTTATCAGCTGAAGTACTATATATATATTTTTTAAATAAAATGGGGGTTAAAAACATAATCGGTGAAATAGA
TTATCCAAAACTGAAGCAAAGACAAAAAGTGATATTGGATGCTGAAGATGAAATGGAATTTAAGACTATT
TTTTCTGATATCCATGAAATCCTTGACAGGAAAAAACCTCCGGCAATAATAAACAAACCGTATTGTAAAA
AATGCGCTTATTATGAATTTTGTTTTGTCTAA

26 518 5

Desulfobacula toluolica Tol2 complete genome GenBank: FO203503.1: 1120549-1120830 CRISPR-associated protein, Cas2 family

ATGTATATTATTGCAATGTATGATATAAA**TACTG**AAACAAAGGCGGGTCGGAA**AAGAT**TAAGACATATGT
TTAAATTATTGAAAAAATACCTTATCCGT**ATTCA**GAATTCTGTCTTTGAGGGGGAGCTTACCAAAGCTAA
ATTTGAGAAA**ATGAA**GT**ATGAA**GTCAACGATATTATTGATAGCTCAA**AAGAT**TCGGTTATTTTT**TTCAA**A
AGCCGTGATATCA**AATGG**ATGGACA**AAGAT**ATCTGCGGGTTTGAAA**AAGATGA**TACGGATAATTTTTATGA

12 278 **4.3**

Methanobacterium congolense isolate Buetzberg genome assembly, chromosome: I GenBank: LT607756.1: 1377274-1377537 CRISPR-associated endoribonuclease Cas2

TCAAAGAATTCCACCGATAGGTGCCTT**TTCAA**CCC**CAAGA**ACCTTCCTTTTAAGTGCTTTCTCGCTCCTC
ATAGTATAAAGTATCACAGAATCCTCATCTTTGTTTATAATACCTTTCAGACCATCCTTTATCTC**TTCAA**
ACTCACTTCTTGTAACCTCTCCCTCAAA**TACTG**AGTTC**TGAAT**CCAGTGC**AGATGA**GTTCTGAGGA**AAGA**
TTTGACCTTGTTAACCCTGTCAACGTTGATGTCATAGACAATTATAACGTACAT

8 260 **3.1**

Methanobacterium congolense isolate Buetzberg genome assembly, chromosome: I GenBank: LT607756.1: c1379814-1378849 CRISPR-associated protein Cas1

ATGAAAAAGAACTACTACCTTATGTCGGATGGATTACTTAAAAGAAAGGAAAACACACTGTATTTTGTCA
ACA**AAGATGGTA**AAAAGCCCATACCCATAAA**CAAGAT**CT**ATTCAA**TATACGCCTACGGATCCCTGACCTT
TTCATCCCAGTCTGTGCACCTCCTGGCAAAGGAAGGCATCCCAATACACTTC**TTCAA**CTACTACGGATTC
TACGACGGCAGTTTCTACCCCCGGGAAACCCTGCTTTCAGGGGATCTTCTGGTGAAACAGGCAGAACACT
ACCTCAACCCTGGAAAAAGGCTCTGGATAGCTAAAAAGTTCATTGAGGGTGCCGCAGGTAACATGGAAAA
GGTTCTGGGCTATTACCACGTTGAA**AATGG**CATTGGAGAAACAGTGAAGGATCTAACCTTTTCATCCAAC
GTGACCGAGGCA**ATGAA**CGTTGAGGCACGTATGCGTTCCGA**GTACT**ACTCAAA**AATGGATGAA**ATATTGC
CTGATGGATTTAA**AATGG**AGGGTAGGAGCAGGAGGCCTCCAGAGAAC**ATGGT**GAACGCCCTCATAAGTTT
TGGAAACTC**AATGGT**CT**ATTCAA**CCGTTATCTCGGAGATATACAACACTCAGCTCAACCCCACAATCTCT
TACCTCCACGAACCCTTTGAGCGGCGCTACTCCCTAGCACTGGATCTGAGCG**AGATGTTCAAG**CCGATAC
TGGTTGACAGGCTCATATTTTAT**ATGGTA**AA**CAAGA**AAATGCTGACCGAGGACGACTTCGAACAGAAGGT
GGAC**TACTG**TATGCTCAACGACAGCGGGCGGAAGAAGTTCA**TCAAG**GAGTAC**GATGA**GAGACTTAAAAAG
ACCATAAAGCACAGGGAACTCAACCGTAAGGTTTCTTACAGGCGTTTGATCCGGCTTGAAGCTTACAAGT
TAA**TCAAG**CA**CATTC**TGGGCACACAGGAGTACAAA**CCATT**TGTTATGTGGTGGTGA

33 962 **3.4**

Methanobacterium congolense isolate Buetzberg genome assembly, chromosome: I GenBank: LT607756.1: c1381325-1380807 CRISPR-associated exonuclease Cas4

ATGCTCCCAAACTCAGAAAAACACCTCCAAGTCAGA**GGTACTG**AAATAAACTACTACTTCATCTGCAGGA
CCAAGCTCTGGCTGTTTTCCCACAACATCC**AGATG**GAGCAGGAATC**TGAAT**CAGT**TTCAATGG**GTAAACA
GCTCCA**CAAGA**AGAGCTATGGCCGTGAGAAGAAGGACTTCA**CCATT**GACAACCTCATAAGCGTGGACTTC
ATAAGGAGGGGTGAAGCCTTGGAGCTCCATGAGGTTAAAAAAAGCAGTAA**AATGG**AGGACGCCCACCGCT
ACCAGCTCCTTTACTACAT**GTACT**ACCTGAAGAAGGAGAAGGGAATCCAGAATATTGTAGG**ATTCA**TAGA
CTATCCAAAGCTGAGGAAAAAAGAGAAGGTTGAGCTCACAGAATCTGGAGAAAAATATTTAGAGGGTATC
ATTGAAGGTATTGGTCAAATA**ATTCA**TGGAA**CCATTC**CAAAACCTGAAAAAAAGAAAATATGCCGTAAAT
GTGCCTACTACGAGTTCTGTTGGGTTTGA

15 515 **2.9**

Methanobacterium congolense isolate Buetzberg genome assembly, chromosome: I GenBank: LT607756.1: c1385116-1384403 CRISPR-associated protein Cas5

ATGGAAAATAAAGCAT**TGGTA**TTTGATATATGGGGGATTATGCACATTTTCGTAAAATTGAAACCACCA
CTTCTCCTCTCACAT**ATTCAA**TTCCAACAGGG**ACTGC**TGTTGCTGGATTAATCGCAGCAATGTTAGGTTG
GCAAAGAGATTCTTATTATGATACTCTTTCTAGGGATAATTT**TGAAT**TTTCTATTAGGATATTAGAACCT
ATAAAAAAAGTTCG**GATGAAT**ATTAATTTGATAAAAAC**AGATGAA**GGATTTTACCTATGGGATATTAAGG
GGACTC**CAAGAT**CCCCTACTCCCTATGAGTTTATAAAAAATCCATCTTATAGAATTTACAT**CCATT**TAAA
AGAAGCGAACACATATAAAACGTTGAAATATTATCTTGAAAATCATAAAACAATCTTTACTCCCTATTTA
GGTATTAGTGAACTCATTGCTAACTTCAGTTTTGTTGG**TGAAT**ATGATATAAATCCTAAAGAGTTAGAAA
GAGCTGAAGTACA**TACTG**TATTAAAAAAG**GATGA**TTACAAAATTTATGT**TGGTA**ATTTAGAAGAAGGAAT

GAGGTGGTCACGAGAGAACATGCCATTATATATGAATTCAGATCGTTGCGTAACTGAATATTCAGAAGTT
CTTCTTGAACAGACTGGAAAACCTCTTATGATTGAAAATGGATTATTCTATGAAATTGGTGAAGATAATG
TTGTTTTCCTGTGA

29 710 **4.1**

Methanobacterium congolense isolate Buetzberg genome assembly, chromosome: I GenBank: LT607756.1: c1386034-1385126 CRISPR-associated protein Cas7

ATGAGTGATGTTGTGAAAAATAGATCAGAAATAATTTTTTTATACGATATTGAGAATGCAAATCCAAATG
GAGATCCAATGGATGAAAACAAGCCTAGGATAGATGAAGAAACTGAAAAGAATATTGTTACTGATGTAAG
GCTCAAAAGAACCATCCGCGATTTTTTATACAAATTTTTAGATGAAGAAATTTTCATAGTAGAAGAAATT
AATAAAGATGGAACCCAAAAAACCAGAGAAAATAGACTTGAAGAACTTAAAATAAGTTCTAAAGAAGATG
GAGTTAAATTACTTGAAAATTATGTCGATTTAAGGTTATTTGGTGCAACAATTGCAGTTAAAAATATGCC
ATTGACATGGATAGGTCCTGTTCAATTTAAATTTGGTAAGTCACTCCATAAAGTAGAACCAAAAACCATT
AAAGGAACTAGTGTAATGCCTTCAAAGGAGAATGTAACTAGAGGAACTTTTATTGAAACTCAAATCCTCC
CATATTCATTAATTTCATTTTATGGAATAATAAATGAAAATGCAGCCAAAAAAACCGGTTTAACAGAACT
GGATGTTGATTTAATGATGAATGGGCTTTGGAATGGAACAAAGAATATCATCACACGATCTAAAATTGGC
CAAATGCCTAGACTGTTATTAAGGGTGGTTTATAAAGAAGATAATTTCCATATTGGAGACTTGGATAAGG
GAATAAAACTCACATTCAATGAGAATATTGAAGAAAAAGCTTTAAGAAATATATCTGAGGTTAAATTAGA
TATCTCGGGTTTAATAGATAAAATTGAGTTAAATAAGGATAAAATTGAAAGGATTGACTTTAAAATCAAT
GATTTAGAAACAACTCCTACATTAAAAGATTCGTTTGAAGGAATCAAACTGAAAGAATTAATGTTTTAA

34 905 **3.8**

Methanobacterium congolense isolate Buetzberg genome assembly, chromosome: I GenBank: LT607756.1: c1388623-1387892 CRISPR-associated endoribonuclease Cas6

ATGCGCTTAAAAATAAGTCTGGCATCCCGGAACAGTGAATACAAAATTCCTTACAATTACAATTATCAAC
TTTCTTCGTTAATTTACAGGAAGATAGCAGACCTTGATCTAGCCAGCGAGCTCCATATTTCCTCTGATTT
TAAATTTTTTACTTTTTCTCAGATTTACGTTCCCAGAAGAAGGATAACCAAGAACGGCATCATCTCAAGG
GATGGTAATCTGCATTTTTACATTTCCTCTCCGAACGACTACTTGATTCAGAGTATGGTTGAAGCTTATC
TCGAGGATCTTGAGGTTAACTTCCGGGGCGATAGGCTGCAGGTTCAGGAGGTTGAGCTTTTAAAGAAACC
TAAATTCAAGAAAAGTGCAGAGTTCAAGACCATATCTCCTGTGATGGCAAGAATAAAAAGGGATGATAAA
ATATGGGATCTGAACCCTGGAGACCTTCAGTTCTATACAGCTCTCCAGAAGAACCTTCTGAACAAGTATA
GGGCATTTCACGGTAGCTACAATGGTGATGAGTATTTGAAGATTGCTCCAGACATGGGATCTGTGAAACG
CAAACGAATAACCATCAAGAAGGACGGCGTGGAAACTTACCACAGGGCTTATCAGATGCGCTTCACGGCT
GAGGGGGATCCGAGACTTTTAGAATTTATGTATGATTGTGGATTTGGGGAGAAAAATAGTATGGGTTTTG
GAATGGTTAATTTAATTAATTATAGTTATTAG

27 728 **3.7**

Metallosphaera hakonensis JCM 8857 = DSM 7519 strain HO1-1 chromosome, complete genome GenBank: CP029287.1: c384573-384313 CRISPR-associated endonuclease Cas2

ATGTATGCCATTTTAGTTTATGACATAGATGAGAGCAGAGTTTCCAAGGTCCTAAAGACCTGCAGAAAGT
ATCTAACGTGGATTCAGAGATCCGTCTTCGAAGGTGAGATTACTGAGGGGAACTTAGAACTACTCTCAGA
AGAATTGAAAAGAATTATCAAGGATCAGGACAACGTCTTGATCTATCTAACCCCAACGAAAAAGAACTTC
AGGGTCGTCTCCATTGGAAAGGAGAGGGATAGAAGCGACAACTTCCTATAA

9 187 **4.8**

Metallosphaera hakonensis JCM 8857 = DSM 7519 strain HO1-1 chromosome, complete genome GenBank: CP029287.1: 392756-393250 CRISPR-associated protein Cas4

ATGTCAATTAAGTTAAGAGAGGACGTAGTGGTAACTGGTACCTTGATCTGGTACCTTAAGATTTGCCCCA
GGGAAGTTTGGTACATGGCGAGGCAGATCACACCCTATGAGGGTCATCCGAAACTGGAGAAGGGACGAGC
GATTCATCAAATATACTCAGATCGAATTCAGATCTCTCTGGAGGGAATGAAGATTGACGGGTTTAGGAGA
GAGGAAAGAACCGTGATTGAGATAAAATCGTCTTCAAGACATCTTGAATCTGCAAGGGCGCAGACAAACT
ACTACTTATATAGATTGAAGGAGGTTGGTTTGAATTGCGAAGGTGAGATTTACGTTCCCAAGAAAGGAGA
GAGGCATAGGGTTACGCTCAACGAAGACCAGGTTAGGAGGGATCTTGAGGAGGTTAAGAGTATCGTAAAT
TCGGAAAATCCACCTCCAAGAGTCAGGATTAAGTATTGCAAAAAGTGCGCATACAAGGACTTCTGTTGGG
GGTAA

20 491 **4.1**

Metallosphaera hakonensis JCM 8857 = DSM 7519 strain HO1-1 chromosome, complete genome GenBank: CP029287.1: 397622-398572 type I-B CRISPR-associated endonuclease Cas1

ATGAAAAGGCTCTTTATACT**TGCCA**AC**GGTAC**GCTGAAGAGAAAGGGGAACACACTCTCTCTGATCACCA
AGGAGGGAACAAAGTACGTACCCATCACCACCATCAG**TTCAA**TTTACGTGTTCGGTGAGGTTAGG**TTCAA**
CAAGAGGCT**CATTCA**GTTCCTGAGCCGTAATCACATACCCGTTCACTTTTTCGGGAAACGTTACCTG**GGT**
ACCTTCTACC**CAAGA**CT**ATTCAA**CGCCTCAGGTTTC**ATGGTACT**TAAACAGGTTCAGACTTACCTGGACC
C**CAAGATGA**GGGCTGGGCTTGCTAGAAAGTTCGTGGAGGGG**GCCATT**AAGAACATGGCCACC**GTACT**CAG
GGAGAACAATTTTCAGGAGGACGCCGTGAAACTTAGGGAGTACC**TCAAG**T**TACTGCC**CGAAGACGT**AGAT**
GGGATCATGGGAGTTGAGGGGAACGCGAGGAGCTTCTACTTCGAGTCCCTGGAT**AAGAT**CCTTGATCAGG
ACTTCAGGC**TGGTA**AGGAGGAGCAGGAGACCTCCCGAGAACATGGGGAACTCGCTCATGAGTTTA**ATGAA**
CACG**GTACT**TTACGCTGCAGTTCTG**GATGA**GGTTTATCAAACCCACCTGGACCCCAGGGTTGGGTTTCTC
CACTCAACCAATTTCCGAAGGTTCTCCCTGAACTTGGACGTGGCCGAAGTCTTCAGGCCGGTTATCGTCG
ATAGACT**GATGA**TTTCCATGATAAATAGGAGGGAGATAACCAAGGACGACTTCGAGG**AGATGA**CGGCTGG
ACTTAAGTTAACCCAAGGGGGGCTAAGGAAGGTGTTAACGGCTTTAGATAAGAGGCTCTCCTCAACGGTA
AAGGTTGGGAACAGGAAAATGAC**GTACT**CCAGGATTATTAGGGCTGAACTGTATAAAATTGAGAGACATT
TGTCCGGGGACGTGGA**GTACT**CCCCGTTCTTAATCAGGTAG

38 947 **4**

Metallosphaera hakonensis JCM 8857 = DSM 7519 strain HO1-1 chromosome, complete genome GenBank: CP029287.1: 401050-401937 type I-B CRISPR-associated protein Cas7/Csh2

ATGACT**AAGAT**AAACAACAACTCCGAGATCCTCTTCTTATATGAGGCAAAGCTCACCAACCCCAACGGGG
ATCCCGACGACGAGAACAGGCCCA**GAATGG**ACCC**CAAGA**C**CAAGA**GGAACCTGGTTAGCGATGTAAGGC**T**
CAAGAGGTATTTCAGGGATTACATCATTTCCAAACTCGGAGAGGACAGCGTATGGGTTACT**AAGAT**TGAG
GGTAAGAACGTCGA**TGCCA**CCGAGAGAC**TCAAGA**GGATAGGTAGTCCCAAAGACGTCTTGGCCAAGTGTA
TAGACGCCAGGCTCTTCGG**TGCCA**CCATACCTGAGAAGGGGGAACAGAAGGGGAAGTCCACGTCCTTCAC
GGGACCGGTCCAGTTCACCTGGGGATATTCCCTCCACAAGGTTGAC**ATGGT**CGACTCCAGGT**CCATT**ACT
TCCCTATTCTCAGGAAGGGACACCG**GGTAC**GGTAACATAGGCAAGGACTACAGGGTATACTACTCCCTGA
TAGCGTTCCATGGGGCCGTGAGCGC**CAAGA**GGGCCGAAATCACGGG**TGCCA**GCGAGGAGGACCTAAAGAC
GCTGGATAACTCCCTATGGGACTCCCTCGT**TACTG**AGACGGTAACCAGGAGCAAGCTGGGACAGAGGCCA
CTCCTCTACCTTAGAGTTGA**GTACT**CCTCCCCAGAG**AAGATGAA**GGGGGACCTGAGGAGGTTCGTGAAGG
TTAAGGAGAAAAGTGAGAGCGTGAGGGACTTGACAGACTTGGAAGTGAACCTCGACGCTTTGG**TGAAT**CT
CGTGAGCTCCAACGACGTGA**TCAAG**GTTGTAGCGAGGTGTTCCGACGAC**TTCAAG**GGATTCTGCGAACAA
GTTAAGAGTGCCTGC**GATGA**GGGGAAGAAGA**ACTGC**ATCGTAGGGTAG

27 884 **3.1**

Methanobacterium sp. BRmetb2 chromosome, complete genome GenBank: CP022705.1: 468549-469262 CRISPR-associated endoribonuclease Cas6

ATGAGATTGAAAATCAATCTAACATCACAAATGAGTAATTATTTAATTCCTTATAATTACAACCACATCC
TTTCTGCTATTATTTATCGT**AAGAT**ATCTGACCTTGATTTAGCAGCAAAACTTCATTT**TTCAA**AGGATTA
TAAATTTTTTACTTTTTCACAGATTTACGTTCCACAATTTAAACGTACAAAACAGGGCATTATTTCCAGA
AATGGAAAATTAGAATTCTATGTTTCTTCTCCTAAT**GATGA**CTTAATAAAAAGCCTCGTAGAAGGGCACC
TTGAGAACACCGAAGTAAAT**TTCAA**AGGTGATAAAC**TACTG**GTGGAGCAGATTGAACTTCTAAAAAAACC
TATTTTTAAAGAAAAT**ATGAA**A**ATGAA**AACCATGTCACCAATTATGGCCAGAATAAAGCGAGAAGTTGGG
GGAAAACTT**AAGAT**ATGGGATCTGGATCCTGG**AGATGA**GCGGTTCT**ATGAA**AGTGTTCAGAAAAATTTGA
TTAATAAATATGTTAAATTTTACGGGGATTTTG**ATGGT**GAAAGGTGGGTTAGGATAAAGCCTGAT**ATGAA**
ATCTGCTAAAAGGCGCAGGATTGATATTAAAGGAACTTATCAC**AGATG**TTTCAT**GATGAAT**CTTGAGATT
GAAGCTGATTTGAGGCTTTTGGAATTTGTTTATGATTGTGGGCTTGGTGAGAAGAATAGTATGGGATTTG
GG**ATGGTTGAAT**AA

20 710 **2.8**

Methanobacterium sp. BRmetb2 chromosome, complete genome GenBank: CP022705.1: 471663-472280 CRISPR-associated endoribonuclease Cas6

ATGAAAACGCTAAGAATATTGATTGAAGGTTGGGTAACTTCTTTCAGATACCCTGCTTTTATCAGCGGAT
TTCAGCCAACTTTACCAGTACCT**CCATT**AAGTACAGTTTATGGCCTTATATCTGCAGCTAAAGGAGAATT
AGTTACTCCAGAAGACTTATCTGTTGGTTATATA**TTCAA**TTACCAGGGAAAAGCAGTAGATTTAGAAACA
ATAT**ATGAAT**TATCTGGTTTAAAAGGTAAATCAAACGTTATTAAAAGAGAATTTCTCGTAAATCCTGAAA
TTTATTTATATATG**GATGA**TTTGGATTATCGAGATTACTTTAAAAAACCGCACTATCCTCTCCTTTTAGG

GCGTTCAACAGATCTAGTAACTATCAGTGAAATTAAAGAAGTAGAGCTTGAAAAAAAATCAAACATAAAA
CTTGGTAGAACCATACTCCCATTAGGCACAAACGGTGCTTTTGGTACTATTCAATCATTACCAACACATT
TTACTAACACAATTCCACGTAAAGCAGTAGGCACTAAACCATTTATATTAATGAACCAGTTCTTTGATTA
TCAAGATGAATGTTATTTTGATAAAGAAAAGGAGTGGGGAGTTTGGTTCCACAAATGA

24 614 3.9

Methanobacterium sp. BRmetb2 chromosome, complete genome GenBank: CP022705.1: 474545-475060 CRISPR-associated protein Cas4

ATGCTTTCAAACTCTGAAAAACAACTCCGGATAATAGGAACCCAAATTAATTACTATTTCATCTGCAAGA
CTAAACTCTGGCTTTTTTCTCATCACATCCAGATGGAACAGGAATCTGATCTAGTTTCTCTTGGAAAATT
ACTGCATTCACAAAGTTACAAAGATGAAAAAGAGTTTACCATTGACAATCTAATCAGTATTGATTTTATT
AAAAAGCAAGATTATTTAGAGCTTCATGAGGTCAAAAAGAGTAATAAGATGAAAGAATCCCATGAATACC
AGCTTCTTTACTACATGTATTATTTAAAAAATGAGAAAGGTATTGAAAATATCAAAGGCGTAATTAATTA
TCCTACAATAAGAAAAAAAGCTACGATTATTTTAGATGAGTCTAAAGAGAAGGAATTAATGAGTATAATT
GAAGATATTGGTAGAATTATTAATAATGACACTCCAAAACCTGAAAAAATGAAAATTTGCAGGAAATGTG
CATATTTTGAGTTTTGTTGGGTTTAA

22 512 4.3

Nitrospira moscoviensis strain NSP M-1, complete genome GenBank: CP011801.1: 1417060-1417353 CRISPR-associated endonuclease Cas2

ATGGCAGAAGATCGGGCCTGGTACTTGATCTGCTATGACATCCGCGACCCTAAGCGGTGGCGGCGCGTGT
TTAAGCTGCTGAAAGGCTATGGGCATAGCCTTCAGTACTCCATCTTCCGCTGTCGGTTGACTAAGCGCCA
GATCGAACAGTTGCGTTGGGAATTGGCGACTCTGCTCGATCCCGTCGATAGCCTGATGGTTGCAAGTCTG
TGTCCAGGATGTGCTTCTCGGATGGAGGTTCAAGGAGGGAAGGGTGACTGGGATTCCGAGCATGAGAAGT
TCAAGGTTCTTTGA

9 290 3.1

Aeropyrum pernix K1 DNA, complete genome GenBank: BA000002.3: c784515-784237 CRISPR-associated protein Cas2

TTGGTGTACGTTTTAATAGCCTACGATATAAGCAATGATTCTAAACGCCTCAAGGCCGCACAGAAACTTC
TGCAGATGGGATTCGCTAGGGTACAGAAGAGCGTGTACATCGCGAAGGGAGGACGGAGTCTTGCCAAGGA
GGCGTACCGAGCTCTCCAGAGGCTAGCGGACAGTGGGAAAGACAAGATCATGGTGATGGTTATTCCGGGC
GACAGTGTTAGGGATGCCTACGGTCTTGGGGGTAGTCTTGAGGATGGGAAGAGAGTTGTGGTGGTGTAG

8 275 2.9

Thermoanaerobacter kivui strain DSM 2030, complete genome GenBank: CP009170.1: c1756606-1756046 CRISPR-associated protein Cas4

ATGATTCTTAATGTAATTTTTTTATTTTTTGTGGTGTATTTACTTATTCAAGCTATTATTGAAAAAAGAC
CACCTTATAAGAAGATGAGGAGAAGTATTGGGTTTCGCGGTGGCAAAATAATATACATAGACAAGCAACA
AGAAGTTAAAGAAAAAGGCGTTACATATGGGAAACTATTAAAATCGGATAAATATGGTTTATCTGGAAAA
CCTGATTATATTTATCAGTTGGGAGAAGAACTTGTTCCAATAGAGTTAAAAAGCAGTGAAGCTGATGATT
CTCCTTATTATAAAGATGTTATGCAGCTGGTGGCGTATTTCTTGATAATAGAAGATGTGTATAACAAGAA
AGTAAGAAGAGGGAGAATAGTTTATAGAAACACTATGTTTGAGGTATACAATAAAAAATATTTGAAAAAA
GAACTTTTAAATCTTGTAGATAAAATGAAAAAGATGAAACAAGGAAATTATTTTCCAGAGATAAAACCTT
CATTTGCCTTATGCAGATTTTGCCCCTGCAGAGGTACTGTCTGTGAAATATACAATGACGCTTCCAAATAA

22 557 3.9

Thermoanaerobacter kivui strain DSM 2030, complete genome GenBank: CP009170.1: c1666773-1666372 CRISPR-associated endoribonuclease Cas2

ATGCCAAATGTTAATATCAGAGCTCTAAAAAAATGAGATATTAAAAGAAGTTAAGACTGCCATCAATCCTG
ATAAGGACAAAGATGGCAAGGTATTTTCTGAGGTTGCAAAGAAAACAGGGCATCTTTTAAAAGAATCCAA
TGTAACGGTTACACAGCTGCGCAAGGTTTTCACAGAGGTCAAAAGACTTTCCCCAGAGGATGAGAACTAC
AAATACAAACTAAAGCTTTTAAAAGCAAAGATGGCATATACATCAGGAAGATTTCCAAAGCTAAAAGATT
TTCAAGATATAGTCGATGAGGCATTGCCAATTGCAGAACAAAATGAGAAAACATTGGAGAGATTTAAAGA
CTTTTTTGAGGCAGTTGTTGCATACCACAAATTCTTTGGCGGAAGAGAATAA

19 398 4.8

Observed mean proximity: 3.6 Expected mean proximity: 2.15 ± 0.9* (* 90%-confidence interval calculated from the smallest sample size equal to 187). Data from https://www.ncbi.nlm.nih.gov/nuccore/CP021507.1?report=genbank

AL-pentamer proximity of the capsid proteins of 15 virophages

Zamilon virophage complete genome NCBI Reference Sequence: NC_022990.1: 3374-5203 capside protein

ATGTCAAATAGTGCTGTACCTTTAAATGTTGTAGCA**ATTCAAGA**ACCCCGTCTTGAACTTAATA**ATGAA**A
GAACTTGGGTAGTGGTTAAAGGTGGTCAACAAGTCACCTATTATCCTTTCCC**TTCAA**CTTCTTTCAGCTC
TAATCAATTTAATTTCATTTGTAATCCTCCTTCAGCACAAACTGTATTGGATCGATTAG**CATTCATTCAA**
GTTCCTTATGATATCACT**TTCAA**TGTTAATCCTGCTCATGCTGGTGTAACTGACAATCTTTTACAACCTG
GTCGTGATGCTTTCCGTGCTTTCCCTATTAGTTCTATTACTAATACTTTA**ACTGC**AA**CCATTAATGGT**TT
CCCTG**TGAAT**AT**TGAAT**TAGGACAAATT**ATTCA**CGCTCTTAGCAGATATCATACTCCTCTTAAACTTAAA
AATGGATGGATGTCTATGCAACCTTCTTTTG**AAGAT**AATTATCAATCATATCGTGATGCTGA**TGCCA**CTA
ATAACAATCCTTTAGGTGATTTTACAAGCGCATCTGGTCTTTCTGAGCTTC**CAAGA**GGATCATATTCC**AT**
GAATGTTGTGTCAAATACTCCTAC**TACTGC**TAGAAT**TACTG**GT**GTACT**TT**ATGAA**CAAGTATTCCTTCCT
CCATTTATTTGG**GATGAA**CA**TCAAG**CTGGTGGTCTTGCAAATCTAACCAGTTTAACC**TTCAA**TTG**GGTAC**
TAAATAATAATCTTGCTAGAAT**ATGGT**CT**CATTCA**GATATTACTAATGATGTTTCTGGAAACA**GTACT**AT
TGG**TTCAATGAATG**TCAGCTTCCAACAACCCTCAATGTATCTTGGATTTGTTACTCCTCGATTAAATATT
CCTATTCCTCCCAGAATAACTTATCCTTAT**TTCAA**ACT**TTCAAGAT**ACACTACACAATTCCAAAATACTC
TTGCTCCCAATGCAACCA**GTACT**TACAAATCTAATATTG**TTCAA**CTTGATTCTATTCCAAGGAAACTTTA
TGT**ATTCATGAA**ACAATCTG**ATTCA**GTGATTTATCAAAATCTCAATAATCAAATTACCACTCCTGATGTA
TTCCTACAAATCAATTCTCTCAATTTAACTTGGAACAATCAACAGGGTGTTCTTTCTGGAGCATCTGCAC
AAAATTTATATGACTTCAGTG**TTCAAAATGGT**TACAA**CAAGA**CTTGGAC**TGAAT**TT**AATGGT**CTTACACA
ACAATTATCAGGAGTCTCTGGATCACCTACCAAAGTAATTGGTCTTGAAGGAGGAATAGTTTGTCTCGAA
TTAGGTA**AAGATG**TAGGATTACGTGAC**GATGAA**GCTGAAGGTGTGCT**TGGTA**AT**TTCAA**TTTACAAGTAC
AAATGACTTGCACTAATACTAATCAATATCTCACAATCGTACCTGATATGTATATTATTGCAGTATATG**A**
TGGTACTCTTGTTATCTCTAATACTAGTGCTATGGCATCAATTGGTGTTGCCTCTAAAGAAGAAGTATTA
AATGCTC**CCATT**AATCATAGCATGTCATATCACGAATTACAAACTGTATATGGAGGAGATTTCTTTAGCA
CATTTAAGAATTTCCTTGGAAAAGCCGCTAATGTAGCCGGAAAGGTAAATAATTTTCTCA**AAGATTCAA**A
AGTAGCTAGTTCTGTGCTAGGAGCTATTCCTCATCCTTATGC**TCAAG**TTCCTGGACAAATTTTAAGGAAT
ATTGGTT**ATGGT**GAAGGTGGAGTATCTGCTGGTGGTGTTGTTGCTGGATCTGGAAGAAGAAAGAAAGGTG
GAGTATTAGTAGGAGGCAG**TGAATG**TGAAGGTGG**AAGAT**ATATGTCCAAAGC**TGAAT**TAAAGAAAGCTTT
AC**GAATG**TAA

83 1826 **4.6**

Sputnik virophage isolate Rio Negro virus capsid protein gene, complete cds GenBank: KJ183141.1

ATGTCTA**ATTCA**GCTATTCCTCTTAATGTTGTAGCAG**TTCAAGA**ACCTAGACTTGAACTTAATA**ATGAA**A
GAACTTGGGTTGTTGTTAAAGGAGGTCAACAAGTCACCTATTATCCTTTTCC**TTCAA**CTT**CATTCA**GTTC
TAATCA**ATTCAA**TTTTATTTGTAATCCTCCTTCTGCACAAACA**GTACT**TGATAGACTTGTTTTT**ATTCAA**
GTTCCCTATGATATCACTTT**TACTGCCA**ATCCATCTCATGCAGGTATAACTGAAAATTTAC**TTCAA**CCTG
GTCGTGATG**CATTC**CGTG**CATTC**CCTATTAGTTCTATTACTAATACTCTCAA**TGCCA**CTATA**AATGGT**TT
CCCTG**TGAAT**ATTGAACTTGCACAAATT**ATTCA**TGCACTTAGTCGATATCATACTCCTCTCAAAGTTAAA
AATGGATGGATGTCTATGCAACCTTCATTTGAAGACAATTACCAATCTTATAG**AGATG**CTGATGGAGCCA
ATAACAATCCTCTTGGAGTATTTACTTCAGCAGCTGGTCTTTC**TGAAT**TACCTAGAGGATCATACACA**AT**
GAATGTTGTAACTAACACTACTAC**TACTGC**TAGAAT**TACTG**GTGTTCTTT**ATGAA**CAAGTTTTCCTTCCT
CCTTTCTTGTGGGATGGAGAACAAGCTGGTGGTTTAGCTAATCTTACTAGTTTAA**CATTCAA**TTGGGTAT
TGAATAATAATTTAGCTAGAATCTGGTCA**CATTCA**GATATTACTAATGATGTTTCTGGAAATA**GTACT**AT
TGGATCA**ATGAAT**ATTAGTTTCCAACAACCAAGTATGTATCTTGGATTTGTTACTCCTAGACTTAATATT
CCTATTCCTCCTAGAATTACTTATCCTTAT**TTCAA**ACTTTCCAGATACACTACTCAATTCCAAAATACTC
TTGCTCCTAATGCTTCTTCTA**CATTCAA**ATCAAATGTTGTACAACTTG**ATTCAA**TTCCTAGAAAACTCTA
TTTGTTTGTGAAACAATCTGATAATGTAATTTATCAAAATCTCAATAATCAGATTACTACTCCTGACGTA
TTCC**TTCAA**ATTAATAACT**TGAAT**TTAACATGGAACAATCAACAGGGTATTCTTTCTGGAGCATCTTCAC
AAAATCTTTATGATTTCAGTG**TTCAAAATGGT**TACAATAAGACTTGGTC**TGAAT**TT**AATGGT**GTAACCCA
ACA**ATTCAATGGT**GTTTCAGGACAACCTACTAAAGTCATTGGACTAGAAGGTGGAATTGTTTGTTTAGAA
TTAGGTA**AAGATG**TTGGTCTTAG**AGATGATGAA**GCTGAAGGTGTTATTGGAAAC**TTCAA**TTTACAAG**TTC**
AAATGACTGTAACTAATACTAATCAATATGTAACTGTTACTCCTGATATGTATATTGTAGCAGTTTATG**A**
TGGTACTCTTGTTATCTCTAATACTAGTGCTATGGCTTCTATTGGAGTAGCTTCTAAAGAAGAAGTAT**TG**
AATGCTCGTATTACTCATGGAGTTTCATACA**ATGAAT**TACAAAGAATTTATGGAGGTGATTTCTTTAGTT
CATTCAAGAATTTCTTAGGAAAAGTAGGTAATGTTGCTGGAAAAGTCAATAATTTCCTCA**AAGAT**TCTAA
AATAGCAAGTTCAGTTCTTGGTGCAATTCCTCATCCTTATGC**TCAAG**TTCCTGGTCAAATCCTTAAAAAT
GTAGGT**TATGGT**GAGAGTCATGTTGGTGGAGGTAAGAAAAAAGGAGGAGTTCTTATTGGCGGAAGACAAC
TTACAAAAGC**TGAAT**TGAGAAAAGAACTTAAAATGTGA

89 1784 **5**

Megavirus vitis isolate vigne Megavirus vitis transpoviron mobile element, complete sequence NCBI Reference Sequence: NC_040848.1

ATGGTTAGAAGAAAAAAATGTATAAAATTTAGAGGTAAAAATGTATATTATGCAAATATACAAGATTTGT
CTAAAAAATTAAATATTCCTATTACGCAAGCTAGGAAAATAATAAATGATAATGGTAGAAGAATTGTTAT
TGATTCAAATAATAACACATTATTAATTAATATCAAGAAAGAAAATTTTACTGGACTTTTGAAAACAAAT
TTTGGTATCAGTAGAATAGATAATAAAAAAATTATTAATGATACTCACATTCTTAATAAGGGTATTGATA
TTGTTAAGGAATTAGGACCAAATACAGAAGGAAAATATTTAATAAAAATTATTCTTTACATAACATTTGT
GAGTCCTGATCCAGTTAATGAAGATGAAATATTAACTAATGATAAAGTGGATTATGTTAAAATACAAAAT
TTAATTGATGAACATCGTCTCGAGGAACGAGAAATTACTACACCATATAATGGTTACAATGACGAAATTC
CTAATTTTATAAGAAGGAAAATTAAGGAATATATGAAAAACATTAAAAAATCTGGTGGTCAATTAATACA
TTATGAATATCGTATTGGTTCATATTATAAAAATAGAAGATTAAAATTTAAAAAGGGATATGTTAGGGAA
TTAAATAATGTTTATGAATTAACAGAATGGGTAAACATTCAATACGAAAATAGTGATAATACAAATGATA
CATGCGTGGTTAGAACAATTTCCAAAAGATATCCTAAATTGTACTGGAAAATAAAAAAACTTGAAACTAT
CCACGGCGTTAAAGTTCAAGATTTTTTAGGATTTTGTCAAAAATATGATATCGGTTACAATATTTTTAAT
GAACTTGGTAAAAAATTATATAATTATCAGGGTTCGGACGGATTACTTAACTGTATTATTTACAATAATC
ATATTTATCCAATAAATGGCGGTAAACCAAAAAAATATTCTACAAAAGAATACAAGATAAATTTTGTAGA
TGATTCGTTAAAAAAACTTAAAAATATATTAAACACTGAAAAAAAAATTACCTTCTAATATTAAAATTGAT
TCAGTTAGAAGAAAAAATAAGATAGATAATATTAAAGATGTTAATATTACATCATTTACAGTGAAGGATA
AAAAATTCATTTGTAATGATGAATATCAAGAATGTTTAAAAATATTAACTAAAATGGGATATAGTGAATA
TATTTTTGATAATATACGAATAACAAATATTCCTAATTTATTAGAAAAAATATTAAAAGTAGACGATGTA
TCATCATTTATTCCTGAAAAAGAATTATTTAAAACATCACCGCTATTATGGAAATGTAATAATGTAATAA
AGCCGAATCGTGTTATAACAAAAAATAATAATGATGTAATAGAATGGTTAACAACCATCGACAAAAATAA
ATGTTATTCATATTGTCTTTATTCTTTACCTTATCTTATAAAATTCGATTATAGAAAACATAAACTTAAT
GATAAACCAACAAAAATTATTGATAATAATTTATATTTGGCAAAACCAAAATTTTGGACTATTTTAATGC
CTAAAACTAAGATATATCCTGGATATTTTTTATCAGAATGTAGAAAATATTGGTATTGAATTTGATTTATT
AGAAGAATTAGAAACAGAAATAGTTCCTAATTATTTTAGAAAAAATAATTAAATTAATGTACGATAATATG
TCAGCTGAATCATTTAAAAGTGCATTAAATATATTTATAGGTACATTTGAAAGAAATGATATGCAAACAT
ATAATTACGAATATAGCGGTATATTTACAAAAGAAGCAGTAAAGGCACAAGAAGGTTTTTATAAAAAAAT
GGGCGATCATTATTTATTATTCAAAGAAACCGAACAATATTTTCATGTTCGTGATAGATTACCAATTGCT
TCACAAATAAAGGATATGTCTAGAATGATTTTGTATAAAAAAATAAAAGAATTAGGATTAAAAGATGATC
AGATTGTACAAATAAATACTGATTCAATATCATATTATGGTGAACTGCCAAAAGAATTAGATCCAAGTAT
TTTATCTGGTTGGAAACGATCGGATTTTAAAGAATTAGGTGATGTTAACAATTTTATCGATGAAGAAGTC
AGTGTTAAAAATATTACTAACAGTAATAAAAAAACAAGAATTATTCATCAACAATATGCAGGATCGGGTA
AAACGACATATATTATTGATAATCTTGTACCCAGATTATTAAAAAAGGGTATATCATTTATTGTATTAAC
GCCTACACATAAGACATTAGATGAATATAGAAAAAAATAATATAAACTGTGAGATCATGCAGAAATATGTT
TTCACAAATACCATTCCAGAAGAAGACTATATTATTGTAGATGAAATTGGTTTTGTTGATCGCGGATGTC
ATGATTTACTTTATAAAATAAATAAAGCCAATAAAAAATTTTGAATGTTTTGGGGATTTTAATCAACTACA
ACCAGTGGGAGAAAATCAACCACTAAATCAGCCACATTATTTGAAATATATGTTCAAATATATATATACACT
GATTTTATTAATTATCGCAATAACTTCACGAAAGAATACTATGATGATTTAATAAACGAAAATATAGATG
TGATATGTGAAGTAAATAAATGGTCGCATAAAAAATATGATGAAAGCGGAATATATTCTATGTTTTCGACA
TAAAACAAAAAATAAATATAATAACATCATGATAAAAAAATTAGGTTTTAATTCATGGCGTGATATTGGT
GTGAAAATTGTTTGTGTAACAAATAAATTAATTGAACACCATATTTATAATAATAAACAATTCACCATAT
CAGATATTAATAAAAATGATAAAAATTATAAATTAAAAGATGATAATGGTGAAGAATTTGTTGTGACCGA
AAGAAGATTATTATCTAATTTCGAACCTGCTTATGCCATTAATATACATCAAGCACAAGGTATGACACTA
AATTCATATTTTTGGGCTTCTGATGATGATAATTTTATTAATGGGAATGTTGCTTATACAATTATCAGCA
GGTTAAAACAAAAACTTAATTTTTCATTGGAAAGAGAATTTATGAAAATATTATTGACTGAAAATAATTA
TTTATAA

123 3083 4

Maverick-related virus strain Spezl (virophage at the origin of large DNA transposons), complete genome NCBI Reference Sequence: NC_015230.1: 14876-16696 putative major capsid protein

ATGAATACACCCCCAGAACTCGATACTGTTTTACAAGCTCCATATGCTTATAATTGGCCCACTTCTAAAA
ATGTTAAAATTGCTTCTAGAATAGGCATTCCATACTCAACATTTCAAACAATTCAACCCGTATCAGATGC
ACCTAATAATGGTATAGGTCAAATAACTTTTAATCAACCTCTTGGAAATTTAACAGGAGGTGCTCCACGT
TTAAGAGTAAGTTTTACTGCTGAAATTAAAAATATTTTAGCTGATTCATCTTTAAAAGATCAAATAGGCC
TGAAATCTTTTCCAGTTAATAGAAGTATACCTGTTGCAGTAATTAATATGAATGGTAAAACTTTTACCAG
TTACCCCGCACAATTAATTAAACTTCATCAATATAATGCGGATCCTCTAGAACTTGCTTTGCTTTCTCCC
TGCTCTGATGTAGATGAATATAATAAAATAAAAGCCGTTTCTATGAATAACCCTTATAGACAAGGTACAG
AATCTACCGATAGCCGAATGTCTCGCGGTTTAGGTTGTAATTATGCCTATTATATCCACCCACGGGCAGC
AGGTAGCACATCCGTAAAAATTGATTTTGTAGTAGATGAAGCACTTGTAGCCAATCCCACACAATATAAA

AATATTAAAGACCCAGTACCTTTTAGAAACCTGAACACATTTAAAGTCATATTAGATGGACAATTTAAAC
CTGAAAATATGATTGGTATTGCGGATGATGTTAAACTTGTGGCGGGTAAAGCAGATTTTGAAGTGGATAT
AACCGGATTTAAAATTAATATGCTTGTTCAAAACTGGGTCGCCCCTCTTGAAATTGGTGACATTCCTAAA
ACTATAATATATAACACCCCGTTAATTTCATTAGAAGGTAATATAAGTTCGATGTGCCTCAATACAAAGG
ATCCTTACGGTATCCCTGGTGAACGAAATAAACACATATTAACAACTCATAGTATGGCAATGAATAATGT
CCCTTCAATGTTTGCTGTTATGGTTTCACAAGAAACACCAACAAAAAAGTTTGCCCCTGATCAACTTGCT
GGAATTATTGGATTAGAAATTAAGGTGGATTCTGATGTTGGTATTTTTAGAGAACTTGAACAACAACAAC
TGTACGAATTATCTTCATCTAATGGATATAATAAAAGATTTTCATGCTTTAGTGGTGCTCTGGCTAATGG
CTTGACTGTGGCAGATCCCGCTGTGGCTGCTGGTAACAAATTTAAAGAAGCTATATTTGGTGCTGGTTCT
GTAATTTTCTTCCGTCCTTCCGATCTGGGATTAAAAGATTATAATGTTATGGCTAATGCTAATAAGTCCA
TTAATATGCAGGTACAAGCAACATTTGTCACCCCTGAAGCTGCGGGTACTGGTGCTCATTATAAATTAGA
AGTGTTTTCAATTAGAGATAATCTCACCTACAGCTTTGAAGATGGTACCTTTATGGATGATTTAACCCTT
TACACTCCCGACCAACTATTACGAAGCCCCCTAAAACTTACTGATGACAATAACAAATTAATGCGGGTTA
TGGGCGGGTCCTTTATGGGTGATGTTATGACTAATTTTAACCATATGGCAGCTCATCCTGTGACTAAAAC
GGTGACTAAACTTTTAAGAAATGCGGGACCATTAAAAGATTATGCGGGAGATGGTACAATGATGGGAAAC
ATTGCCTCCGTTTATGGTTATGGTAAAAAAAAAACAACCACTAGAAAAAAGAAAGGTGGAGAAATTGTTT
TACTAGGATCAGGAAAAAAAGGCGGTAAAAAATTATCAGATAAACAACTTCACGACTTAAGAAACCTCTAA

71 1817 **3.9**

Moumouvirus australiensis isolate 10A, complete genome GenBank: MG807320.1: 471905-475239 capsid protein 1

ATGGCAGGAGGATTATTACAACTCGTAGCTTATGGTGCTCAAGATGTTTACCTAAACTAGTTGGGTAGAA
AAGTAGGACGCCGTGATGAATATGGATAAGTCACGGATAAACCGGTTTGTGGTCCATTAAATCGTAATAA
ACTTACGAAACCACGCCTGCTAGTGGGAATAATTTTATTCCTGCGACACCTTCAAATTCAGATGAACCCC
TAAAGCCGTATAAAGTAGATAAAAAAATTGAATTCTCAAAAATTGTTTTATTACAAATTCATGTTATTAT
TAATATGGAAACTAAAATATGCACACAATGTAAAGATGAAAAATCTTTAACAGAATTTGGTGTAAGAAAT
AAAAAGAAAAATATTTTACATCAATGGTGTAGAGTATGTCTTAACGAATATGCCAAAAAATACCGCGAAG
AGAATAAAAATTTGCTTAAAAAAAGACAAAAAAAGTGGTATAAAACTAAAGGTAGAAAATGGAAAAAAAT
ATATGATAAAAATAATCTCGAAAGAACAAGACTAAGAGATAATGAAAGATATCATTCTGATCCAAGTTTT
AGAACCAAAAAAATACTAAGATCAAGATTAGCAAAAGTACTTAAAGGTGAGAAAAAAATCTAAAAAAACTT
TAGAATATGTAGGATCTGATATATTATTTATCAGACAATGGTTTGAATTTCAGTTCGATGAAAATATGAA
TTGGGAAAATCAAGGTACTTATTGGGACGTAGAACATGTTATACCTTGTTCTGCTTTTGATCTTTCTCTT
GAGGAAAATATTTATAAGTGTTTTAATTGGAGAAATTTAAGACCTTATGAAAAGAAAGCAAACGGTAAAA
AAGGCAATAAAATAATTATTGAAGACATATTTAATCACAAGAAAAAATACAGAGAATTTGAATTATTTTA
TCTATTCGGTACCAAGTAACATGGGAAACTATGTTATGGCCTGGAGAAGTAGCCAGGGTATGGTCACAAG
CCGACGGATGATGAATTTGGTTAAAAAATTTAATCAAATTTTGAAATGGGCAAATGCTGAGCCAAGTCCT
AAACCTCGTTATGCAAGAGGCAAGGATGCAGTCCAGAGACTAGAAGGAGGTGCGCGTGAGAGAATTAGCA
GTTCTCGATGATCGTGTGAGGTATAGTCCGTCCCCATCTCGAAAGAATGGGATCCGACAGACAGGGAATC
CGCAAATAACTTTTTTTAAGGTTTGAATACAACATTTCTGGCCTTAGTAGTTATTAAAGGTAACTGCTAG
TCATTTTATCAATATTGGTGTAGTAAGCAACCTGCCGCTGAAAAGATCCGATATTGAAAAATGGCGACAC
TGTCAAATTGCGGGAACGTCCTAAAGACGTCGTGTACCAAGTCAATGGAGAAATCTATTGATGGCCGAGA
TTGGAACTCGGGTATGGTAAAAATCACGCGTATGATTAAATAGAGTCATTCTATTTATGAAATGGATAAT
CCGCAGCCAAGTCCTAAATACCGTAAAAATGATGAGTATTTCAAAAAGTAAAATTTTTGGAATCACGGTA
CATGGATGCAGTTCAGAGACTAAATGTCAGTGGCGATGAAAGAAACTAATCATTTCTTTATGATTCGTTA
AGTTATAGTCCGGCCACATGGGAAACTATGTGGACTAACCGCGTATATCGTCGTCATACTAACTTTGCGA
CTGAATCAATTGAGCAATATTTTAATGGTAATACTAATTTCGGAAGAAAATCCACCGCAGAAATATCTCG
TAATGGAGATCTTATTACCCAAGCTTTCCTTAAAGTAGTATTACCCGAAGTAAGATTTACTGGTCCTTTT
GATAAATTCGGACATGTTAAATTCGCTTGGGTCAAACATATTGGTCATGCTATTGTTGATGAAACTGAAC
TCGAAATCGGTGGTTCTACTATTGATAAACAATATGGTGATTGGCTTCACCTCTGGCAAGAAGTTTCTCA
AAGTAAAGATCATGAAGCCGGTGCCAATAAGATGCTTGGTAATGTTCCTGAATTAACATCTATCAGTACT
CTTGATTGGGACAGTTATGATAACAATCTTCTTAAAAATTCATACACTCTTTACGTTCCTCTTCAATTCT
ATTTTTGTCGCAATAATGGTCTCGCTCTCCCTCTTATTGCTCTCCAATATCACGTAGTCAGAATCTACGT
TAAATTCAGACCCGCTGAACAATGTTACATTGCCAGTGATGCTTTCAAGAGTGGTGCTGATAACTTTGAA
CTTGACGATGTTTCAATTTATGTCAACTATGTTTATCTTGACACTGAAGAAAGACGAAGATTCGCTCAAG
TTTCTCATGAATATCTTATTGAACAACTTCAATTCACTGGTGAAGAATCAATTGGTACTAGCAACTCTGC
TAAATATAAACTCAATTACAATCACCCCGTCAAAGCTCTTTATTGGATCACCAAATTAGGTAATTATCAA
GGAGGTAAATTCATGGTTTATGATCACGAAGATTGGGAACGTGCTCGTGAAAATGCCGCTAAACTTCTTA
TCCTTGCTCAATATGATCTTGACGAATTTGGTTATTTCAATGATGTTGCTCCTGAACAAAACGATAACAG
TTATCATGGTGATTGTGGTCTTCAATATGTTGGTGTAGATCCTTCAAGTACTTCTGAAGAACCCCATTAT
ACCTTCAATGATTCACACACCGCTGATGCCTTTGATGGTACTGTTCTTATTGGTAAATTAGCTCCTTGTG
TTCCTCTCCTTAAGAGAAACAAAGATGTTGATCTCCGTGATAAAGTTGATGGTGTTATCCGCATTGTTAC
TGATTTCGAAAATGATAACTTAAGATATCCTGAAGTTGAAAAAGTTACCAGAAACGATCTTTCTATTTCT

GATCT**TTCAA**TTCCTGTTGATAAATTC**GATGAA**GACAACCGTGTTGATTACATTAAGAAACACGATATTA
CCGTATGGCAACACGACAACTACGGTCTTCTCATTGACG**GTACT**AAAAATCCTGTAAGTGATGCTGAACT
CCAACTC**AATGGT**CAATCACGTCAAAG**CAAGA**GATCTGGCTTCTGGCATGA**TACTG**TTGAACCCTACATG
CATCACACTAAAACACCCA**AAGATGGT**CTCAATGTCTTCTCATTCGCTCTTAACCCTGAAGAACATCAAC
CATCAT**GTACT**TGCAATTTC**TCAAGA**ATTGA**TACTGC**TCAACTCAATCTCTGG**TTCAA**TCACTTTGCTAA
CAGCAAATATGCTGATGTTTTCGCTGACAATGATAATAAAGTTCTTATCTTCGCTATCAATTATAATGTC
CTCAGAATTATGAGCG**GAATGG**GAGGCTTAGCCTACTCAAACTGA

150 3331 **4.5**

Pleurochrysis sp. Polinton-like virus isolate PleuroPLV, partial genome GenBank: KY203338.1: 11562-12602 minor capsid protein

ATGAACGCGCCTGGATACCATATTGTTGACCAGACCAATGTGTATATCAATACCGGCTCTGGACTGAACC
CAG**ATTCAA**CGGG**AGATGA**TA**TCAAG**CTGACGTTTAACGATGTGAACGTGAAATGCGGAGACAACCAGTT
C**TTCAAG**CTCACGTTAAAACAGTTTAACATGTACAAGTCATGGACGAACG**TGAATG**CGAATAACT**CCATT**
TTCGTTCTAAACATTGGCGGAT**CATTC**TTCACCGAGTCGCTTACACAGCGGAACTATTCGAGTTTTCGTG
ATCTGGTGAC**TGAAT**TCTCCTCAATTGTTGATT**TGGTACTG**ATTAGAGAGGGAGCCTACACTACATCGTC
AGT**TACTGTGAAT**AACCCTAACACAGCAAGCGATATATCCGGTAACGTGGAATTCGATTTTTCGATATCG
ACCACAGAGGCTCACGG**ATTCA**GTGACGGAGAAA**TCAAG**CTGCATGC**GAATG**TGTCATTGGGTGACTTCT
ACAG**ACTGC**TTGGGG**TCAAG**CGTAGCAAGGTGGACAACGATGTGGACGGTTGGC**AGATGA**CCGCGCCTGA
CGCGAACACTCTGCGCTTCAGAGGATATTACAACTGTCAGCTGA**GTACTG**AGAGCTACGTCTACCTACAT
TGCTCGACATCGAGCTGCAGTATCGCTACGAGGAACTACACTCTCGGCTCCAAGGACAGCCCTGGACCCC
AGCTCGATCATAGCACGATCCTGGCT**AAGAT**CCCG**ATTCAA**AATAACGTCTTGGCATATGACTCCCAGTC
AGACGAGTTCGTAACGCTCCTGAAAAACA**TCAAG**CAGATCTCGTATATGGAACTCGACCTTAGGGACAGT
AAGGGCCGT**AAGAT**CCCACTCATCG**CTGCC**GGTCAGAACAC**AGATG**GGAACCGCT**CATTCGAATG**CCTAC
TCGAAATCCAAACG**ATTCA**GCGGACCGGGTCTCACCAAGGTCACTTTAAGTCTCCGG**AAGATGACAAGA**C
ATATGCGCATCACAACACTCGACCCCTCGTCTTCCA**CAAGA**ACGGACAAACTGGA**TACTG**A

44 1037 **4.2**

Cafeteria virus-dependent mavirus, complete genome GenBank: KU052222.1: 24414-25325 minor capsid protein

ATGAAACAGTATATTTGGCTTA**ATGAA**ACTA**TCAAGA**GTAATAAACAATTAGCTGGACCGCGGGGCTCTT
ATAAAAGACCCGTTAGTGTTGATATTTTTCGTTCTTCTACAATTTTAGACCCTGATAAAAATTATCTCTT
AATTGTTGAAGAGTTCCATCTCCATAAAATAAGACTT**CCATT**ATTTAAACCCGCGGGACATGACTA**TCAA**
GTAGGAATTTTTAACCGTTCCACC**GATGAA**ATTATGGGAGTACGTGAGGTTGATTTTAGTACCTTTGT**AG**
ATGAAGATGGGTACATGTACGATTATGTTGATGTAGGTACC**GCCATT**A**ATGAA**ACTTTAGCTGGATTATG
TGACGGTATCATAGGAGAAGAAGACATACCCGTTTTTAGTTTTAATAAACATAGTAAAAAATTTGAAATC
ACCACGACTGAAAATTTTAGAAACGGTCATTTTATTATGTTTAAT**GATGA**CATGAGAGTAGACTTTAACT
CTTT**TGAAT**TTGACGACATAGACGAAGAGT**ATTCA**TTAGTTATATTAA**ATGAAGATG**TAGAAACA**CAAGA**
TGCTTCCACTTTAGAATTTTTAACCCCTATATCGCATATTGTTATAGAATCTAACGACCTCCCCGTATCT
T**ATGAAT**TACTCCC**TTCAA**TATCTAAAAACACCACTATATCAGATAACACGGGGGTTTTCCTTACTAATT
ATAAATATTTACAACAAAACAA**TCAAGAT**TATAACAGCATTTTATTTAGGGTTGAAAATTCCTCTAATAA
ATACCATAATATTTTACAAACTAATTTTAACCGTTTTAATTTATCCTTTACCATATACGATTATGATA**AT**
GAAAAACACCCTTTAACTCTTCTTCCTCAAACAGTAATACAACTTAAGCTTTTATT**TGAAT**CAATAGATTAA

33 908 **3.6**

Cafeteria virus-dependent virophage mavirus, complete genome GenBank: KU052222.1: 25440-27260 putative major capsid protein

ATGAATACACCCCCAGAACTCGA**TACTG**TTTTACAAGCTCCATATGCTTATAATTGGCCCACTTCTAAAA
ATGTTAAAATTGCTTCTAGAATAGG**CATTC**CATACTCAACAT**TTCAA**ACA**ATTCAA**CCCGTATC**AGATG**C
ACCTAAT**AATGGTA**TAGGTCAAATAACTTTTAATCAACCTCTTGGAAATTTAACAGGAGGTGCTCCACGT
TTAAGAGTAAGTTT**TACTGC**TGAAATTAAAAATATTTTAGCTG**ATTCA**TCTTTAA**AAGAT**CAAATAGGCC
TGAAATCTTTTCCAGTTAATAGAAGTATACCTGTTGCAGTAATTAAT**ATGAATGGTA**AAACTTTTACCAG
TTACCCCGCACAATTAATTAAACTTCATCAATATAATGCGGATCCTCTAGAACTTGCTTTGCTTTCTCCC
TGCTCTGATGT**AGATGAAT**ATAATAAAATAAAAGCCGTTTCT**ATGAAT**AACCCTTATAGACAA**GGTAC**AG
AATCTACCGATAGCC**GAATG**TCTCGCGGTTTAGGTTGTAATTATGCCTATTATATCCACCCACGGGCAGC
AGGTAGCACATCCGTAAAAATTGATTTTGTAGT**AGATGAA**GCACTTGTAGCCAATCCCACACAATATAAA
AATATTAAAGACCCAGTACCTTTTAGAAACCTGAACACATTTAAAGTCATATT**AGATG**GACAATTTAAAC
CTGAAAATATGAT**TGGTA**TTGCG**GATGA**TGTTAAACTTGTGGCGGGTAAAGCAGATTTTGAAGTGGATAT
AACCGGATTTAAAATTAATATGCTTG**TTCAA**AACTGGGTCGCCCCTCTTGAAATTGGTGA**CATTC**CTAAA

ACTATAATATATAACACCCCGTTAATTTCATTAGAAGGTAATATAAGTTCGATGTGCCTCAATACAAAGG
ATCCTTACGGTATCCCTGGTGAACGAAATAAACACATATTAACAACTCATAGTATGGCAATGAATAATGT
CCCTTCAATGTTTGCTGTTATGGTTTCACAAGAAACACCAACAAAAAGTTTGCCCCTGATCAACTTGCT
GGAATTATTGGATTAGAAATTAAGGTGGATTCTGATGTTGGTATTTTTAGAGAACTTGAACAACAACAAC
TGTACGAATTATCTTCATCTAATGGATATAATAAAGATTTTCATGCTTTAGTGGTGCTCTGGCTAATGG
CTTGACTGTGGCAGATCCCGCTGTGGCTGCTGGTAACAAATTTAAAGAAGCTATATTTGGTGCTGGTTCT
GTAATTTTCTTCCGTCCTTCCGATCTGGGATTAAAAGATTATAATGTTATGGCTAATGCTAATAAGTCCA
TTAATATGCAGGTACAAGCAACATTTGTCACCCCTGAAGCTGCGGGTACTGGTGCTCATTATAAATTAGA
AGTGTTTTCAATTAGAGATAATCTCACCTACAGCTTTGAAGATGGTACCTTTATGGATGATTTAACCCTT
TACACTCCCGACCAACTATTACGAAGCCCCCTAAAACTTACTGATGACAATAACAAATTAATGCGGGTTA
TGGGCGGGTCCTTTATGGGTGATGTTATGACTAATTTTAACCATATGGCAGCTCATCCTGTGACTAAAAC
GGTGACTAAACTTTTAAGAAATGCGGGACCATTAAAAGATTATGCGGGAGATGGTACAATGATGGGAAAC
ATTGCCTCCGTTTATGGTTATGGTAAAAAAAAAACAACCACTAGAAAAAAGAAAGGTGGAGAAATTGTTT
TACTAGGATCAGGAAAAAAAGGCGGTAAAAAATTATCAGATAAACAACTTCACGACTTAAGAAACCTCTAA

73 1817 4

Qinghai Lake virophage, complete genome GenBank: KJ854379.1: c15342-13609 major capsid protein (MCP)

ATGGCATCCGCAGACTTTAAAACTACTCTCATCAAGCAATCGACCCTTGCTGATATTACCGACCAATTGG
ACTACGCAGTTCTAAGTGGTGCTTCCAGCAATACTTACCAGCAATTTAACGCTGTATCAACATCCGCATC
AAGTATTACCTTTTCTTTACAGGTACCTTCTGAGTCTATAGTCGTATCAAGAGAAATATTGATAAGAACC
GACATTAATTTTACTGTTGATGTAACAAATGTTCCTATCGGTGCAAAGGCATTTGACTACGGAAAAACCG
ATGCTCTTCAAGCATTCCCTCTTAATAGTTTATTTAGCACTTCATCCGCACAAATCAACAACACTAATGT
CTCTGTTAATACCCAAGATGTATTGCCTCAATTGTTAAGACTTAACAATAGCAGAGAACTTTATAAATAT
AACTCATTTTGTCCTTCTCTACCCGATCAAGCATACCATTCATATGCCGATGGTGTTGATGCTTTTAATA
ACCCATTAGGGGCTTACTCTAATGCATCTTACGATGTCGACCTTATTCCTAGGGGAGCATTTAAACTTAA
AACATTTTCATATGTATATACTCCTGTCGCTGGTGGCTCTCCTGACACTCTTGTCTCTGCCAATGTTGGT
GATGTTTGGAAGATATCTATCACAGTTGAAGTATGTGAACCTATTATTGGTCTCTCACCATTTGTATTTG
GAGACTGCGATTACAATAAGCAAGGTTTAGTTGGTATTAACGCCTTGTCCTTTGTCTTCAATATTGATAG
TAGTTGTAAGAGATTTTTCAGTTGCTCTCCTTCACCATCAAGAGACATATCTGTTAGTTTAGGAACTTCT
GCCAATCCTCAACCATTCGAGAAATGTTCCCTGTTGGTTAATTTCCTTAGTACTCAACCATCCGACTTGA
TTCCTGCTCGTAATGTAGTACCATACCACGATTTTCCAAGATTTTTATCCCTTCAATCTGCTACAAATCC
ACTTGGTGCTGGTGCTTCTACCACAGTTAATTCTCAAAACATTCAAATTAATCAATTGCCCGATTACTTT
ATCGTCTGTGCTAGAAAACCAATGTCTCAACAAACCATTAAGGATAGTTCTTCCTTCGCTGTTATTAATA
GTATAAGTGTTAATCTTAACAATGCATCGGGTCTTCTTTCATCTGCTACCGCCCAAGACCTTTGGAGAAT
TTCTGAGCGTAATGGTTCTACTCAAAACTGGCTAGAATTTAGCGGTAAGGCATCAAACGAAGGAAACAAC
GATATCATCGCCACTGATATTGCTACAACTGGTTCGCTACTTATTTTGTCTCCTCCATTTGACTTGTCTC
TCCCTGACTACTTAACATCAGGAAGCATCGGTCAATACAACTTCCAACTTCAAATGAATATCACCAACCA
ATCTGTCGATGCTTACACTCCTGAAATTTGTATAATCTGTGTTAATAGTGGTGTATTTACTACGGTTGCT
GGTTCATCAAGCATCTTTACTGGTATCCTTACTAAACAAATGGTTCTTGATAGCAAGACAACTGAGGCAA
TTGACCCTGTATCATCCGTTCTATATAAGAGAATGATTGGTGGAAATCTTGGAAACAGAATTGCTACTGC
CGTTAAGAAAATGCCATTCCTAAGAGATTTAGCAGGAAAATTGAGAGAAATGGTAGGTATGGGTGTCCCA
GTTGGAGGAGGTATAATGTCCGCTGGAGGAAAATTGGATGCTCTCTGTGCTTAA

64 1730 3.7

Miers Valley soil virophage partial MCP gene for coat protein, segment MCP, isolate Miers Valley soil GenBank: LT221025.1

ATAAAATACCCACTGTATAGTCCAAATAGGATTACCTCAACATCAAATCTTGCACCTGTTGCTGCTCTAG
CAAGTATTTCTCTGACTTTACAACCATTGACTCTTACTCAAATTCCTTATTGTGTTTATTTATGGGTTGG
TCGATCAGATGCCGAAACATTAGCAAATGTTAATAATATAGTTACATCAACCGATACTAACTTTAGAATT
GATTCATTGAATATGACTTGGGGTACTGTACAAGTTATGAGTTCGTCTGGTCCATTAGATCATTATAATG
TTTCTATAAAGAATGGTTTAAAGAACGTCTCATTTACAGAATATTATGGAGAGACCAACTCGTTTTCACA
ATCTATCGCCGCTCCTTCTACTAAAATTGGATTAACTGGAAGTGTAATAAGATTAATACCTGGTATTGAT
TTTGCATTAGCGGATACCTCTCTAGCACCTGGAGTGAATGCACAAACTACTTTCCAAATTAGTGTAGTTG
CAACAAATCTTAATAATAAATATGCTGTAACACCTCAACTGAATGTTTTATTTGTAGATGAAGGATACGT
TGATATCCAAGGTGATTTAGCAACTAAGCGTATCGGAGTTATTCCTCGTGAAGCTGTTATCCACATGCCA
GCAAATGATAGTTATAAGGCTGAAGATTTAAGATATTTTGGTGGAGCACCATCTTTCTCTAGTCGCTTTA
ATGTGTTTTCTAAAAATTTAGCAAATCGAGTTAAAGATTTTGATATTCCTGGTAAAGCAAGACAAGCCAA
TCGTTTTTTACAAGACAAAAAAGTAATCAGTAGAGGATTAGATGCAGCTTCGTGGTTACCAACAAGATTT
AGTGGTAATTTACAAAGATTATCAGGTGTAGCTCGTGATTTTGGTTATGGAGGATGTGGAGATGATATGG

GTGCTTATTTACCTGAAAATATAC**ATGAAGAT**AGAGGAGGTTATTTGGCTGTTAATAA**TCAAGAT**AATAT
GGG**TGGTA**GATCTATTCCTCTTGATAGATTACGCAATGCTATGTGTAAACGATAA

42 1031 **4.1**

Organic Lake virophage, complete genome GenBank: HQ704801.1: 7791-9518 major capsid protein

ATGTCTAGCGAACTTTCTACCTTTCTAGTC**CAAGAT**CCACGTATCAGTCGTATCACCAGCGATG**TTCAAG**
TTGCGGTGA**AAGATGGT**CCGGCTTCGAGCGTGGTCCAAGGCTACCCTACTAACTCGAACAGCTCGTCCAC
TACCCTT**TTCAA**TGTCAATGTCCCAAGCGAAAACACTCTTGTAGACCGCAACCTTCGTGTCCAA**GGTAC**G
ATCCAATGTGTTATGGAATTAACTGTAGGCACAACTGAGATTAATGTCGATACTTTACAAATTGTACCAT
CTGCTTTTCCCTTAAA**TCAAG**CAC**TTCAA**TCGGCATCTCTTACCCTTAACAATGCTAAAGTCTCGG**TTCA**
ATCGGCGGATATTCTCAACGTTATAACGAAACAATACCATCAACGATTTTTAAGCAAACACATCCAAACT
ACTCCATCT**ATGGTA**GACAAATATTACGCCAAAGCGGTCGATGCTATGTCGGAGAACAAACCCTCGGCGT
GGGGTGCTGGTGT**TGAATCTGCC**GAAAAAGACAGCGACACCGCTGGTCGTGCCGACTCTAGCATCAGTTA
CGTTGTCTACACAAGTGCTGGTGTTGCGGTTGCCTCAGGAGCTAACTTGACTGTCGCTACAACCTATTAT
GTAGAAATCTCTCTTGATG**TGAATGAA**CCCATATTAG**GAATGCCA**ACTCT**TGAAT**TTAAAGAAGAAGAGT
CGTCTTATTTAGGCATTAACAACCTCGAACTTGTGC**TTCAA**TACAATGACTTTAA**GAATG**TATTTAATGT
AAACAAAGAACTCGTATTGTCTTTCTCGAGTGGAGTTAAATTTGGAACTCAGGCGTCCACTCTTTTCTTG
A**AAGATGA**CGCCCGACTTATGGCTCGGTATATTAGTCTTCATCCATCCCAATATGCAAAACTCAATGCTA
AAAATATCCTTCCATATGACGAATTTGTAGCCTACAAAACAACCCTTTCTCTTCCGTCGG**ATGGT**GTAGG
TGTA**AATGG**GGCTATGACGAACGTCATTTCTATGCG**TCAAG**TGCCTGA**CAAGAT**TTACCTTG**TGGTAC**GC
CCTCAATATCGGTCACAAAAAGCATATTGGTCAAATAACCTCAGCTACCCAATCTC**TCAAG**TTAATATCA
CT**TTCAA**CAACAAAGCGGGACTTCTATCTGA**AATGG**ATGCTTATTCGCTTTACCAAATGTCCCGCCGT**AA**
TGGTTCTCAACAAACTTG**GAATG**AGTTCCGTGGCGTGGTTCGATCTGGGAACGGAGGGAA**ATTCA**CTTCT
TTAGGAAGTATTATCGTCATCGATCCGGTCCGTGATTTAGGATTGAGCGATATGCTTTCGAGTTCTAGTC
TTGGACAATTTGGTTTCCAAGCGATTGTGACTACAGAACC**AATGG**AAGGTATTGCTAACTCGGCCTTAGC
CAATATTGAACTTTGTGTTCTTGCGAACT**ATGGT**GGTGTAATGAT**TACTG**AAAGGGGATCGAGTGCGAC**G**
ATGAGTGGATTACTCACCAAATCAGAAGTATTAGAAGCGAAAGAAAAAGGAACTTCCAAAATTGATT**ATG**
AAGATGTCGAAGCTATGTCGGGCGGTAATCTT**ATGAA**GAAAGGCGTGACCGCTCTCGGCGATGTGCTAAA
ACGCAACAAAGGAGCAATCGGTAAAGGAGTTGCCTCAGCGGTAGAGTCGGCTATTGGTGGAGCAAAAACG
TCGTCGT**ATTCAA**CGTCTGGCGGTTCTCGGCTCTCCAAATATATGTAA

52 1724 **3**

Yellowstone Lake virophage 6, complete genome NCBI Reference Sequence: NC_028270.1: c6005-4254 putative major capsid protein

ATGAGCGACTTAAAAACAGTTTTAATTAATG**ATTCAAGA**ATTG**AAGAT**ATAAGCGGTGAAATTGGGTTTT
CCGTGCTAGGCGGTCCTCAACAATCCACGT**TTCAA**CCACAACAGGCGAATACATCATCTAATACTTC**CCA**
TTCATGGCAAATTAATGTACCTAGTGAAAATATTGTCGTTGATAGAAGAATTTTACTTGA**TACTG**ATGTA
GCATTTACAATTACAATTACAAATGTTCCAGTAGGTCAGGTAGCGTTTAATTGGGGTAAAACTG**ATGGT**T
TTGGACCTTAT**CCATT**AAATAACTGTTATCTTACTCAAAATGTTTTAATTAATAATTGTGGTTTATCTGT
AAATACC**CAAGAT**ATAATCGCCCCTTTACTTCGTT**TGAATG**ACCAAAAAATACACACTTATTATGATGGA
TGGACTCCATC**AATGGT**T**GATGA**TACTTATTATAATTATTCTGATGCTGT**AGATG**CTAACAACAACC**CCA**
TTGGAACTGTTGTTAATAATAGTATGGATAATAAA**ATGAA**ACCACGAGGATGTCATACTATTAAATCTAT
GTTAATTAAACATTATTTAAGC**ACTGC**ACCGCTTGTTATTGCTGATGCTTCTGTTATTTCTACTAATGTA
GCGGATACTTGGACTATTGATTTAACTACTCATTT**TACTG**AACCTTTACTTTTTCTATCTCCTTTTATAT
CTAATCCGTGCGTATCTAATGAGGCTGGATTTTTAGGAATTAATAATTTTTC**ATTCAA**TTTTACTATTGA
TTCTAATTTACGTCGTGTATTTAGAACTTCTAATGCTTGGACAAATACAT**ATTCATTCA**CAA**ATTCAACT**
GCATTTACTAAATTACAAATTCTT**ATGAAT**TATCTAAGTGTCCAACCTTCCCAATATGCTAAAATATCCG
CTCGTAATGTTTTACCACTTTTACAATATGATAGATATATCGCTAACTCTAGTAATACTACCGCTAATAT
TCCACCTGCTGTTGTTGGTGCTAATAATAGTATTACTTTAACCTCAACCCCCTATACCTCATCTAATATT
GCAT**TGAAT**CAAATCCCAGACA**CCATT**TTAATTTTTGGTCGTATTCCAGTATCTAGTCAAACCGCTAAAA
ATAGTGATG**CATTC**TATAAAATTGATTCTATTTCTATTAAC**TTCAA**TAACGTAGCAGGTATTCTTAGTAG
TGCTACACCTCACGAATTATGGGTATTATCTCAAAAGGCGGGTTCATCTCAATCTTGG**GATGAAT**TTAGT
GGACAAGTTAATAC**TTCAAG**TGCATCAGGTGCAAG**TTCAA**TTATTCCAACAACTGG**TTCAA**TTCTCGTTC
TATCACCATC**AATGGTATTCAA**TTTACC**TGAAT**ACCTCAGTAGTTCCAGTTTAGGAAGTTATAATTTACA
ATTTACTATTAATTTATCAAATCAAAGTTTAACTGCTTCTGCTATTGAAATCGTTGTTGTTACTATTACA
TCAGGATACATCACAACACA**TCAAG**GAACTACTCAATCTTTTACAGGTGTAT**TGAAT**AAAG**AGATGGTA**A
TGAGTGCGAAAGAAGGGTCGGCTGTTCCTCGTCTCGCACAATCTGATT**ATGAA**CGCCTTGTTGGTGGTGT

AAGAGATAATAGAGGAGTAATGAATATGATGAAACAATTTAAAAATAGACGTATGGGCTCAACTTCTGGC
GGTGTAATGTCTGGTGGTGTAATGTCTGGTGGTATGCAAACTGGTAGTCGTCTAGGAAAATATATTCGTTAA

61 1748 3.5

Yellowstone Lake virophage 7, complete genome NCBI Reference Sequence: NC_028257.1: c17093-15336 putative major capsid protein

ATGGCAAATAATTCTATGAAAGTTATGAAGGTAATTGATGAGCGTATTAATGTTGCACAGGCTCCAGTGC
TTATTGTAAATGAATCAACTCCAAGTGTGCAATATTCAACTATTAATGTTGCTGGTAATGCTTCACTTAC
GCCTACTTTTACAATTCCGTGTCCTCCAAATCAAGGGTTAGATAGAACCGTGACGATGGCATTCACTGTT
CAATTTTTAATTACTGGAACTGATTTGGAACAATTTCAAGCAAATCCAGCAATTGCATTGAGAGCGTGGC
CATTACATAATGCATGCACATCTATGAATATTAATTTAGGTAATGCCGGTATTGGTATTAATCCTAGTCA
ATATGCTGCGGCATTATTAACACAATGGAATTGTGATAGTCATACACAAGCAACTGATTTATCATCATTT
CCATCGGCTCCAGATAGATATTCAAGTTATGCGGCCGCGGCATTATCAACTACATCACCATTTGATTCTG
ATTTTGCAGCTGTAAATTCTGATTATGCTAATACATCAAGAACTGGACAAATTACATCTATTGAATATTA
TGATGGCACATATGTATTGGGTGGAACTGGAGCTACTCCAGTTGCATGGGGTGCTCCTGCAACTCAAATG
GTAATTACTGCAACGGTTTATGAACCTATTGCCGCTTCTCCTTTTGTATATACTGGAATTAGAAATCCTA
AAAAAGCATTTTTTGGATTAGGAAACGTCACGGTGAGTTTGAGCTTTAGTAATTTACAAAGAATGTTAAG
TTATTATATTCCTGGAACTGCTACAATTACATCAATTCAAGGACAATTTGCATCTCAATCTTTGTTAGTT
TCTTACATATCAGCATTTGAAGATAGTGTATCAAATTATGTATCTCCTATGGCATATAATTATGCAACTC
TTAGAACTGCAACATCTGTTTTTACGGTTGGGGCTTCAACTGGTGGGGTAATGGCATCTGCAACTGCTAA
TTCTGCTACAATGCAATTAAGTGTTGTTCCATCACACTTTTTAATATGGGCAACGCCTCCATTGAATTTT
GTTGAATCGCAAACACAATCATTACCTGATTTTTGTTTTACTCTTAGTAATGCAACTATTAATTTTGCTG
GTAAAGATAACATTTTAGGAACTAACTGTACTGCATATCAACTATATAATATTAGTAAAAAAAATGGTTC
AAATACTTCATTTTCGCAATGGACTGGAGCACAAATTTTATCTTCTTCAACCACAAACTAATCCAGCAAAT
CCACCACAATATTACGGTGGGGGAGTTTTAATTTTAAAAACGAGTGAAGACCTTCGCCTCCCTGCAACGA
GTTGCGCTGGTATGAATCGTGCAATTAACTTTCAAATTCAAGTGCAATGCACAAATTTAACTGATATCGC
ATTTGGTGCAAATTGTCAATTAAATGTGTTGGCAATTACTGATGGTGTTTGTCTTACTCAACATGGTTCT
CGTGTTCAACTTATTGAAGGTGGTATTACTGAAGATATTTATAATAATGCTCCAGTTGTTTCTGGATTAG
AAGAACTGGCCGTAAAAAGTTATACAAATAAAAATGGATATTCTGGAGGCAGTTGGGCATCATTTAAAAA
TGATATGTCAAAATTTGCTGATTATATATCTCCGGTCACGAAACCTATTATTGGAGCATTGACAAATAAA
GCGGTTGCTGGAATTGAATCCGGCGCTGGTCGTATGAATCGTGGTAAAATTCGTGGATTATTGAAAGACA
TGTATTAA

79 1754 4.5

Dishui Lake virophage isolate 5 putative major capsid protein gene, complete cds GenBank: KU245928.1

ATGGCTATGCTTCTGCTCTCGGACCATTTCCATTCCATTCTCTCTGTAATACCGTCCAAGCAACCATTAA
TAACAATACCGTTTCCCAGAATATGCGAGATGTTATGTATCAGTTTCTCCGCTTTAATGACCGCAGAGAA
CTTGCACGATACAATAACGCTTGTCCCACCATGTATGACTCCTATTTTAATTATCAAGATTGTCTCGGCA
CTAATAACAACCCTCTCGAATAATGTAAGCAACGACCAAGATTTTCAACCCAAGAGGTGCTTTCGTTCTTG
ACAGCATTACTGGTAATGAACCCAAAGGTTCTATTGCTTCCAACACTCGTAATGTGATCTTGACCTTTAC
TACAACTGAACCTCTTATGCTTTCACCTTTTATTTGGTGTGATCCTAAGAGCAACAATCAAGGTATGTAT
GGCGTTCAAACACTCAACTTTGTCTTCAATTCTTGGTCTTCCAAATCGTATTGTTCGTCTTGCTAATAAAG
ATTTATTTGACGGCGGTGTTGCTACTATTTCTCTTCCCAATCAAGCAATTAACAACGCACAATTACTCAT
GGAATTTTATACAAGACAGCCAAGCGATTTGGTTTCCAGCCGTAATGTTGTCCCGTTCGCAGAATATCCC
CGTTATTTAACCCCTATGACTTCTCTTGGTGCTCTTGCTGCCTATAACCCTCAAACTCCATTAGTTATTC
CCTCTGTATCTGTAAATTTTCAGTCAATTCAGTTGAATTCTGTTCCTGATAAACTTATCATTTGTTGCCG
TAAAGTTCTCGCACAGCAAGACGCCTTTGATAGTGATAGTTTCTTACCTATTACCAATATAAGTATCAAT
TTCAACAACAAAGCAGGTCTGTTGTCAAGTGCTCGTCAGTATGATCTATGGCGTATGAGTGTGGAATCAG
GAAGCAACCAAACATGGGCGGAATTTACGGGTTTCGCAAATATTGGAAATAATGTTCCTCTGTCAAATGG
TCCTTCGTCAGGTGCTAAGAGAGTTCCAACTTGTGGATCTGTTCTTTGTTTAGAGATGGGGAGACATGTA
GAATTAGACGATGTTTATGCCCCAGGAAGTATCGGGGCATTTCAACTTCAATTCCAAACTACATTTCAAA
ACAATACCCTTGAAGCAATTGACGACCAAAACAATCAGTATGAGATCGTGCTTATTACCATGAATAGCGG
TGTCTTCACAATTGAGCGTGGAACTTCACAGACTTATACTGCTATTCTGTCCCGTGCCGATGTGCTTTCT
GTTTCTTCTCAACCTTCTCATTCCAAGTCTTCTGTTGCTCGTCTTGTTGGTGGATCATGGGAAGATTCAT
TCAAATCCCTTTGTTCCTCAATTGCTCCTTATGCTGGTAAAGCAGAAAAAGTTAAAGATCTAATAATGGG
TGAAGGTGCGTCAGGTGGCGGTCAGTCAGGCGGTCGTATGAGAAAACATCTCGCCATGTAA

51 1457 3.5

Dishui Lake virophage isolate 6 putative major capsid protein gene, complete cds GenBank: KU245929.1

ATGTCTTCCAGCGATTTTGAGAAAGTTTGCGTC**CAAGA**CGATGTGCTTAACAC**TACTG**ACAAAGTGCGAT
ACGCAGTATTTAAGGGAGCACAGAATATTACTCCCGCACAATACAAC**GCCATT**TCTACAAGCA**ATTCAAG**
TATTACTTGGAAT**ATTCA**GT**TGCCA**AG**TGAAT**C**TACTG**TATTCTCTCGTAGGATC**ATGGTA**AGGGCAA**CC**
ATTTCAGTTAAAATTACGGGTGATTTCGCCGCCACCGCTCCCAACGGGTCTGTGC**TGGTA**AATT**ATGGT**T
ATGCTTCCGCATTAGGTCCTTTCCCCC**TTCAA**TCTCTTTGTAATACT**ATTCA**GGCAACTATTAACAATAA
CACAGTATCACAAAATCAACG**AGATG**TCATGTTCCAGTTGCTACGATTTAATGACCGCAGGGAACT**TGCC**
AAATATAACAACTCAACTCCCAACATGTATGATTCTTATTTAGATTA**TCAAGATG**CTCTTGGAACTAATA
ACAACCCACTTGGTGGTTGGAATAATGTAAGCAACGAC**CAAGAT**T**TTCAA**C**CAAGA**GGTGCTTTCGTTCT
TGACAGCAT**TACTGGTAATGAA**CCCAAAGGTTCTATTGCTTCCAACACTCGTAATGTGATCTTGACCTTT
ACTACAACTGAACCTCTTATGCTTTCACCTTTTATTTGGTGTGATCCTAAGAGCAACA**ATCAAG**GTATGT
ATGGCG**TTCAA**ACMCTCAACTTTGTC**TTCAA**TCTTGGTCTTCCAAMTCGTATTGTTCGTCTTGCTAATA**A**
AGATTTATTTGACGGCGGTGTTGCTACTATTTCTCTTCCCAA**TCAAG**CAATTAACAACGCACAATTACTC
ATGGAATTTTATA**CAAGA**CAGCCAAGCGATTTGGTTTCCAGCCGTAATGTTGTCCCGTTCGCAGAATATA
CCCGTTATTTAACCCCTATGACTTCTCTTGGTGCTCTTG**CTGCC**TATAACCCTCAAACT**CCATT**AGTTAT
TCCCTCTGTATCTGTAAATTTTCAGTCA**ATTCA**GT**TGAAT**TCTGTTCCTGATAAACTTATCATTTGTTGC
CGTAAAGTTCTCGCACAG**CAAGA**CGCCTTTGATAGTGATAGTTTCTTACCTATTACCAATATAAGTATCA
AT**TTCAA**CAACAAAGCAGGTCTGTTG**TCAAG**TGCTCGTCAGTATGATCTATGGCGTATGAGTGTGGAATC
AGGAAGCAACCAAACATGGGCGGAATTTACGGGTTTCGCAAATATTGGAAATAATGTTCCTCTGTCA**AAT**
GGTCCTTCGTCAGGTGCTAAGAGAGTTCCAACTTGTGGATCTGTTCTTTGTTTAG**AGATG**GGGAGACATG
TAGAATTAGACGATGTTTATGCCCCAGGAAGTATCGGGGCAT**TTCAA**C**TTCAA**TTCCAAACTACAT**TTCA**
AAACAATACCCTTGAAGCAATTGACGACCAAAACAATCAGTATGAGATCGTGCTTATTACC**ATGAAT**AGC
GGTGTCTTCACAATTGAGCGTGGAACTTCACAGACTTA**TACTGC**TATTCTGTCCCGTGCCGATGTGCTTT
CTGTTTCTTCTCAACCTTCT**CATTC**CAAGTCTTCTGTTGCTCGTCTTGTTGGTGGATCATGGG**AAGAT**T**C**
ATTCAAATCCCTTTGTTCCTCAATTGCTCCTTATGC**TGGTA**AAGCAGAAAAAGTTA**AAGAT**CTAAT**AATG**
GGTGAAGGTGCGTCAGGTGGCGGTCAGTCAGGCGGTCGTATGAGAAAACATCTC**GCCAT**GTAA

65 1739 **3.7**

Observed mean proximity: 59.8/15≈4 Expected mean proximity: 2.15±0.8* (* 90%-confidence interval calculated from the minimal size of ligases, 908). Data from https://www.ncbi.nlm.nih.gov/nuccore/CP021507.1?report=genbank

Standard deviation s for the minimal ligase: s=0.48. Then, the number X of standard deviations between observed and expected mean proximities equals:

$$X = (4-2.15)/0.48 = 3.9$$

and, if the observed mean proximity O is a Gaussian random variable, following the Table of the distribution function below, we have :

$$\text{Proba}(O \geq 0.04) = \text{Proba}(X \geq 3.9) \approx 5\ 10^{-5}$$

Table of the distribution ∏(t)=P(X≥t) of the N(0,1) random variable X

t	3	3,1	3,2	3,3	3,4	3,5	3,6	3,8	4	4,5
∏(t)	0,998650	0,999032	0,999313	0,999517	0,999663	0,999767	0,999841	0,999928	0,999968	0,999997
